# Heteroleptic Cr^4+^ molecular color center candidates

**DOI:** 10.1039/d6sc05602d

**Published:** 2026-09-18

**Authors:** Cindy Serena Ngompe Massado, Rianna B. Greer, Arailym Kairalapova, Andrew Ozarowski, Kelsey A. Collins, Majed S. Fataftah, Noah Mendelson, Chloe A. Washabaugh, Daniel W. Laorenza, Michael K. Wojnar, David D. Awschalom, Timothy C. Berkelbach, Danna E. Freedman

**Affiliations:** a Department of Chemistry, Massachusetts Institute of Technology Cambridge Massachusetts 02139 USA danna@mit.edu; b Department of Chemistry, Columbia University New York New York 10027 USA tim.berkelbach@gmail.com; c National High Magnetic Field Laboratory, Florida State University Tallahassee Florida 32310 USA; d Department of Chemistry, Northwestern University Evanston Illinois 60208 USA; e Pritzker School of Molecular Engineering, University of Chicago Chicago Illinois 60637 USA awsch@uchicago.edu; f Department of Physics, University of Chicago Chicago Illinois 60637 USA; g Initiative for Computational Catalysis, Flatiron Institute New York New York 10010 USA

## Abstract

Most quantum sensing experiments require the creation of a spin-optical interface, where optical light can polarize and read out information from spin sublevels. This combination creates a sensor capable of detection down to the single-spin limit. Heteroleptic coordination complexes are promising architectures for enabling positional control of spins in quantum sensing experiments. Towards this end, we use complementary magnetic and optical spectroscopies supported by multiconfigurational electronic structure calculations to establish the first generation of heteroleptic *S* = 1 Cr^4+^ complexes as molecular color center candidates. This family of trigonal bipyramidal tris[2-(trimethylsilylamido)ethyl]amine chromium–halide complexes, referenced here as ^TMS^trenCrX (X = F, Cl, Br, and I, respectively known as 1, 2, 3, and 4), exhibits highly modular magnetic and optical properties. 1–4 display largely axial zero-field splitting (ZFS) characterized by high-field, high-frequency continuous-wave electron paramagnetic resonance (HFHF cw-EPR) spectroscopy, where the axial ZFS parameter *D* continually increases from +5.194(4) cm^−1^ (1) to +7.34(1) cm^−1^ (4) as halide donor strength weakens. 1–4 also display near-infrared spin-flip photoluminescence (PL) ranging from 959.57(1) to 1043.81(1) nm. These PL spectra exhibit traits critical for enabling optical addressability, such as optical linewidths narrow enough to rival the magnitude of ZFS. A dilute sample of 4 in its Ti^4+^ diamagnetic host (4′) even displays optically resolvable spin sublevels under zero applied magnetic field; using variable-field PL spectroscopy, we optically determine its ZFS parameters. These results will lead toward the realization of well-defined spin-analyte interactions and subsequent spatial control of molecular color centers, wherein tunability is maintained through the donor at the axial coordination site.

## Abstract 2

Increasing halide size in a series of emissive trigonal bipyramidal Cr^4+^ complexes increases magnetic anisotropy, decreases emission energy, and increases D-to-linewidth ratios, enabling optical determination of zero-field splitting parameters.

## Graphical abstract

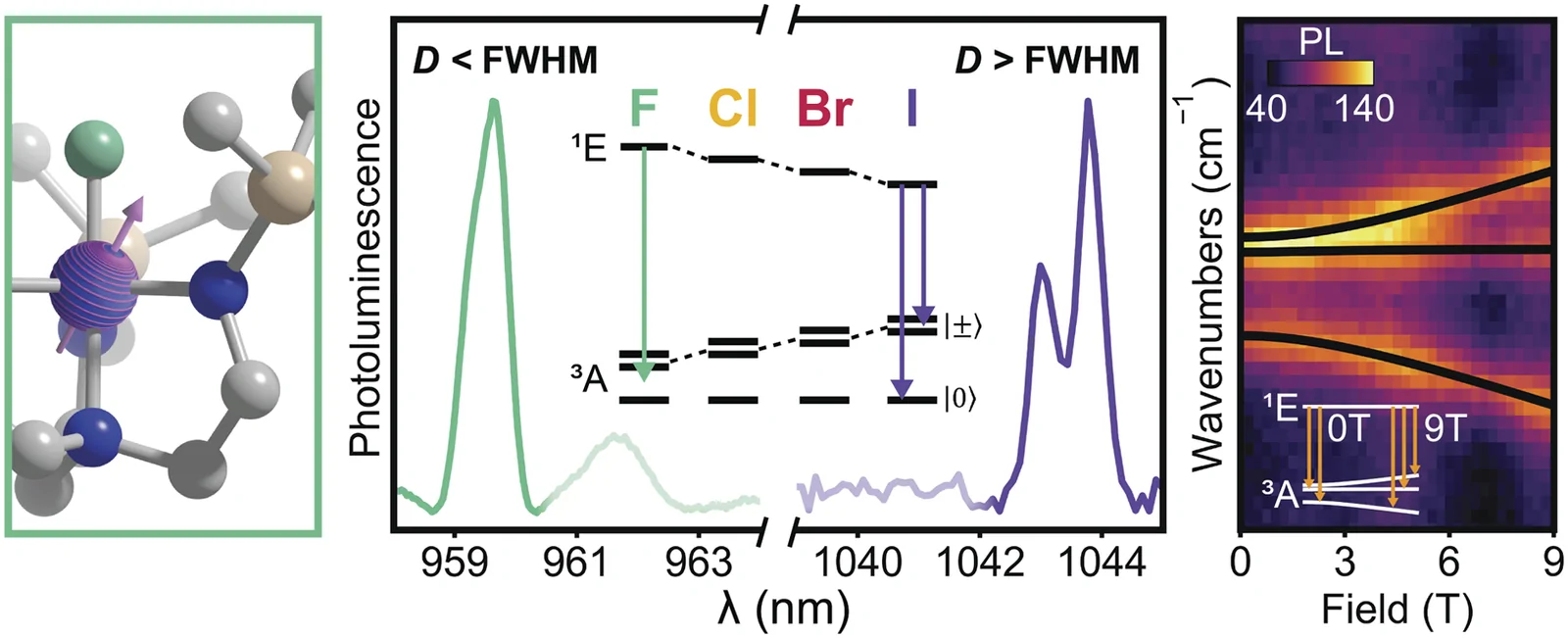

## Introduction

Quantum sensors enable more precise read-out of magnetic and electronic information than their classical counterparts. The most common approach to quantum sensing in chemical, biological, and condensed matter systems is optically detected magnetic resonance (ODMR) spectroscopy.^1,2^ By creating a connection between spin sublevels separated by microwave-scale energy and optical frequency, it is possible to use the small changes at ∼cm^−1^ scale to acquire information and then read out with optical light. The primary quantum bit, or qubit, platform for ODMR-based quantum sensing is electron spin defects in the solid-state, or “color centers.” These color centers are optically addressable, meaning that spins can be optically driven to an initial known state and their state manipulation subsequently interpreted through changes in photoluminescence (PL).^2–4^ A spin-optical interface enables addressability of individual spins, a highly desirable property for magnetic resonance-based sensing down to the single-cell or even molecular limit.^5–7^ Experiments, primarily with the anionic nitrogen vacancy pair defect in diamond (NV^−^), have realized *in vivo* thermometry, electrical activity monitoring in living tissue, and nanoscale imaging of magnetic and electric fields in materials, amongst other findings.^8–18^ These powerful proof-of-concept experiments illustrate the potential of quantum sensing.

While spin defects in the solid-state provide a valuable sensing platform,^19–22^ molecular analogues allow for both the compositional and spatial control necessary to tune electronic structure while enforcing deterministic placement. Molecular electronic spins have emerged as a promising alternative class of qubits, as their bottom-up syntheses offer a customizable approach to sensor design.^23–29^ We can reconstitute optical addressability in a synthetically tunable qubit by incorporating the relevant components of defect electronic structure within a coordination complex: a microwave-addressable *S* = 1 ground state and a low-lying emissive *S* = 0 excited state. The narrow linewidths afforded by spin-flip transitions in transition metal complexes are also required to selectively and resonantly excite from one spin-sublevel for initialization.^30,31^ A molecular platform offers synthetic handles for controlling spin-analyte distance with ångström-scale resolution.^32,33^ We previously reported the first examples of “molecular color centers” in a series of homoleptic *S* = 1 tetrahedral Cr^4+^ complexes featuring strong-field carbon-based donors.^30^ By varying ligand donor strength, we can tune both emission energy and zero-field splitting (ZFS) parameters within these tetrahedral Cr^4+^ complexes.^34^ Excitement over these findings has led to the proposal of numerous candidate molecules to meet a range of possible sensor design requirements.^35–42^

Leveraging the potential of molecules for sensing requires the creation of qubits with tunable spin-analyte interactions of known distance and orientation. Building on our previous work, we sought to develop a series of *S* = 1 Cr^4+^ complexes with a ligand architecture that allows directed chemical attachment to an analyte of choice at an open coordination site on the metal center. We therefore targeted a family of heteroleptic Cr^4+^ complexes varying by an apically bound halide, which is primed for substitution through salt metathesis with ligands containing functional groups capable of selective tethering to a desired analyte, substrate, or other spins.^43^ Here we evaluate four trigonal bipyramidal (TBP) Cr^4+^ complexes featuring the tripodal ligand tris[2-(trimethylsilylamido)ethyl]amine (denoted here as ^TMS^tren), as well as an apical halide (X = F, Cl, Br, and I, respectively known as 1, 2, 3, and 4).^43,44^ Powder samples of 1–4 exhibit an axial ZFS parameter *D* ranging from +5.194(4)–+7.34(1) cm^−1^ as derived from high-field, high-frequency continuous-wave electron paramagnetic resonance (HFHF cw-EPR) spectra. Through combined spectroscopic and computational analyses, we illustrate that magnetic properties are tuned by the donor strength and spin–orbit coupling imparted by the apical halide. These complexes, when diluted in their Ti^4+^ analogues, denoted as 1′–4′, exhibit near-infrared emission ranging from 959.57(1)–1043.81(1) nm by low-temperature photoluminescence (PL) spectroscopy. The inhomogeneous linewidths of the emissive spin-flip transitions and the axial ZFS are of the same order of magnitude; 1′–4′ were then subjected to variable-field PL measurements. These data were then used to optically estimate ZFS parameters *D* and the transverse ZFS parameter *E* in 4′. This new class of molecular color center candidates shows potential use for all-optical magnetic field sensing experiments.

## Results and discussion

1–4 were synthesized from literature procedures (Fig. 1) and their identities were confirmed by X-ray crystallography.^45,46^ Following adapted procedures, the diamagnetic Ti^4+^ analogues were synthesized *via* reaction of 1 eq. Li_3_^TMS^tren with the appropriate Ti^4+^ precursor as specified on page S4.^47,48^ All complexes feature Cr^4+^ in a TBP geometry defined by an apical halide ligand and a ^TMS^tren^3−^ ligand bearing three equatorial amido donors and an axial amino donor. The primary coordination spheres of 1–4 were evaluated by *τ*_5_ and Continuous Shape Measurement (CShM) analyses to quantify their deviation from ideal TBP geometry (Tables S1 and S2).^49,50^ These calculations support the adherence of bond angles to TBP geometry and bond length elongation along the axis of threefold symmetry (more details in SI page S9). Notably, these complexes also show significant displacement of the Cr atom from the equatorial plane towards the halide. The increase in Cr–X bond length by 0.8 Å from 1 to 4 is expected due to both increasing halide diameter and decreasing electronegativity. The more subtle increase in the Cr–N_axial_ bond length in 1 to 4 can be attributed to steric repulsion between the TMS groups and the halide, as well as increasing trans influence of the halide relative to the amine (Cl^−^ < Br^−^ < I^−^).^46,51^ This trend is observed in other metal-halide complexes.^52^ The bond lengthening along the axis of threefold symmetry (X–Cr–N_axial_) suggests weakening orbital overlap between Cr^4+^ and its axial ligands.

**Fig. 1:**
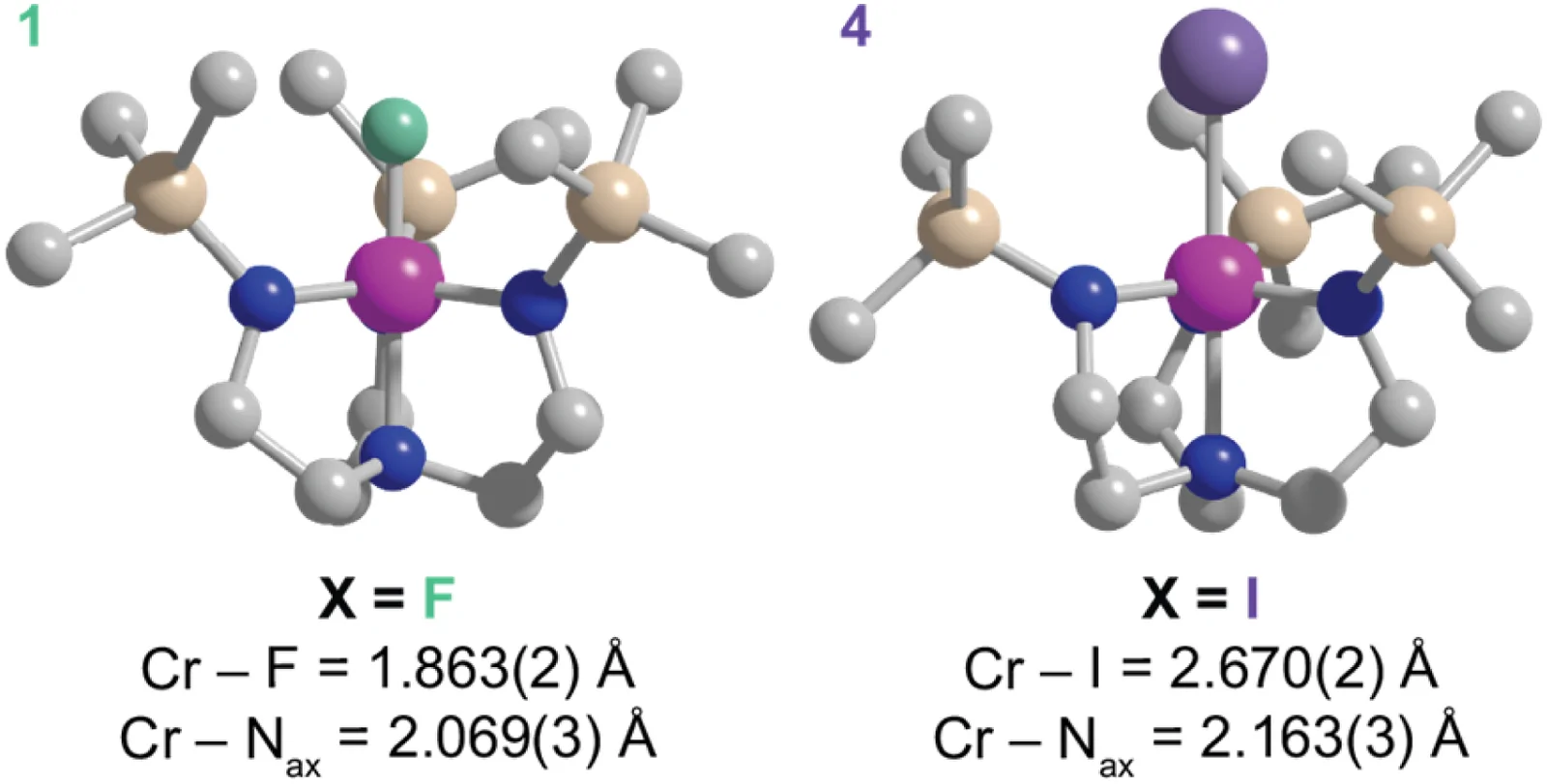
Molecular structures of 1 and 4 determined from X-ray diffraction at 160 K and 100 K respectively.^44^ Carbon, nitrogen, silicon, fluorine, iodine, and chromium atoms are colored as gray, blue, tan, green, purple, and pink, respectively. Hydrogen atoms were omitted for clarity.

To quantify and compare the magnetic anisotropies of each complex, we used cw-EPR spectroscopy. Non-Kramers ions in low-symmetry coordination environments, such as these *S* = 1 TBP complexes, often preclude characterization by conventional X-band (9–10 GHz) cw-EPR spectroscopy due to prohibitively large ZFS. We therefore turned to high-field, high-frequency (HFHF) cw-EPR spectroscopy as the ideal technique for probing such spin systems.^53^ Varying frequency from 63 to 910 GHz, spectra of 1–4 were collected over 0–16 T (Fig. 2, S34, S39, S44, and S49).^53^ The magnetic ground state parameters of 1–4 were obtained from global least-squares fits of variable-frequency HFHF cw-EPR spectra using the spin Hamiltonian 
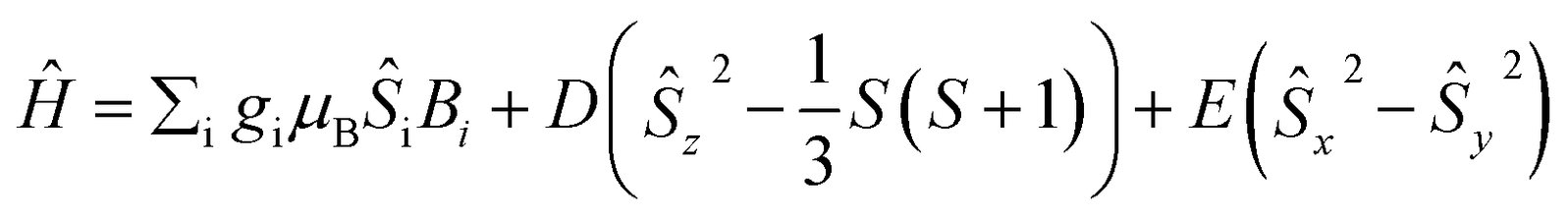
, where *D* and *E* are the respective axial and transverse ZFS parameters, *µ*_B_ is the Bohr magneton, *Ŝ*_*i*_ is the spin operator, *g*_*i*_ is the anisotropic electron g-tensor, and *B*_*i*_ is the magnetic field (*i* = *x*, *y*, *z*) (Table 1). All spectra are dominated by largely axial ZFS, which continually increases as a function of halide size from 1 (*D* = 5.194(4) cm^−1^/155.7(1) GHz) to 4 (*D* = 7.34(1) cm^−1^/220(3) GHz). We confirmed positive values of *D* for 1–4 through analysis of variable-temperature spectra (Fig. S35, S36, S40, S41, S45, S46, S50, and S51). Extracted *g*-values, ranging from 1.956(2) to 1.990(2), are below the free electron *g*-value (∼2.0023) as anticipated for transition metal complexes with less-than-half-filled d shells (d^*n*^, *n* < 5).^54^ Interestingly, for 3, 4 and 4′, *g*_*z*_ is not greater than *g*_*xy*_ (Table 1) as we would expect for a d^2^ spin system with a positive D.^55^ This is indicative of a difference in trend in contributions of triplet states and singlet states across our series; only states of the same multiplicity as the ground state contribute to the *g*-values, whereas the *D*-tensor also contains contributions from Δ*S* = ±1 excited states.^56^ These spin systems are highly analogous to isoelectronic TBP V^3+^ complexes;^57,58^ however, a notable new characteristic of 1–4 is the presence of field-dependent splitting, particularly evident at high fields. We attribute this splitting to weak through-space exchange interactions engendered by short Cr–Cr distances in concentrated samples (Fig. S54 and Table S22), as seen in recent literature on similar systems.^59^ We collected HFHF cw-EPR spectra on samples of 4 diluted in an isostructural Ti^4+^ host. We observe two sets of features indicating the presence of Cr^4+^ in two different environments: (a) a set of broad features with splitting from the aforementioned weak exchange interaction corresponding to a higher concentration of Cr^4+^ and (b) a set of narrow features with no splitting corresponding to isolated Cr^4+^ (Fig. S58). Overall, *D* is tunable by halide identity, with the magnitude increasing with halide size as the result of lower donor strength and higher spin–orbit coupling (SOC) contributions.

**Fig. 2:**
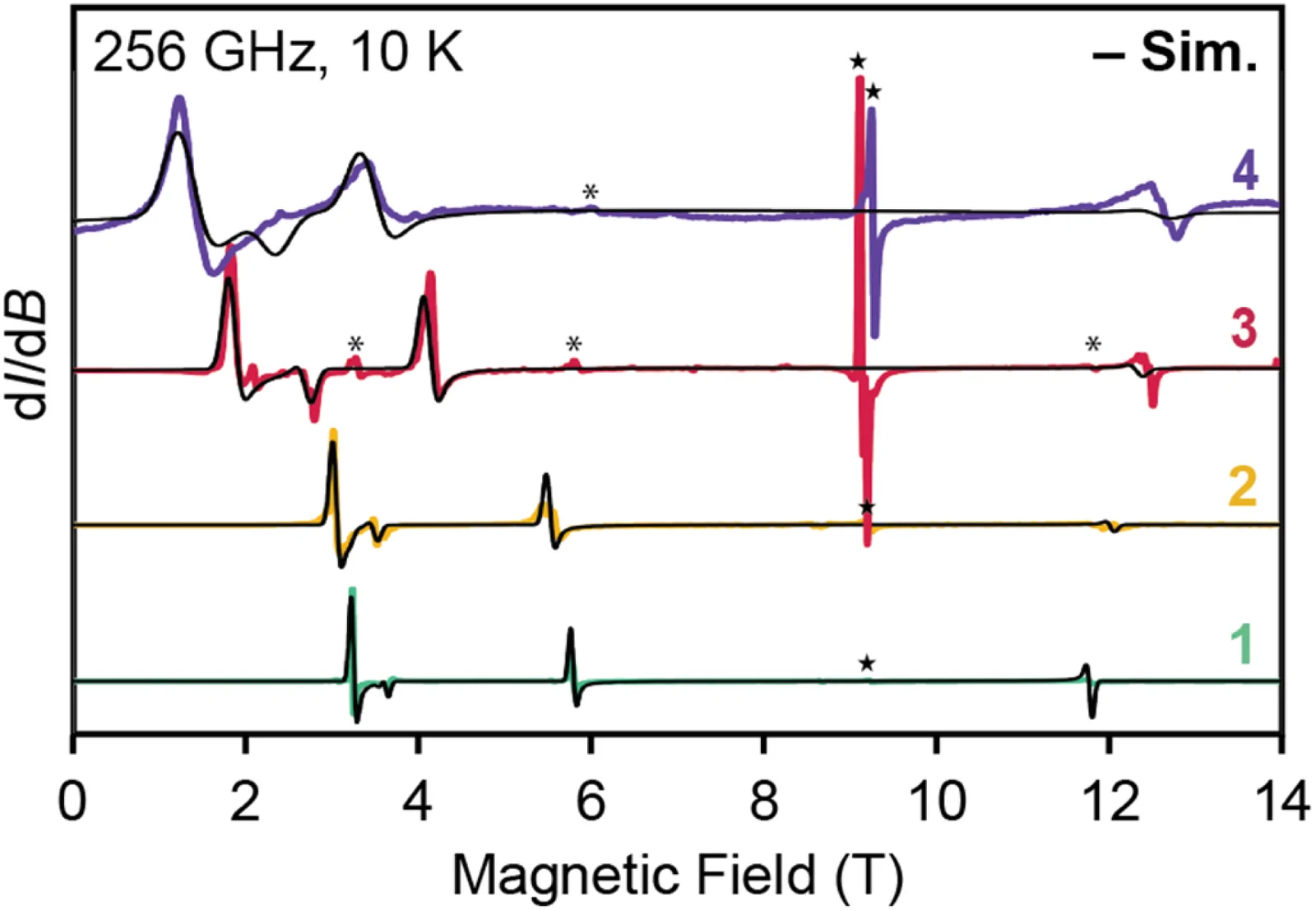
High-field high-frequency cw-EPR spectra of 1–4 taken at 256 GHz and 10 K. The best simulations are shown in black. Stars denote Cr^3+^ impurities and asterisks denote byproduct 2.

**Table 1:** ZFS parameters derived from fitting HFHF cw-EPR spectra in Fig. S34–S53

Complex	*g*_*xy*_	*g*_*z*_	*D* (GHz)	*D* (cm^−1^)
1	1.972(2)	1.990(1)	+155.7(1)	+5.194(4)
2	1.956(2)	1.967(3)	+166(3)	+5.52(1)
3	1.990(2)	1.978(2)	+204(3)	+6.81(1)
4	1.980(2)	1.956(4)	+220(3)	+7.34(1)
4′ (isolated)	2.001(2)	1.997(2)	+215.0(1)	+7.173(3)

Variation in ligand field strength was also evaluated through steady-state absorption spectroscopy. UV-vis absorption spectra of 1–4 in tetrahydrofuran (THF) exhibit broad features that span 350–550 nm with large molar absorptivity coefficients (*ε* > 2000 M^−1^ cm^−1^), characteristic of charge transfer transitions (Fig. 3 and S15–S18).^60,61^ Visualization of natural transition orbitals obtained from time-dependent density functional theory calculations suggests that these are ligand-to-metal charge transfer transitions originating from electron donation from the equatorial amido ligands to chromium (Fig. S24, S25 and Tables S17–S20).^62–64^ These transitions obscure most d–d transitions in the spectra of 1–4; however, both 1 and 4 have features of sufficiently low energy absorptivity to resolve. These features have smaller molar absorptivity coefficients (*ε* < 900 M^−1^ cm^−1^) consistent with assignment as spin-allowed d–d transitions. 2 and 3 feature long tails that could suggest the presence of absorption features too broad or of molar absorptivity too low to resolve from the CT bands. The low energy feature in 4 (∼680 nm) is redshifted from the features resolved in 1, (∼575–610 nm). This result suggests an overall stabilization of d orbitals within the series.

**Fig. 3:**
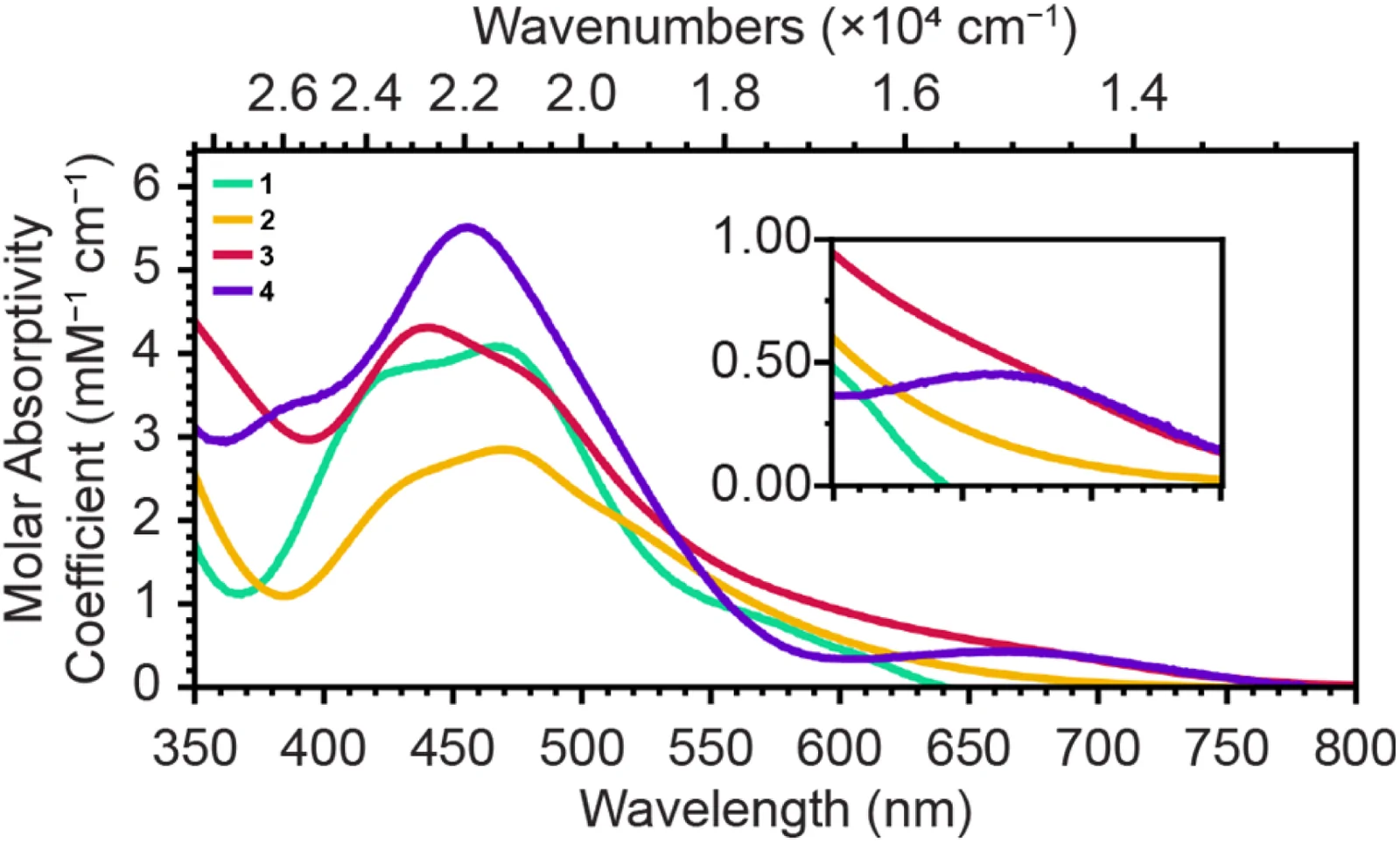
UV-vis spectrum of 1–4 in THF at 298 K. Most metal-centered absorption features are obscured by charge transfer transitions. The inset shows a feature that can be assigned as a d–d transition by it's low molar absorptivity coefficient (*ε* < 900 M^−1^ cm^−1^).

We performed first principles electronic structure calculations in ORCA^65^ to reveal relevant excited triplet and singlet state contributions to ZFS. Starting with the crystal structures of 1–4, we optimized the positions of H atoms using density functional theory (DFT) with the B3LYP functional.^66,67^ Using the def2-TZVP basis set,^68^ we performed complete active space self-consistent field (CASSCF) calculations^69,70^ with second-order *n*-electron valence state perturbation theory (NEVPT2)^71,72^ to account for dynamic correlation. We used spin-unrestricted natural orbitals (UNOs)^73^ from a DFT-PBE^74^ calculation as initial guess orbitals for CASSCF. Although the minimal active space consists of two electrons in five orbitals (2, 5), we employed a larger (4e, 8o) active space where the additional doubly occupied orbital and two unoccupied orbitals have a considerable d-orbital contribution. We state-averaged over 6 triplet and 12 singlet states in the following calculations, which are used in quasi-degenerate perturbation theory to calculate the ZFS parameters.^75,76^ Calculated values of *D* range from 4.23 to 4.51 cm^−1^ (128 to 135 GHz) for complexes 1–3, which are comparable in order of magnitude and increasing trend to our experimentally measured values (Fig. 2 and Table S14).

These trends in electronic structure are produced by the interaction of zeroth-order ground and excited electronic states through SOC. First, we must consider the orbitals that compose the lowest-lying triplet excited states. Increasing halide size corresponds to decreasing σ- and π-donation capabilities, weakening the ligand field along the molecular *z*-axis.^77^ The weaker ligand field lowers the energy of the d_*z*_^2^ orbital towards the singly occupied molecular orbitals (SOMOs), resulting in a larger, more positive contribution to *D* in this *C*_3v_ geometry (see further discussion of ZFS in SI page S9).^78–80^ Supporting this, the calculated five frontier molecular orbitals reveal a large decrease in the relative energy of the orbital of principally d_*z*_^2^ character going from 1–4 and a slight increase in the energy gap between the orbitals of principally {d_*xz*_, d_*yz*_} and {d_*xy*_, d_*x*_^2^_–*y*_^2^} character between 1 and the rest of the molecules in the series (Fig. 4, Tables S15 and S16). This trend in ligand field strength with halide identity is also supported by the decrease in the magnitude of the Cr^4+^/Cr^3+^ reduction potential for 1–4 (Fig. S23). Looking at contributions to ZFS in our CASSCF calculations (Tables S25–24), these triplet excited states are not the only contributors to ZFS in this series. We must also consider the singlet excited states—particularly the singlet state in which the spins pair in the same orbital. This singlet state alone contributes almost half of the *D* in complexes 1–4 (Tables S25–S32). The same dominant singlet contribution to ZFS has been observed in TBP V^3+^ systems.^81^ To spectrographically probe the changes in energy of these spin-flip transitions, we will later turn to PL spectroscopy. Assuming non-zero metal-halide bonding covalency, halides of increasing size also impart a “heavy-atom effect,” contributing increasingly large SOC.^82,83^ The heavy-atom effect further contributes to the positive increase in *D* across the halide series.^84–87^

**Fig. 4:**
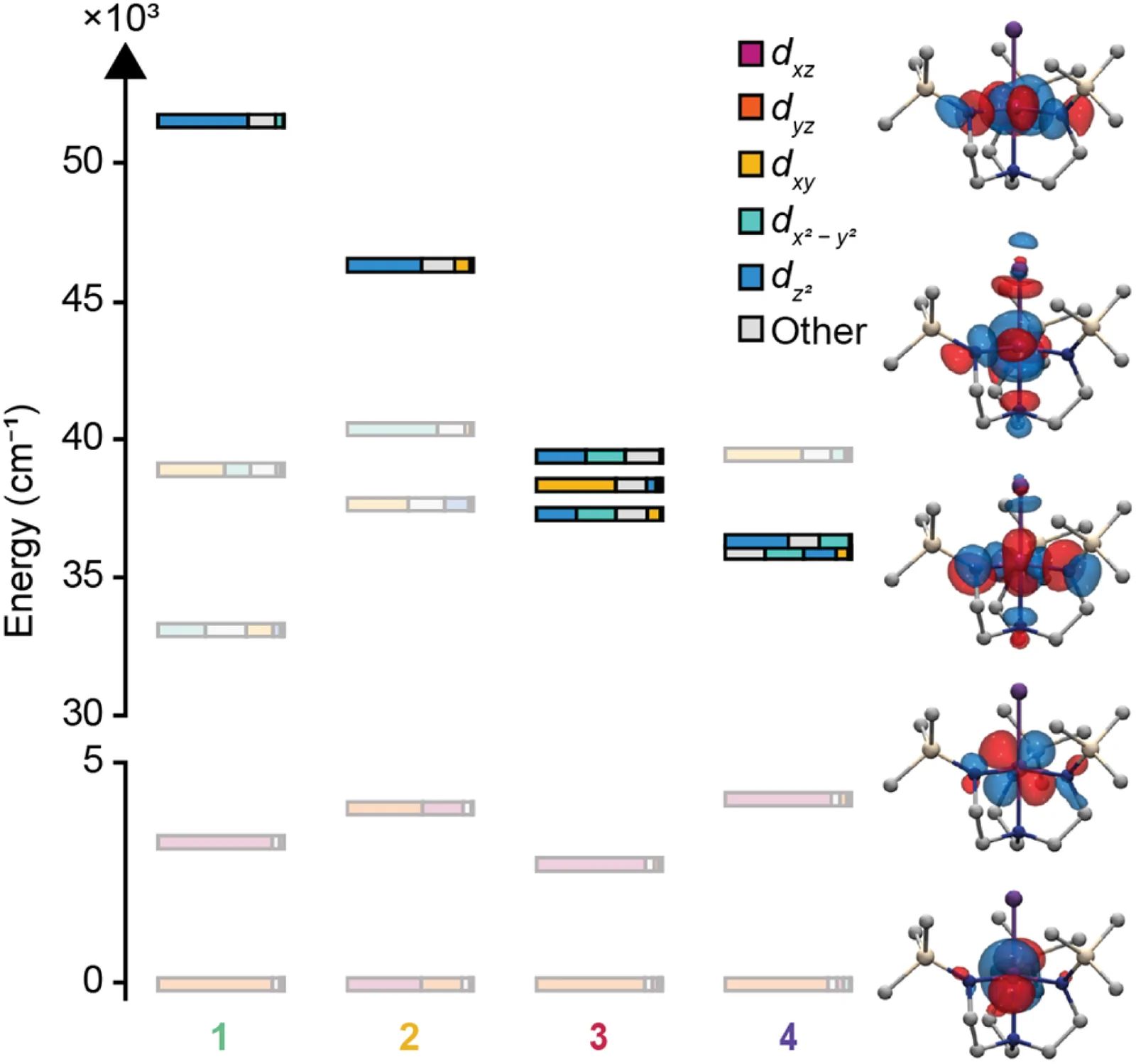
Energies of the five partially occupied natural orbitals from CASSCF (4e, 8o) calculations for 1–4. Orbitals containing significant d_*z*_^2^ character are boldened to highlight the effect of decreasing apical ligand donor strength on the ligand field splitting. Example orbitals of 4 are shown at the right, plotted with an isosurface value of 0.01.

The triplet excited states computed with CASSCF + NEVPT2 (Table S21) also allow us to assign transitions in our absorption spectra. For 1, we attribute the two features centered at 575(2) nm and 611(3) nm to d–d transitions (^3^A_2_ → ^3^A_1_, ^3^A_2_) between the {d_*xz*_, d_*yz*_} and {d_*xy*,_ d_*x*_^2^_–*y*_^2^} orbital sets. For 4, we attribute the feature centered at 685(3) nm instead to d–d transition (^3^A_2_ → ^3^E) between the {d_*xz*_, d_*yz*_} orbital set and the d_*z*_^2^ orbital. 1 therefore partially displays the lowest magnitude of axial ZFS due to its stronger bonding, and illustrates how increasing the donor strength of the ligand at the apical site could further minimize *D*.

To evaluate the impact of the halide on the emissive spin-forbidden transition, which in turn governs *D*, we performed low-temperature PL spectroscopy on 1–4. To optimize these spectra for increased brightness and narrow linewidths, we diluted 1–4 in optically and magnetically transparent d^0^ Ti^4+^ analogues; these diluted samples are denoted as 1′, 2′, 3′, and 4′. Their concentrations were determined by ICP-OES (Table S12). Under off-resonant excitation at 660 nm, 1′–4′ demonstrate near-infrared (NIR) emission with spectral line profiles characteristic of spin-flip transitions: a zero-phonon line (ZPL) with a red-shifted phonon sideband (Fig. 5).^88^ These ZPLs appear from 959.57(1)–1043.81(1) nm, halide size tuning emission energy. As the size of occupied molecular orbitals increases, interelectronic repulsion decreases due to increasing delocalization. This is a phenomenon known as the nephelauxetic effect and an established strategy for tuning the emission energy of spin-flip transitions.^88–90^ The decrease in emission energy observed in these complexes (F^−^ > Cl^−^ > Br^−^ > I^−^) is consistent with trends observed in tetrahedral and octahedral metal-halide complexes.^77^ Electron delocalization onto the apical ligand therefore tunes the singlet energy. The nephelauxetic effect can therefore also be attributed as a significant contributor to the increasing *D*, as lowering the energy of this excited singlet state increases SOC, and subsequently increases magnetic anisotropy across the series.

**Fig. 5:**
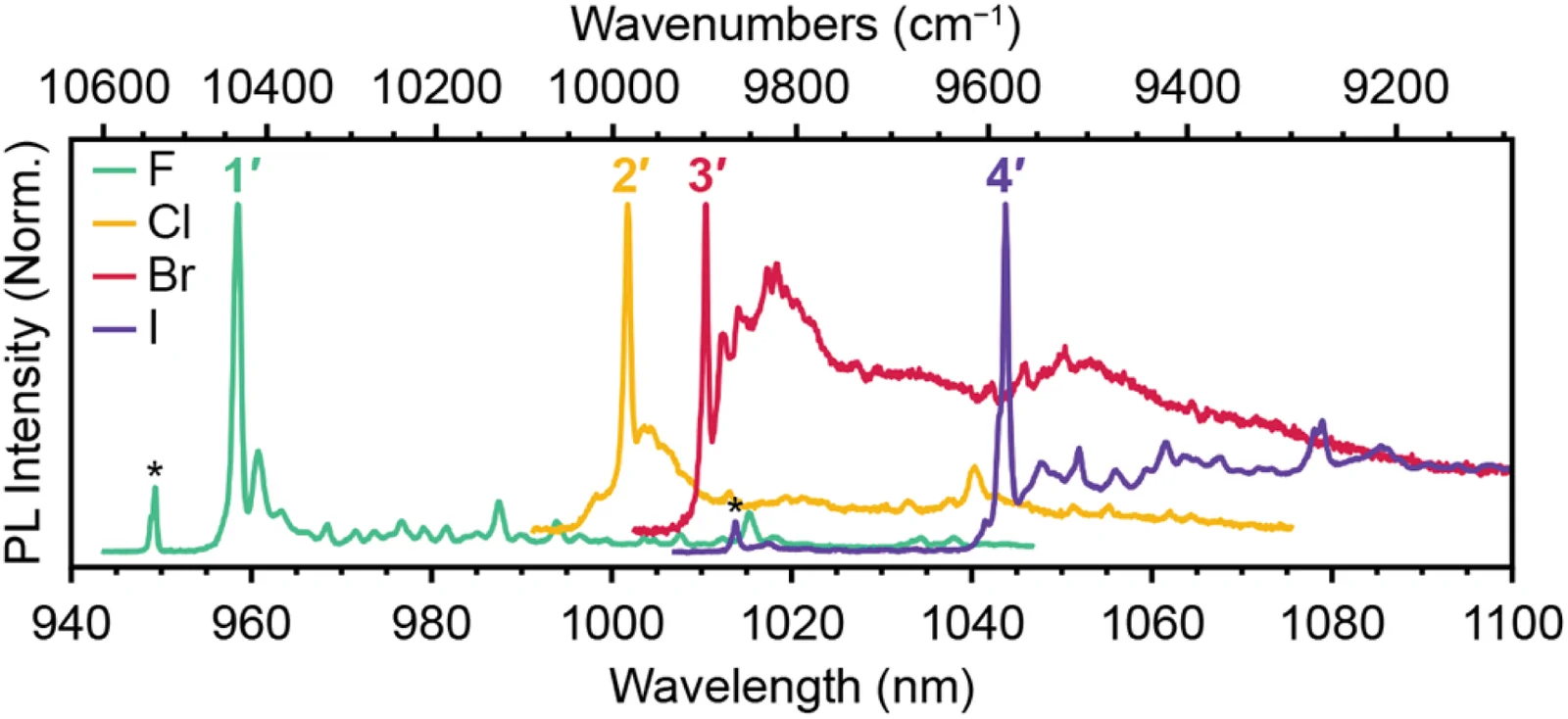
Low-temperature (1.8 or 2.5 K) PL spectra of 1′–4′ under 660 nm excitation (other conditions specified in the SI). The ZPLs labelled (*) are 2 impurities as assigned through single-crystal photoluminescence measurements of this molecule diluted in non-isostructural hosts (Fig. S82–84).

Complexes 1–4 possess narrow linewidths at low temperatures, with their full width at half maximum (FWHM) ranging from 3.22(9)–7.9(4) cm^−1^ because of the intra-configurational nature of spin-flip emission (Fig. 5). Confining the metal-centered electronic transition within the nominally nonbonding {d_*xz*_, d_*yz*_} orbital set generates nested excited singlet states that require minimal geometric distortion to relax.^88^ We observe a decrease in signal intensity and broader linewidths for all ZPLs as temperature is increased from 1.8 K to 300 K due to the increased rate of nonradiative decay resulting from vibronic coupling and increased thermal motion of emitting molecules (Fig. S61, S66, S71, and S76). Emission persists until 300 K for 1′, showing higher stability than our previously reported pseudo-tetrahedral Cr^4+^ systems.

The large *D*-to-optical linewidth ratio in these systems presents an opportunity for all-optical magnetic sensing experiments. From the PL spectrum of 4′, where emission from relaxation to the ground state spin sublevels is resolvable at zero applied field, *D* can be estimated to be 7.2(1) cm^−1^ based on peak-to-peak distance (Fig. S74). Though the optical resolution of these features at zero field is promising for sensing experiments with limited or no access to an applied magnetic field, the margin of error at zero field is still limited by the linewidths. For 4′, we find that *D* obtained by fitting the PL data at zero field is smaller than 4 measured by HFHF cw-EPR spectroscopy. We expect some deviation in magnetic parameters extracted from neat and dilute samples, but by increasing the data-to-parameter ratio, we can obtain a more accurate estimate of ZFS parameters from PL spectra.

As a proof-of-concept of how optical determination of ZFS could be performed in similar systems for calibration in sensing experiments, we subjected a single crystal of 4′ to variable-magnetic-field PL (VF PL) spectroscopy. To fit these data, an experimental Zeeman diagram is constructed by collecting PL spectra from 0–9 T in 0.25 T intervals and plotting peak center as a function of field (more discussion on page S9). These three peaks correspond to relaxation to the three spin sublevels in the *S* = 1 system (Fig. 6a and b). The resultant field-dependent energy diagram is then fit using a simplified spin Hamiltonian. We assume *g*_iso_ = 2 for simplicity and *φ*, the angle defining a rotation of the molecular frame in the *xy* plane, is restricted to 0 due to the axial nature of ZFS in this system. From this fit we obtain *D*, *E*/*D*, and *θ*, a free variable defined as the angle between the applied magnetic field and the principal axis of the ZFS tensor (Fig. S75 and S76). The Zeeman diagrams generated from the parameters obtained overlay well on heat maps of magnetic field (*x*) *vs.* energy (*y*) *vs.* PL intensity (*z*) for 4′ (Fig. S81 and 6c). As a comparison, we generated a Zeeman diagram from the parameters extracted from HFHF cw-EPR spectra and overlayed it onto the same VF PL heatmap (Fig. S78). Given that the EPR data were taken on a powder sample and not a single crystal, we chose *θ* = 75(5)° based on the closest match to experimental PL data.

**Fig. 6:**
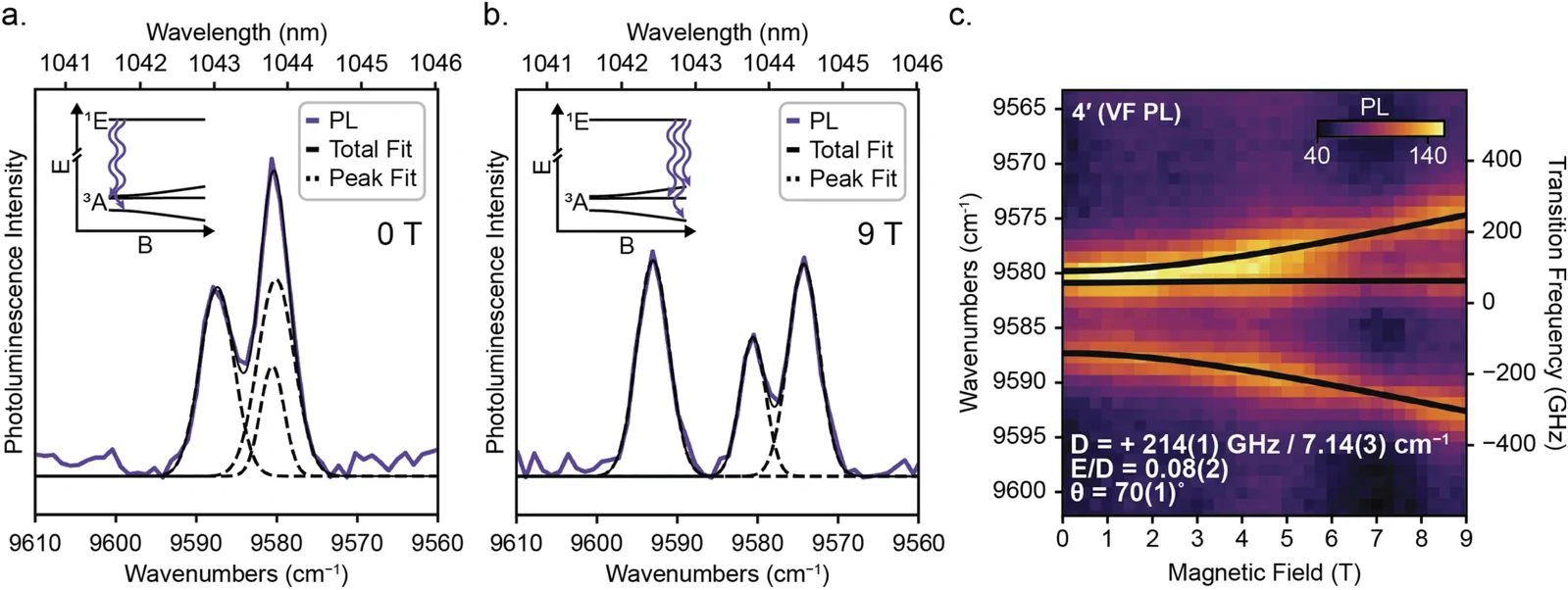
High resolution PL spectra of 4′ single crystal at 0 T (a) and 9 T (b). The raw data are plotted in purple, and Lorentzian fits are shown in black solid (total) and dashed (deconvoluted) black lines. The schematics at the top left illustrate how the variable splitting of the ZPL originates from the splitting of the M_S_ levels. (c) Heatmap of 4′ of the variable-field PL overlayed with the Zeeman diagram generated from VF PL fitting.

Using variable field spectra, we can optically determine ZFS parameters with increased accuracy, with *D* estimated as 7.14(3) cm^−1^ (214(1) GHz). The ZFS parameters determined by VF PL (Fig. 6c) and HFHF cw-EPR are on the same order of magnitude. *D* is smaller in the measurements done on dilute sample 4′; we attribute this difference to a change in crystal packing from the space group *P*2_1_/*c* of the Cr^4+^ sample used for HFHF cw-EPR measurements to *Pbca* upon dilution in a diamagnetic Ti^4+^ host for VF PL measurements. To confirm this, we fit the narrow features in the HFHF cw-EPR spectra collected on dilute samples of 4′; we obtain *D* = 7.173(3) cm^−1^ and *E* = 0.08(2) cm^−1^, which is in excellent agreement with the values obtained from VF PL spectra (Table 1, Fig. S55 and S56). We fit these data using a simplified spin Hamiltonian to avoid overparameterization, but further development of this technique could utilize variable orientation measurements to model *g* anisotropy. VF PL spectroscopy can therefore be a powerful technique for optical determination of ZFS parameters in optically active high-spin materials with competitively large *D*-to-optical linewidth ratios.

## Conclusions

In this work, we demonstrated tunability across a series of heteroleptic Cr^4+^ complexes through shifts in both magnetic ground state ZFS parameters and excited singlet state energies through the identity of the apical ligand. Additionally, we demonstrated optical determination of ZFS parameters at zero applied magnetic field. Molecular color centers with *D*-to-optical linewidths that allow for the optical resolution of spin sublevels at low fields, such as those displayed in this series, hold potential in enhancing spin selectivity of optical pumping. These complexes can also serve as valuable precursors to more complex systems, enabling the integration of molecular color centers into materials and multi-spin systems. Prior work demonstrates that substitution of the apical halide provides routes for covalent attachment of electron spin to analytes, substrates, or other spins for sensing experiments. Future work will focus on further tuning of the apical ligand by making use of strong aryl and alkyl donors to induce a smaller *D* for addressability by conventional microwave frequencies. Measurements of spin dynamics *via* pulsed EPR experiments and optical lifetimes, and the introduction of pertinent functional groups for mild coupling reactions, are key next steps for developing the second generation of optically addressable heteroleptic Cr^4+^ molecular color center candidates.

## Author contributions

C. S. N. M. and R. B. G. contributed to this work equally and led data acquisition, synthesis, and analysis. As this work spans seven years, author contributions will be listed chronologically. M. S. F. and K. A. C. initiated data acquisition and analysis on this project. M. K. W. and D. W. L. continued data acquisition and analysis. C. S. N. M., R. B. G., C. W., and N. M. acquired and analyzed spectroscopic data. A. K. and T. C. B. contributed theoretical analysis. A. O. acquired and contributed to the analysis of magnetic resonance data. D. D. A., T. C. B., and D. E. F. supervised the project and contributed to data analysis. All authors contributed to the manuscript.

## Conflicts of interest

There are no conflicts to declare.

## Supplementary Material

SC-017-D6SC05602D-s001

SC-017-D6SC05602D-s002

## Data Availability

The datasets supporting this article have been included as part of the supplementary information (SI). Supplementary information: crystallographic, EPR, optical, electrochemistry, and computational data for **1–4**. See DOI: https://doi.org/10.1039/d6sc05602d. CCDC 2566674, 2566679 and 2566680 contain the supplementary crystallographic data for this paper.^91*a*–*c*^
