# Supplementary material for: Heteroleptic Cr4+ molecular color center candidates
Source: Chem Sci. 2026 Sep 18;17(42):20344–53. doi: 10.1039/d6sc05602d (PMC13587845; doi:10.1039/d6sc05602d)
Supplement: Supplementary file 1 [file SC-017-D6SC05602D-s001.pdf]

Supplementary Information (SI) for Chemical Science.
This journal is © The Royal Society of Chemistry 2026

Supporting Information for:

Heteroleptic Cr^{4+} Molecular Color Center Candidates

Cindy Serena Ngompe Massado[1,†], Rianna B. Greer[1, †], Arailym Kairalapova[2], Andrew Ozarowski[3], Kelsey A. Collins[4], Majed S. Fataftah[4], Noah Mendelson[5,6], Chloe A. Washabaugh[5,6], Daniel W. Laorenza[4], Michael K. Wojnar[1], David D. Awschalom[5,6,*], Timothy C. Berkelbach[2,7,*], and Danna E. Freedman[1,*]

[1]Department of Chemistry, Massachusetts Institute of Technology, Cambridge, Massachusetts 02139, USA

[2]Department of Chemistry, Columbia University, New York, New York 10027, United States

[3]National High Magnetic Field Laboratory, Tallahassee, Florida 32310, United States

[4]Department of Chemistry, Northwestern University, Evanston, Illinois 60208, USA

[5]Pritzker School of Molecular Engineering, University of Chicago, Chicago, Illinois 60637, USA

[6]Department of Physics, University of Chicago, Chicago, Illinois 60637, USA

[7]Initiative for Computational Catalysis, Flatiron Institute, New York, New York, 10010, USA

† Cindy Serena Ngompe Massado and Rianna B. Greer contributed equally to this work.

Full Experimental Details S5

Supplementary text S9

Table S1 | Calculated τ5 values for **1–4**. S12

Table S2 | Calculated CShM values for **1–4**. S12

Table S3 | AOM Parameters for Cr^{3+} in Octahedral Geometry. S12

Table S4 | Comparison of structural parameters in other ^{TMS}trenCrR systems. S12

Tables S5–S7 | Summary of crystallographic data for **4**, **5**, and **7** at 100 K. S13

Tables S8–S11 | Features identified by second derivative UV-vis spectra of **1–4**. S16

Table S12 | Cr^{4+}:Ti^{4+} ratios determined from ICP-OES. S17

Tables S13–S20 | Contributions of molecular orbitals composing the active space used in CASSCF (4e, 8o) calculations for **1–4**. S18

Table S21 | Ground state to triplet excited state transition energies from CASSCF (4e, 8o) with NEVPT2. S26

Table S22 | ZFS and g-factor data determined by CASSCF (4e, 8o) with NEVPT2. S26

Table S23 | Orbital energies (eV) from CASSCF (4e, 8o) with NEVPT2. S26

Table S24 | Orbital energies (cm^{-1}) from CASSCF (4e, 8o) with NEVPT2. S26

Table S25–S34 | Triplet and singlet-state ZFS contributions and configurations as determined by CASSCF (4e, 8o) with NEVPT2 for **1–4**. S27

Tables S33–S36 | TD-DFT natural transition orbitals (NTO) for **1–4**. S35

Table S37 | Spin Hamiltonian parameters derived from HFHF cw-EPR spectra. S41

Table S38 | Exchange coupling parameters extracted for complexes **1–4**. S41

Figures S1–S9 | NMR spectra of **5–8**. S42

Figures S10–S11 | Peak positions of ^{1}H NMR spectra of complexes **1–4** and **5–8**. S51

Figures S12–S14 | Thermal ellipsoid plots of **4**, **5**, and **7**. S53

Figures S15–S18 | UV-vis spectra of **1–4**. S56

Figures S19–S22 | FT-IR spectra of **4–8**. S19

Figure S23 | Narrow-scan cyclic voltammograms (CVs) for **1–4**. S64

Figures S24–S25 | Absorption spectra of **1–4** generated by TD-DFT**.** S65

Figures S26–S33 | DC magnetic susceptibility and variable-field, variable-temperature magnetization data for **1–4**. S67

Figure S34 | Variable-frequency HFHF cw-EPR spectra for **1** at 10 K. S75

Figure S35 | Variable-temperature HFHF cw-EPR spectra for **1.** S76

Figure S36 | Simulated EPR spectra for **1** using a negative value of *D*. S77

Figure S37 | Zeeman diagrams along the x, y, and z axes for **1**. S78

Figure S38 | Turning points extracted from variable-frequency EPR spectra of **1** at 10 K overlaying transitions generated from fits. S79

Figure S39 | Variable-frequency HFHF cw-EPR spectra for **2** at 10 K. S80

Figure S40 | Variable-temperature HFHF cw-EPR spectra for **2**. **S81**

Figure S41 | Simulated EPR spectra for **2** using a negative value of *D*. **S82**

Figure S42 | Zeeman diagrams along the x, y, and z axes for **2**. **S83**

Figure S43 | Turning points extracted from variable-frequency EPR spectra of **2** at 10 K overlaying transitions generated from fits. **S84**

Figure S44 | Variable-frequency HFHF cw-EPR spectra for **3** at 10 K. **S85**

Figure S45 | Variable-temperature HFHF cw-EPR spectra for **3**. **S86**

Figure S46 | Simulated EPR spectra for **3** using a negative value of *D*. **S87**

Figure S47 | Zeeman diagrams along the x, y, and z axes for **3**. **S88**

Figure S48 | Turning points extracted from variable-frequency EPR spectra of **3** at 10 K overlaying transitions generated from fits. **S89**

Figure S49 | Variable-frequency HFHF cw-EPR spectra for **4** at 10 K. **S90**

Figure S50 | Variable-temperature HFHF cw-EPR spectra for **4**. **S91**

Figure S51 | Simulated EPR spectra for **4** using a negative value of *D*. **S92**

Figure S52 | Zeeman diagrams along the x, y, and z axes for **4**. **S93**

Figure S53 | Turning points extracted from variable-frequency EPR spectra of **4** at 10 K overlaying transitions generated from fits. **S94**

Figure S54 | Exchange coupling modeled in the forbidden ($\Delta m_s = 2$) half-field transitions of complexes **1**–**4**. **S95**

Figure S55 | Variable-frequency HFHF cw-EPR spectra for **4’** at 10 K. **S96**

Figure S56 | Turning points extracted from variable-frequency EPR spectra for the isolated species in **4’** at 10 K overlaying transitions generated from fits. **S97**

Figure S57 | Turning points extracted from variable-frequency EPR spectra for the concentrated species in **4’** at 10 K overlaying transitions generated from fits. **S98**

Figure S58 | Spectra of the forbidden ($\Delta m_s = 2$) half-field transitions of complexes **4** and **4’**. **S99**

Figure S59 | PL spectrum of **1′** at 2.5 K. **S100**

Figure S60–S61 | Variable-temperature PL collected on **1′**. **S101**

Figure S62–S63 | Variable-field PL collected on **1′**. **S103**

Figure S64 | PL spectrum of **2′** at 2.5 K. **S105**

Figure S65–S66 | Variable-temperature PL collected on **2′**. **S106**

Figure S67–S68 | Variable-field PL collected on **2′**. **S108**

Figure S69 | PL spectrum of **3′** at 2.5 K. **S110**

Figure S70–S71 | Variable-temperature PL collected on **3′**. **S111**

Figure S72–S73 | Variable-field PL collected on **3′**. **S113**

Figure S74 | PL spectrum of **4′** at 2.5 K. **S115**

Figure S75–S76 | Variable-temperature PL collected on **4′**. **S116**

Figure S77–S78 | Variable-field PL collected on **4′**. **S118**

Figure S79 | Peak centers obtained from freely fitting VFPL collected on **4′**. **S120**

Figure S80 | Zeeman fit of peak centers obtained from fitting VFPL collected on **4′**. **S121**

Figure S81 | Variable-field PL collected on **4′** overlayed with Zeeman diagram modeled with parameters obtained from fitting peak centers. **S122**

Figure S82–S84 | PL collected on **1′** overlayed with **2** diluted in **5**, **7**, and **8**. **S123**

References **S126**

Full Experimental Details

General considerations and synthesis.

All complexes were synthesized and handled under a N_2 atmosphere in a Vacuum Atmosphere Nexus II glovebox. Glassware was oven-dried at 150°C for at least six hours prior to use. Pentane (pent), diethyl ether (Et_2O), hexanes (hex), tetrahydrofuran (THF), dichloromethane (DCM), and toluene (tol) were degassed by argon sparging and dried using a commercial solvent purification system (Pure Process Technology) and stored over 4 Å sieves for a minimum of one day or purchased from Sigma Aldrich and used as is. Prior to use, the following chemicals were deoxygenated and dried as specified. deuterated benzene (C_6D_6, Cambridge Isotopes) was dried NaH and deoxygenated by three successive freeze-pump-thaw cycles. Celite® 545 (Celite, Sigma Aldrich) was dried at 250°C under vacuum for two days. Reagents such as *n*-butyllithium (*n*-BuLi, 2.5 M in hex, Sigma Aldrich), tris(2-aminoethyl)amine, chlorotrimethylsilane (TMSCl), titanium tetrafluoride (TiF_4), titanium tetrabromide ($TiBr_4$), and titanium tetraiodide (TiI_4) were used as received. $CrCl_3{\cdot}3THF$,[1] $TiCl_4{\cdot}2THF$,[2] $TiF_4{\cdot}2THF$,[3] $Li_3{}^{TMS}$tren,[4,5] ^{TMS}trenCrX (X = F, Cl, Br, I),[6,7] ^{TMS}trenTiCl[8] were prepared according to previously published literature procedures, and the identity of the products was confirmed by X-ray crystallography and other physical characterization methods.

^{TMS}trenTiF (5): A solution of $TiF_4{\cdot}2THF$ (1 eq, 192 mg, 0.717 mmol) in 5 mL of pentane was added to a 20 mL scintillation vial charged with a magnetic stir bar, 300 mg of $Li_3{}^{TMS}$tren (1.1 eq., 0.788 mmol), and 5 mL of pentane. The mixture was then left to stir overnight, after which it was filtered, and the solvent was removed under vacuum. The yellow solid was dissolved in minimal pentane and stored at −22 °C, recrystallizing 99 mg (32% yield). ^{1}H NMR (400 MHz, C_6D_6): 3.28 ppm (t, 6H, J = 5.5 Hz), 2.46 ppm (t, 6H, J = 5.5 Hz), 0.34 ppm (s, 27H). ^{13}C NMR (126 MHz, C6D6): 58.57 ppm, 50.11 ppm, 1.49 ppm. ^{19}F NMR (471 MHz, CDCl3): 139.48 ppm. MALDI ToF MS: m/z (calc) = 426.1942 $[M]^-$

^{TMS}trenTiCl (6): ^{1}H NMR (400 MHz, C_6D_6): 3.38 ppm (t, J = 5.0 Hz, 6H), 2.29 ppm (d, J = 5.1 Hz, 6H), 0.36 (s, 27H). ^{13}C NMR (101 MHz, C_6D_6): 63.19 ppm, 50.84 ppm, 1.66 ppm.

^{TMS}trenTiBr (7): A solution of $TiBr_4$ (1 eq, 263 mg, 0.717 mmol) in 5 mL of pentane was added to a 20 mL scintillation vial charged with a magnetic stir bar, 300 mg $Li_3{}^{TMS}$tren (1.1 eq., 0.788 mmol), and 5 mL of pentane. The mixture was then left to stir overnight, after which it was filtered, and the solvent was removed under vacuum. The orange solid was dissolved in minimal pentane and stored at −22 C, recrystallizing 105 mg (30% yield). ^{1}H NMR (400 MHz, C_6D_6): 3.40 ppm (t, 6H, J = 5.2 Hz), 2.23 ppm (t, 6H, J = 5.2 Hz), 0.39 ppm (s, 27H). ^{13}C NMR (126 MHz, C6D6): 63.69 ppm, 50.92 ppm, 1.79 ppm. MALDI ToF MS: m/z (calc) = 486.1139 $[M]^-$

^{TMS}trenTiI (8): A solution of TiI_4 (1 eq, 239 mg, 0.717 mmol) in 5 mL of pentane was added to a 20 mL scintillation vial charged with a magnetic stir bar, 300 mg $Li_3{}^{TMS}$tren (1.1 eq., 0.788 mmol), and 5 mL of pentane. The mixture was then left to stir overnight, after which it was filtered, and the solvent was removed under vacuum. The red solid was dissolved in minimal pentane and stored at −22 ˚C, recrystallizing 142 mg (37% yield). ^{1}H NMR (500 MHz, C_6D_6): 3.43 ppm (t, 6H, J = 5.0 Hz), 2.13 ppm

(t, 6H, J = 5.2 Hz), 0.43 ppm (s, 27H). ^{13}C NMR (126 MHz, C6D6): 64,24 ppm, 50.83 ppm, 2.18 ppm. MALDI ToF MS: m/z (calc) = 426.1942 $[M]^-$

X-ray diffraction.

Single crystal X-ray diffraction data were collected using various instruments. Data for ^{TMS}trenTiF and ^{TMS}trenTiBr were collected using a STOE STADIVARI diffractometer using Axo microfocus Ag Kα and Cu Kα sources, respectively, an EIGER2 1M CDTE detector, and an Oxford Cryostream cooler. Raw data were integrated using X-Area,[9] and absorption corrections were then applied by Gaussian integration in STOE X-Red32.[10] Scaling of reflection intensities was performed with STOE LANA.[11] Data for ^{TMS}trenCrI were collected using a Bruker D8 Venture Kappa DUO four-circle diffractometer using an IμS micro-focus sealed X-ray tube (Mo Kα) and an Incoatec IμS multilayer mirror monochromator. Data was integrated with the Bruker SAINT software package,[12] and the Multi-Scan method (SADABS)[13] was used for absorption correction. The space groups of each compound were determined by examination of systematic absences, E-statistics, and successive refinement of the structure. Using the OLEX2 interface,[14] the structures were resolved with intrinsic phasing (SHELXT) methods,[15] and refined using least squares minimization (SHELXL).[16] Non-hydrogen atoms were refined anisotropically. All hydrogen atoms were fixed at ideal positions and refined using a riding model.

High-Field, High-Frequency Continuous-Wave Electron Paramagnetic Resonance (HFHF cw-EPR) measurements.

All high-field EPR measurements were performed at the National High Magnetic Field Laboratory (NHMFL) in Tallahassee, Florida, on a 24–660 GHz EPR spectrometer with a superconducting 15/17 T magnet.[17] Samples were prepared in a nitrogen glovebox; the Cr^{4+} complexes were recrystallized out of pentane, the crystals ground up with a mortar and pestle, and powdered samples were used to prepare EPR samples in polyethylene vials secured with Teflon rods. All HFHF cw-EPR spectroscopy data were processed through a combination of MATLAB,[18] EasySpin,[19] EPRtoolbox,[20] EPR Simulation Program,[21] and Python. The magnetic field values of major features were originally fit using the following Hamiltonian:

$$\hat{H} = \Sigma g_i \mu_B \hat{S}_i B_i + D\left(\hat{S}_z^2 - \frac{1}{3}S(S+1)\right) + E\left(\hat{S}_x^2 - \hat{S}_y^2\right)$$

D and E are, respectively, the axial and transverse zero-field splitting (ZFS) parameters, $\hat{S}_i$ is the spin operator, μ_B is the Bohr magneton, g_i (i = x, y, z) is the anisotropic electron g-value, and B_i, the magnetic field. The best fits used axial magnetic ground states where $E = 0$ and $g_x = g_y$. Absolute intensities of the cw-EPR spectra were phase-corrected and normalized between 0 and 1 prior to simulation of the spectra in EasySpin using the pepper function for frozen solids. The sign of D was obtained from variable-temperature HFHF cw-EPR spectra at one frequency for each complex (see Figures S35, S36, S40, S41, S44, S45, S49, and S50).

Some features demonstrated field-dependent splitting at high enough fields. To model this effect, the half-field ($\Delta m_s = 2$) transitions at their highest field were fit using the following Hamiltonian:

$$\hat{H} = \sum_{ij} \left\{ J_{ij}(\hat{S}_i \cdot \hat{S}_j) + D_{ij}\left(\hat{S}_{zi}\hat{S}_{zj} - \frac{\hat{S}_i \cdot \hat{S}_j}{3}\right) + E_{ij}(\hat{S}_{xi}\hat{S}_{xj} - \hat{S}_{yi}\hat{S}_{yj}) \right\} + \left\{ \Sigma g_i \mu_B \hat{S}_i B_i + D_i\left(\hat{S}_{zi}^2 - \frac{1}{3}S_i(S_i+1)\right) + E_i(\hat{S}_{xi}^2 - \hat{S}_{yi}^2) \right\}$$

J_{ij} is the exchange integral between adjacent Cr^{4+} ions i and j. The fits for parameters from the original Hamiltonian were used to generate another fit with the latest Hamiltonian, resulting in the spectra simulated in Figure S54.

High-field photoluminescence measurements.

Photoluminescence measurements were performed on samples diluted in their respective ^{TMS}trenTiX analogues and recrystallized in hexanes at −22 °C. High-field measurements from 0 to 9 T were performed in a cryostat with an integrated superconducting magnet (Quantum Design Physical Property Measurement System, PPMS). The samples were cooled to 1.8 or 2.5 K. To provide optical access to the sample, we use an optical fiber (Thorlabs, FP400ERT) mounted in a custom-built probe. The sample end of the probe mounts to the base of the PPMS through a sample-mount puck. The end of the optical fiber at the sample is mounted directly on the sample to provide excitation and PL collection, and is clamped in place. A custom-made vacuum-compatible fiber feedthrough connects this fiber to excitation and collection optics outside of the cryostat, which consist of a free-space optical breadboard setup. We illuminate samples using an off-resonant 660 nm diode laser (Thorlabs, S1FC660), which is spectrally filtered, passes through a dichroic mirror (Thorlabs, DMSP1000R), and is then coupled into the optical fiber connected to the sample. The PL from the sample is collected using the same fiber, spectrally separated from the excitation light using the dichroic mirror and a longpass filter (Thorlabs, FELH1000), and sent to a spectrometer (Princeton Instruments, SP2500) equipped with a liquid nitrogen cooled InGaAs CCD (Princeton Instruments, OMA V:1024-2.2). For the optical Zeeman experiments, we used a 1200 grooves/mm grating. PL spectra as a function of field are measured by ramping the magnetic field from 0 to 9 T, while simultaneously acquiring spectra. The applied magnetic field is parallel to the excitation beam path. Spectra are then averaged over a window of ±100 mT. To obtain the differential PL spectra, we take [PL(λ, B0) − PL(λ, 0)]/PL(λ0, 0), where PL(λ0, 0) is the peak maximum in the 0 T spectrum.

Electronic structure calculations.

All calculations were performed using Orca v6.0 with the def2-TZVP basis set, resolution of the identity, and chain of sphere exchange (RIJCOSX) approximations[22,23]; test calculations with the larger def2-QZVPP basis set did not change our results significantly. The initial geometries were obtained from experimental crystal structures, and DFT calculations with the B3LYP functional were performed to optimize the positions of hydrogen atoms while fixing heavier atom positions. To facilitate analysis in terms of textbook 3d orbitals, a series of translations and rotations were

performed such that the Cr metal is located at the origin, the Cr-X bond is along the z-axis, and one of the equatorial Cr-N bonds was placed in the xz-plane.

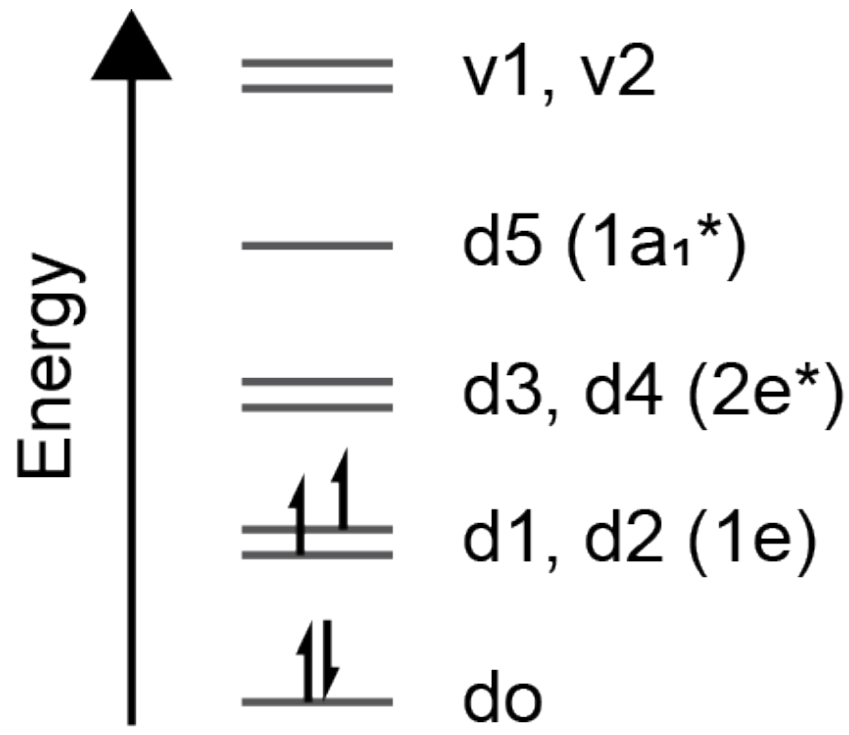

CASSCF calculations were performed with an active space of 4 electrons in 8 orbitals (pictured above, tables S13–S20), performing state-averaging over 6 triplet and 12 singlet states (this number of states was identified as sufficient for converging the ZFS parameters). The initial guess orbitals were spin-unrestricted natural orbitals (UNOs) obtained from a PBE calculation, from which we included the five UNOs with partial occupation numbers and Cr 3d character (roughly two singly occupied orbitals and three unoccupied orbitals), as well as one energetically nearby doubly occupied orbital and two higher energy unoccupied orbitals with significant Cr 3d character. The five most important natural orbitals are plotted in Fig. 2 using the VMD software,[24] and orbital energies are estimated as diagonal elements of the CASSCF Fock operator (plotted in Fig. 2 and given in Table S15 (in eV) and Table S16 (in cm^{-1})). To account for dynamical correlation, CASSCF calculations were followed by strongly-contracted second order n-electron valence perturbation theory (NEVPT2).

To analyze the full absorption spectrum, including possible participation of ligand orbitals, time-dependent density functional theory (TDDFT)[25] calculations were performed using the B3LYP functional. Natural transition orbitals[26] were plotted to visualize shifting of transition electron density for excited states of interest, as shown in Figures S17-S20. State mixing due to spin-orbit coupling is treated via quasi-degenerate perturbation theory in the basis of 6 triplet and 12 singlet states, from which we estimate ZFS axial and transverse parameters, *D* and *E*, using an effective Hamiltonian approach. The calculated values are listed in Table S14. We observe that D increases from complexes **1** through **3**, in agreement with the experimental trend (Table 1). Complex **4**, however, does not follow the trend. We have confirmed that basis set effects are not significant, changing *D* and *E* by less than 0.2 cm^{-1} when larger basis sets are used. The calculated values of *D* differ from experiment by 20–100%, which is typical for first-row transition metals. The increasing error with increasing halide atomic number suggests that relativistic effects of the halide are not being adequately captured, requiring more sophisticated treatments, including larger active spaces.

Other physical measurements.

Direct-current (dc) and alternating-current (ac) magnetic susceptibility measurements on microcrystalline powdered samples of **1–4** were performed with a Quantum Design MPMS-XL SQUID

magnetometer prepared under an inert atmosphere and secured with eicosane. Matrix-Assisted Laser Desorption Ionization Time of Flight (MALDI-TOF) mass spectra were measured on a high-resolution Bruker Autoflex LRF Speed mass spectrometer in reflector negative ion mode. Samples were prepared with anthracene as a matrix. Inductively coupled plasma optical emission spectrometry (ICP-OES) data were collected on an Agilent ICP-OES 5100 VDV spectrometer in MIT.nano to determine the experimental Cr:Ti ratio in diluted crystalline samples. This ratio was quantitatively determined using a "blank" 0 ppm sample of 3.5% nitric acid solution as well as standards of 5 ppm, 25 ppm, 50 ppm, 100 ppm, and 250 ppm of Cr and Ti in 3.5% nitric acid solution. Standards were prepared from stock solutions of 1002 ± 4 μg/mL chromium and 999 ± 5 μg/mL titanium procured from Inorganic Ventures. The results of this analysis are listed in Table S12. A Bruker Alpha II Compact FT-IR spectrometer equipped with an attenuated total reflectance accessory was used to collect Infrared spectra. Ultraviolet-visible-near infrared (UV-Vis-NIR) electronic absorption spectra were collected on 0.1–0.6 mM samples in THF with a Varian Cary 5000 spectrophotometer. Molar absorptivity coefficient values (ε, M^{-1} cm^{-1}) were determined via Beer-Lambert law in the 0.1–0.6 mM range, where raw absorbance values increase linearly with concentration. Peak minima of each spectrum′s second derivative were identified to determine all distinguishable peaks. Cyclic voltammetry experiments were conducted on a CH Instruments Electrochemical Workstation on THF solutions of complexes prepared under a N_2 atmosphere at a concentration of ~ 1 mM. $[^nBu_4N](PF_6)$ was used as the supporting electrolyte at concentrations of ~ 0.1 M. These experiments used a three-electrode cell comprised of a silver reference electrode, a glassy carbon working electrode, and a platinum counter electrode.

Analysis by τ_5.

The structural parameter τ_5, a unitless value ranging from 0 to 1, describes the geometry of a 5-coordinate compound based on valence angle.[27] A value of 0 indicates adherence to perfect square pyramidal geometry, while a value of 1 indicates adherence to perfect trigonal bipyramidal geometry. This parameter is calculated from the two largest valence angles of a coordination complex, β and α (β > α).

$$\tau_5 = \frac{\beta - \alpha}{60^\circ}$$

Analysis by Continuous Shape Measurements (CShM).

CShM values were calculated through the geometry analysis software SHAPE 2.1,[28] where TBP indicates adherence to the trigonal bipyramidal (D_{3h}) geometry, and SPY indicates adherence to the square pyramidal (C_{4v}) geometry. Continuous Shape Measurements calculate the position of the geometry along the continuous shape pathway between these two 5-coordinate geometries. A CShM value of 0 corresponds to perfect TBP geometry, while a value of 5.383 corresponds to perfect SPY geometry. Values greater than 5.383 correspond to a geometric distortion that is not along the continuous shape pathway between these two geometries.

Discussion of geometric analyses.

The structural parameter τ_5, a value ranging from 0 to 1, evaluates a 5-coordinate complex′s adherence to either a square pyramidal (0) or trigonal bipyramidal (1) geometry through an analysis of the two largest valence angles of the complex. All complexes possessed values of τ_5 ranging from 0.98 to 1 (Table S1), indicating a strong adherence to trigonal bipyramidal geometry through the lens of bond angles.

The Angular Overlap Model.

The angular overlap model allows us to predict how the relative energies of these orbitals change with halide identity.[29] The energies of d orbitals in trigonal bipyramidal geometry are given by:

$$\boldsymbol{E(z^2) = 2e_\sigma(L_{ax}) + (3/4)e_\sigma(L_{eq}) - E_{ds}}$$
$$\boldsymbol{E(x^2 - y^2, xy) = 9/8e_\sigma(L_{eq}) + (3/2)e_\pi(L_{eq})}$$
$$\boldsymbol{E(xz) = E(yz) = 2e_\pi(L_{ax}) + (3/2)e_\pi(L_{eq})}$$

Where the effect of d-s mixing is represented by the term E_{ds}. Changes in the identity of the apical halide will change the energies of the d_{xz}, $d_{yz,}$ and $d_z{}^2$. This can be quantified using the parameters e_σ and e_π. Although we did not perform a full AOM analysis for these complexes, we can predict a trend using reported values for halides in comparable geometries. Notably, in Cr^{3+} in O_h geometry, we see a decrease in both parameters with increased size, with a greater change observed in sigma bonding (Table S3). This would imply a greater decrease in energy for the $d_z{}^2$ orbital compared to the d_{xz}, d_{yz} orbitals.

SOC and Zero Field Splitting.

The increase in ZFS across the series is produced by the changes in the interaction between ground and excited electronic states through spin-orbit coupling (SOC). The effect of SOC on *D* and *E* is inversely proportional to the energy gap between states of relevant symmetry and proportional to the magnitude of the SOC constant ξ_{eff} . The sign of this contribution to *D* is also dependent on the symmetry relation of interacting orbitals.[30,31] For d^2 complexes of C_{3v} geometry, the degenerate orbital set {d_{xz}, d_{yz}} is related to the {d_{xy}, $d_{x^2-y^2}$} orbital set by the L_z operator, so the {d_{xy}, $d_{x^2-y^2}$} orbital set contributes negatively to *D*. The inverse is true for the orbital {$d_z{}^2$}, which is related by the L_{xy} operator and so the {$d_z{}^2$} orbital contributes positively to *D*.[32,33] With the support of our calculation of orbital energies, the angular overlap model allows us to predict how the relative energies of these orbitals change with halide identity. As we move down the group to heavier halides, the e_σ and e_π parameters of the halide ions decrease due to lower overlap between the ligand orbitals and the metal d orbitals.[29] The decrease in overlap stabilizes the energy of axial orbitals, with a greater decrease expected for those involved in sigma interactions {$d_z{}^2$}. The {$d_z{}^2$} orbital therefore lowers in energy at a quicker rate than the {d_{xz}, d_{yz}} set, resulting in an overall decrease in the energy gap between the degenerate {d_{xz}, d_{yz}} orbitals and the {$d_z{}^2$} orbital and an increase in the energy gap with the degenerate {d_{xy}, $d_{x^2-y^2}$}, leading to a larger positive contribution and smaller negative contribution to *D* respectively down the series (Figure 3).

This trend in decrease in donor strength with halide size is also supported by the decrease in the Cr^{4+}/Cr^{3+} reduction potential for **1–4** (Figure S23). **1** therefore displays the lowest magnitude of axial ZFS due to its stronger bonding and illustrates how increasing the donor strength of the ligand at the apical site could further minimize *D* to pursue addressability at commercial microwave frequency ranges.

Fitting VFPL Spectra.

To fit VFPL data, we first used non-linear least squares to deconvolute the 9 T spectra to three Gaussian functions describing relaxation to the three spin sublevels in the $S = 1$ system (Fig. 5b). Using extracted parameters as constants describing the intensity and linewidth of the three peaks, we used a modified Gaussian function to obtain only the three peak centers at each field point. Including only three fitting parameters simplified the deconvolution of the low-field spectra, where some peaks are not resolvable (Fig. 5a). Finally, using these peak centers, intensities, and linewidths as more accurate initial parameters, the spectra were freely fit for all parameters at all fields (Figures S79 and S80). This fitting procedure resulted in the best convergence, or lower standard deviation error, of the parameters by providing more accurate initial parameters for the final complete fitting step.

Table S1 | Calculated τ5 values for **1–4**.

**Complex**	**1**	**2**	**3**	**4**
τ_5	0.99	0.98	1.00	0.98

Table S2 | Calculated CShM values for **1–4**.

**Complex**	**1**	**2**	**3**	**4**
CShM (TBP)	0.24	1.08	1.82	2.96
CShM (SPY)	5.43	6.09	6.86	7.96

Table S3 | AOM Parameters for Cr^{3+} in Octahedral Geometry

**Cr^{3+}**	**e_σ**	**e_π**
F^-	8,200	2,000
Cl^-	5,700	980
Br^-	5,380	950
I^-	4,100	670

Table S4 | Comparison of structural parameters in other ^{TMS}trenCrR systems

**Complex**	**N—M—X angle (°)**	**Cr—X (Å)**	**Cr—N (amino) (Å)**	**$SiMe_3$ ppm (lw)**	**CH_2 ppm (lw)**	**CH_2 ppm (lw)**	**T (K)**
Cr-H[34]	175.8(9)	1.56(3)	2.140(2)	1.56 (170)	−22.2 (1100)	−203 (5900)	180
Cr-Me[35]	179.43(8)	2.078(2)	2.250(2)	4.88 (220)	−29.3 (1200)	−139 (5200)	180
Cr-nBu[35]	178.4(2)	2.074(4)	2.288(2)	4.68 (340)	−25.6 (1600)	−52.7 (2200)	180
Cr-F	179.4(1)	1.8633(2)	2.0693(29)	3.28 (190)	−33.1 (1400)	−208 (5100)	160
Cr-CN[6]	179.06(7)	2.062(2)	2.140(2)	3.49 (220)	−39.0 (1400)	−188 (6900)	180
Cr-Cl[7]	179.4(1)	2.269(2)	2.043(3)	4.47 (220)	−40.8 (1300)	−211 (6000)	273
Cr-Cl [This work]	171.1(8)	2.320(16)	2.149(15)	4.47 (220)	−40.8 (1300)	−211 (6000)	100
Cr-Br[6]	179.0(1)	2.440(1)	2.135(3)	5.17 (250)	−47.4 (1000)	−185 (2700)	180
Cr-I [This work]	178.79(14)	2.670(2)	2.163(3)	5.88 (140)	−54.1 (950)	−145 (2100)	100

Table S5 | Crystallographic information for the structural refinement of **4**

Empirical Formula	$C_{15}H_{39}I_{0.84}CrN_4Si_3Cl_{0.16}$
Formula Weight	523.81 g/mol
Temperature	100 K
Wavelength	0.71073 Å
Crystal System	monoclinic
Space Group	$P2_1/c$
Unit Cell Dimensions	a = 16.1302(9) Å, α = 90 °
	b = 9.7459(5) Å, β = 93.493(2) °
	c = 16.0627(8) Å, γ = 90 °
Volume	2520.4(2) $Å^2$
Z	4
Density (calculated)	1.380 g/cm^3
Absorption coefficient	1.646 mm^{-1}
F000	1081.0
Crystal color	brown
2θ range	4.886 – 62.068°
Index ranges	$-23 \leq h \leq 23$
	$-14 \leq k \leq 14$
	$-21 \leq l \leq 23$
Reflections collected	101110
Independent reflections	8043 [R_{int} = 0.0328, R_{sigma} = 0.0153]
Completeness to θ = 50.5 °	100 %
Absorption correction	Multi-scan
Refinement method	Full-matrix least-squares on F^2
Data / restraints / parameters	8043/1301/452
Goodness-of-fit on F^2 [a]	1.200
Final R indices [I>2σ(I)] [b]	R_1 = 0.0374 %, wR_2 = 0.0837 %
R indices (all data)	R_1 = 0.0420 %, wR_2 = 0.0855 %
Largest diff. peak and hole	1.09/-0.65 e/$Å^{-3}$

[a] GooF = $[\Sigma[w(F_o^2 - F_c^2)^2] / (n-p)]^{1/2}$ where n is the number of reflections and p is the total number of parameters refined. [b] $R_1 = \Sigma||F_o| - |F_c|| / \Sigma|F_o|$; $wR_2 = [\Sigma[w(F_o^2 - F_c^2)^2] / \Sigma[w(F_o^2)^2]]^{1/2}$

Table S6 | Crystallographic information for the structural refinement of **5**

Empirical Formula	$C_{15}H_{39}N_4Si_3TiF$
Formula Weight	426.67g/mol
Temperature	110 K
Wavelength	0.56083 Å
Crystal System	trigonal
Space Group	R3
Unit Cell Dimensions	a = 16.412(4) Å, α = 90 °
	b = 16.412(4) Å, β = 90 °
	c = 7.732(2) Å, γ = 120 °
Volume	1803.7(10) Å^2
Z	3
Density (calculated)	1.178 g/cm^3
Absorption coefficient	0.267 mm^{-1}
F000	690.0
Crystal color	yellow
2θ range	3.916 – 57.906°
Index ranges	$-28 \leq h \leq 23$
	$-28 \leq k \leq 26$
	$-13 \leq l \leq 11$
Reflections collected	37577
Independent reflections	3708 [R_{int} = 0.0714, R_{sigma} = 0.0250]
Completeness to θ = 39.3 °	100 %
Absorption correction	Integration
Refinement method	Full-matrix least-squares on F^2
Data / restraints / parameters	3708/1/76
Goodness-of-fit on F^{2a}	1.037
Final R indices [I>2σ(I)][b]	R_1 = 0.0507 %, wR_2 = 0.1300 %
R indices (all data)	R_1 = 0.0521 %, wR_2 = 0.1324 %
Largest diff. peak and hole	1.32/-0.86 e/Å^{-3}

[a] GooF = $[\Sigma[w(F_o^2-F_c^2)^2] / (n-p)]^{1/2}$ where n is the number of reflections and p is the total number of parameters refined. [b] $R_1 = \Sigma||F_o|-|F_c|| / \Sigma|F_o|$; $wR_2 = [\Sigma[w(F_o^2-F_c^2)^2] / \Sigma[w(F_o^2)^2]]^{1/2}$

Table S7 | Crystallographic information for the structural refinement of **7**

Empirical Formula	$C_{15}H_{39}N_4Si_3TiBr$
Formula Weight	487.58 g/mol
Temperature	110 K
Wavelength	1.54186 Å
Crystal System	Orthorhombic
Space Group	Pnma
Unit Cell Dimensions	a = 16.235(5) Å, α = 90 °
	b = 16.573(6) Å, β = 90 °
	c = 9.416(3) Å, γ = 90 °
Volume	2533.7(14) $Å^2$
Z	4
Density (calculated)	1.278 g/cm^3
Absorption coefficient	6.106 mm^{-1}
F000	1024
Crystal color	Orange
2θ range	10.862 – 141.372 °
Index ranges	$-13 \le h \le 19$
	$-17 \le k \le 20$
	$-11 \le l \le 9$
Reflections collected	15529
Independent reflections	2463 [R_{int} = 0.0216, R_{sigma} = 0.0143]
Completeness to θ = 135.4 °	98.2 %
Absorption correction	Integration
Refinement method	Full-matrix least-squares on F^2
Data / restraints / parameters	2463/1802/434
Goodness-of-fit on F^{2a}	1.097
Final R indices [$I>2\sigma(I)$][b]	R_1 = 0.0276 %, wR_2 = 0.0628 %
R indices (all data)	R_1 = 0.0369 %, wR_2 = 0.0681 %
Largest diff. peak and hole	0.20 and -0.19 e $Å^{-3}$

[a] GooF = $[\Sigma[w(F_o^2-F_c^2)^2] / (n-p)]^{1/2}$ where n is the number of reflections and p is the total number of parameters refined. [b] $R_1 = \Sigma||F_o|-|F_c|| / \Sigma|F_o|$; $wR_2 = [\Sigma[w(F_o^2-F_c^2)^2] / \Sigma[w(F_o^2)^2]]^{1/2}$

Table S8 | Features identified by second derivative UV-vis spectrum in Figure S15.

**Wavelength (**nm**)**	**Molar Absorptivity Coefficient (**M^{-1} cm^{-1}**)**
419(1)	3651(100)
471(1)	4093(30)
575(2)	800(40)
611(3)	369(40)

Table S9 | Features identified by second derivative UV-vis spectrum in Figure S16.

**Wavelength (**nm**)**	**Molar Absorptivity Coefficient (**M^{-1} cm^{-1}**)**
426(1)	2345(90)
475(1)	2861(30)
523(3)	1895(60)

Table S10 | Features identified by second derivative UV-vis spectrum in Figure S17.

**Wavelength** (nm)	**Molar Absorptivity Coefficient** (M^{-1} cm^{-1})
436(1)	4312(40)
487(1)	3576(100)

Table S11 | Features identified by second derivative UV-vis spectrum in Figure S18.

**Wavelength** (nm)	**Molar Absorptivity Coefficient** (M^{-1} cm^{-1})
388(1)	3403(40)
458(1)	5531(30)
500(3)	3734(170)
685(3)	422(10)

Table S12 | Cr^{4+}:Ti^{4+} ratios determined from ICP-OES.

Sample	Notation	Dilution Percentage by ICP-OES (%)
**1** diluted in **5** (Powder)	**1′**	0.47
**2** diluted in **6** (Powder)		1.3
**3** diluted in **7** (Powder)	**3′**	0.13
**4** diluted in **8** (Powder)	**4′**	1.1
**2** diluted in **5** (SC)	**2′**	0.61
**3** diluted in **6** (SC)	**3′**	0.51
**4** diluted in **7** (SC)	**4′**	0.17
**2** diluted in **5** (SC)		0.38
**2** diluted in **7** (SC)		0.22
**2** diluted in **8** (SC)		0.27

Table S13 | Orbital contributions of molecular orbitals composing the active space used in CASSCF (4e, 8o) calculations for **1**.

F	do	d1	d2	d3	d4	d5	v1	v2
energy (H)	−0.42693	−0.08778	−0.07138	0.06437	0.09088	0.14857	1.03175	1.03656
occupation	1.81019	0.7053	0.65371	0.29638	0.31154	0.21506	0.00401	0.00381
Cr dz2	0.5	0.0	0.0	5.8	3.7	71.4	0.0	0.0
Cr dxz	0.4	0.1	90.4	2.5	0.3	0.5	0.6	60.2
Cr dyz	0.5	90.5	0.1	0.4	2.6	0.0	63.6	0.0
Cr dx2y2	29.2	0.7	3.1	37.6	20.5	6.5	0.5	8.9
Cr dxy	6.6	2.7	0.3	21.5	52.6	0.2	6.9	1.9
N px	21.3	0.4	0.3	6.9	1.6	0.3	0.0	0.0
N px	13.0	0.0	0.0	4.3	0.5	4.2	0.0	0.0
N dxz	0.0	0.0	0.0	0.0	0.0	0.0	0.0	5.3

Table S14 | Visualization of molecular orbitals composing the active space used in CASSCF (4e, 8o) calculations of **1**, plotted with an isosurface value of 0.01 a.u.

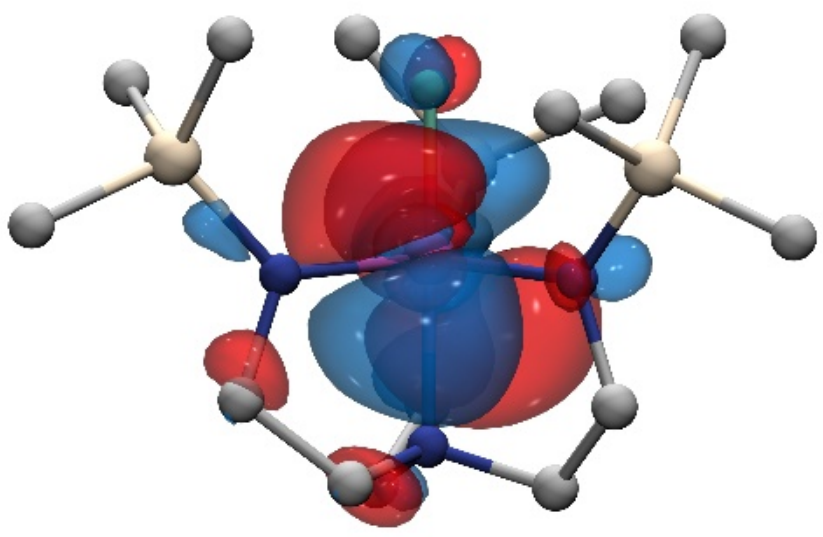

v1

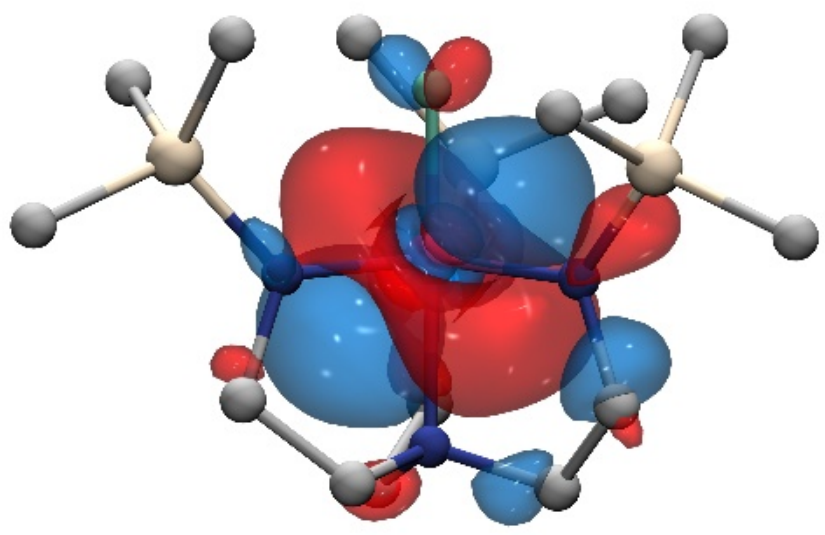

v2

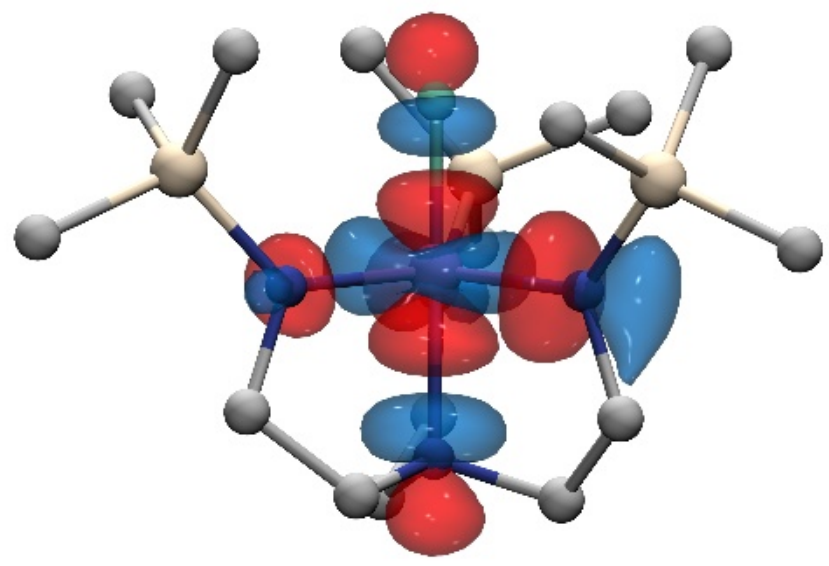

d5

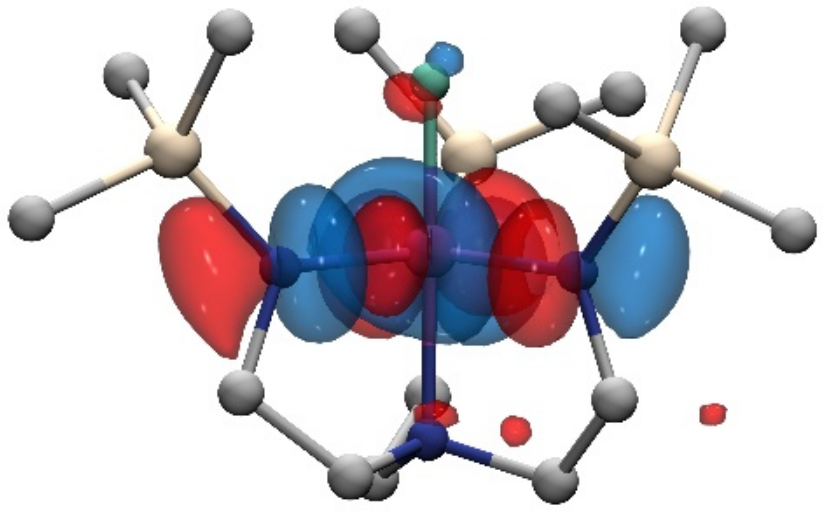

d3

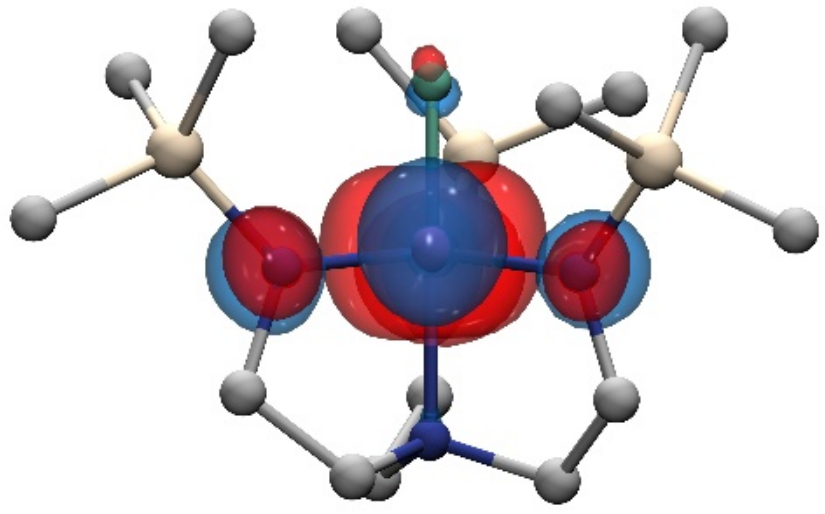

d4

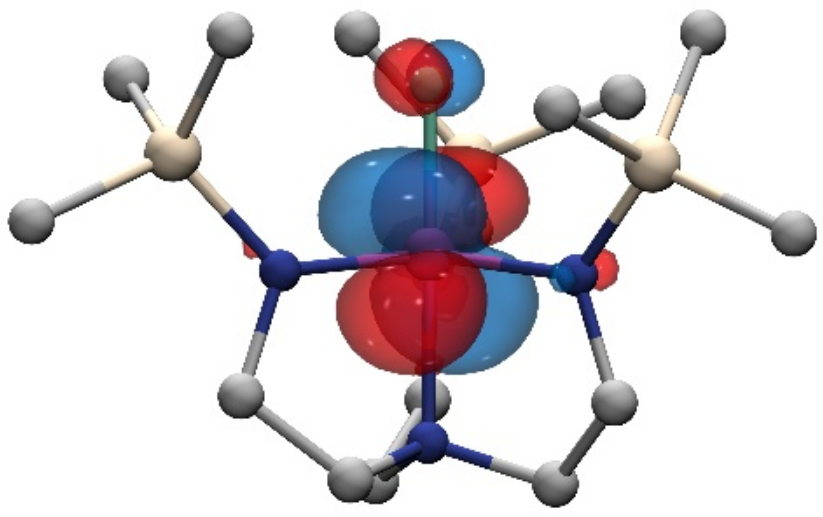

d1

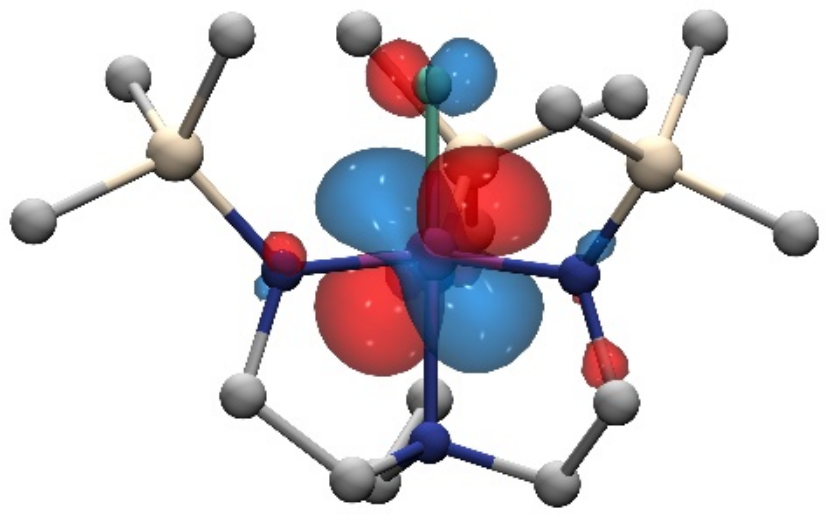

d2

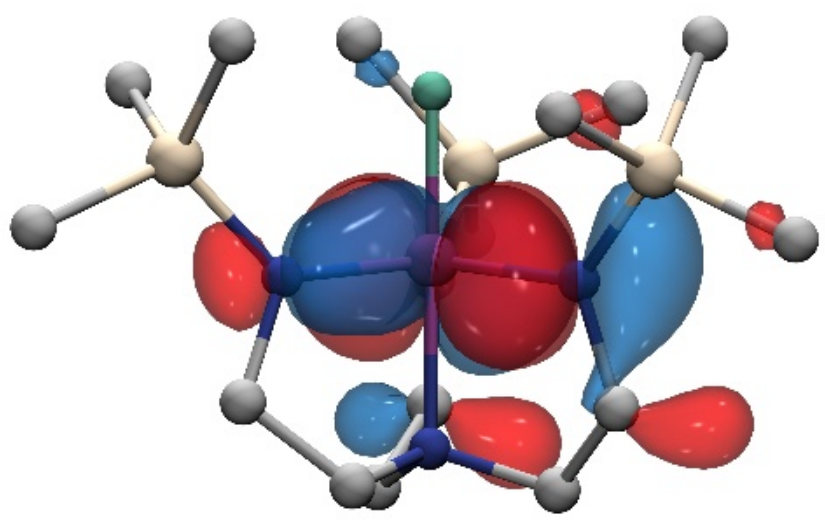

do

Table S15 | Orbital contributions of molecular orbitals composing the active space used in CASSCF (4e, 8o) calculations for **2**.

**Cl**	**do**	**d1**	**d2**	**d3**	**d4**	**d5**	**v1**	**v2**
energy (H)	−0.44378	−0.10098	−0.08068	0.07196	0.08439	0.11147	0.95472	0.96398
occupation	1.83061	0.70596	0.64358	0.29048	0.30046	0.2204	0.00437	0.00412
Cr dz2	0.0	0.0	0.0	18.8	0.4	58.9	0.0	0.0
Cr dxz	0.2	58.7	32.5	0.1	2.0	0.0	51.6	15.9
Cr dyz	0.3	32.2	59.6	1.2	0.0	0.3	15.5	51.6
Cr dx2y2	0.0	0.0	1.1	2.4	71.8	2.2	1.2	1.0
Cr dxy	34.1	2.0	0.4	48.3	4.4	12.5	1.6	1.3
N py	12.8	0.1	0.0	7.2	0.3	0.1	0.1	0.0
N py	8.9	0.1	0.0	0.0	0.0	0.0	0.0	0.0
N py	18.5	0.0	0.0	0.0	0.0	0.0	0.0	0.0

Table S16 | Visualization of molecular orbitals composing the active space used in CASSCF (4e, 8o) calculations of **2**, plotted with an isosurface value of 0.01 a.u.

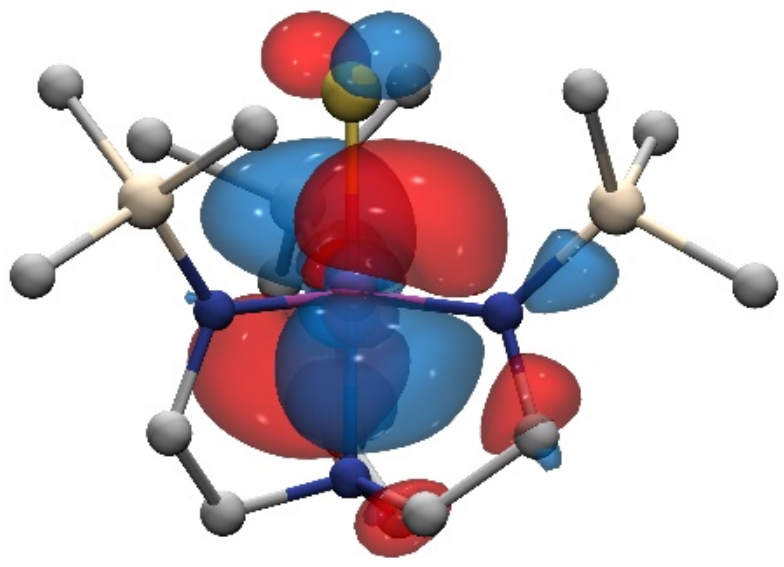

v1

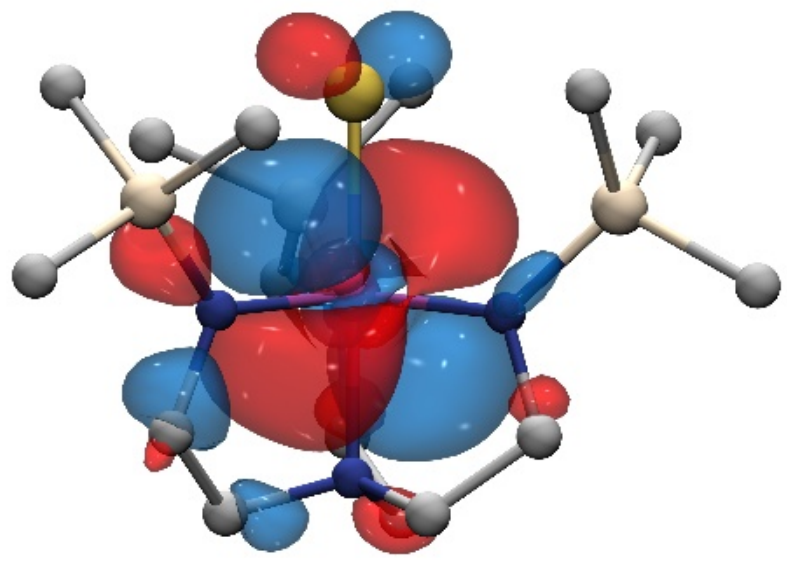

v2

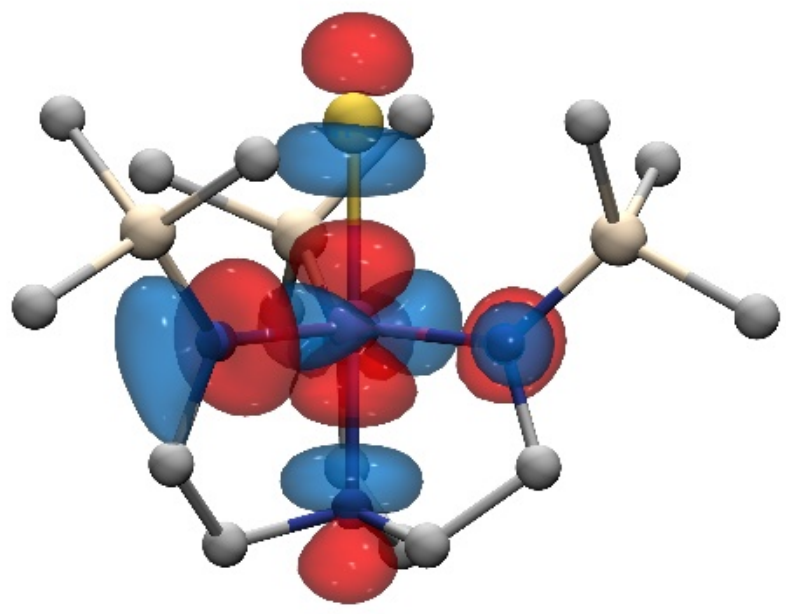

d5

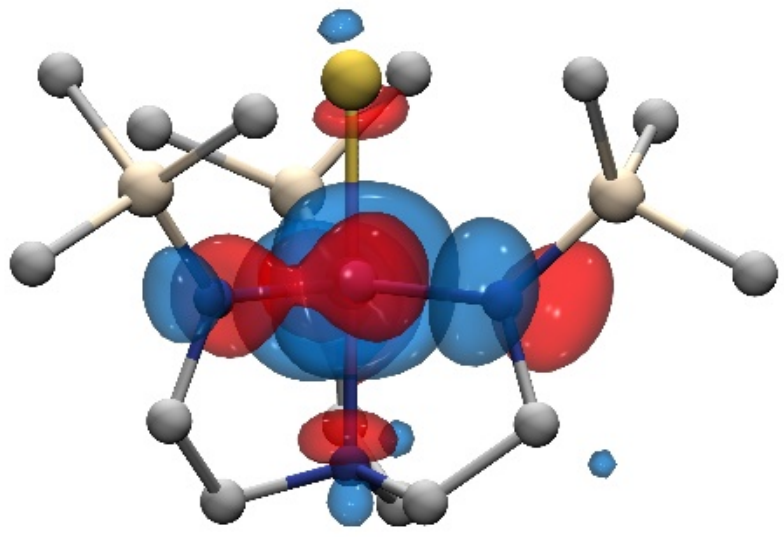

d3

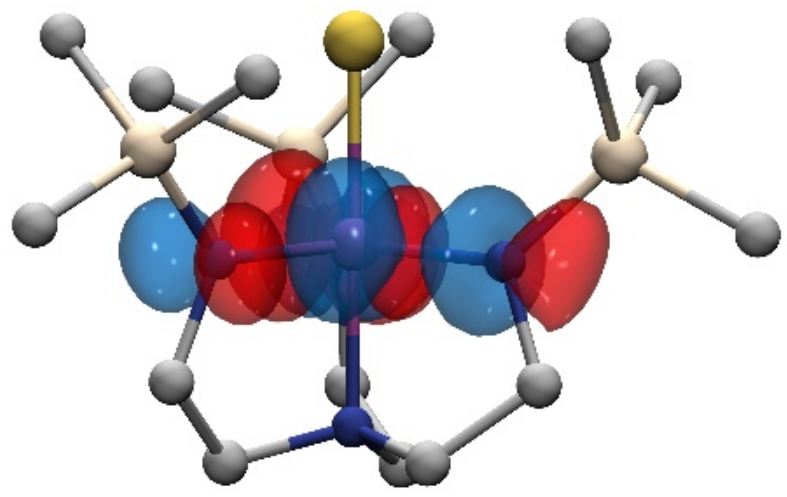

d4

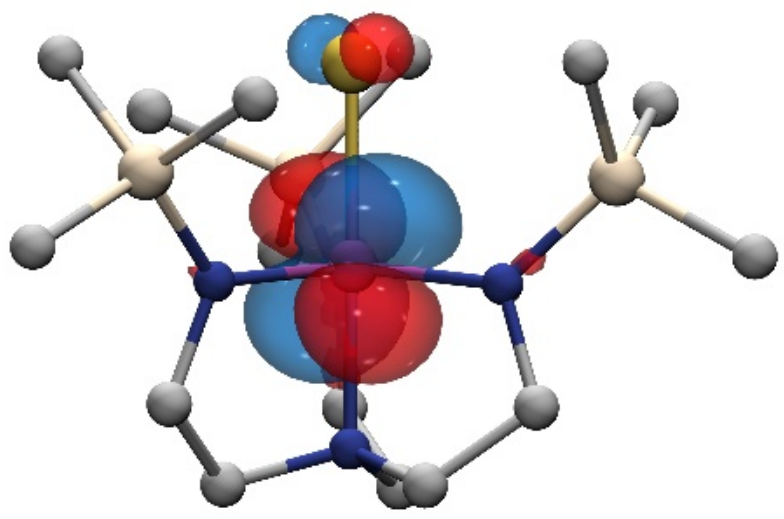

d1

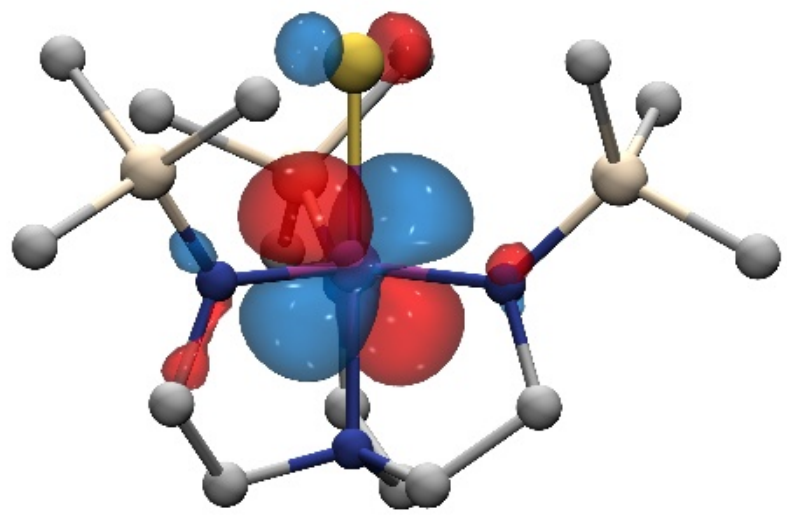

d2

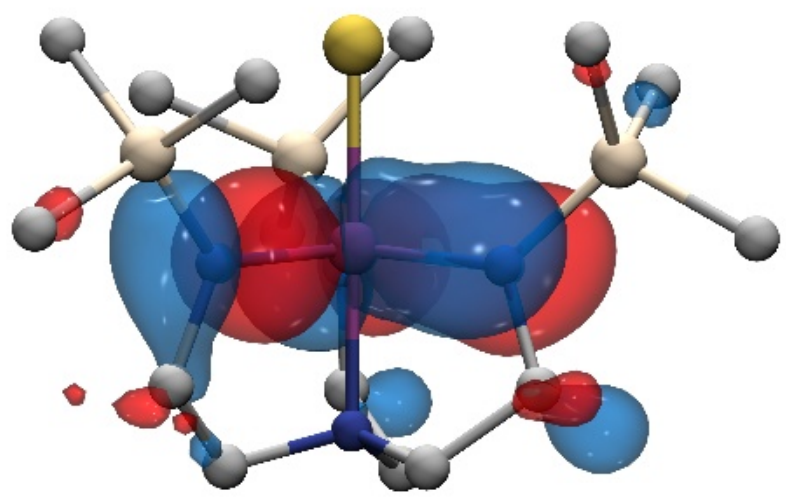

do

Table S17 | Orbital contributions of molecular orbitals composing the active space used in CASSCF (4e, 8o) calculations for **3**.

**Br**	**do**	**d1**	**d2**	**d4**	**d4**	**d5**	**v1**	**v2**
energy (H)	−0.43991	−0.09793	−0.08409	0.07337	0.07816	0.08289	0.94909	0.96078
occupation	1.82931	0.69041	0.64438	0.30099	0.28427	0.24223	0.00436	0.00404
Cr dz2	0.0	0.1	0.0	31.8	7.5	39.3	0.0	0.0
Cr dxz	0.0	4.0	87.0	1.3	0.7	0.5	1.8	64.6
Cr dyz	2.0	86.1	3.4	0.1	2.0	0.7	67.5	1.6
Cr dx2y2	32.9	1.4	0.7	31.3	2.2	31.3	0.4	3.0
Cr dxy	2.8	2.1	2.3	10.5	63.0	0.0	0.0	0.0
N px	15.8	0.3	0.1	3.9	1.3	0.0	0.0	0.0
N px	12.2	0.0	0.2	4.3	0.6	0.0	0.0	0.0
N px	7.4	0.0	0.1	0.8	0.1	6.9	0.0	0.0

Table S18 | Visualization of molecular orbitals composing the active space used in CASSCF (4e, 8o) calculations of **3**, plotted with an isosurface value of 0.01 a.u.

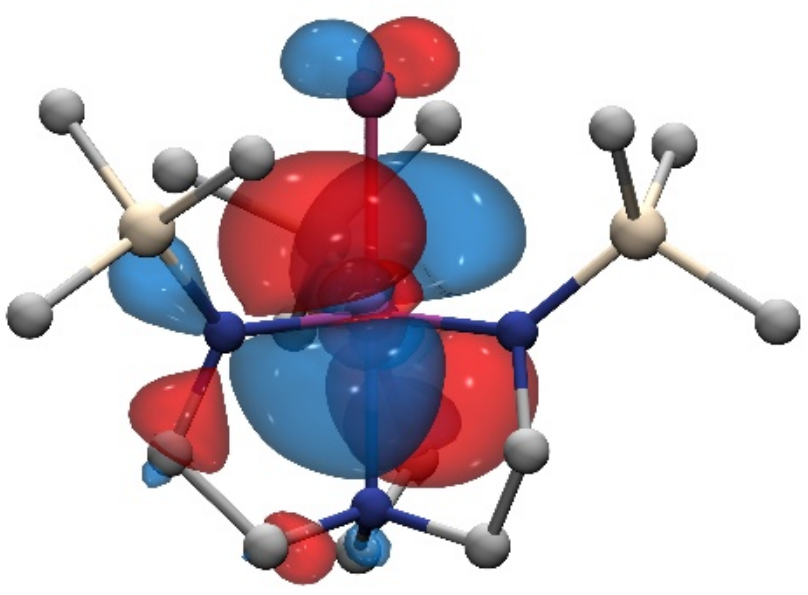

v1

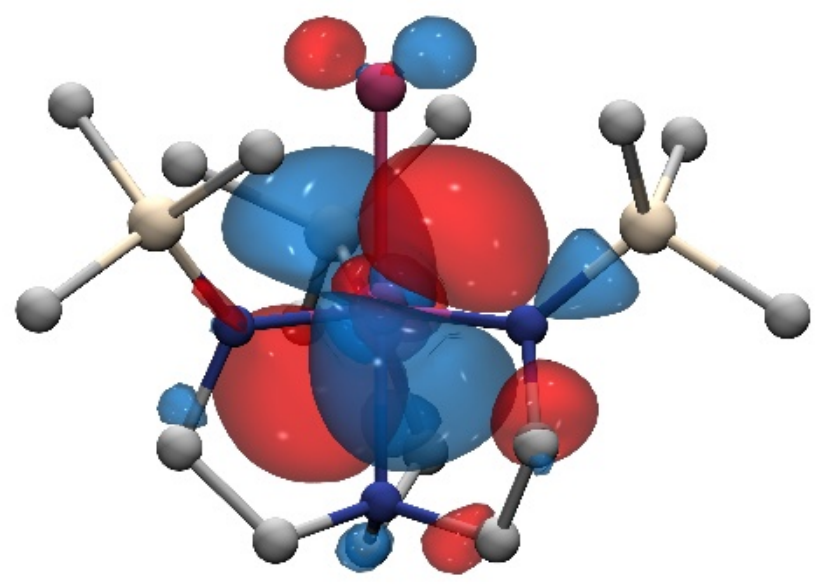

v2

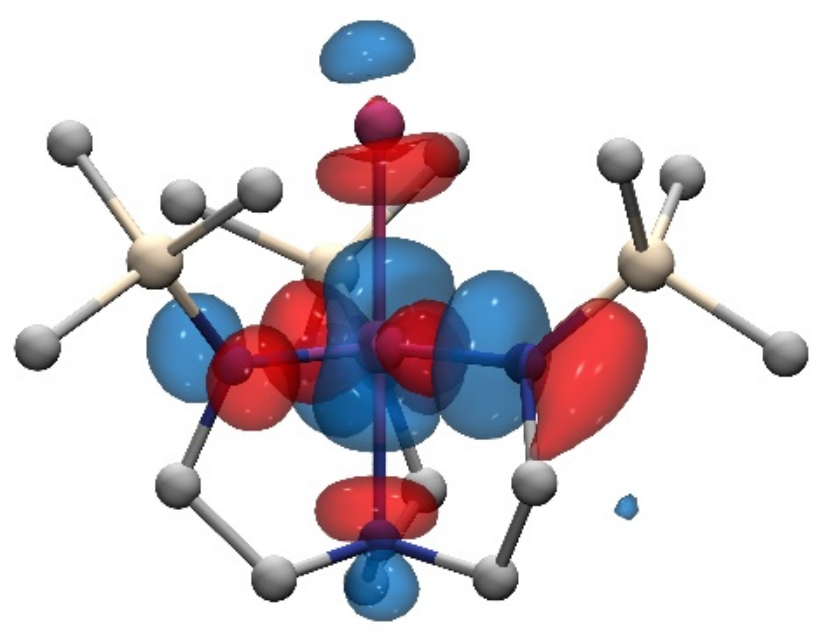

d5

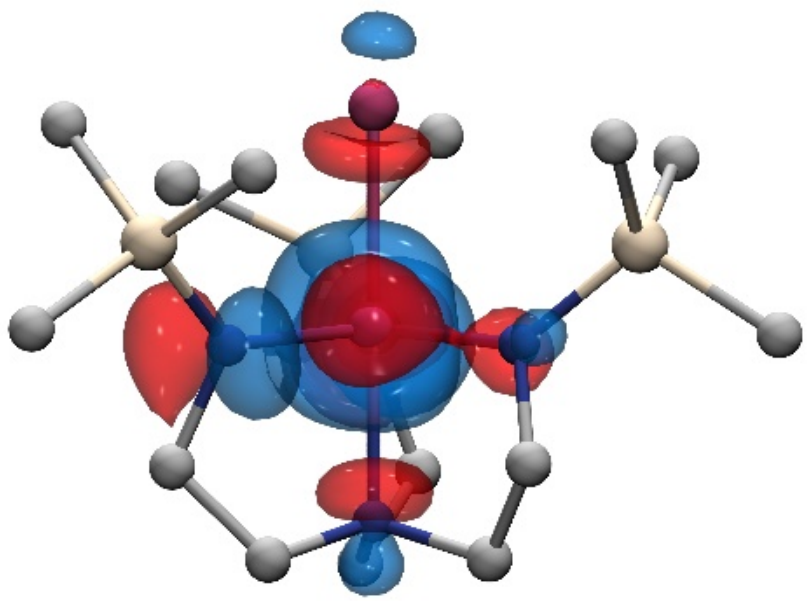

d3

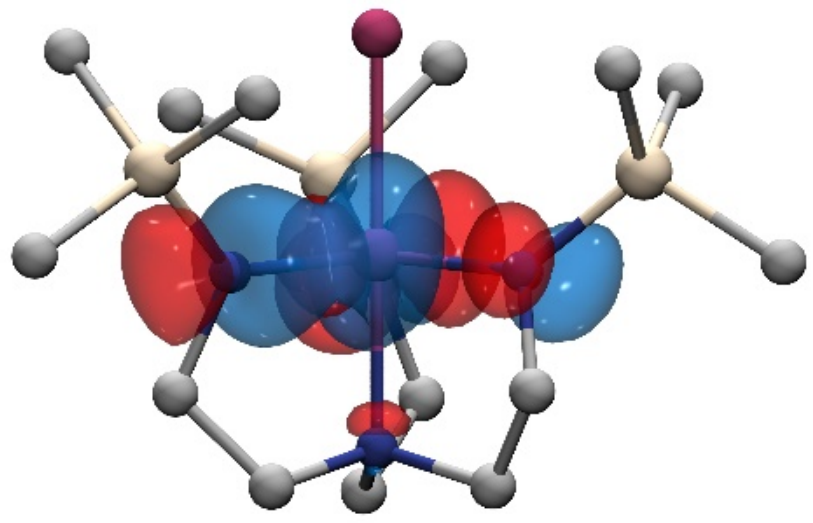

d4

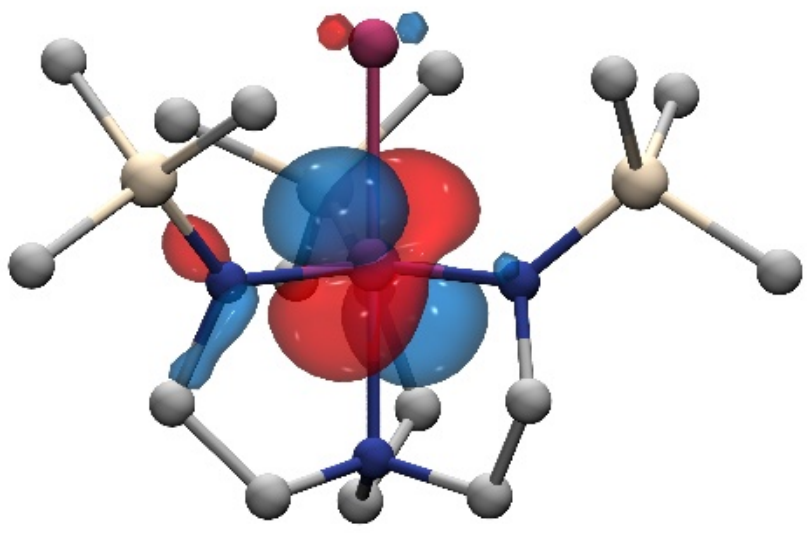

d1

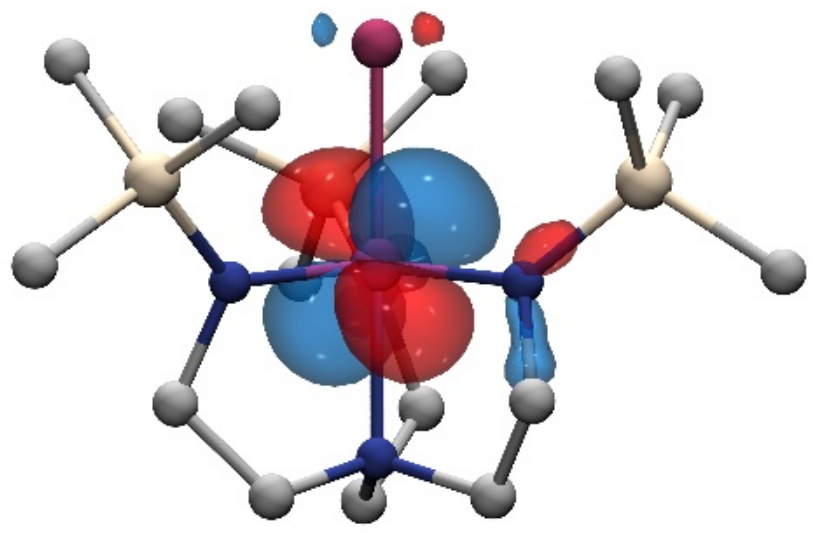

d2

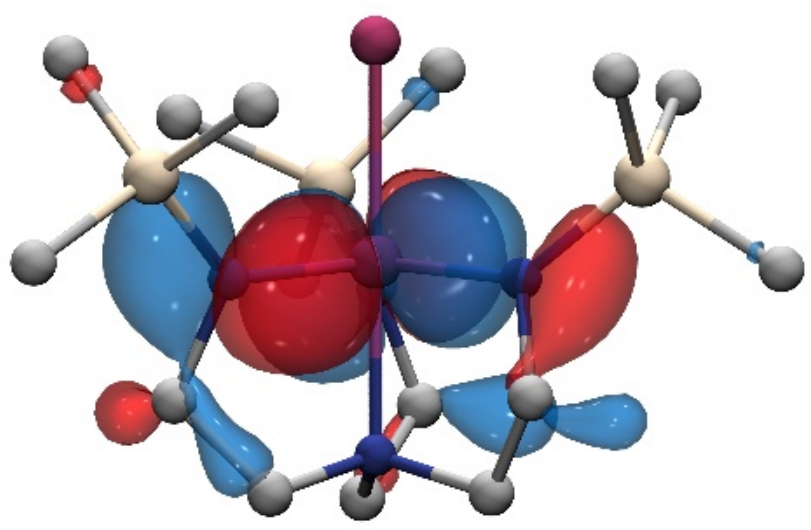

do

Table S19 | Orbital contributions of molecular orbitals composing the active space used in CASSCF (4e, 8o) calculations for **4**.

**I**	**do**	**d1**	**d2**	**d5**	**d4**	**d4**	**v1**	**v2**
energy (H)	−0.44403	−0.09841	−0.07704	0.0666	0.06837	0.08276	0.94171	0.95821
occupation	1.83605	0.69182	0.62689	0.24415	0.31103	0.28165	0.0044	0.004
Cr dz2	0.2	0.0	0.0	25.8	49.8	2.3	0.0	0.0
Cr dxz	0.2	5.3	83.9	0.3	0.4	2.6	2.3	63.6
Cr dyz	5.4	81.5	5.3	2.2	0.8	0.1	66.8	3.6
Cr dx2y2	20.7	4.9	0.8	30.8	24.0	11.2	1.8	3.3
Cr dxy	11.3	1.2	2.8	9.4	0.3	60.8	0.1	1.9
N py	9.6	0.2	0.3	0.0	0.1	0.0	0.0	0.0
N px	12.3	0.4	0.1	0.0	3.7	0.0	0.0	0.0
N px	0.0	0.0	0.0	5.2	0.0	1.3	0.0	0.0

Table S20 | Visualization of molecular orbitals composing the active space used in CASSCF (4e, 8o) calculations of **4**, plotted with an isosurface value of 0.01 a.u.

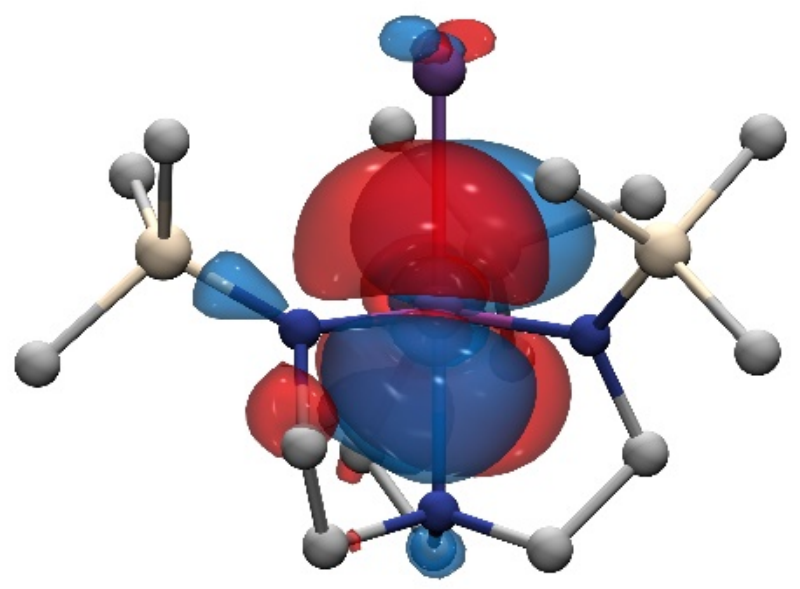

v1

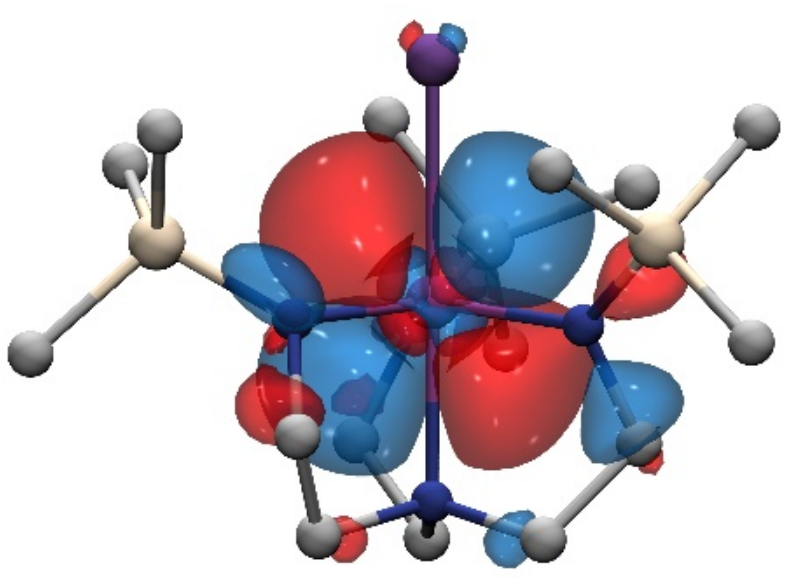

v2

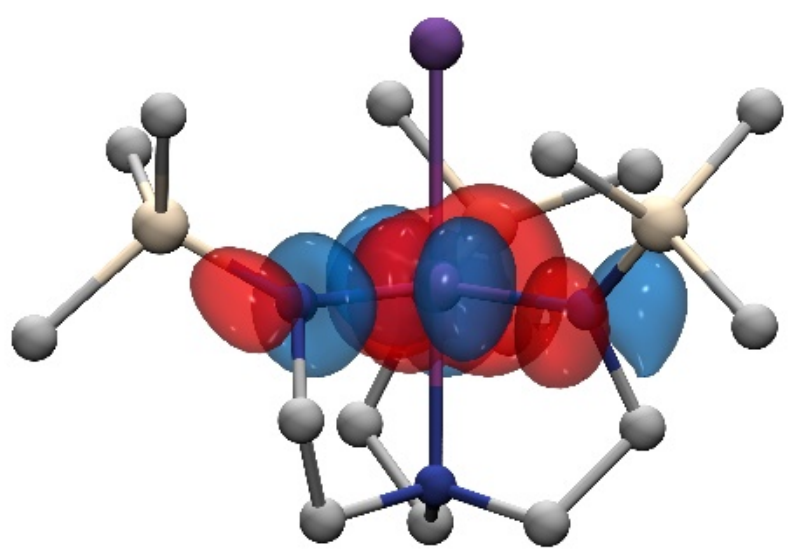

d5

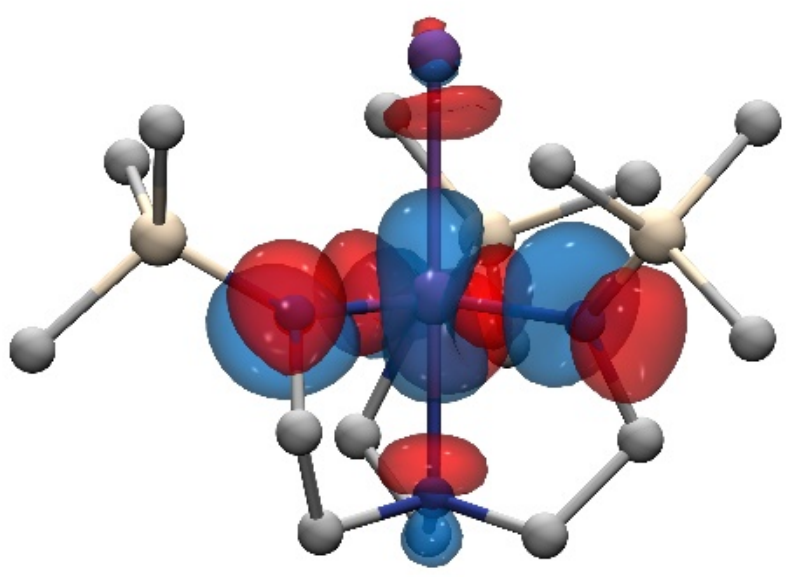

d3

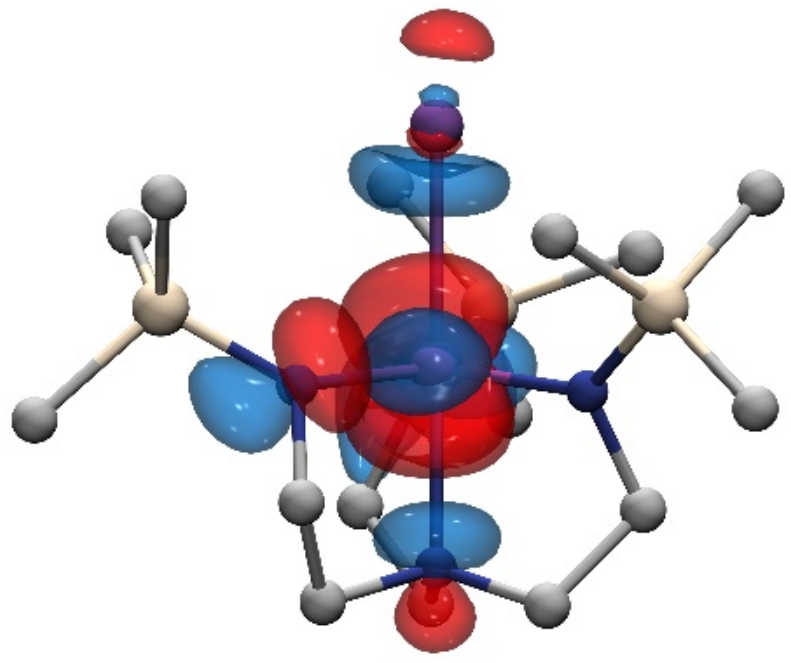

d4

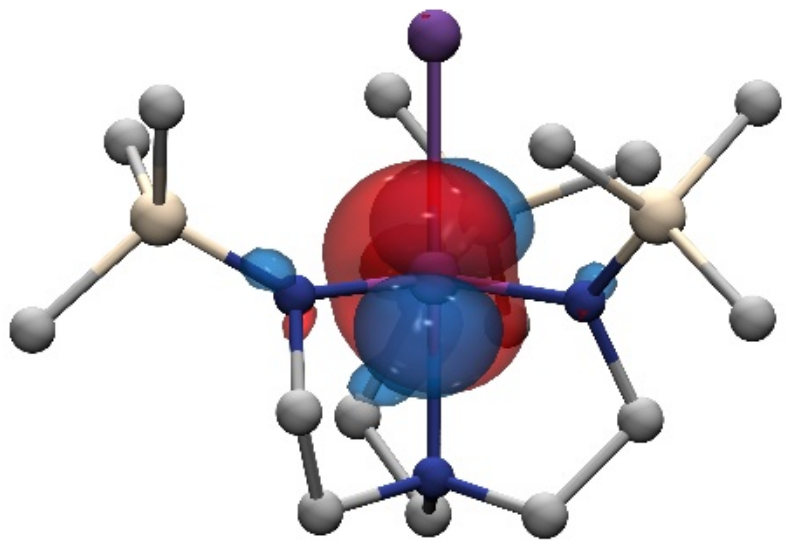

d1

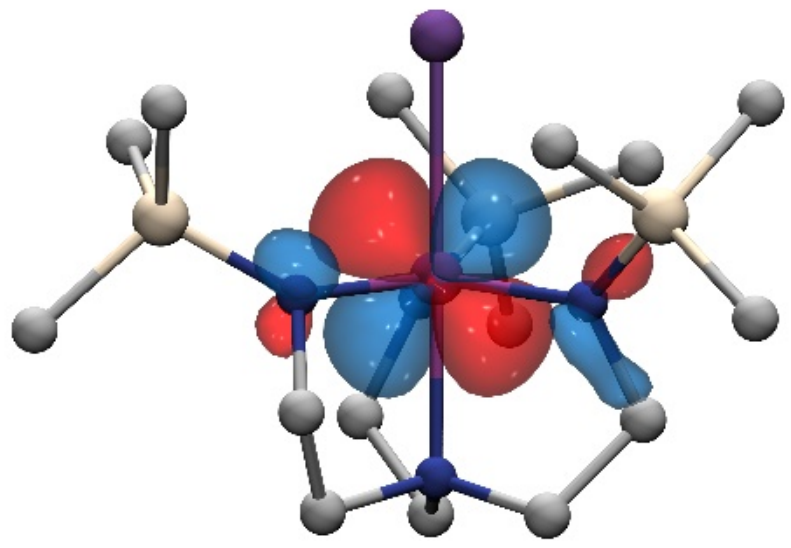

d2

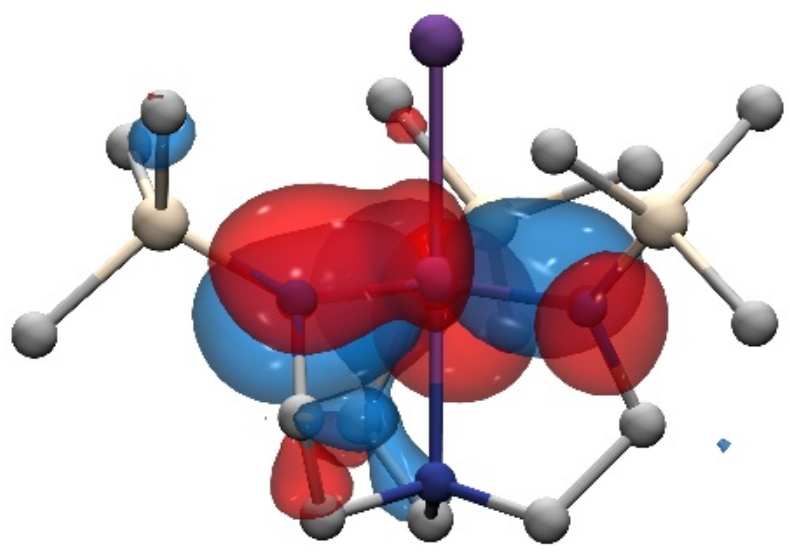

do

Table S21 | Ground state to triplet excited state transition energies (nm) from CASSCF (4e, 8o) with NEVPT2.

State	1	2	3	4
1	526.1	492.2	521.6	563.4
2	517.0	481.1	508.9	536.2
3	443.6	424.9	442.7	462.8
4	438.4	420.2	433.2	455.7
5	341.5	342.8	364.0	389.1

Table S22 | ZFS and g-factor data from CASSCF (4e, 8o) with NEVPT2.

	1	2	3	4
D (cm^{-1})	4.231	4.263	4.510	3.669
E (cm^{-1})	0.1480	0.1853	0.0501	0.0620
E/D	0.0350	0.0435	0.0111	0.0169
g_{iso}	1.967	1.970	1.969	1.964

Table S23 | Orbital energies (eV) from CASSCF (4e, 8o).

	1	2	3	4
d1	0.00	0.00	0.00	0.00
d2	0.45	0.55	0.38	0.58
d3	4.14	4.71	4.66	4.49
d4	4.86	5.04	4.79	4.54
d5	6.43	5.78	4.92	4.93

Table S24 | Orbital energies (cm^{-1}) from CASSCF (4e, 8o).

	1	2	3	4
d1	0.00	0.00	0.00	0.00
d2	3600	4400	3100	4700
d3	33400	38000	37600	36200
d4	39200	40700	38600	36600
d5	51900	46600	39700	39800

Table S25 | Triplet-state ZFS contributions and configurations as determined by CASSCF (4e, 8o) with NEVPT2 for **1**.

**Root**	**ZFS, D**	**ZFS, E**	**NEVPT2 (cm^{-1})**	**Weight**	**Configuration**							
					**do**	**d1**	**d2**	**d3**	**d4**	**d5**	**v1**	**v2**
ground			0	0.94246	↿⇂	↿	↿					
1	1.133	1.272	19007.3									
				0.6343	↿⇂	↿			↿			
				0.12665	↿⇂		↿			↿		
				0.06034	↿⇂		↿		↿			
				0.05878	↿⇂	↿				↿		
2	1.098	−1.097	19341.2									
				0.71371	↿⇂		↿		↿			
				0.16454	↿⇂	↿				↿		
3	0.407	0.661	22545.3									
				0.70933	↿⇂		↿	↿				
				0.12157	↿⇂	↿			↿			
				0.0771	↿⇂		↿			↿		
4	0.689	−0.696	22811.9									
				0.49474	↿⇂	↿				↿		
				0.39204	↿⇂	↿		↿				
5	0.02	−0.031	29282.9									
				0.32597	↿⇂	↿		↿				
				0.21569	↿⇂	↿				↿		
				0.13098	↿⇂		↿	↿				
				0.12595	↿⇂	↿			↿			
				0.06403	↿⇂		↿		↿			

Table S26 | Singlet-state ZFS contributions and configurations as determined by CASSCF (4e, 8o) with NEVPT2 for **1**.

**Root**	**ZFS, D**	**ZFS, E**	**NEVPT2 (cm^{-1})**	**Weight**	**Configuration**							
					**do**	**d1**	**d2**	**d3**	**d4**	**d5**	**v1**	**v2**
0	0	0	12283.7									
				0.3813	↿⇂	↿⇂						
				0.37502	↿⇂		↿⇂					
				0.19549	↿⇂	⇂	↿					
1	0.02	0	12553.6									
				0.75469	↿⇂	⇂	↿					
				0.12468	↿⇂	↿⇂						
				0.07373	↿⇂		↿⇂					
2	2.843	−0.003	21050.3									
				0.46306	↿⇂		↿⇂					
				0.40981	↿⇂	↿⇂						
3	−0.826	−0.841	29314.9									
				0.59445	↿⇂	⇂			↿			
				0.10723	↿⇂		⇂	↿				
				0.07951	↿⇂	⇂		↿				
				0.05248	↿⇂		⇂		↿			
				0.05211	↿⇂		⇂			↿		

4	−0.779	0.776	29437.8									
				0.72433	↿⇂		⇂		↿			
				0.1221	↿⇂	⇂				↿		
5	−0.086	−0.212	32543.4									
				0.55267	↿⇂		⇂	↿				
				0.2007	↿⇂	⇂			↿			
				0.07603	⇂	↿⇂	↿					
6	−0.096	0.11	30894.3									
				0.54798	↿⇂	⇂		↿				
				0.19292	↿⇂	⇂				↿		
				0.11203	⇂	↿	↿⇂					
				0.05527	↿⇂		⇂		↿			
7	−0.246	0.252	36137.6									
				0.56633	↿⇂	⇂				↿		
				0.14998	↿⇂	⇂		↿				
				0.1301	↿⇂		⇂	↿				
				0.05774	↿⇂	⇂			↿			
8	−0.155	−0.096	36074.4									
				0.82091	↿⇂		⇂			↿		
9	0.043	0.035	40338.4									
				0.41611	⇂	↿	↿⇂					
				0.14377	⇂	↿⇂	↿					
				0.13158	⇂	⇂	↿	↿				
10	0.052	0.016	41434.7									
				0.39636	⇂	↿⇂	↿					
				0.18894	⇂	↿	↿⇂					
11	0.096	0	47582									
				0.41782	⇂	⇂	↿		↿			
				0.22445	↿⇂				↿⇂			
				0.05892	⇂	↿⇂	↿					
				0.05402	↿⇂					↿⇂		

Table S27 | Triplet-state ZFS contributions and configurations as determined by CASSCF (4e, 8o) with NEVPT2 for **2**.

**Root**	**ZFS, D**	**ZFS, E**	**NEVPT2 (cm^{-1})**	**Weight**	**Configuration**							
					**do**	**d1**	**d2**	**d3**	**d4**	**d5**	**v1**	**v2**
ground			0	0.94948	↑↓	↑	↑					
1	1.293	1.146	20317.4									
				0.61784	↑↓	↑			↑			
				0.12057	↑↓		↑			↑		
				0.10836	↑↓		↑	↑				
				0.0918	↑↓		↑		↑			
2	0.907	−0.881	20784.8									
				0.60859	↑↓		↑		↑			
				0.16078	↑↓	↑				↑		
				0.07977	↑↓		↑		↑			
				0.07738	↑↓	↑			↑			
3	0.359	0.403	23537.1									
				0.78224	↑↓		↑	↑				
				0.15534	↑↓	↑			↑			
4	0.524	−0.557	23800.3									
				0.68635	↑↓	↑				↑		
				0.21028	↑↓	↑		↑				
5	0.006	−0.006	29169.6									
				0.62468	↑↓	↑		↑				
				0.18136	↑↓		↑		↑			
				0.08741	↑↓	↑				↑		

Table S28 | Singlet-state ZFS contributions and configurations as determined by CASSCF (4e, 8o) with NEVPT2 for **2**.

**Root**	**ZFS, D**	**ZFS, E**	**NEVPT2 (cm^{-1})**	**Weight**	**Configuration**							
					**do**	**d1**	**d2**	**d3**	**d4**	**d5**	**v1**	**v2**
0	0.016	0	11815.8									
				0.53452	↑↓	↑↓						
				0.42175	↑↓		↑↓					
1	0.001	0	12113.1									
				0.95752	↑↓	↓	↑					
2	3.143	0.004	21050.3									
				0.49974	↑↓		↑↓					
				0.39312	↑↓	↑↓						
3	−0.881	−0.839	30078.7									
				0.6097	↑↓	↓			↑			
				0.23463	↑↓		↓	↑				
				0.05077	↑↓		↓			↑		
4	−0.866	0.837	30667.5									
				0.64038	↑↓		↓		↑			
				0.24834	↑↓	↓				↑		
5	−0.165	−0.117	34234.2									
				0.43645	↑↓		↓	↑				
				0.21904	↑↓	↓			↑			

				0.09854	↿⇂	⇂		↿				
				0.07114	↿⇂	⇂				↿		
				0.06006	↿⇂		⇂			↿		
6	−0.004	−0.003	33826.9									
				0.72444	↿⇂	⇂		↿				
				0.09548	↿⇂		⇂	↿				
7	−0.174	0.168	33231.5									
				0.58995	↿⇂	⇂				↿		
				0.15661	↿⇂		⇂		↿			
				0.08905	⇂	↿	↿⇂					
				0.06746	↿⇂	⇂		↿				
8	−0.014	−0.033	34734.2									
				0.76114	↿⇂		⇂			↿		
				0.09305	↿⇂		⇂	↿				
				0.0813	⇂	↿⇂	↿					
9	0.002	0.078	40849.3									
				0.64254	⇂	↿	↿⇂					
				0.0632	↿⇂	⇂		↿				
				0.05317	↿⇂		⇂		↿			
10	0.02	−0.017	41585.4									
				0.63698	⇂	↿⇂	↿					
				0.05731	↿⇂		⇂	↿				
				0.0555	↿⇂		⇂			↿		
11	0.076	0.001	50125.3									
				0.33363	⇂	⇂	↿		↿			
				0.30174	↿⇂				↿⇂			
				0.11959	↿⇂			⇂		↿		
				0.07559	↿⇂					↿⇂		

Table S29 | Triplet-state ZFS contributions and configurations as determined by CASSCF (4e, 8o) with NEVPT2 for **3**.

Root	ZFS, D	ZFS, E	NEVPT2 (cm^{-1})	Weight	Configuration							
					**do**	**d1**	**d2**	**d3**	**d4**	**d5**	**v1**	**v2**
ground			0	0.94605	↿⇂	↿	↿					
1	1.186	0.475	19173.4									
				0.31794	↿⇂		↿	↿				
				0.27095	↿⇂	↿		↿				
				0.18763	↿⇂		↿		↿			
				0.13605	↿⇂	↿				↿		
2	1.164	−0.467	19651.6									
				0.36382	↿⇂	↿			↿			
				0.26312	↿⇂		↿		↿			
				0.15209	↿⇂		↿			↿		
				0.08663	↿⇂	↿				↿		
				0.05235	↿⇂	↿		↿				
3	−0.059	0.265	22586.4									
				0.62818	↿⇂	↿				↿		
				0.20561	↿⇂		↿	↿				
				0.05977	↿⇂	↿			↿			
				0.05939	↿⇂		↿		↿			
4	0.239	−0.245	23085.2									
				0.35203	↿⇂		↿	↿				
				0.32236	↿⇂		↿		↿			
				0.13347	↿⇂	↿			↿			
				0.08547	↿⇂	↿				↿		
				0.05775	↿⇂	↿		↿				
5	0.01	0.014	27476.2									
				0.51536	↿⇂	↿		↿				
				0.15695	↿⇂	↿			↿			
				0.08849	↿⇂		↿		↿			
				0.05294	↿⇂		↿			↿		

Table S30 | Singlet-state ZFS contributions and configurations as determined by CASSCF (4e, 8o) with NEVPT2 for **3**.

Root	ZFS, D	ZFS, E	NEVPT2 (cm^{-1})	Weight	Configuration							
					**do**	**d1**	**d2**	**d3**	**d4**	**d5**	**v1**	**v2**
0	0.004	−0.001	11809.9									
				0.49849	↿⇂	↿⇂						
				0.44689	↿⇂		↿⇂					
1	−0.001	0.001	12111.1									
				0.94705	↿⇂	⇂	↿					
2	3.579	−0.007	20368.5									
				0.4653	↿⇂		↿⇂					
				0.41845	↿⇂	↿⇂						
3	−0.891	−0.083	29252									
				0.35286	↿⇂		⇂	↿				
				0.24511	↿⇂	⇂		↿				

				0.18186	↿⇂	⇂				↿		
				0.08147	↿⇂	⇂			↿			
				0.06875	↿⇂		⇂		↿			
4	−0.915	0.077	29561.9									
				0.34751	↿⇂		⇂		↿			
				0.23656	↿⇂	⇂			↿			
				0.20426	↿⇂	⇂				↿		
				0.07548	↿⇂		⇂			↿		
5	0.032	−0.02	33145.8									
				0.3681	↿⇂		⇂	↿				
				0.24231	↿⇂	⇂		↿				
				0.16312	↿⇂	⇂				↿		
				0.08513	↿⇂		⇂		↿			
6	−0.046	0.034	33392.8									
				0.25556	↿⇂		⇂		↿			
				0.24553	↿⇂	⇂			↿			
				0.18576	↿⇂	⇂				↿		
				0.16601	↿⇂		⇂			↿		
				0.05213	↿⇂		⇂	↿				
7	0.04	−0.018	31356.7									
				0.35534	↿⇂	⇂		↿				
				0.27541	↿⇂	⇂			↿			
				0.1767	↿⇂	⇂				↿		
				0.1148	⇂	↿	↿⇂					
8	0.031	0.01	31853.7									
				0.5691	↿⇂		⇂			↿		
				0.1423	⇂	↿⇂	↿					
				0.1148	↿⇂		⇂		↿			
				0.09039	↿⇂		⇂	↿				
9	0.162	−0.047	39484									
				0.5594	⇂	↿	↿⇂					
				0.06126	⇂	⇂	↿	↿				
10	−0.057	0.059	39809.2									
				0.60837	⇂	↿⇂	↿					
				0.08741	↿⇂		⇂			↿		
11	0.014	−0.001	48633.1									
				0.29386	↿⇂			⇂		↿		
				0.19222	↿⇂			⇂	↿			
				0.1271	⇂	⇂	↿		↿			
				0.10417	↿⇂				↿⇂			
				0.06779	↿⇂					↿⇂		
				0.06303	⇂	↿	↿⇂					

Table S31 | Triplet-state ZFS contributions and configurations as determined by CASSCF (4e, 8o) with NEVPT2 for **4**.

Root	ZFS, D	ZFS, E	NEVPT2 (cm^{-1})	Weight	Configuration							
					**do**	**d1**	**d2**	**d3**	**d4**	**d5**	**v1**	**v2**
ground			0	0.94486	↑↓	↑	↑					
1	1.497	1.155	17748.3									
				0.32965	↑↓		↑		↑			
				0.21825	↑↓	↑			↑			
				0.21355	↑↓		↑			↑		
				0.11866	↑↓	↑				↑		
				0.05014	↑↓	↑		↑				
2	1.635	−1.176	18650.8									
				0.3556	↑↓	↑				↑		
				0.23348	↑↓		↑			↑		
				0.17519	↑↓	↑		↑				
				0.09725	↑↓		↑		↑			
3	−0.256	−0.048	21606.7									
				0.62991	↑↓	↑		↑				
				0.29388	↑↓		↑			↑		
4	0.218	0.143	21943.3									
				0.52065	↑↓		↑		↑			
				0.26869	↑↓	↑				↑		
				0.0851	↑↓	↑			↑			
5	0.019	−0.021	25701.2									
				0.55188	↑↓	↑			↑			
				0.15067	↑↓		↑			↑		
				0.11098	↑↓	↑		↑				
				0.07	↑↓	↑				↑		

Table S32 | Singlet-state ZFS contributions and configurations as determined by CASSCF (4e, 8o) with NEVPT2 for **4**.

Root	ZFS, D	ZFS, E	NEVPT2 (cm^{-1})	Weight	Configuration							
					**do**	**d1**	**d2**	**d3**	**d4**	**d5**	**v1**	**v2**
0	0.014	0.001	11686.5									
				0.53496	↑↓	↑↓						
				0.40852	↑↓		↑↓					
1	0.004	−0.003	11998.9									
				0.94374	↑↓	↓	↑					
2	2.442	0.001	20299									
				0.4992	↑↓		↑↓					
				0.37663	↑↓	↑↓						
3	−1.107	−0.989	28059.2									
				0.42614	↑↓		↓		↑			
				0.18337	↑↓	↓			↑			
				0.17416	↑↓	↓				↑		
				0.08058	↑↓	↓		↑				
				0.05807	↑↓		↓			↑		
4	−1.148	1.035	28454.5									

				0.31124	↿⇂	⇂		↿				
				0.22162	↿⇂	⇂				↿		
				0.20227	↿⇂		⇂			↿		
				0.10159	↿⇂	⇂			↿			
				0.08758	↿⇂		⇂		↿			
5	−0.014	−0.019	31044.6									
				0.32967	↿⇂	⇂			↿			
				0.25716	↿⇂		⇂		↿			
				0.18556	↿⇂	⇂				↿		
				0.12964	↿⇂		⇂			↿		
6	0.053	−0.034	32249.7									
				0.4531	↿⇂		⇂			↿		
				0.19411	↿⇂	⇂				↿		
				0.10103	↿⇂		⇂	↿				
				0.07454	↿⇂	⇂		↿				
				0.06261	↿⇂		⇂		↿			
7	0.077	0.006	30508.6									
				0.41883	↿⇂	⇂		↿				
				0.24398	↿⇂	⇂			↿			
				0.11376	⇂	↿	↿⇂					
				0.10703	↿⇂	⇂				↿		
8	−0.002	0.002	31281.4									
				0.63469	↿⇂		⇂	↿				
				0.15469	⇂	↿⇂	↿					
				0.0838	↿⇂		⇂		↿			
9	0.231	0.007	38910.5									
				0.54053	⇂	↿	↿⇂					
				0.06994	⇂	⇂	↿			↿		
				0.05976	↿⇂	⇂		↿				
10	−0.046	0.005	39013.5									
				0.57124	⇂	↿⇂	↿					
				0.12018	↿⇂		⇂	↿				
11	0.002	−0.003	46548.1									
				0.31691	↿⇂			⇂	↿			
				0.26511	↿⇂				↿⇂			
				0.18837	↿⇂					↿⇂		
				0.05664	⇂	↿	↿⇂					

Table S33 | Significant TD-DFT natural transition orbital (NTO) contributions for major transitions calculated for the absorption spectrum of **1** (a – alpha, b – beta).

Transition 5: 462.4 nm, 116a → 117a, 86.5%

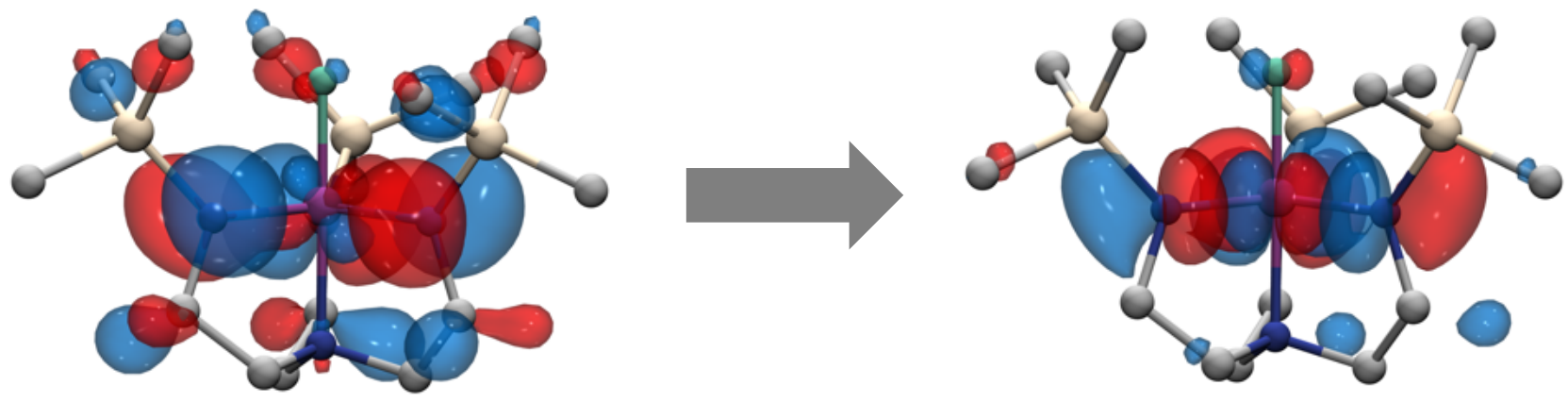

Transition 6: 460.3 nm, 116a → 117a, 87.5%

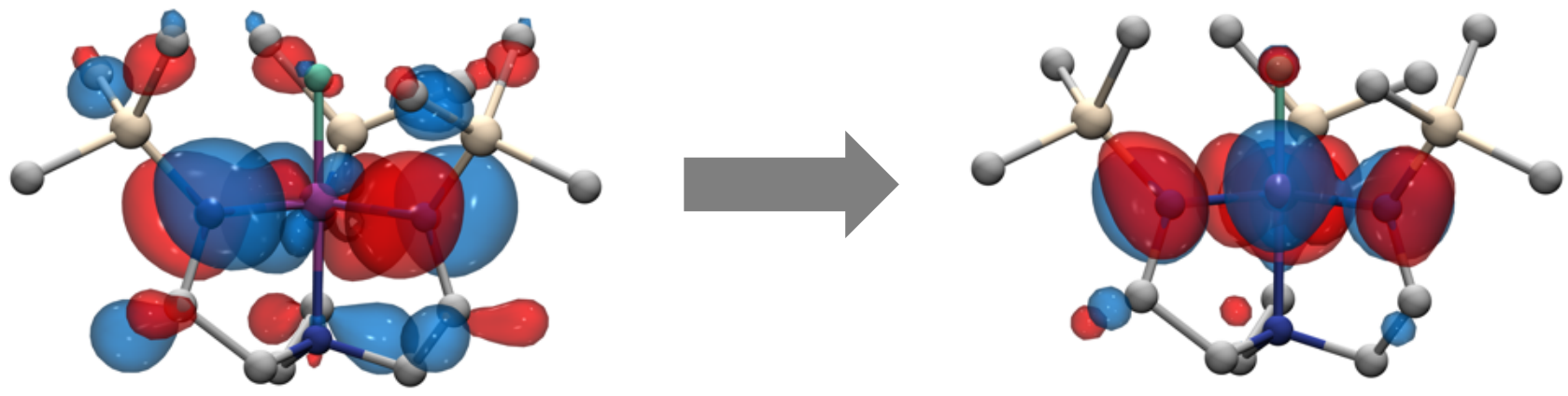

Transition 15: 302.7 nm, 114b → 115b, 80.8%

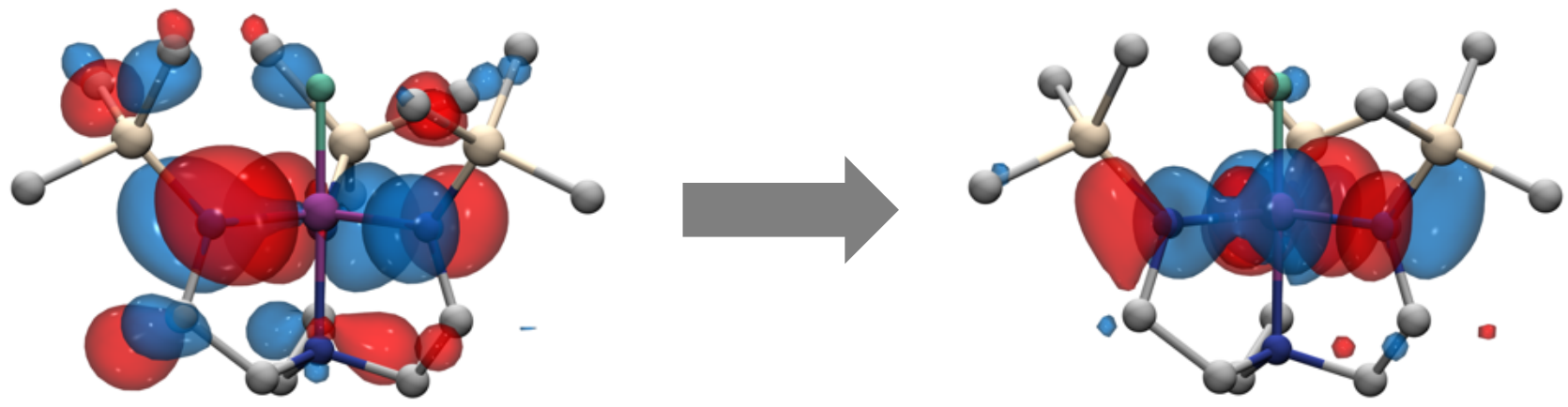

Transition 16: 301.6 nm, 114b → 115b, 77.8%

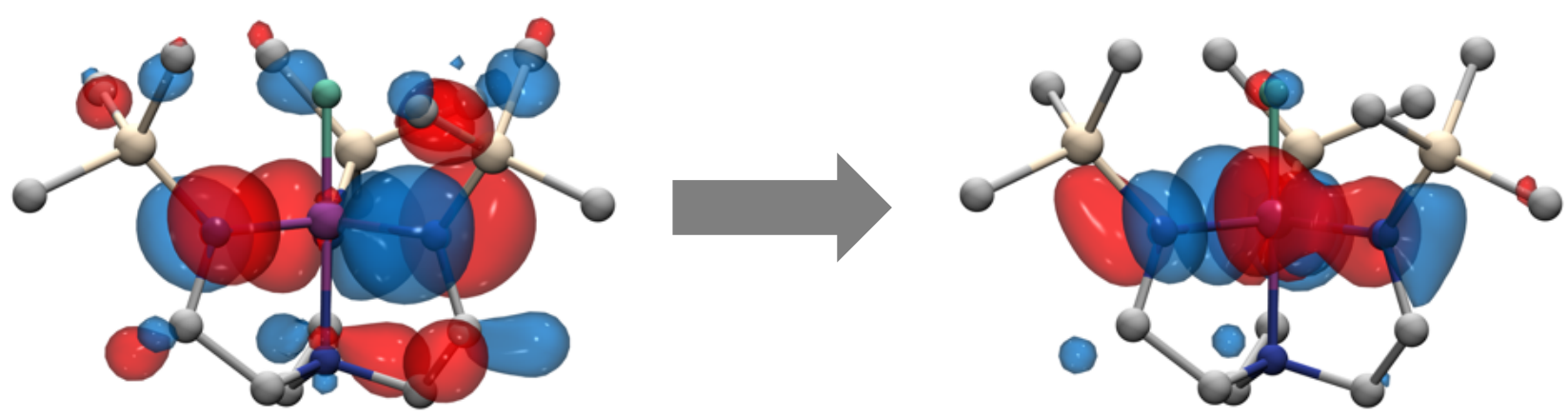

Table S34 | Significant TD-DFT natural transition orbital (NTO) contributions for major transitions calculated for absorption spectrum of **2** (a – alpha, b – beta).

Transition 5: 465.4 nm, 120a → 121a, 78.8%

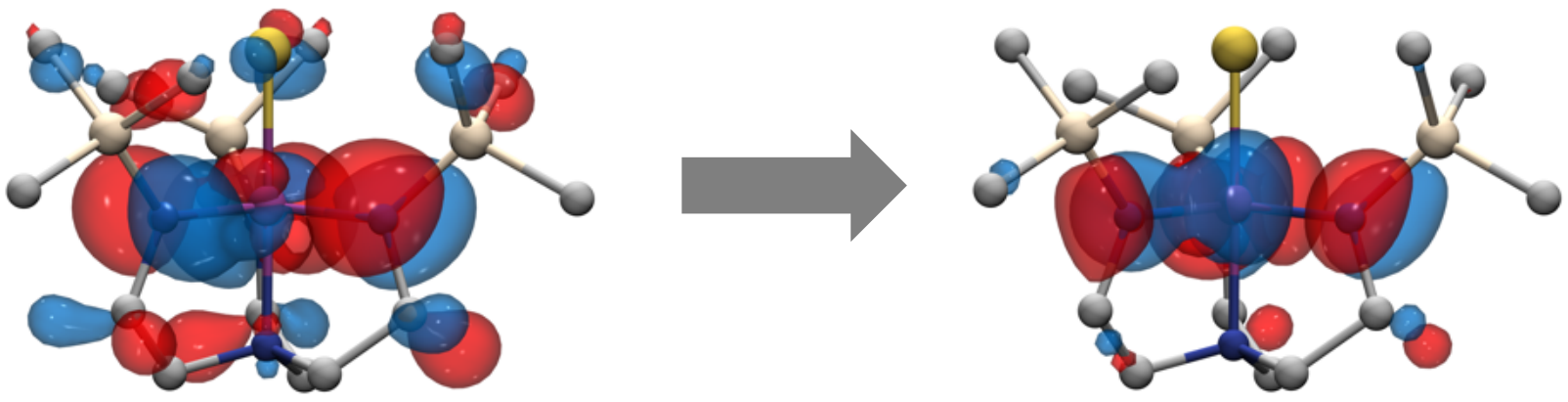

Transition 6: 460.0 nm, 120a → 121a, 81.1%

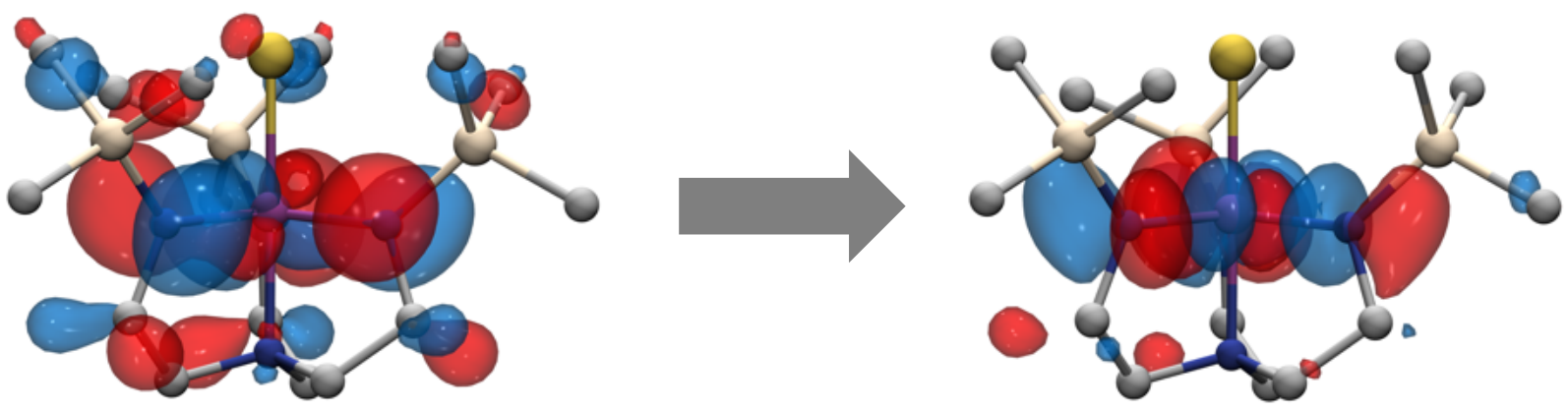

Transition 20: 302.8 nm, 118b → 119b, 76.5%

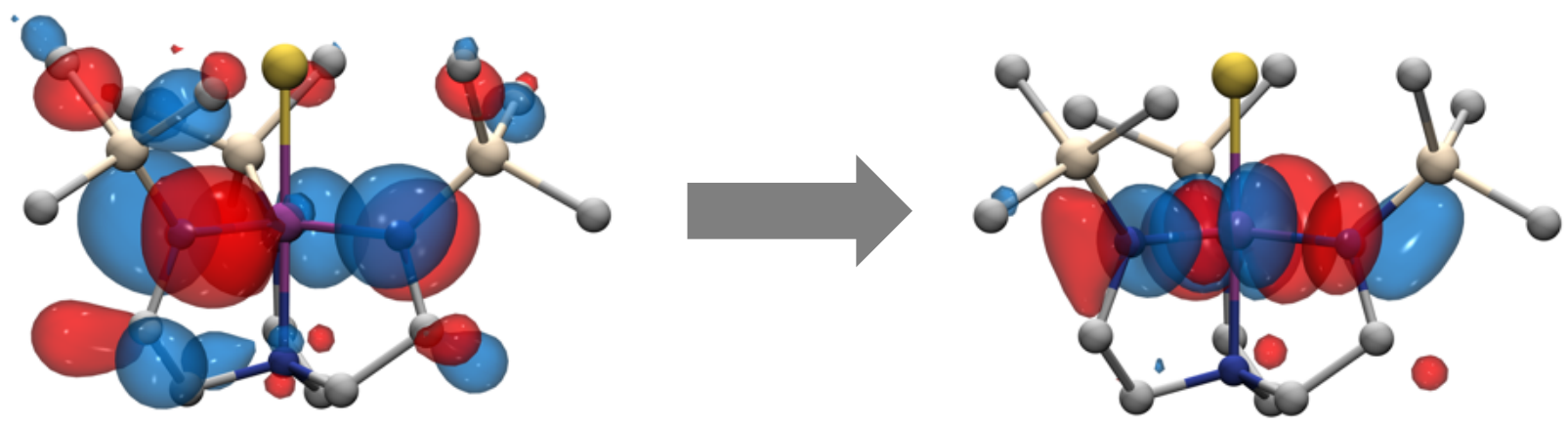

Transition 21: 301.2 nm, 118b → 119b, 76.8%

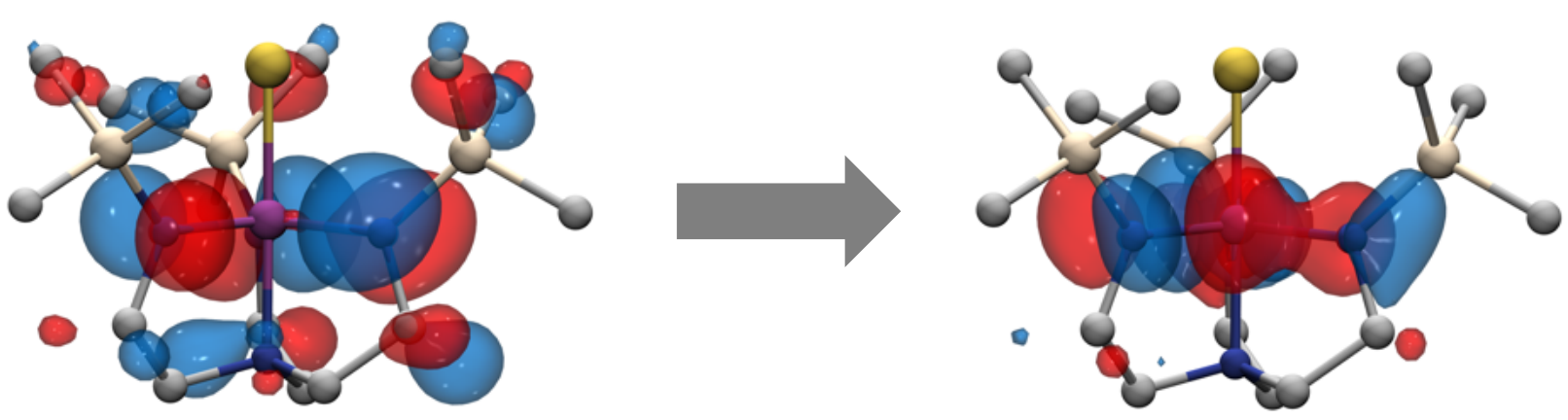

Table S35 | Significant TD-DFT natural transition orbital (NTO) contributions for major transitions calculated for absorption spectrum of **3** (a – alpha, b – beta).

Transition 5: 449.8 nm, 129a → 130a, 81.3%

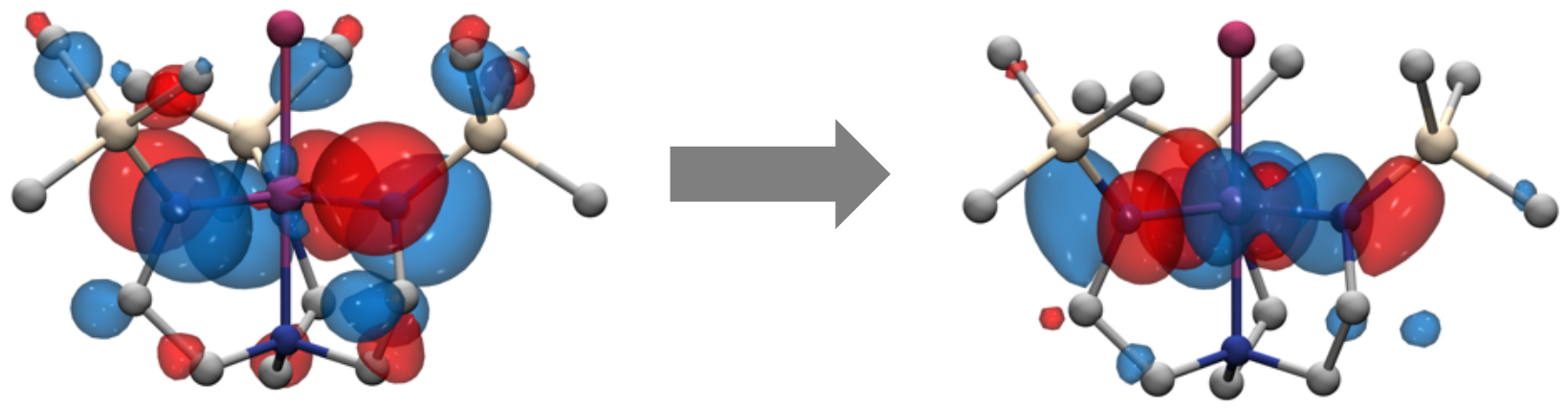

Transition 6: 446.0 nm, 129a → 130a, 80.8%

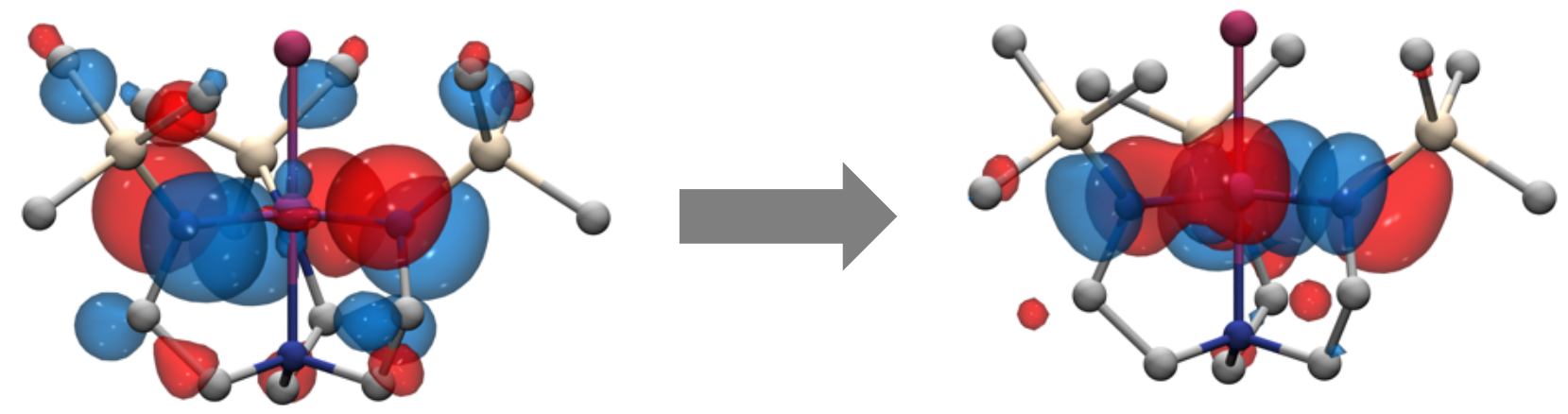

Transition 7: 426.7 nm, 129a → 130a, 74.8%

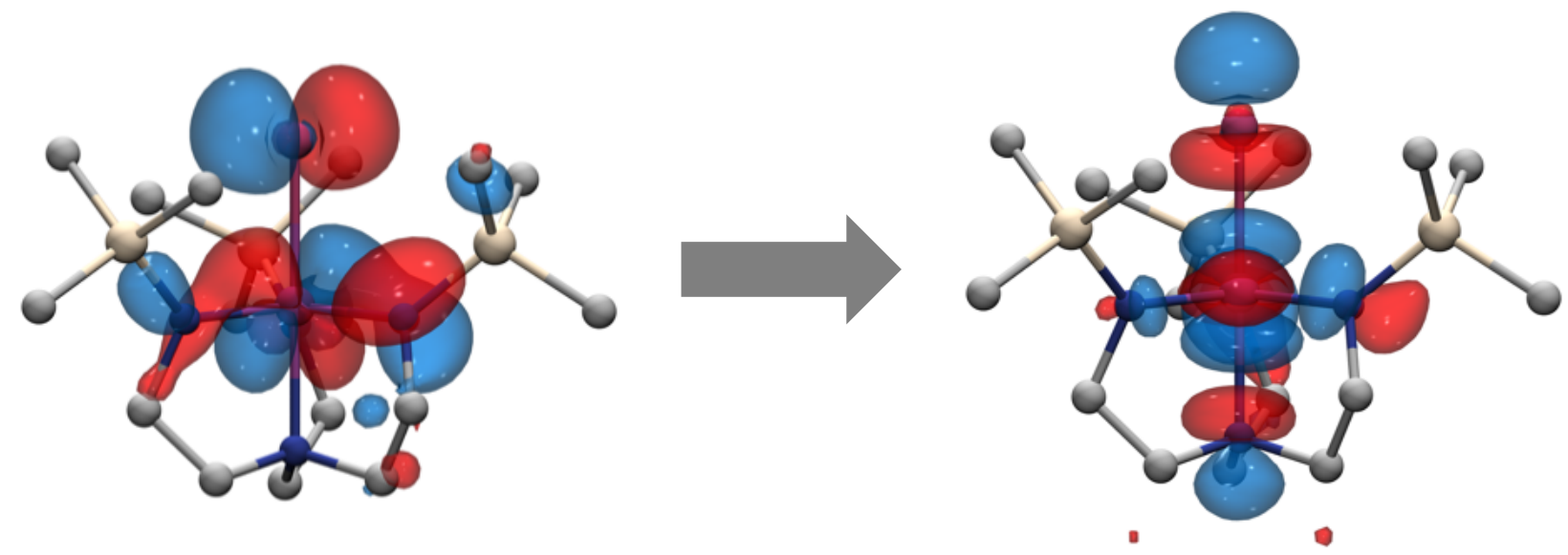

Transition 8: 423.5 nm, 129a → 130a, 78.3%

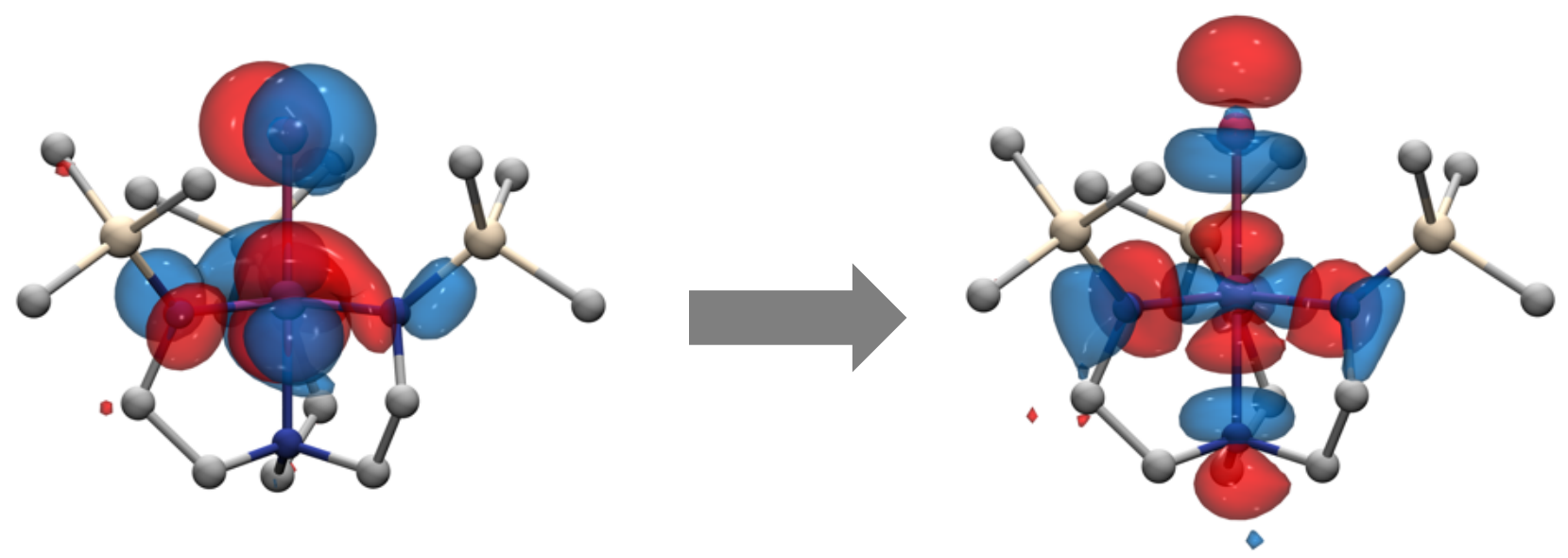

Transition 20: 302.6 nm, 127b → 128b, 59.7%

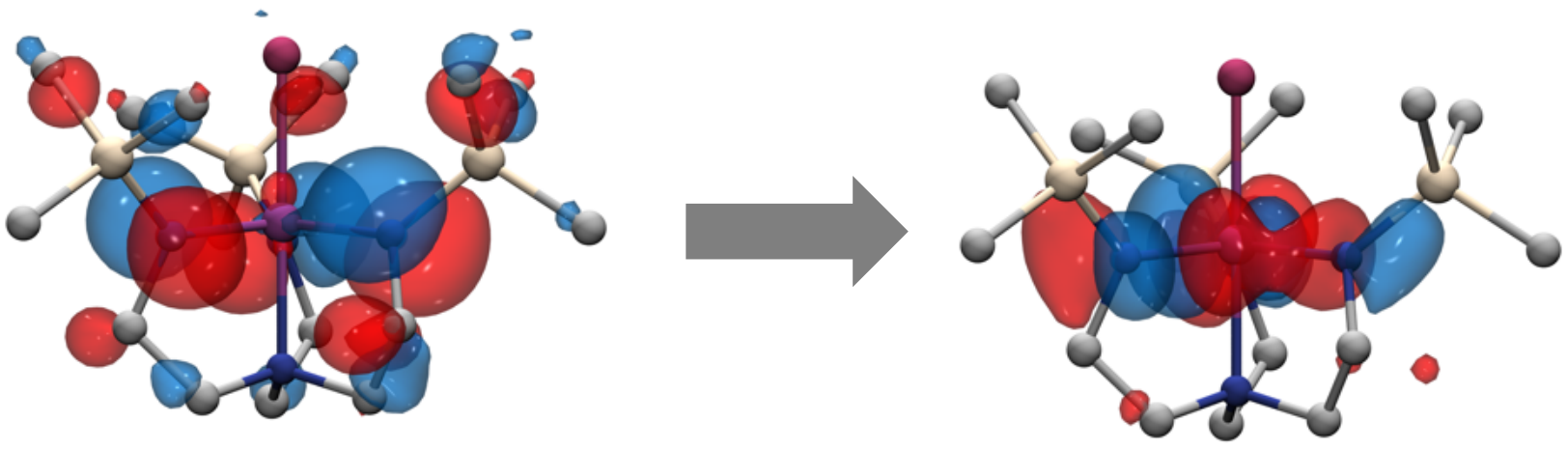

Transition 22: 301.0 nm, 127b → 128b, 59.4%

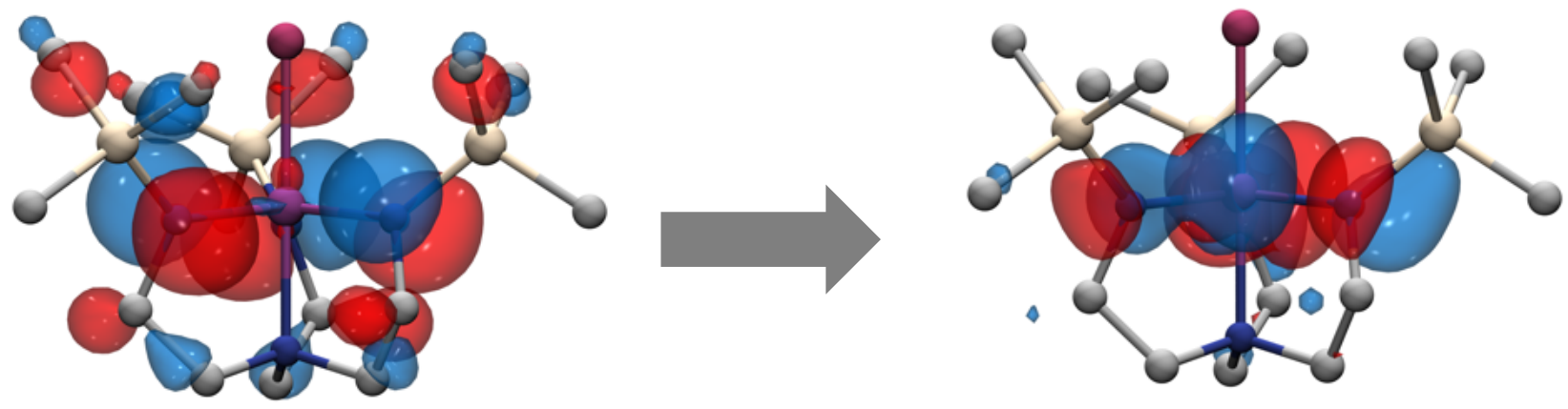

Table S36 | Significant TD-DFT natural transition orbital (NTO) contributions for major transitions calculated for the absorption spectrum of **4** (a – alpha, b – beta).

Transition 7: 433.0 nm, 124a → 125a, 86.3%

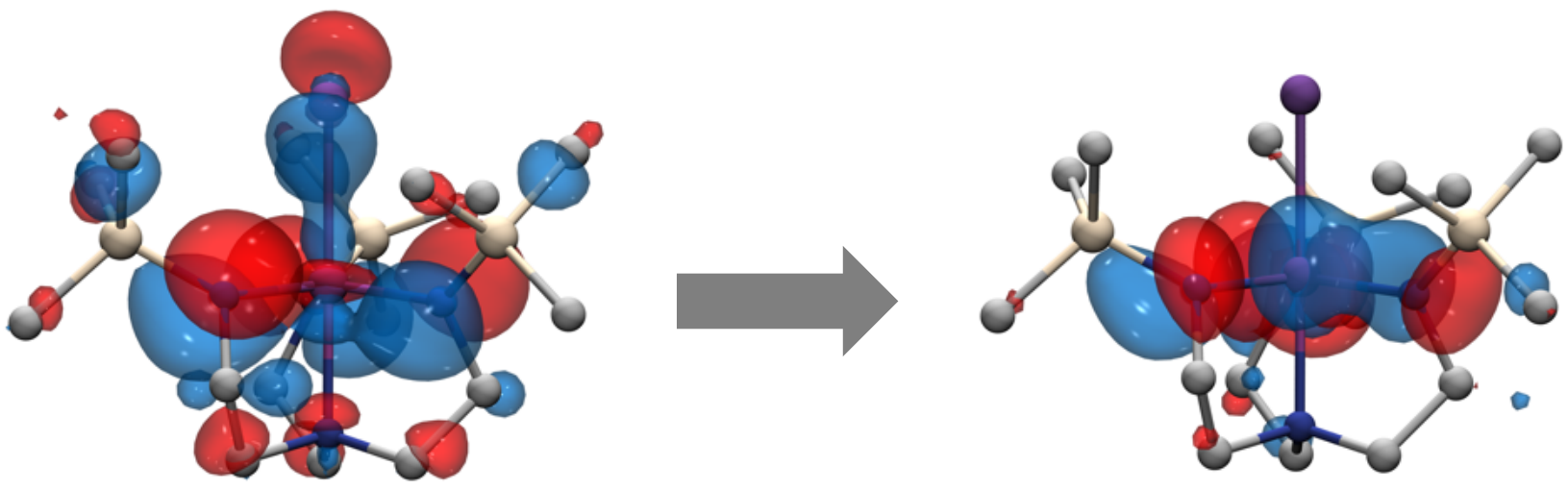

Transition 8: 427.3 nm, 124a → 125a, 89.1%

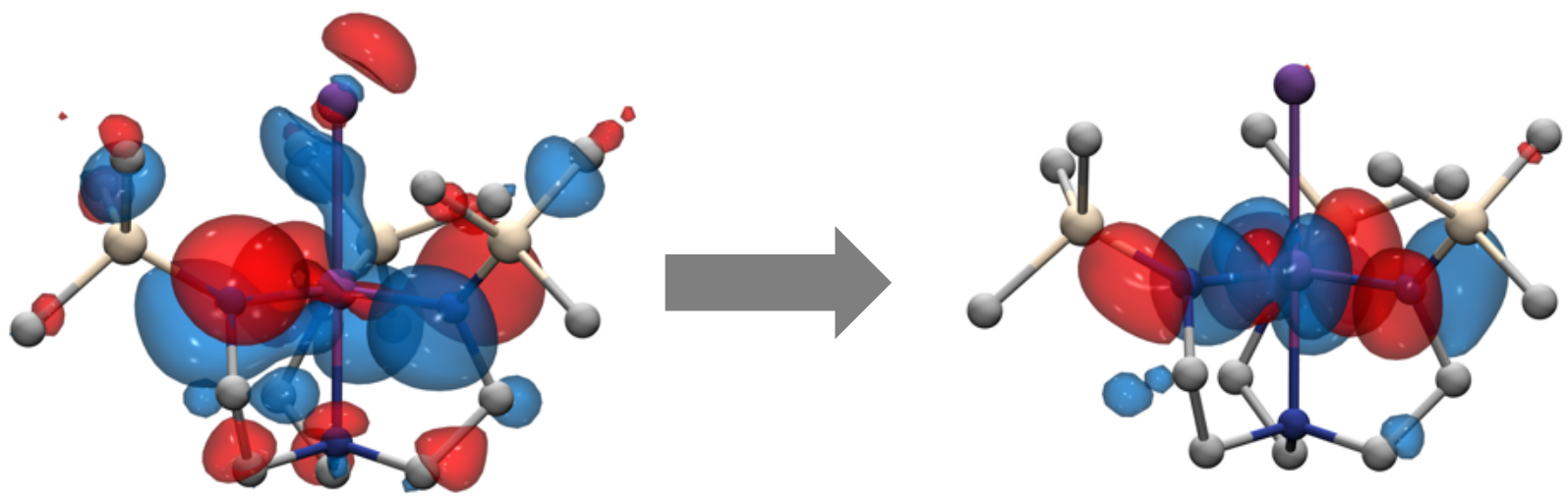

Transition 19: 351.0 nm

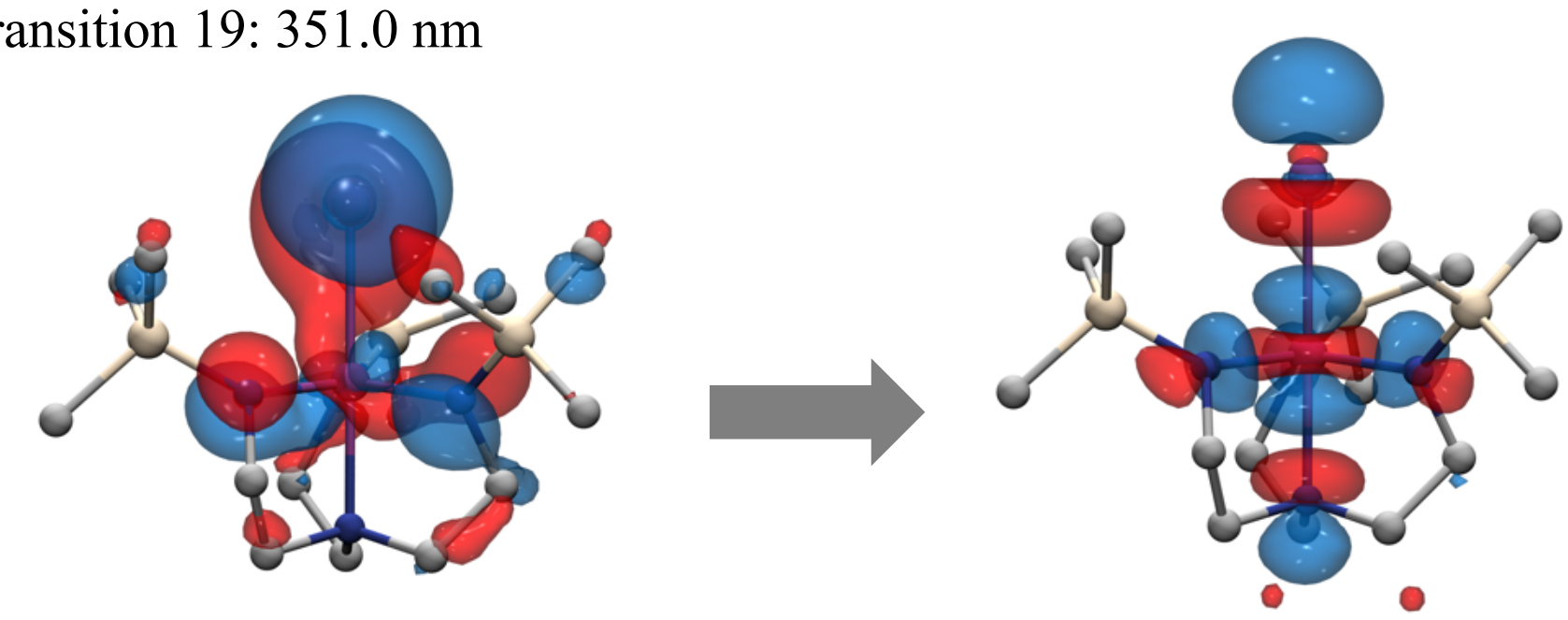

35.0% 124a → 125a

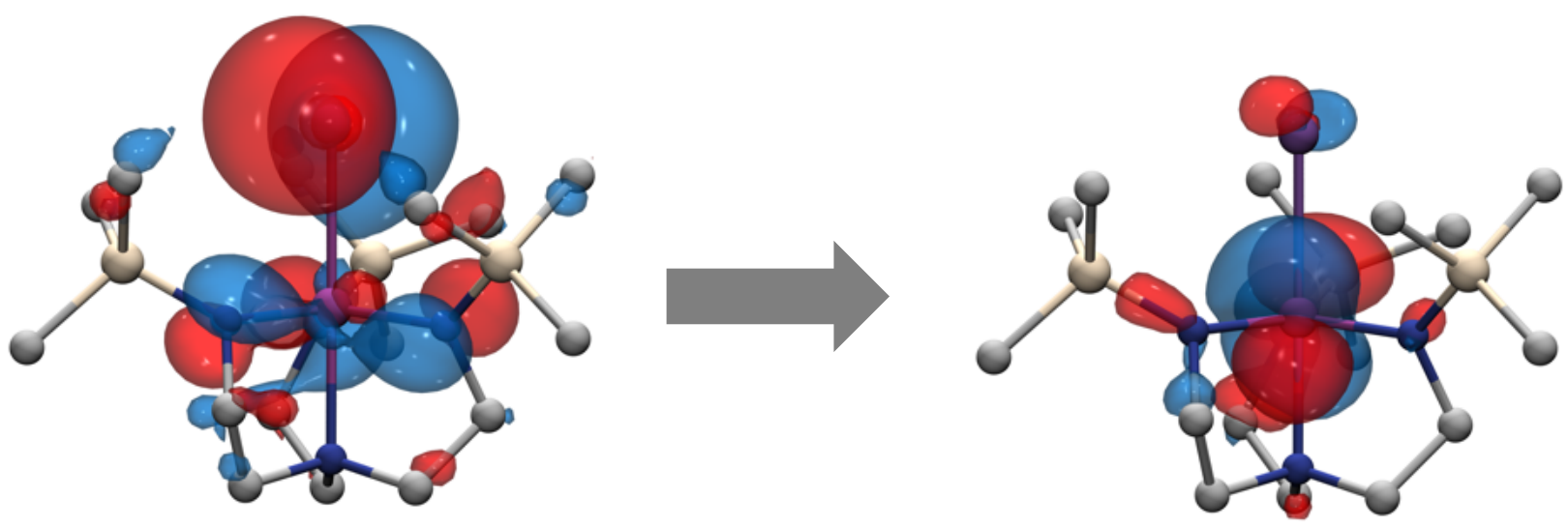

36.3% 122a → 125a

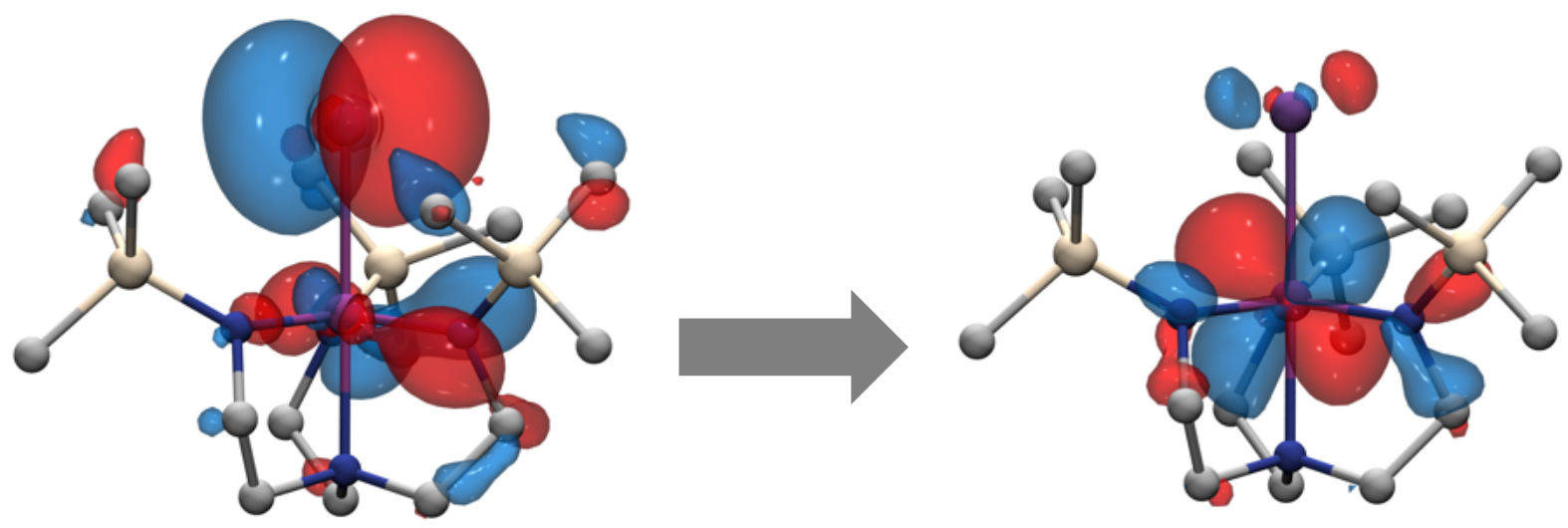

24.1% 121b → 124b

Table S37 | Spin Hamiltonian parameters derived from fits of variable-frequency HFHF cw-EPR spectra (Figures S34–S53).

**Complex**	g_{xy}	g_z	***D*** **(GHz)**	***D*** **(cm^{-1})**	**| *E* | (cm^{-1})**	**lwpp (mT)**
**1**	1.972(2)	1.990(1)	+155.7(1)	+5.194(4)	0	40(30)
**2**	1.956(2)	1.967(3)	+166(3)	+5.52(1)	0	60(30)
**3**	1.990(2)	1.978(2)	+204(3)	+6.81(1)	0	120(55)
**4**	1.980(2)	1.956(4)	+220(3)	+7.34(1)	0	300(50)
**4′ (isolated)**	2.001(2)	1.997(2)	+215.0(1)	+7.173(3)	0.08(1)	75(10)
**4′ (conc.)**	2.004(3)	2.00(1)	+225(3)	+7.50(1)	0.08(1)	250(50)

Table S38 | Exchange coupling parameters extracted from forbidden ($\Delta m_s = 2$) half-field transitions of complexes **1**–**4** in Figure S54.

**Complex**	***J*** **(GHz)**	**lwpp (mT)**
**1**	2.4(3)	60(10)
**2**	2.3(3)	40(10)
**3**	2.0(3)	30(10)
**4**	3.7(3)	50(10)
**4′ (conc.)**	3.7(3)	250(50)

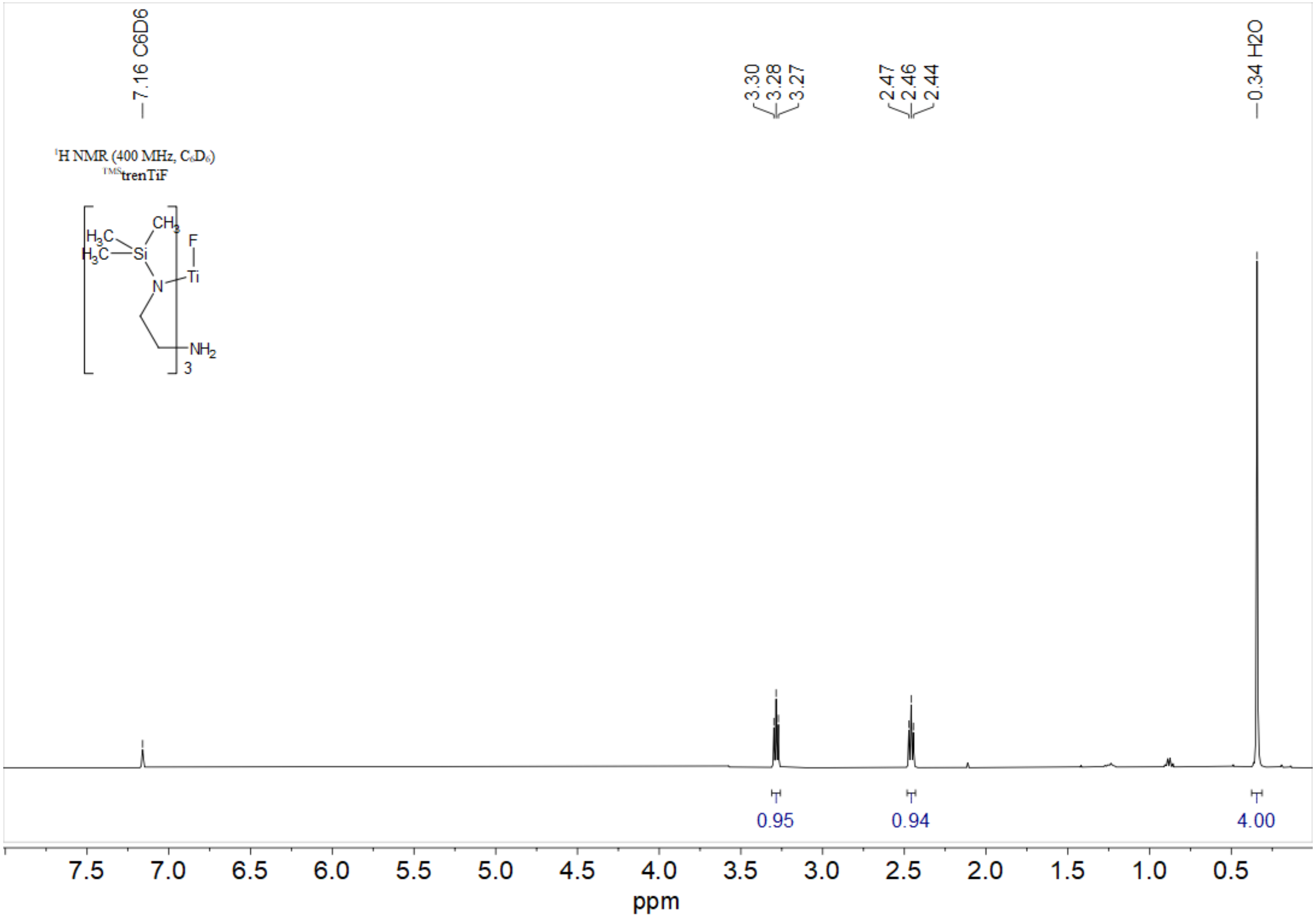

Figure S1 | ^{1}H NMR spectrum of **5** in C_6D_6.

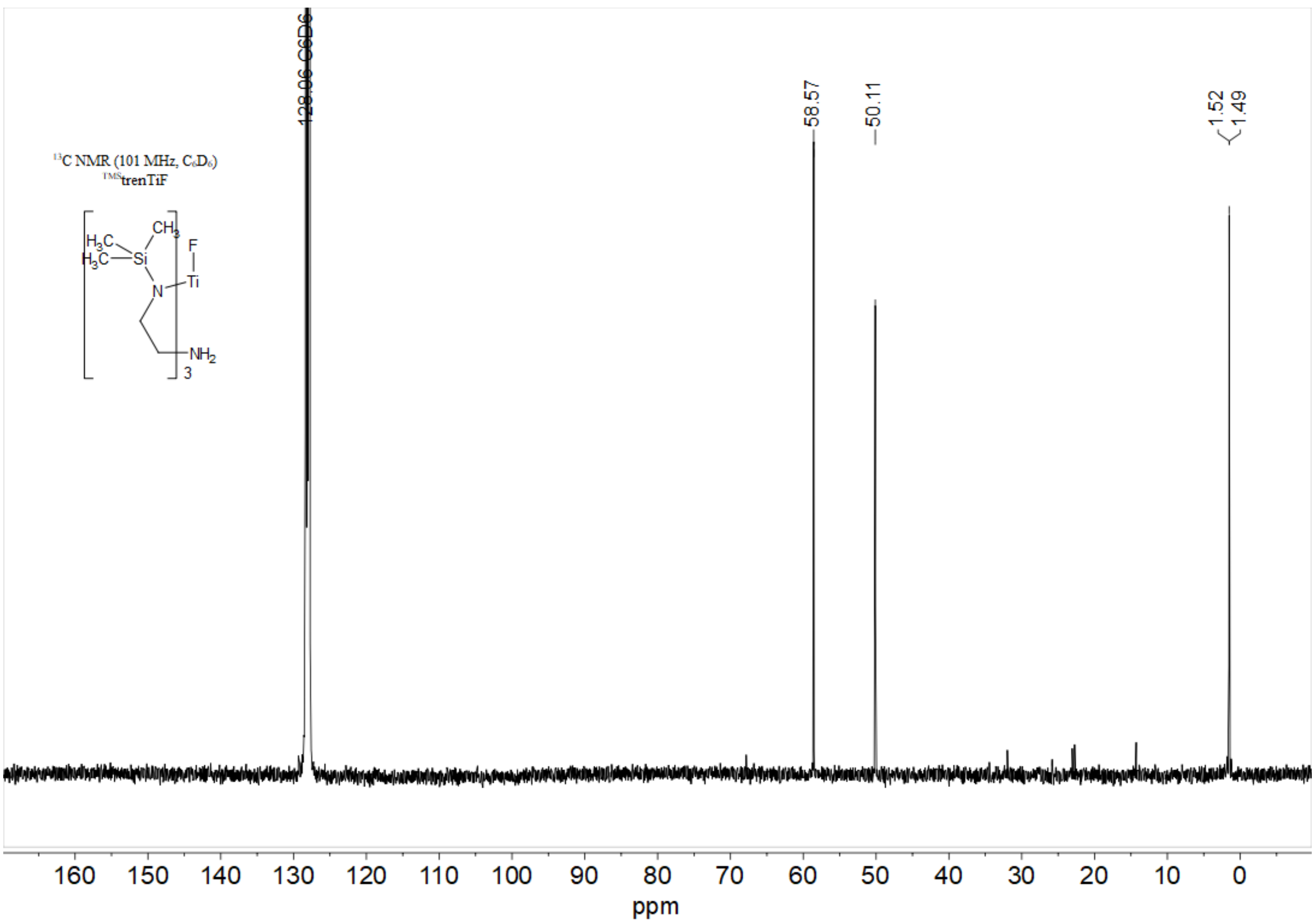

Figure S2 | ^{13}C NMR spectrum of **5** in C_6D_6.

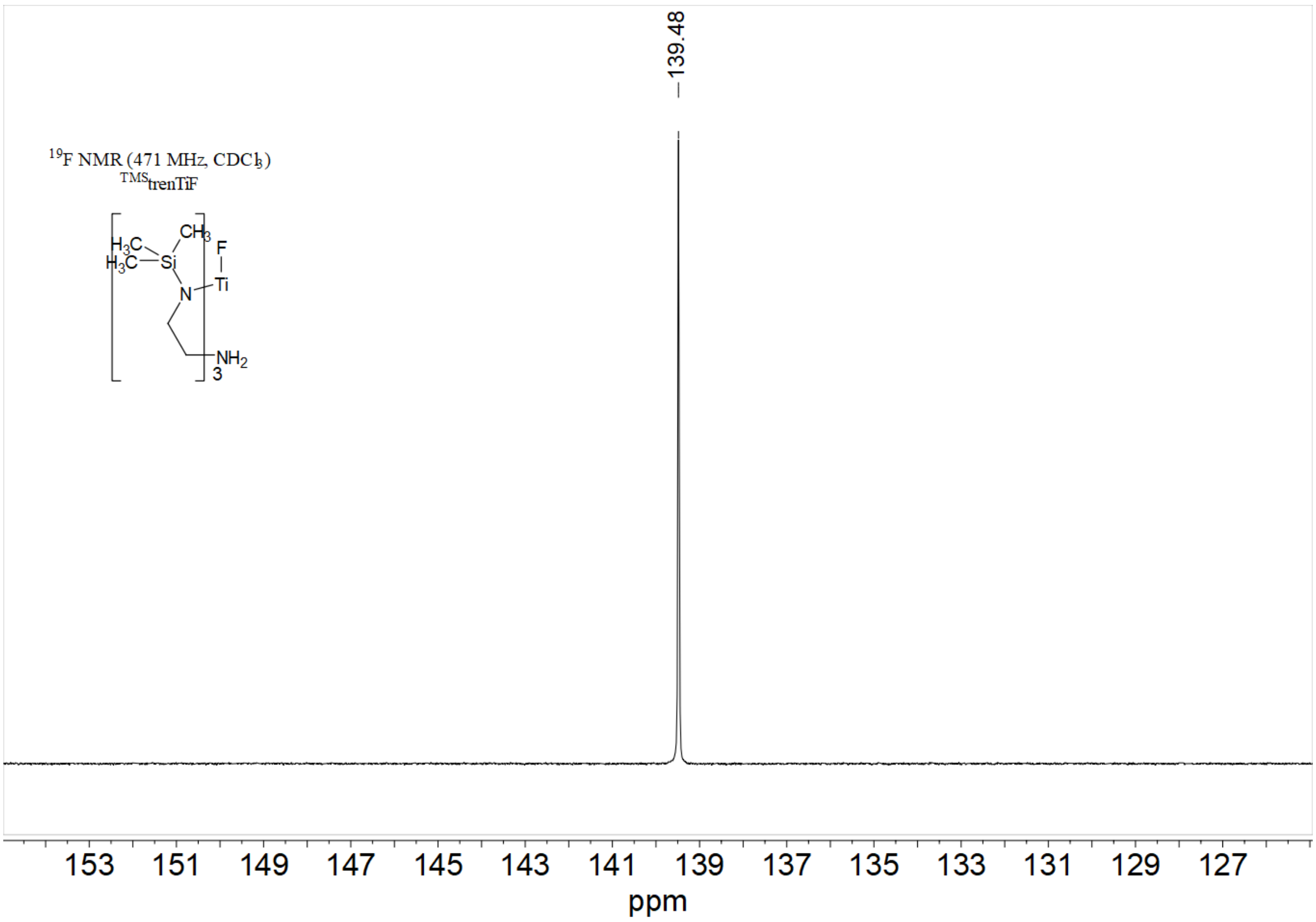

Figure S3 | ^{19}F NMR spectrum of **5** in C_6D_6.

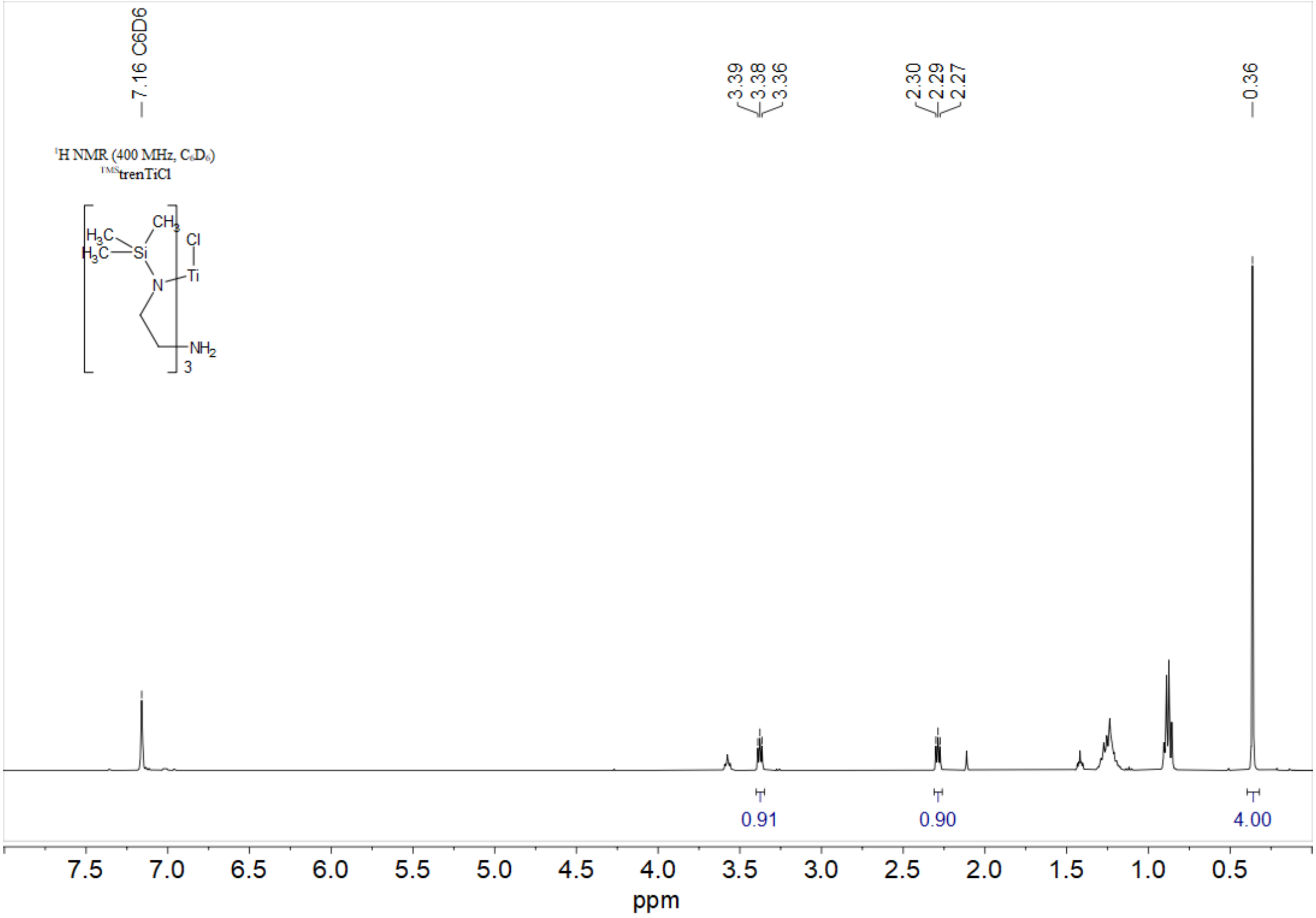

Figure S4 | ^{1}H NMR spectrum of **6** in C_6D_6

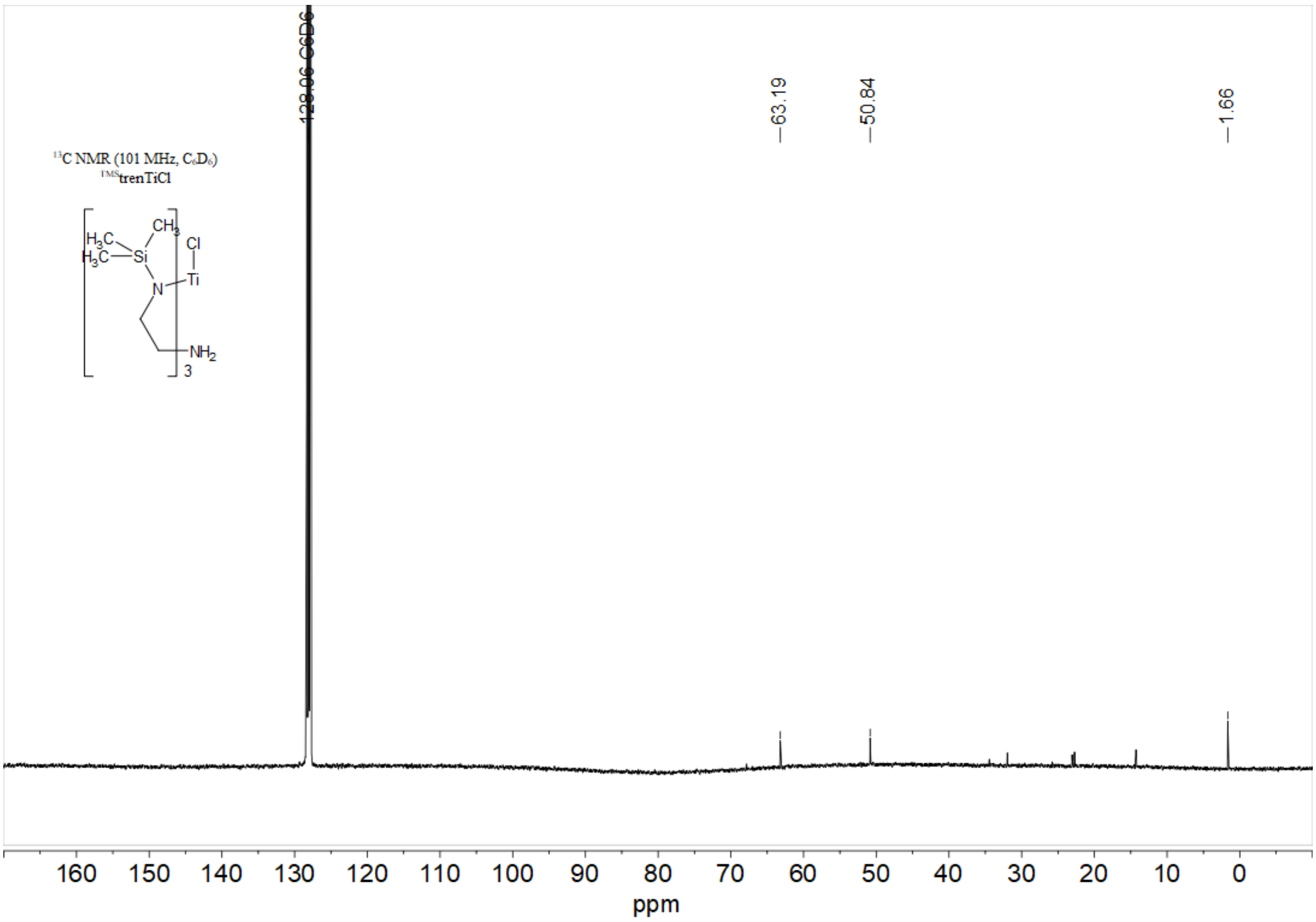

Figure S5 | ^{13}C NMR spectrum of **6** in C_6D_6.

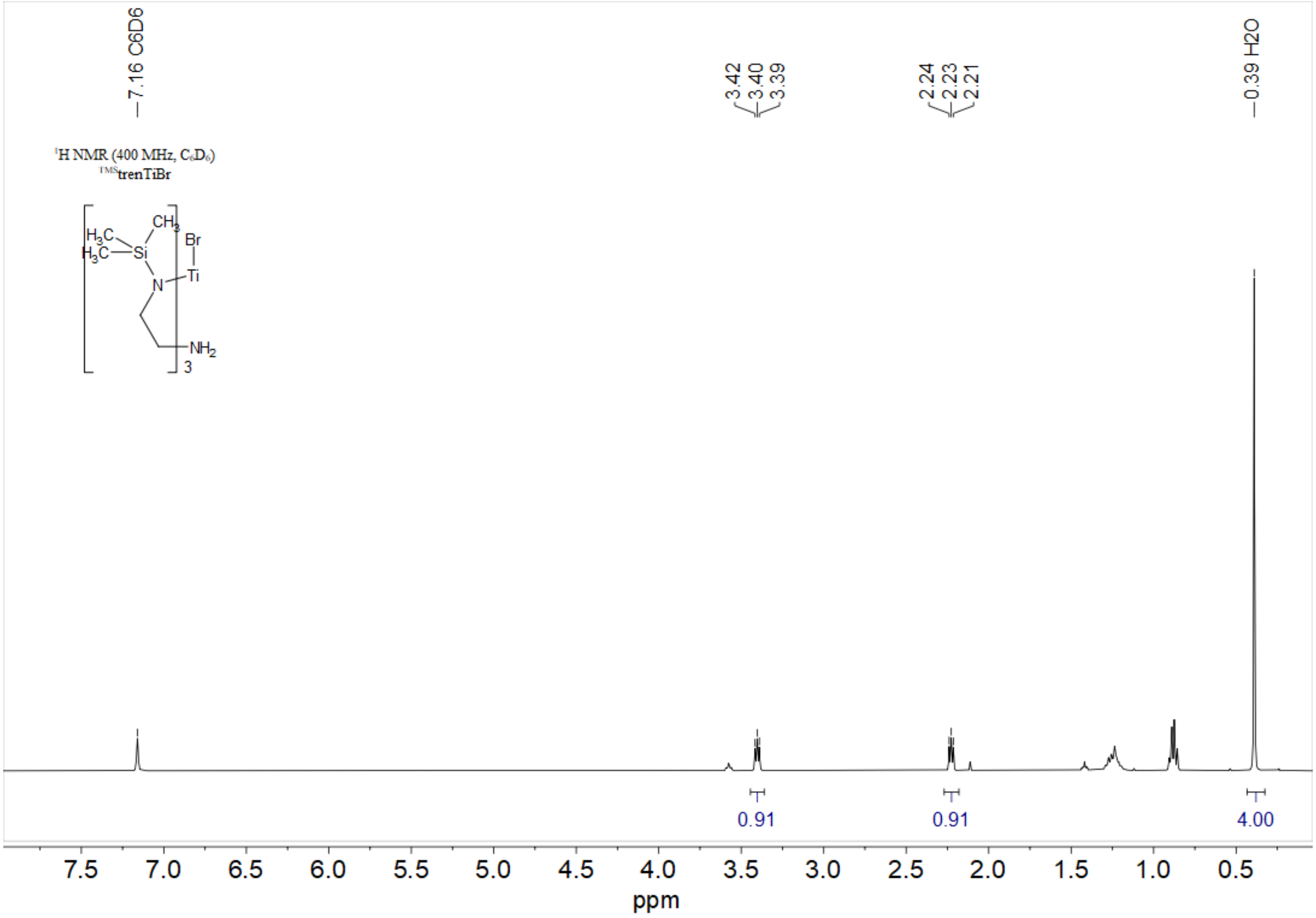

Figure S6 | ^{1}H NMR spectrum of **7** in C_6D_6.

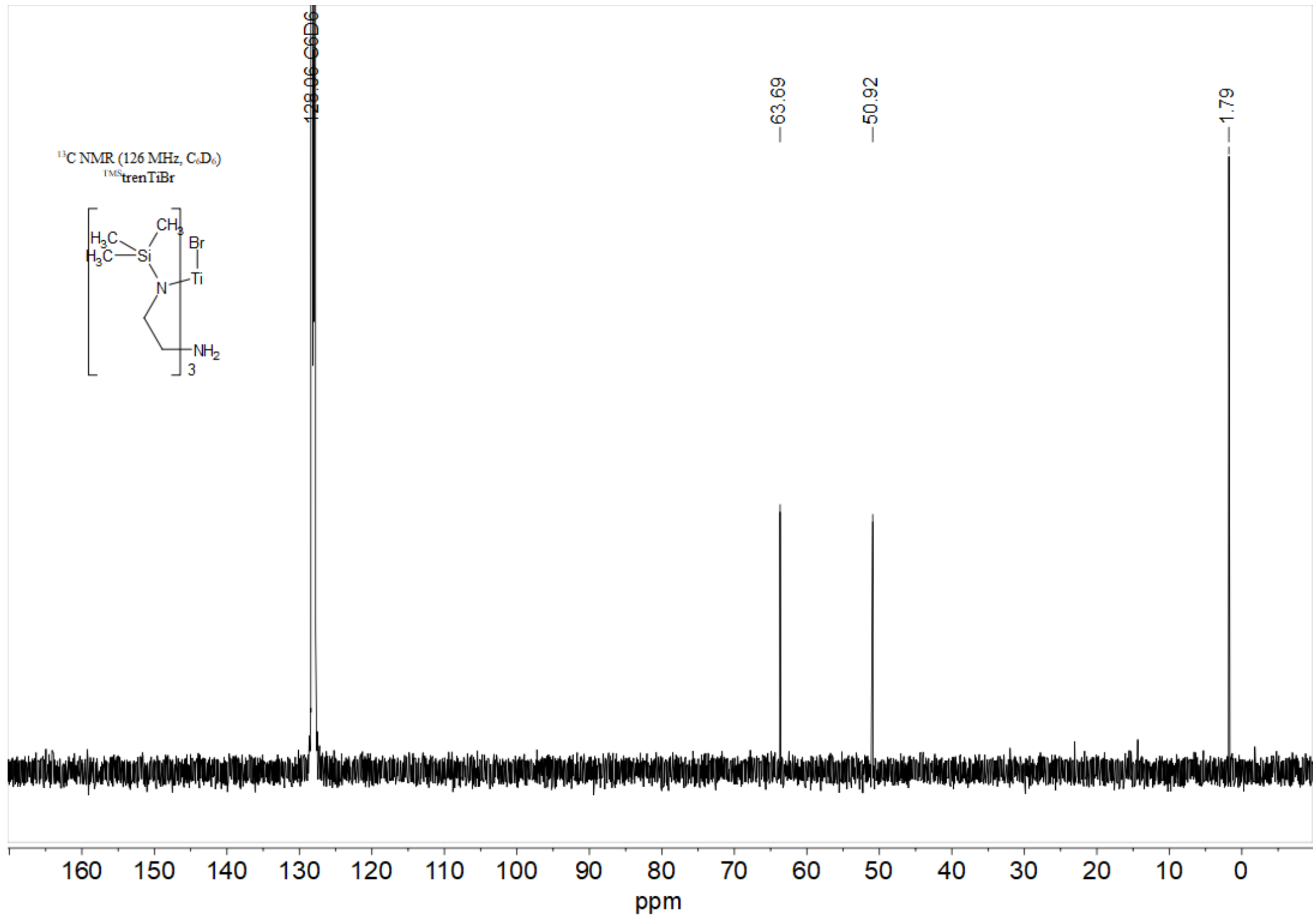

Figure S7 | ^{13}C NMR spectrum of **7** in C_6D_6.

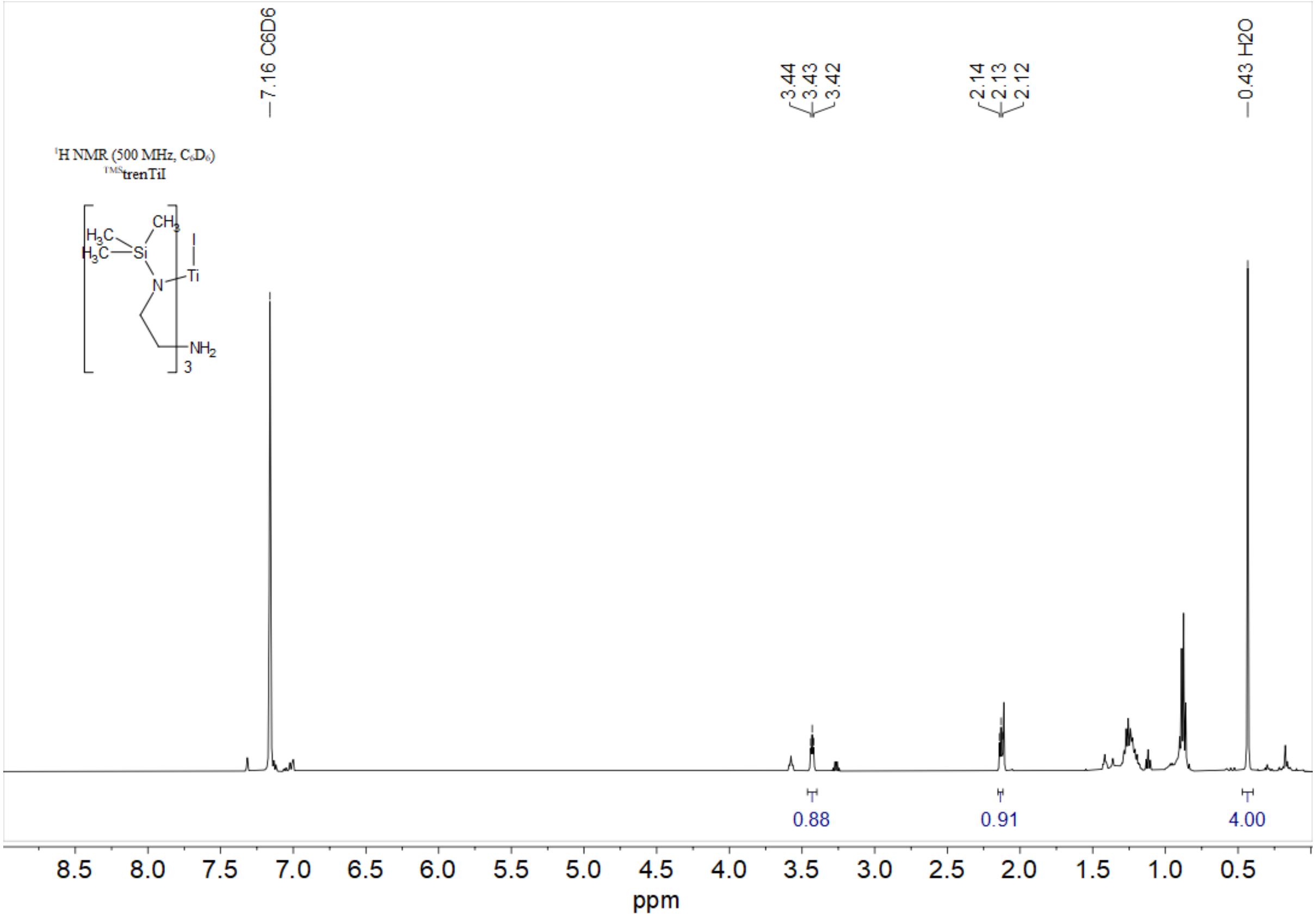

Figure S8 | ^{1}H NMR spectrum of **8** in C_6D_6.

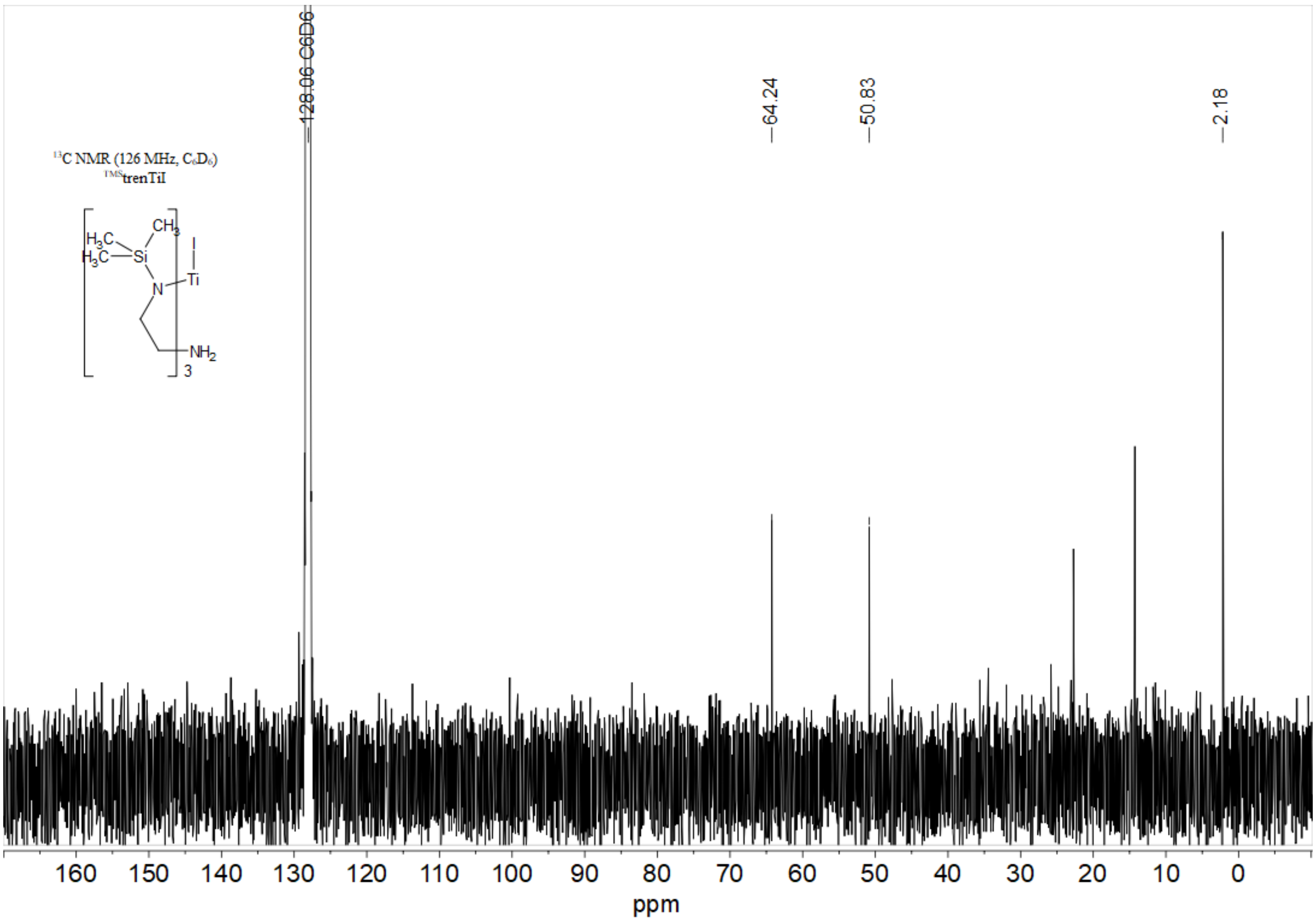

Figure S9 | ^{13}C NMR spectrum of **8** in C_6D_6.

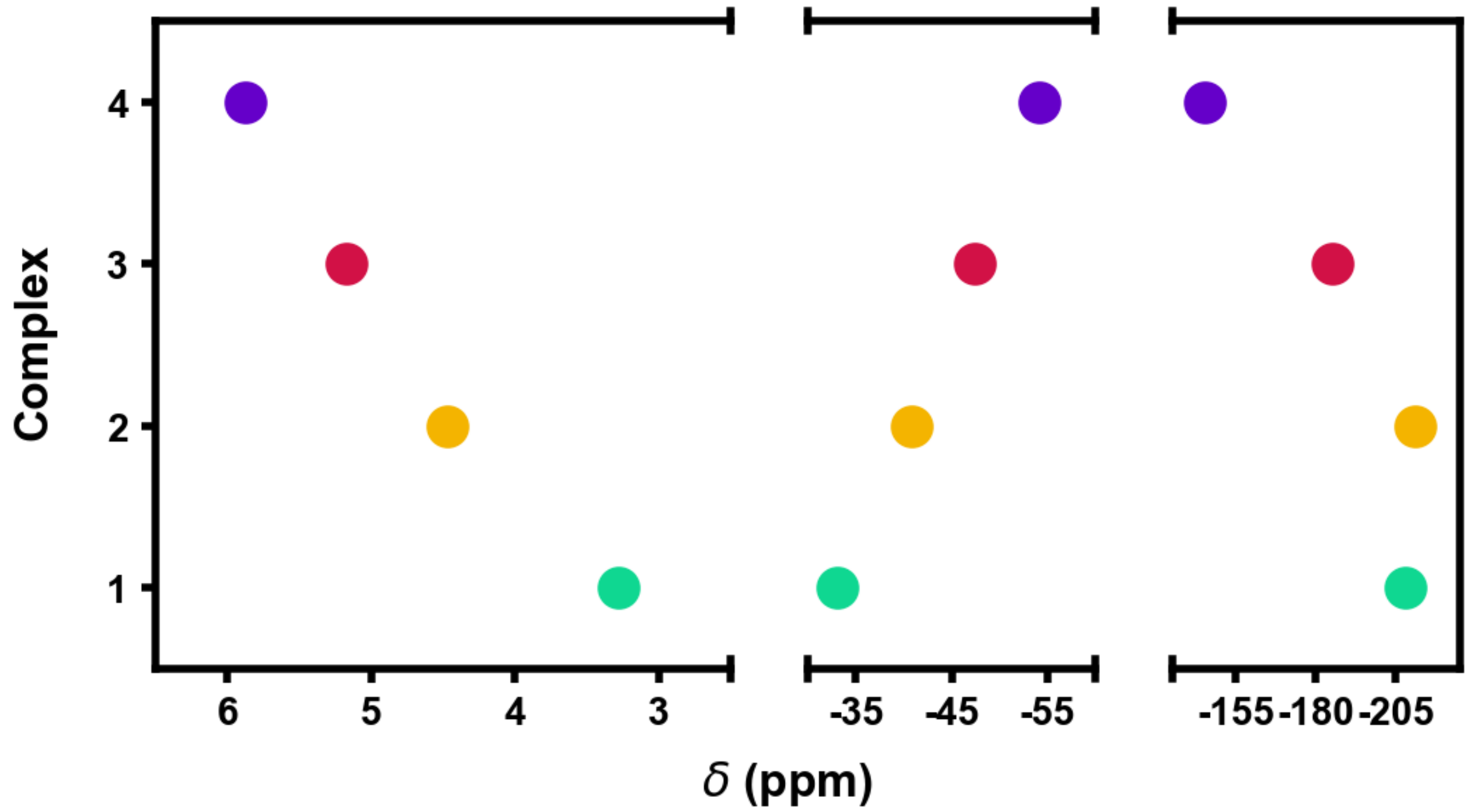

Figure S10 | Peak positions of ^{1}H NMR spectra of ^{TMS}trenCrF, ^{TMS}trenCrCl, ^{TMS}trenCrBr, ^{TMS}trenCrI complexes (green, yellow, red, purple, respectively) in C_6D_6 at 298 K.[6,7] The peak representing ^{1}H from the trimethylsilyl groups is on the left subplot, while the peaks representing ^{1}H from the two distinct methylene groups are on the right subplots.

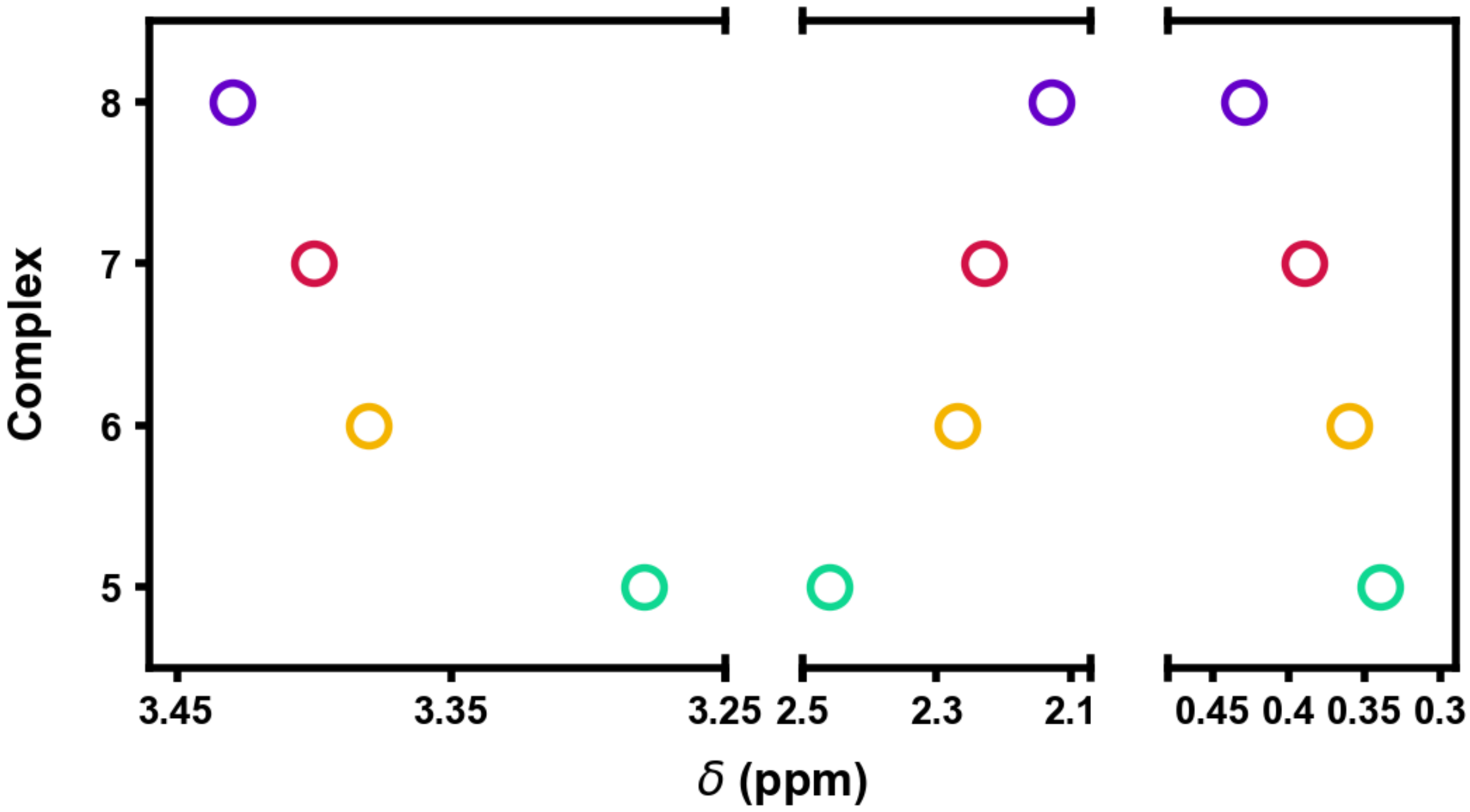

Figure S11 | Peak positions of ^{1}H NMR spectra of ^{TMS}trenTiF, ^{TMS}trenTiCl, ^{TMS}trenTiBr, ^{TMS}trenTiI complexes (green, yellow, red, purple, respectively) in C_6D_6 at 298 K from this work. The peak representing ^{1}H from the trimethylsilyl groups is on the right subplot, while the peaks representing ^{1}H from the two distinct methylene groups are on the left subplots.

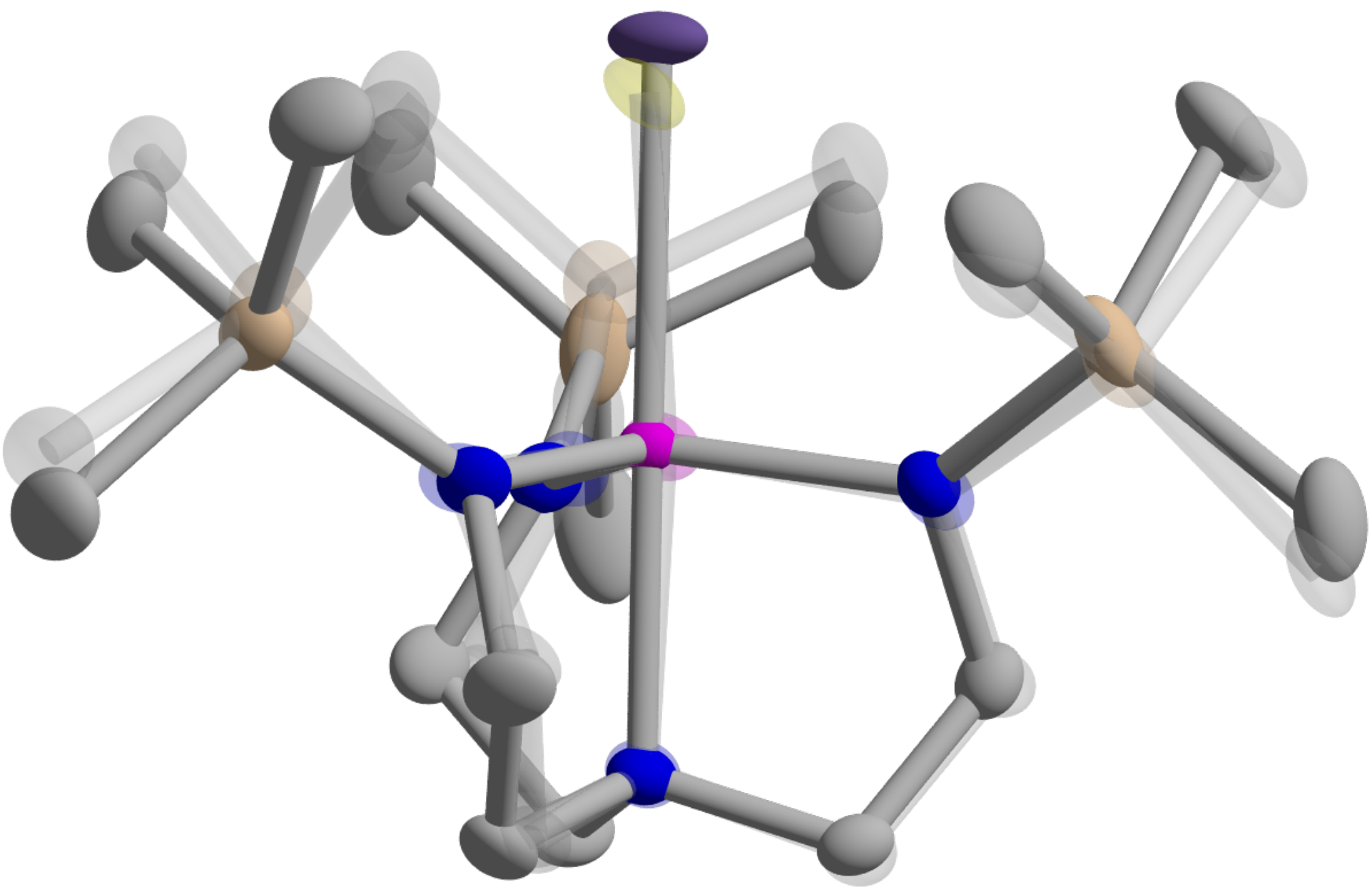

Figure S12 | Thermal ellipsoid plot of **4** crystal structure. Carbon, nitrogen, silicon, chlorine, iodine, and chromium atoms are colored gray, blue, tan, yellow, purple, and pink, respectively. Hydrogen atoms were omitted for clarity. The ^{TMS}trenCrCl fragment of 16% population is displayed with 80% transparency.

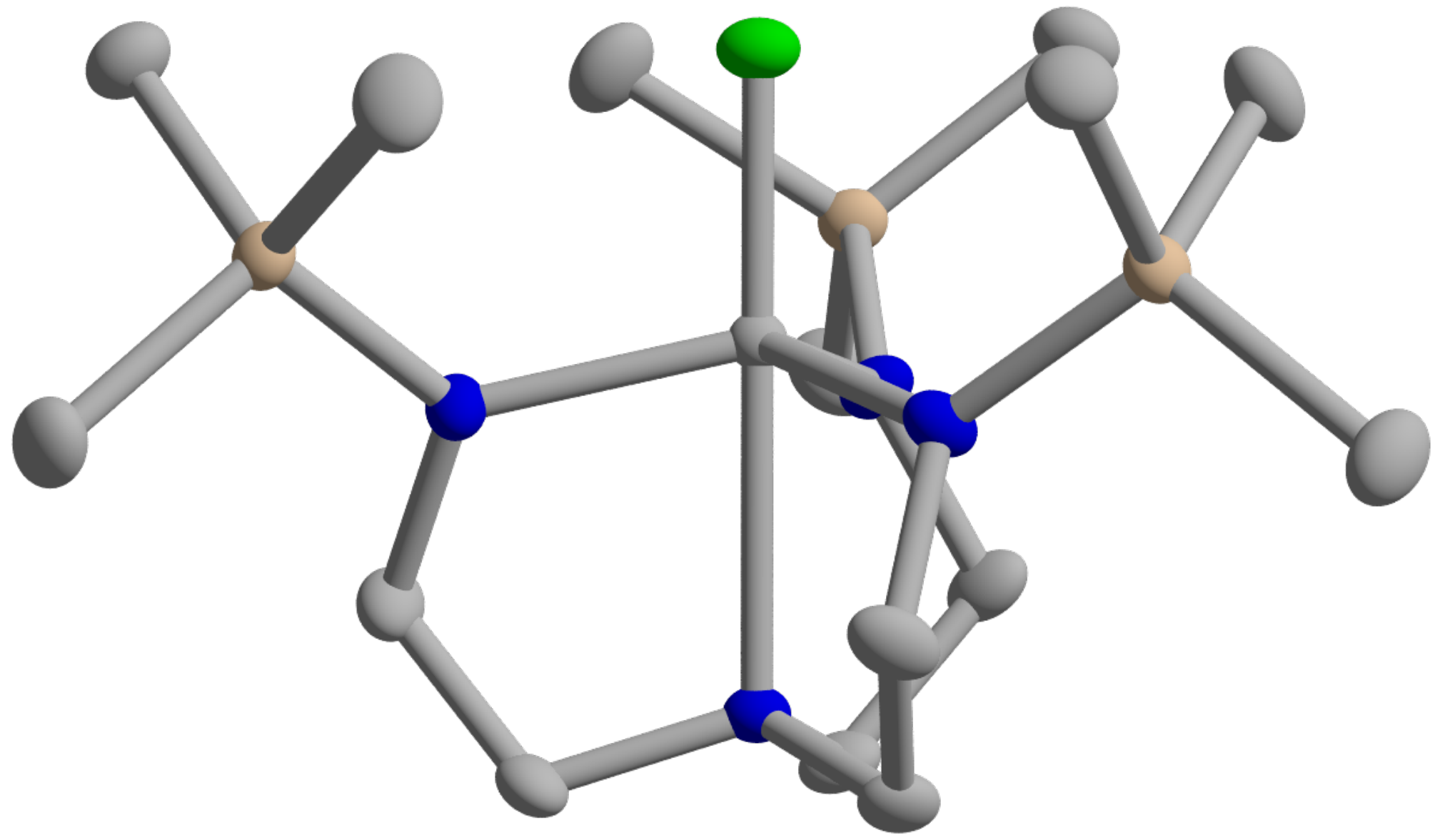

Figure S13 | Thermal ellipsoid plot of **5** crystal structure. Carbon, nitrogen, silicon, fluoride, and titanium atoms are colored gray, blue, tan, green, and gray, respectively. Hydrogen atoms were omitted for clarity.

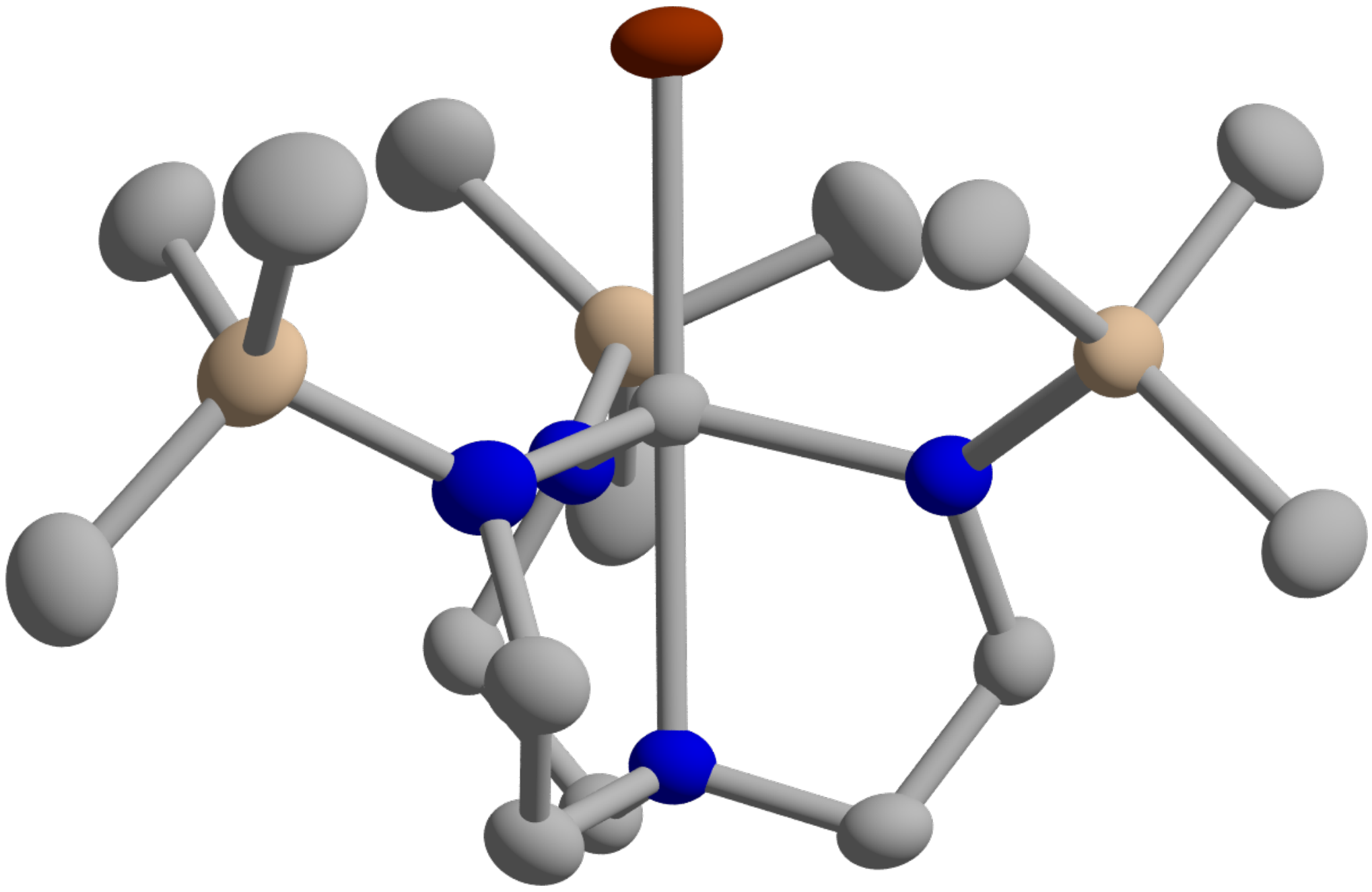

Figure S14 | Thermal ellipsoid plot of **7** crystal structure. Carbon, nitrogen, silicon, bromine, and titanium atoms are colored gray, blue, tan, red, and gray, respectively. Hydrogen atoms and the disordered fragments were omitted for clarity.

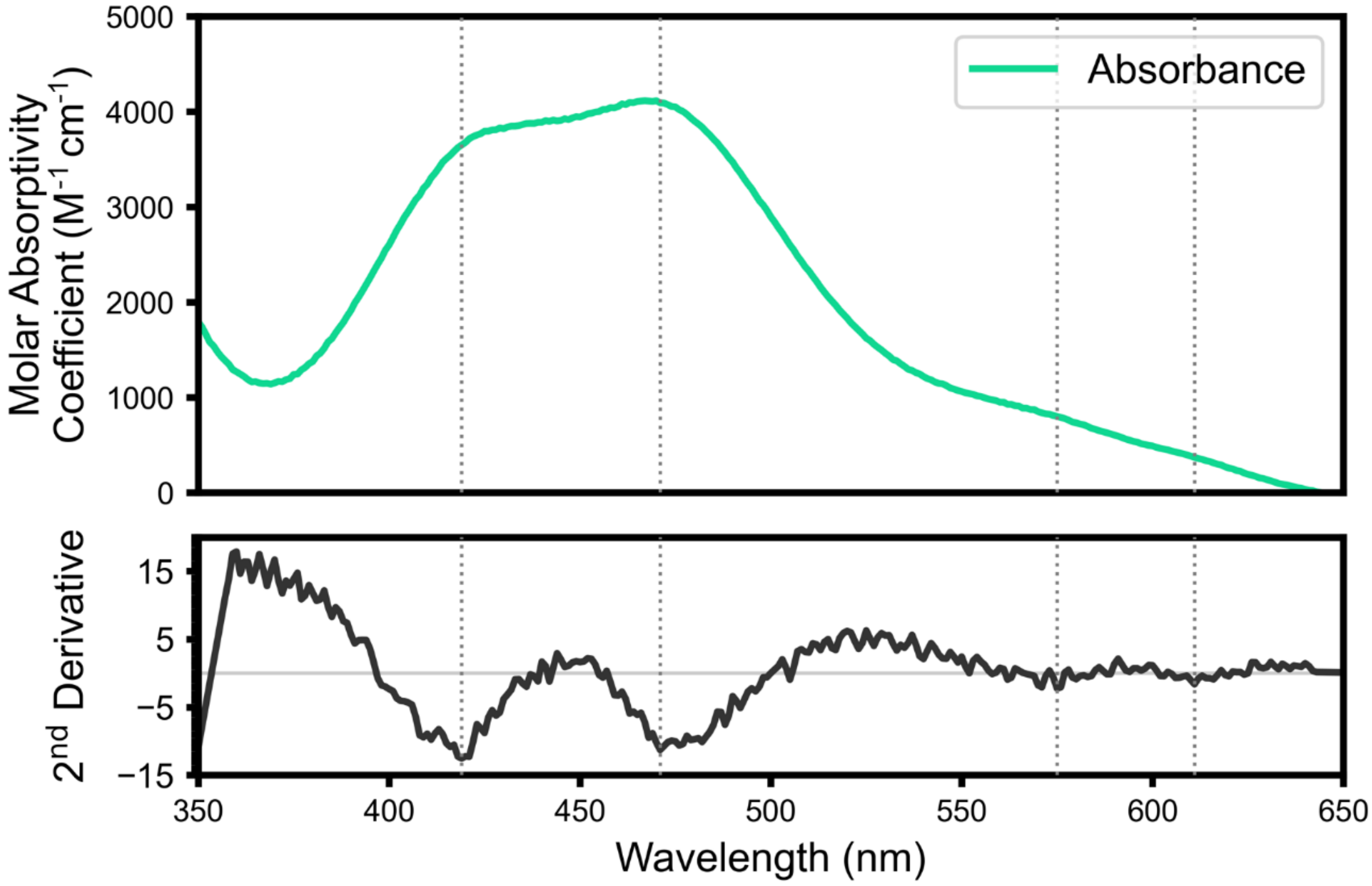

Figure S15 | Top: UV-vis spectrum of **1** in THF at 298 K. Bottom: second derivative of UV-vis spectrum with features denoted by vertical dashed gray lines.

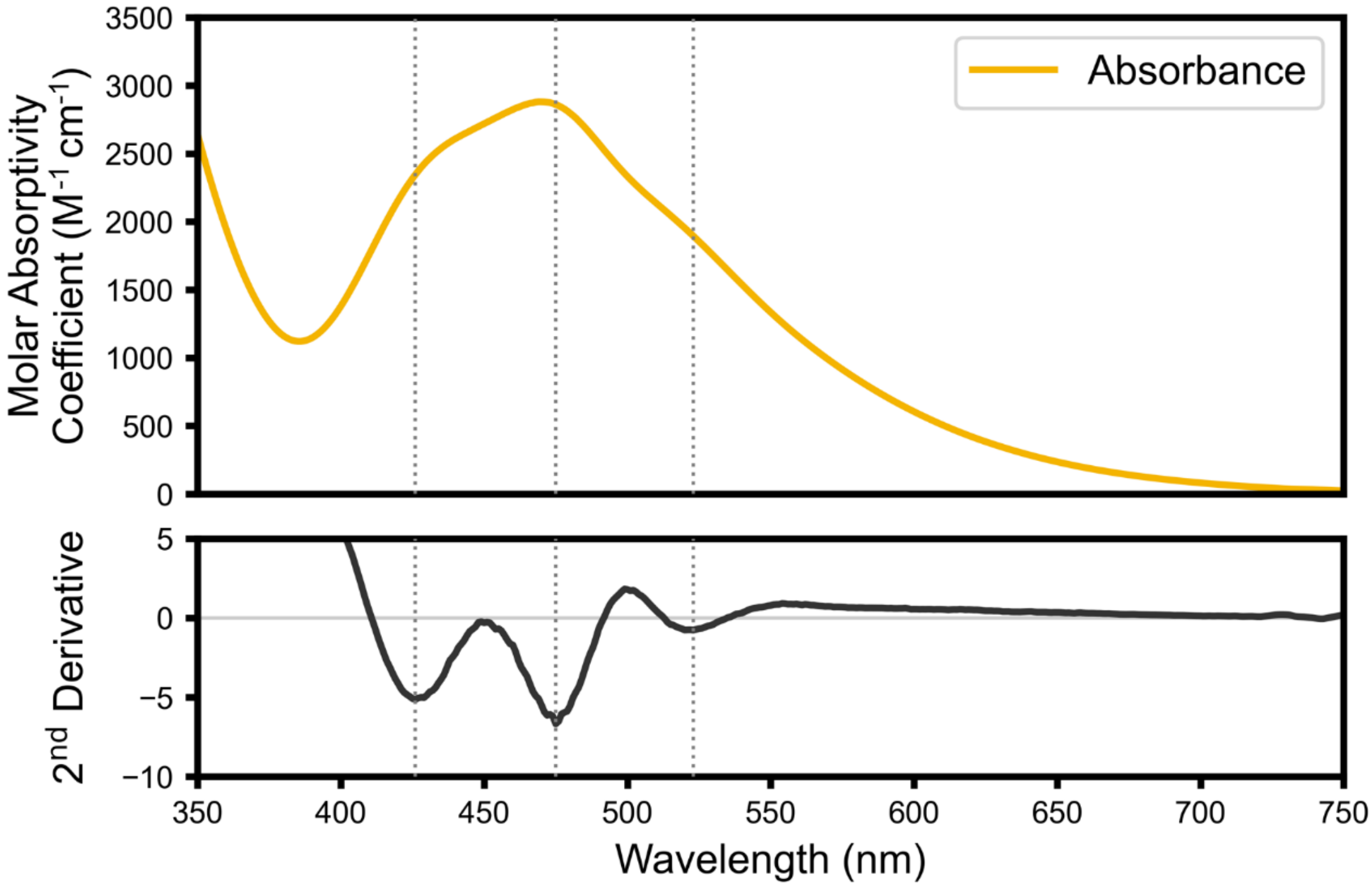

Figure S16 | Top: UV-vis spectrum of **2** in THF at 298 K. Bottom: second derivative of UV-vis spectrum with features denoted by vertical dashed gray lines.

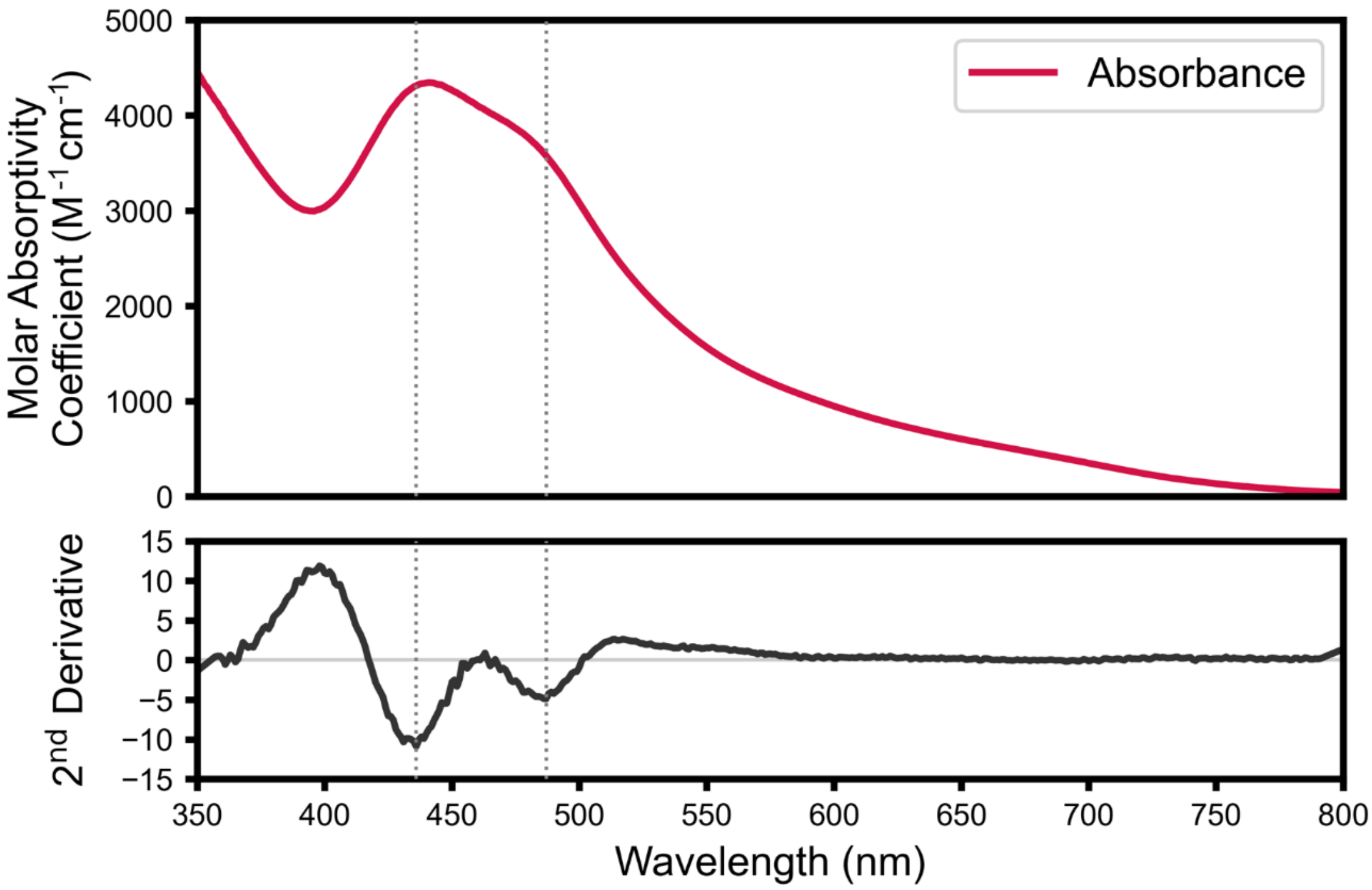

Figure S17 | Top: UV-vis spectrum of **3** in THF at 298 K. Bottom: second derivative of UV-vis spectrum with features denoted by vertical dashed gray lines.

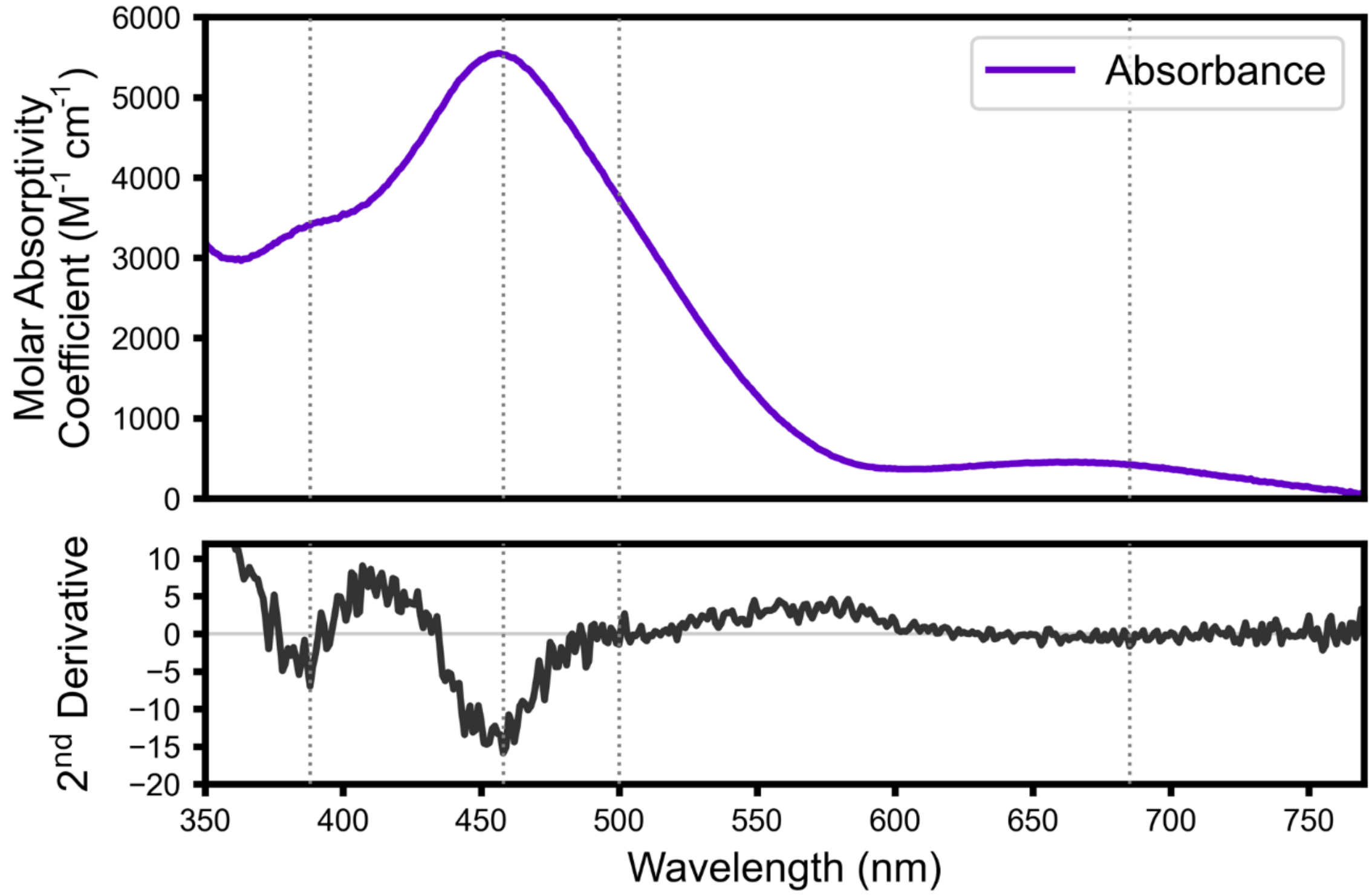

Figure S18 | Top: UV-vis spectrum of **4** in THF at 298 K. Bottom: second derivative of UV-vis spectrum with features denoted by vertical dashed gray lines.

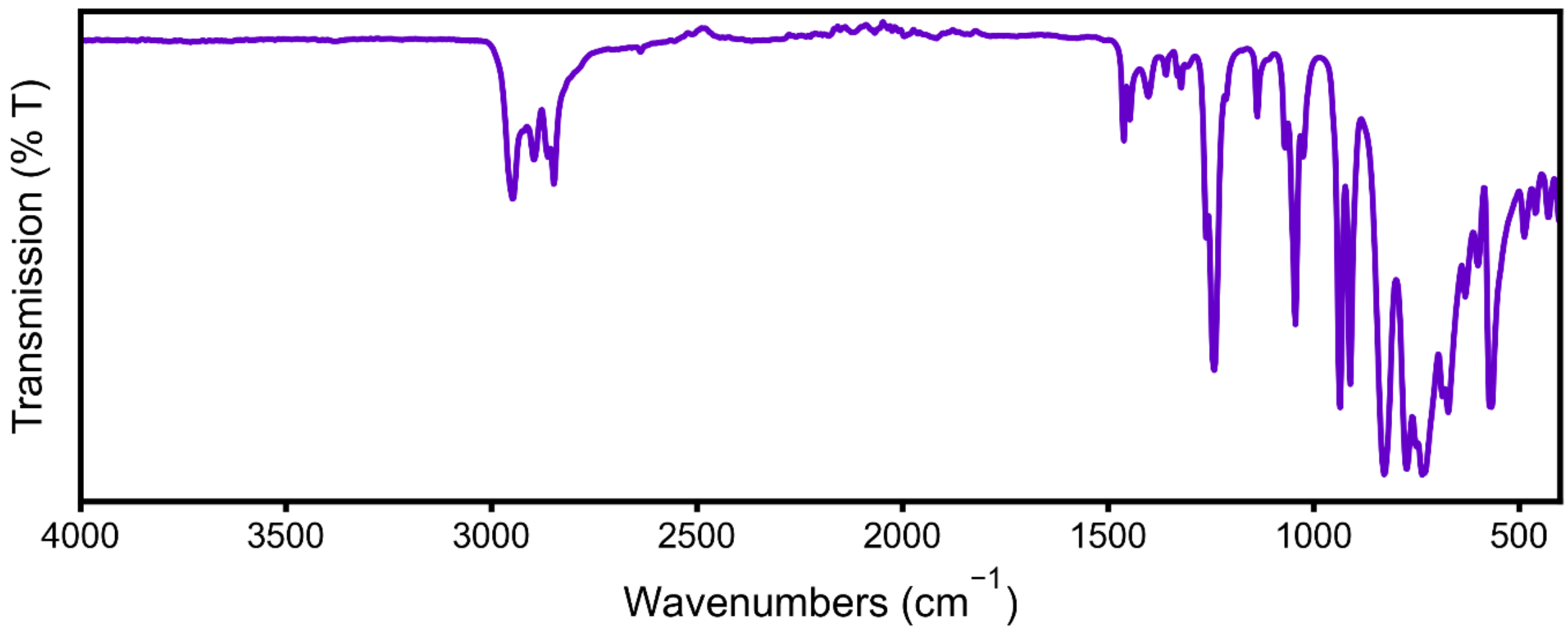

Figure S19 | FT-IR spectrum of **4** crystalline powder at 298 K.

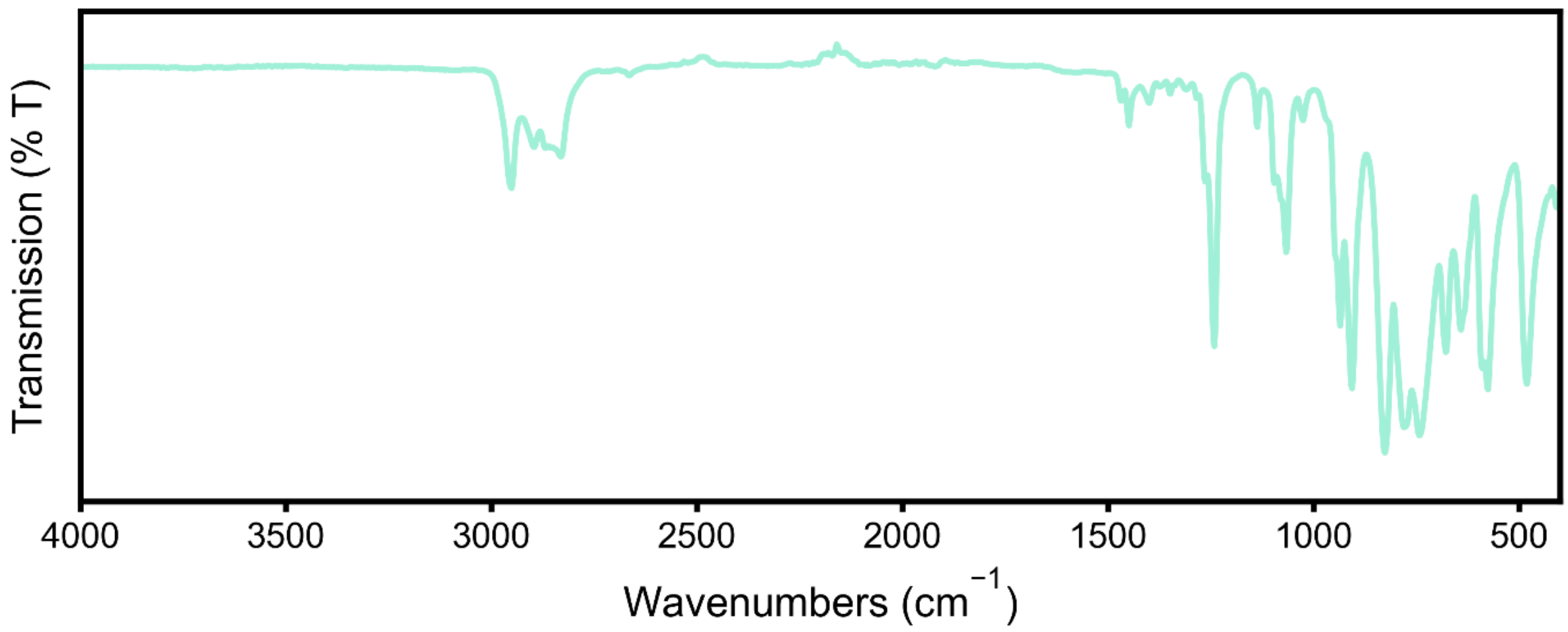

Figure S20 | FT-IR spectrum of **5** crystalline powder at 298 K.

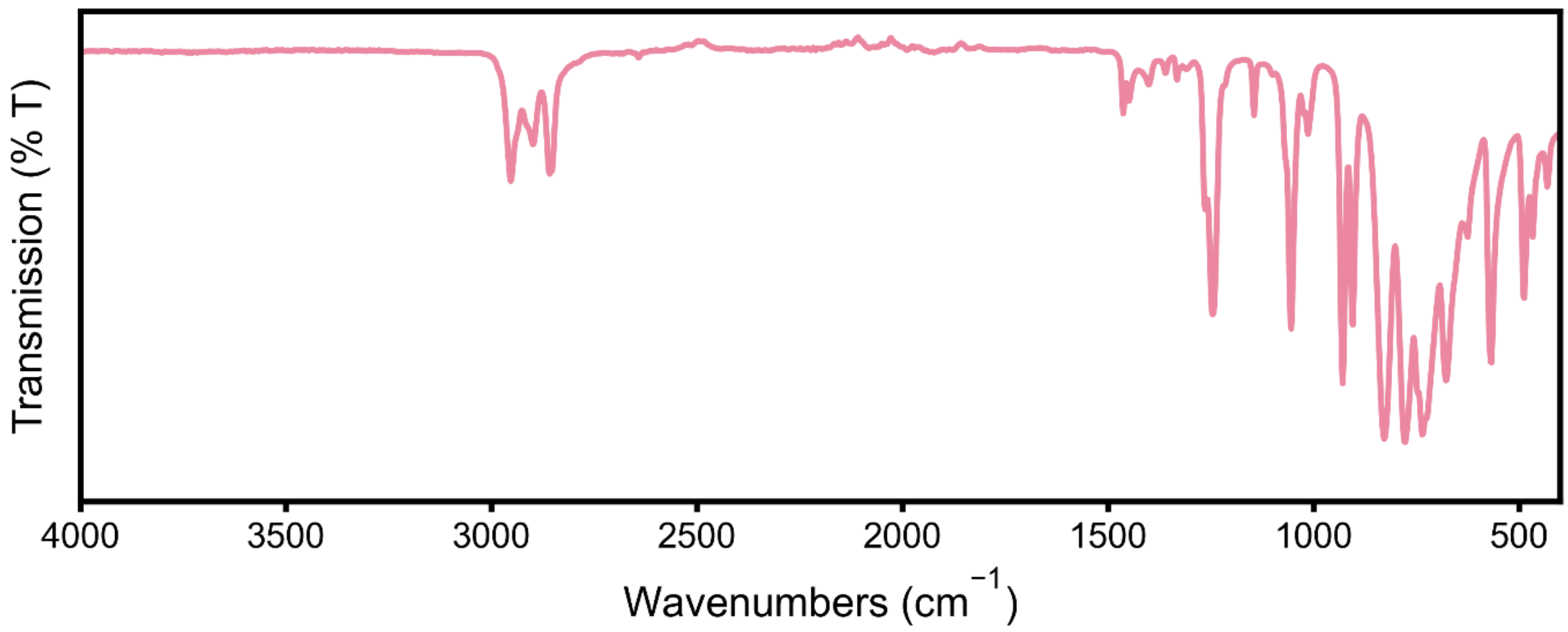

Figure S21 | FT-IR spectrum of **7** crystalline powder at 298 K.

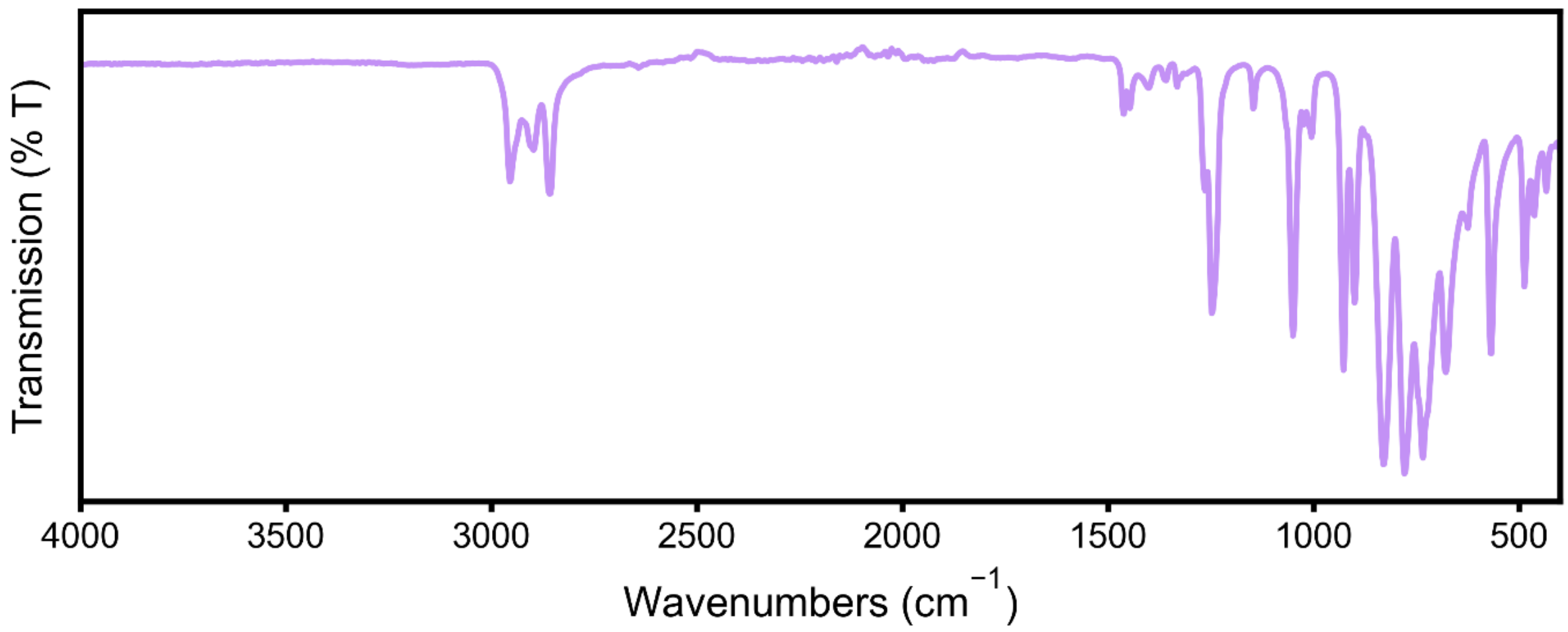

Figure S22 | FT-IR spectrum of **8** crystalline powder at 298 K.

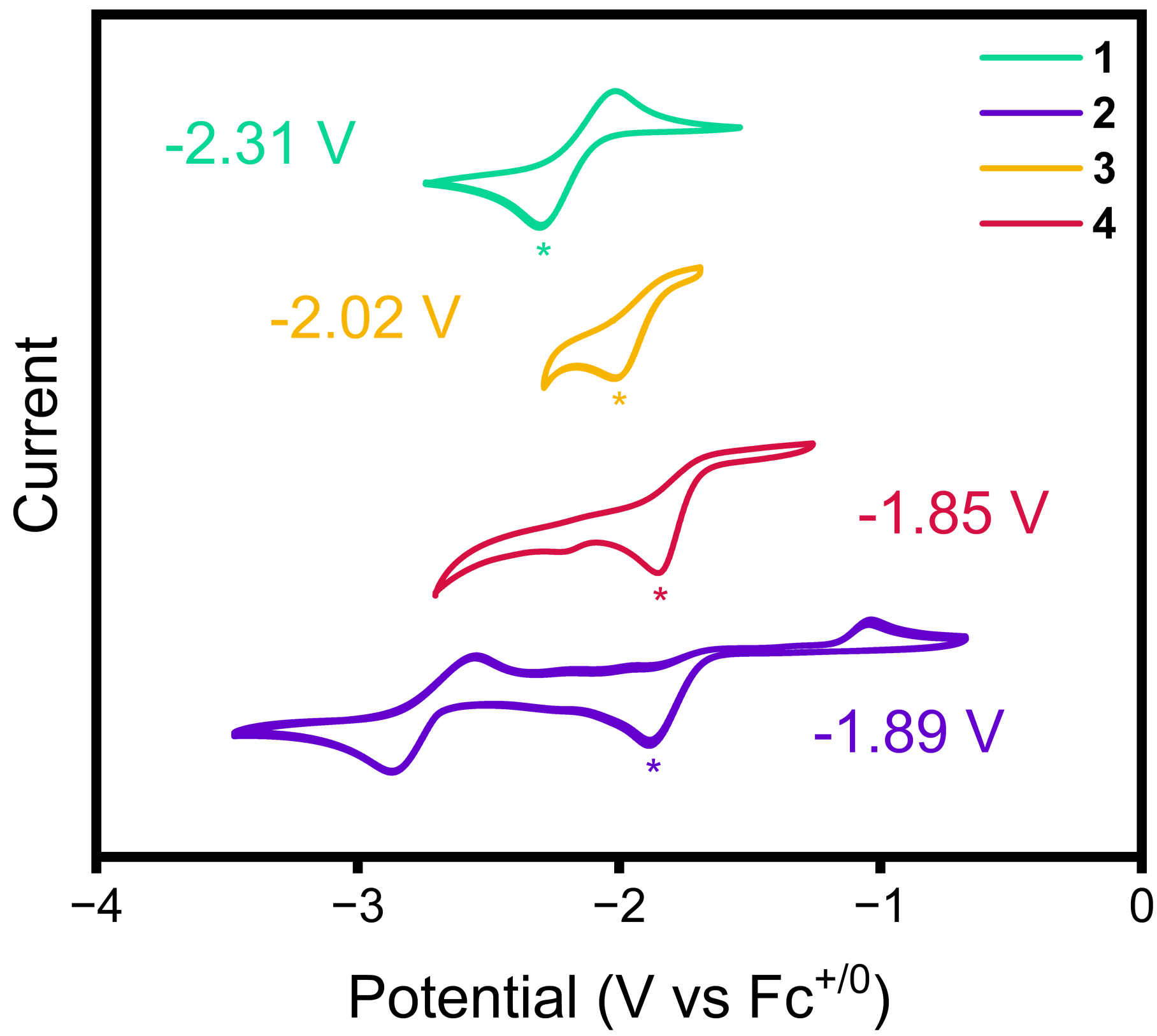

Figure S23 | Narrow scan cyclic voltammograms (CVs) for **1–4**, in THF showing the Cr^{4+}/Cr^{3+} redox wave (scan rate = 50 mV/s). All CVs were performed at 298 K in THF with 0.1 M tetrabutylammonium hexafluorophosphate and 1–2 mM of **1–4** under N_2 atmosphere. Labeled potentials correspond to the position of the reduction event (*).

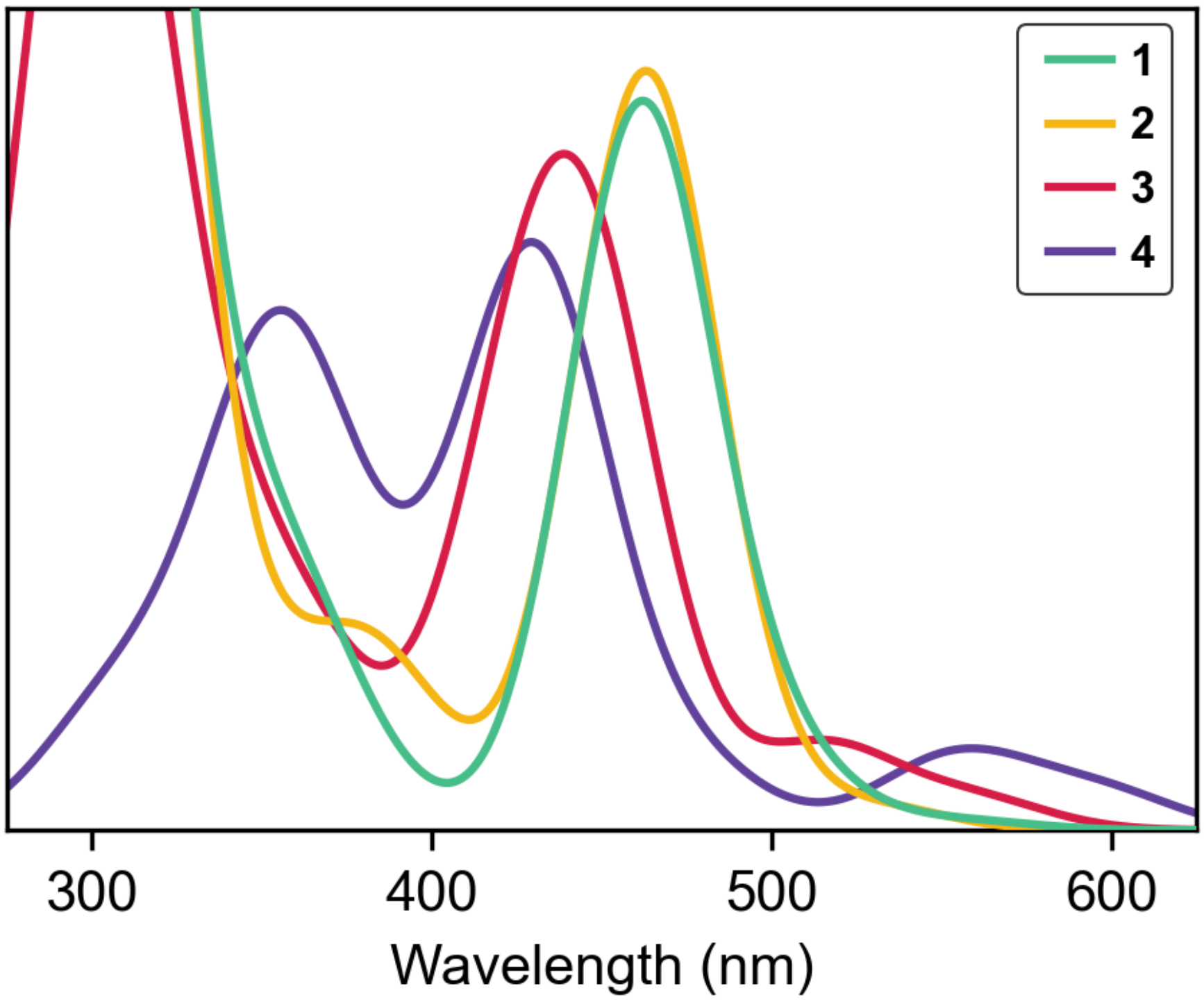

Figure S24 | Absorption spectra of **1–4** generated by TD-DFT**.**

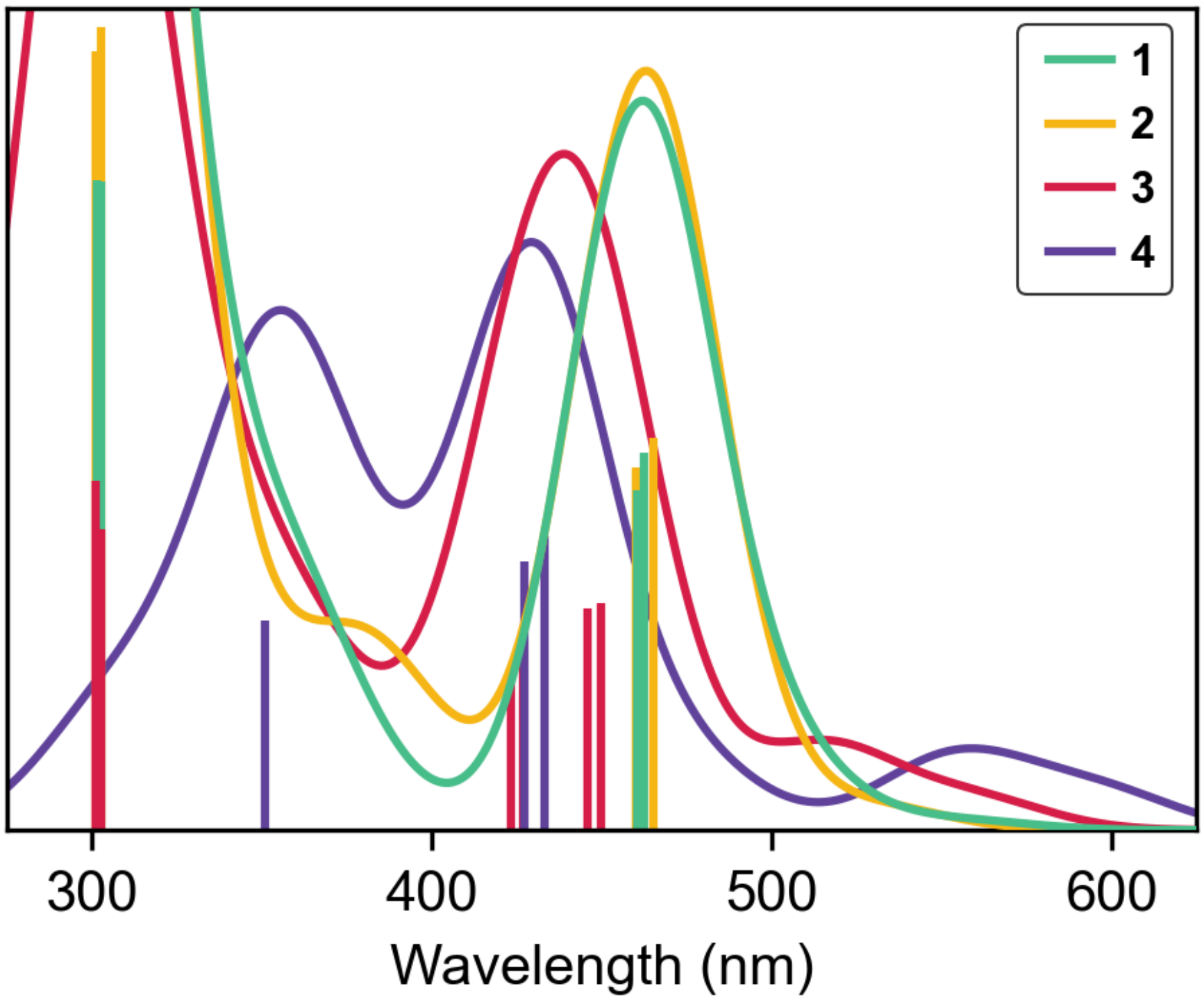

Figure S25 | Absorption spectra of **1–4** generated by TD-DFT with major transitions visualized as sticks.

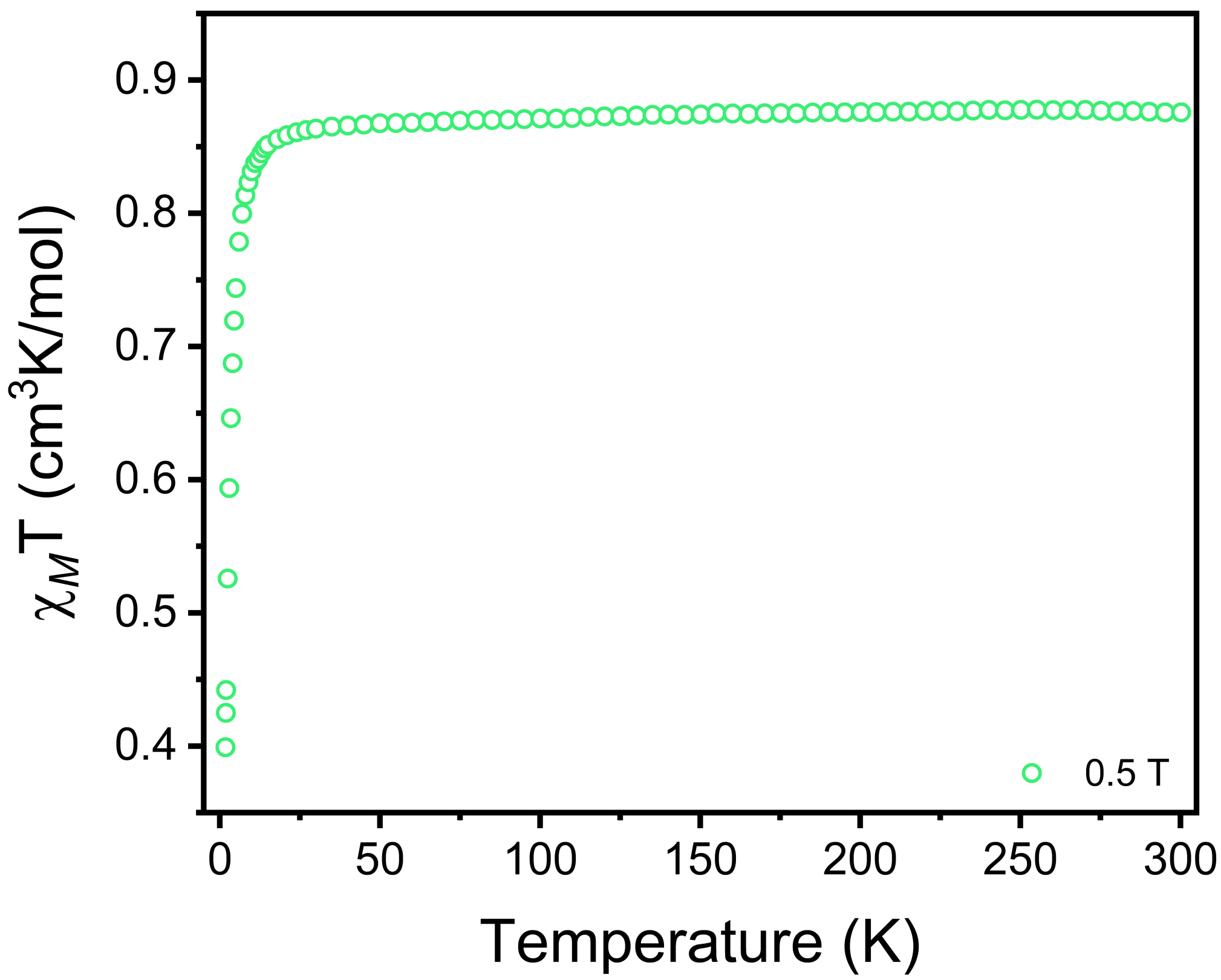

Figure S26 | Variable-temperature dc magnetic susceptibility data for **1** collected from 1.8 K to 300 K under a dc field of 0.5 T.

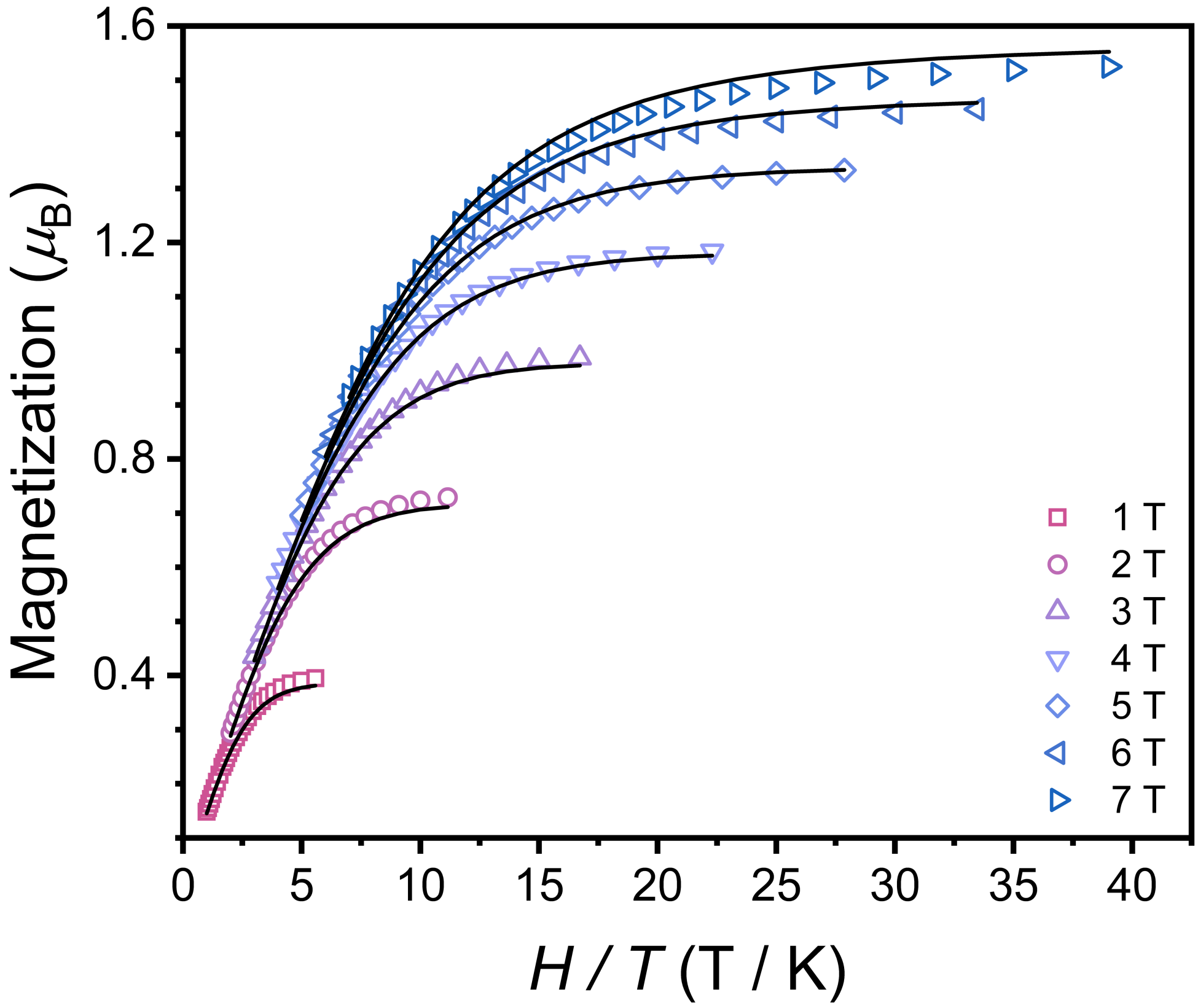

Figure S27 | Variable-temperature, variable-field magnetization data for **1** collected between 1.8 and 10 K from 1 to 7 T in 1 T increments. The black lines are simulations of the magnetization data using the program MagProp with the spin Hamiltonian $\boldsymbol{\hat{H} = D(\hat{S}_z^2 - S(S+1)/3) + \sum_i g_i \mu_B S_i B_i}$. Magnetic parameters: g_{iso} = 1.833, D = +5.34 cm^{-1}. The value of E was restrained to 0 cm^{-1} during the fitting process.

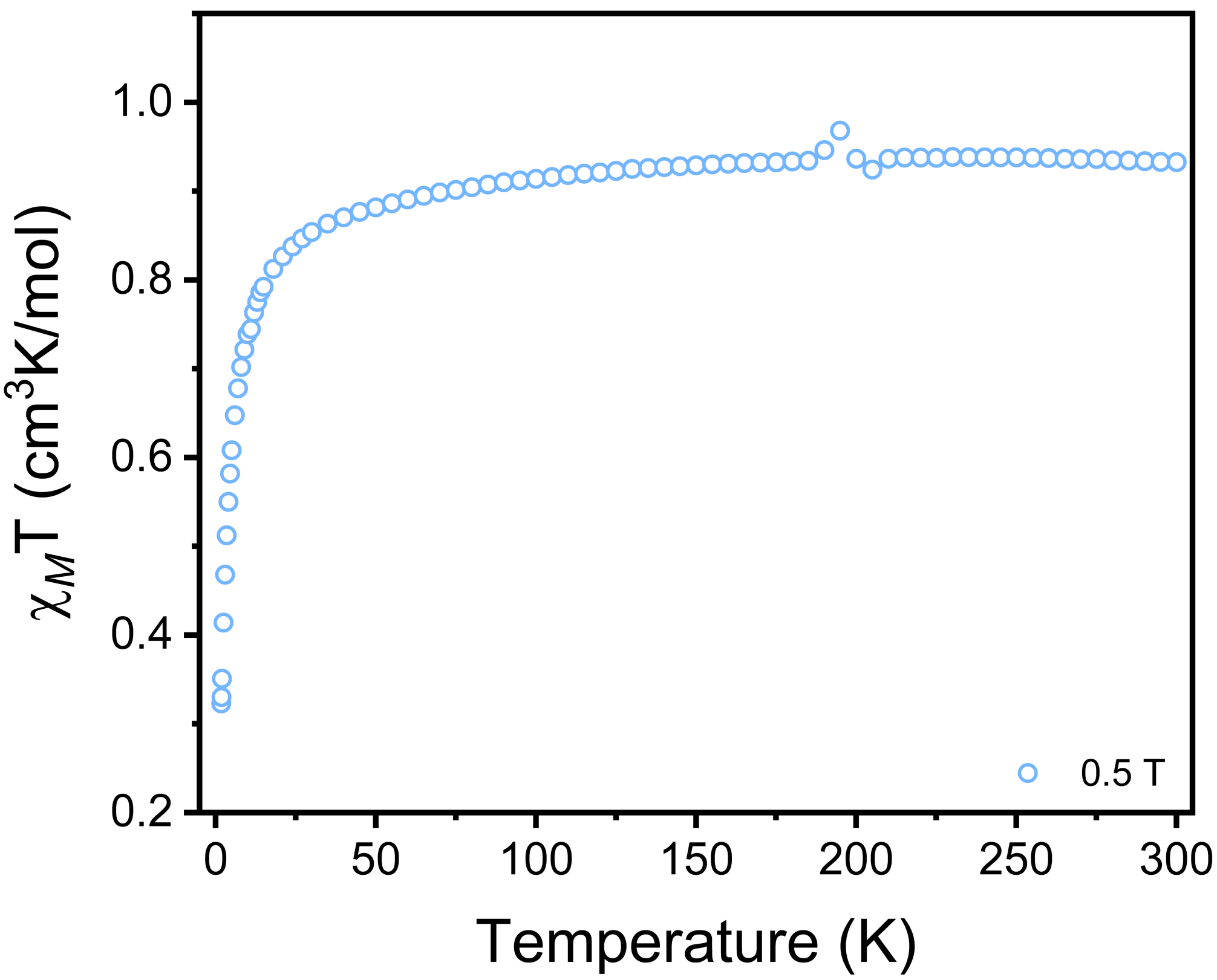

Figure S28 | Variable-temperature dc magnetic susceptibility data for **2** collected from 1.8 K to 300 K under a dc field of 0.5 T.

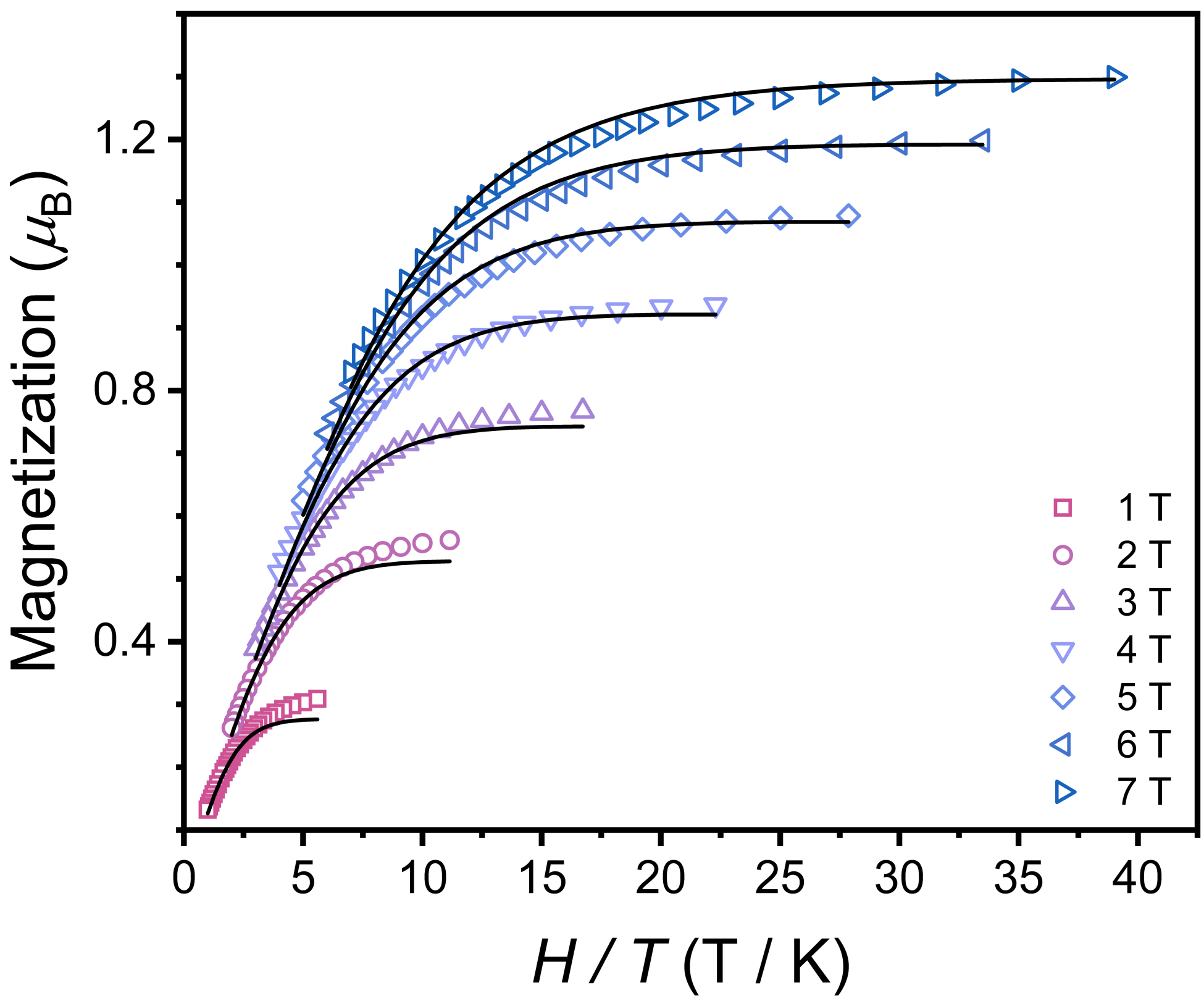

Figure S29 | Variable-temperature, variable-field magnetization data for **2** collected between 1.8 and 10 K from 1 to 7 T in 1 T increments. The black lines are simulations of the magnetization data using the program MagProp with the spin Hamiltonian $\boldsymbol{\hat{H} = D(\hat{S}_z^2 - S(S+1)/3) + \sum_i g_i \mu_B S_i B_i}$. Magnetic parameters: g_{iso} = 1.727, D = +6.68 cm^{-1}. The value of E was restrained to 0 cm^{-1} during the fitting process.

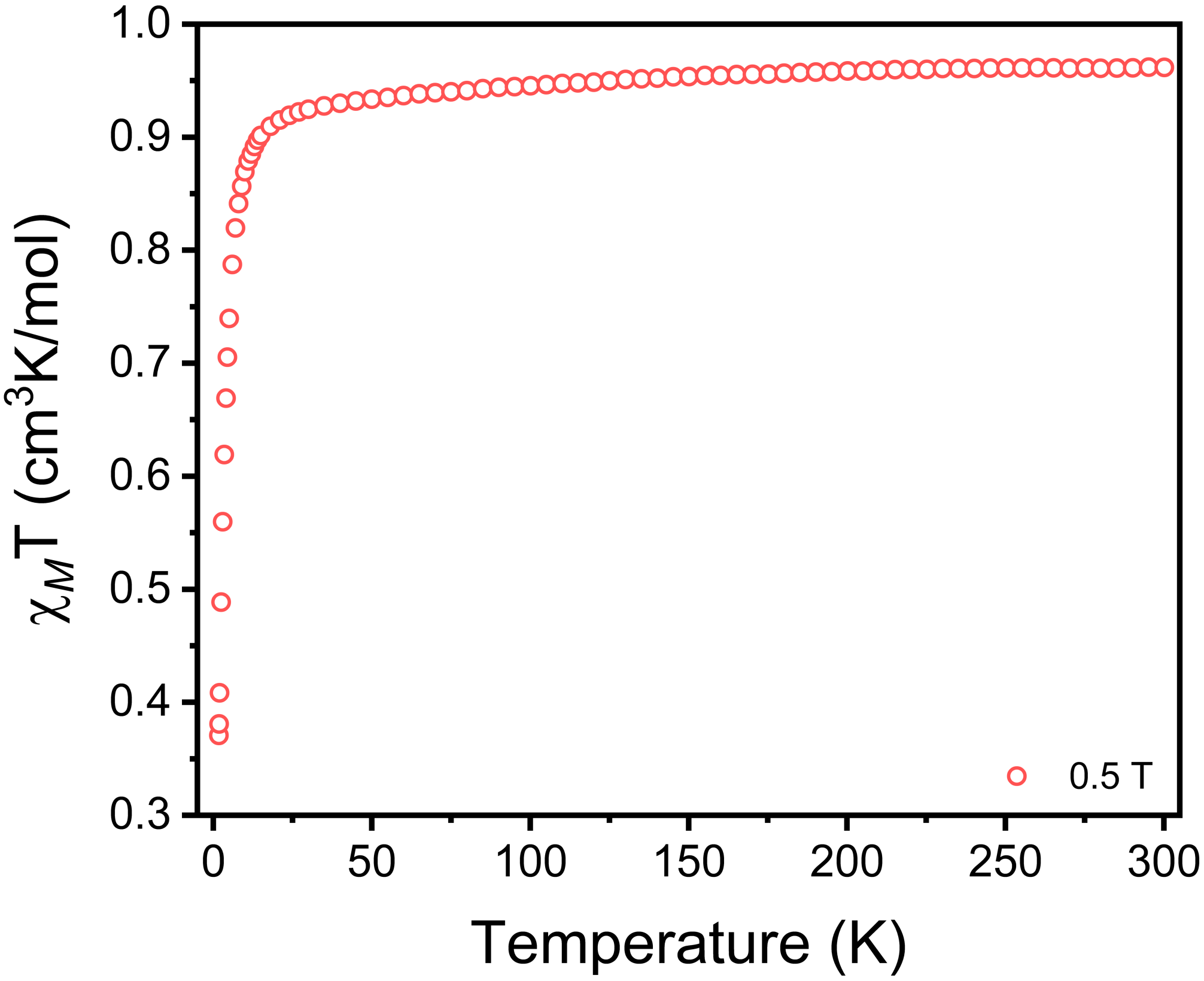

Figure S30 | Variable-temperature dc magnetic susceptibility data for **3** collected from 1.8 K to 300 K under a dc field of 0.5 T.

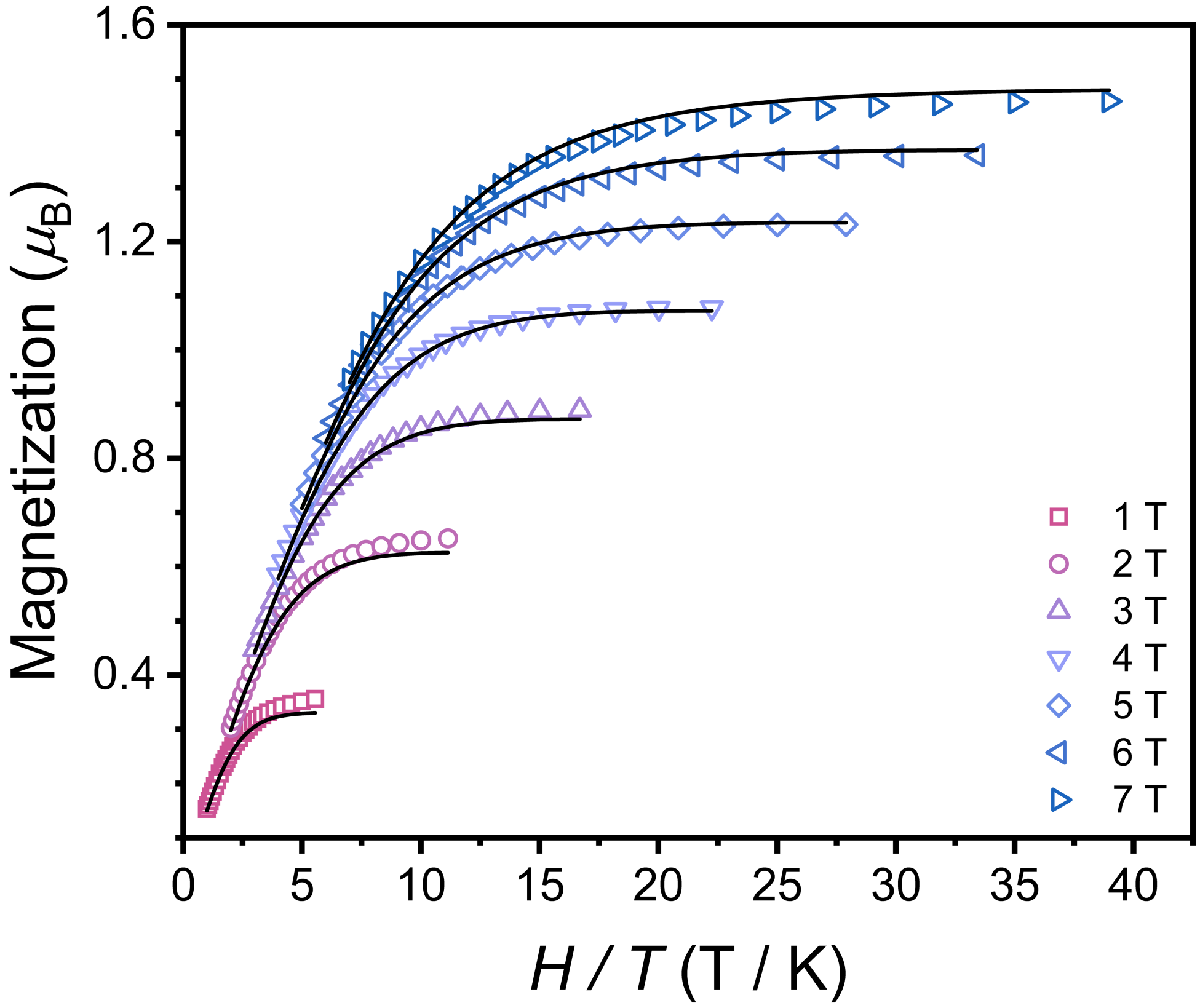

Figure S31 | Variable-temperature, variable-field magnetization data for **3** collected between 1.8 and 10 K from 1 to 7 T in 1 T increments. The black lines are simulations of the magnetization data using the program MagProp with the spin Hamiltonian $\boldsymbol{\hat{H} = D(\hat{S}_z^2 - S(S+1)/3) + \sum_i g_i \mu_B S_i B_i}$. Magnetic parameters: g_{iso} = 1.880, D = +6.60 cm^{-1}. The value of E was restrained to 0 cm^{-1} during the fitting process.

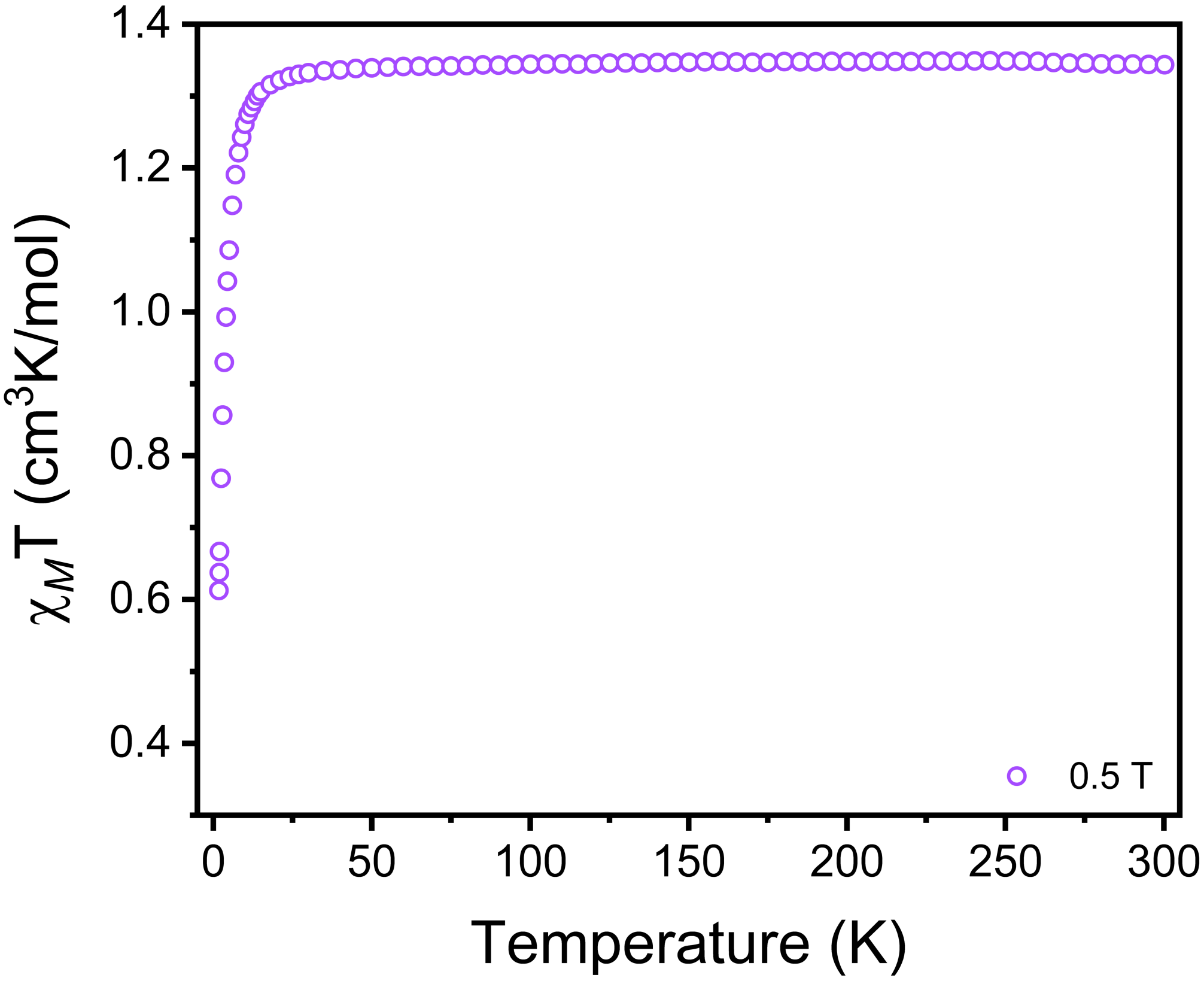

Figure S32 | Variable-temperature dc magnetic susceptibility data for **4** collected from 1.8 K to 300 K under a dc field of 0.5 T.

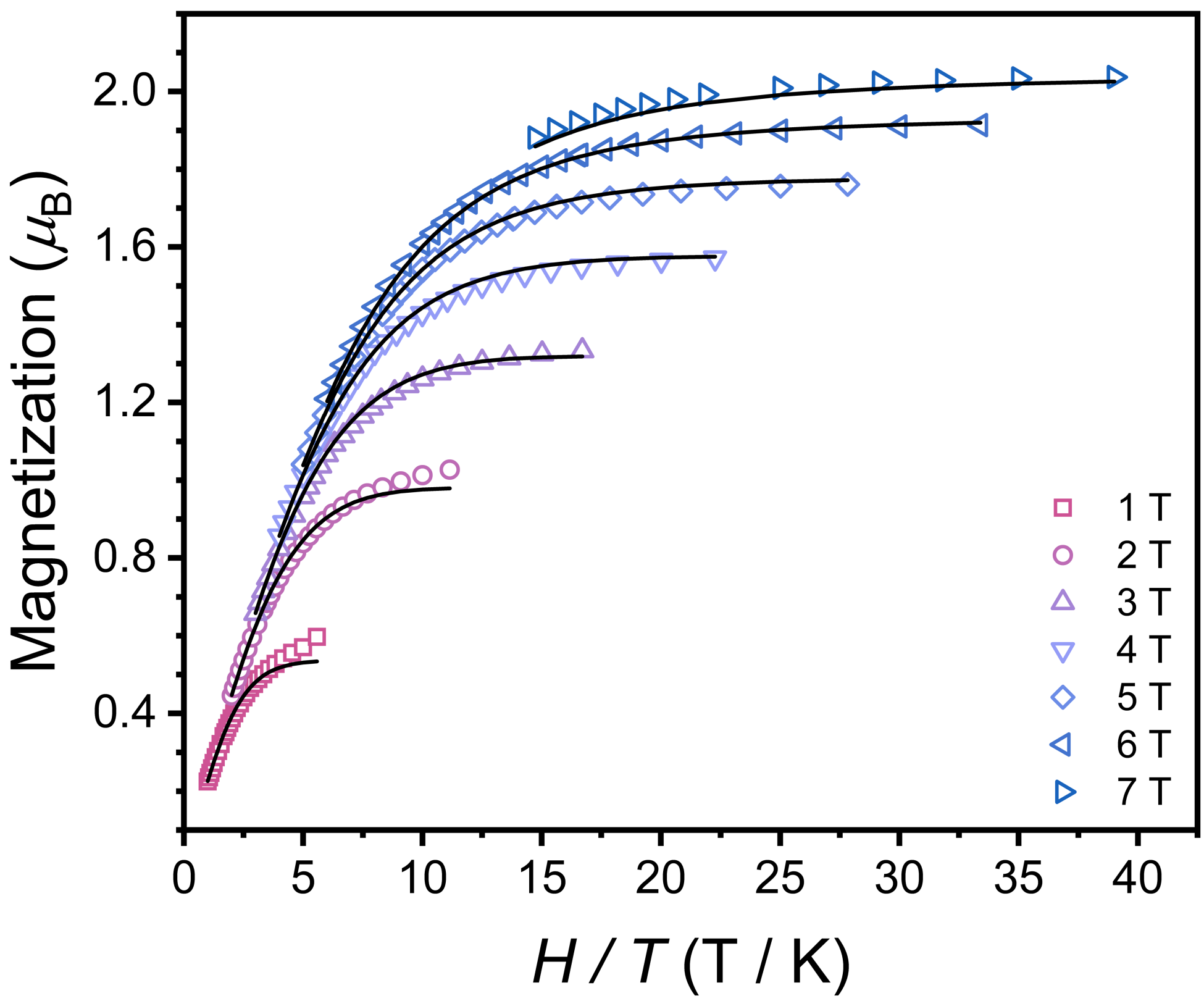

Figure S33 | Variable-temperature, variable-field magnetization data for **4** collected between 1.8 and 10 K from 1 to 7 T in 1 T increments. The black lines are simulations of the magnetization data using the program MagProp with the spin Hamiltonian $\hat{\boldsymbol{H}} = \boldsymbol{D}(\hat{\boldsymbol{S}}_z^2 - \boldsymbol{S}(\boldsymbol{S}+\mathbf{1})/\mathbf{3}) + \sum_i \mathbf{g}_i \boldsymbol{\mu}_B \boldsymbol{S}_i \boldsymbol{B}_i$. Magnetic parameters: g_{iso} = 2.298, D = +6.00 cm^{-1}. The value of E was restrained to 0 cm^{-1} during the fitting process.

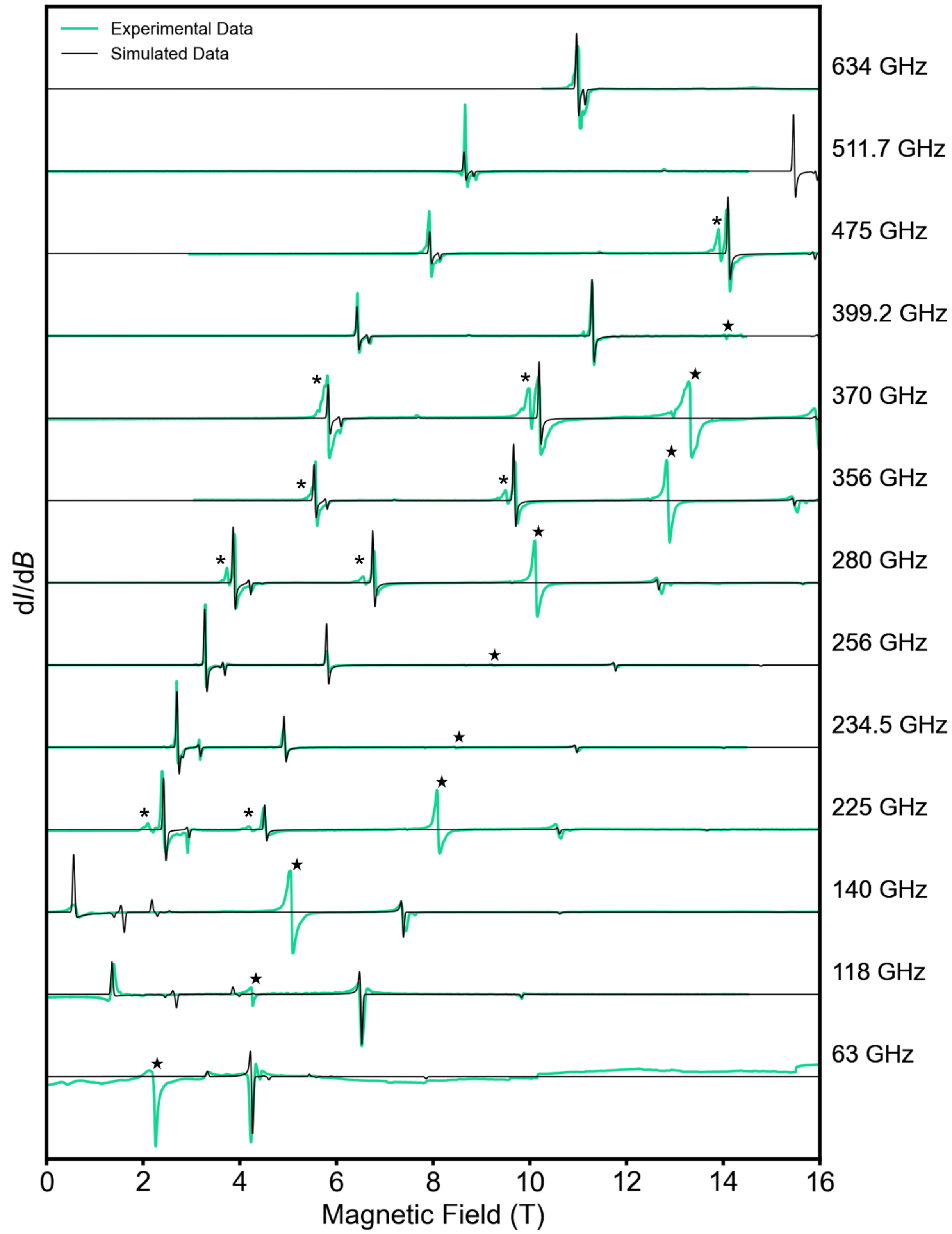

Figure S34 | Variable-frequency HFHF cw-EPR spectra for **1** at 10 K using spin Hamiltonian parameters from Table S21**.** Stars denote Cr^{3+} impurities, asterisks denote byproduct **2**, green lines denote experimental data, and black lines denote simulated data.

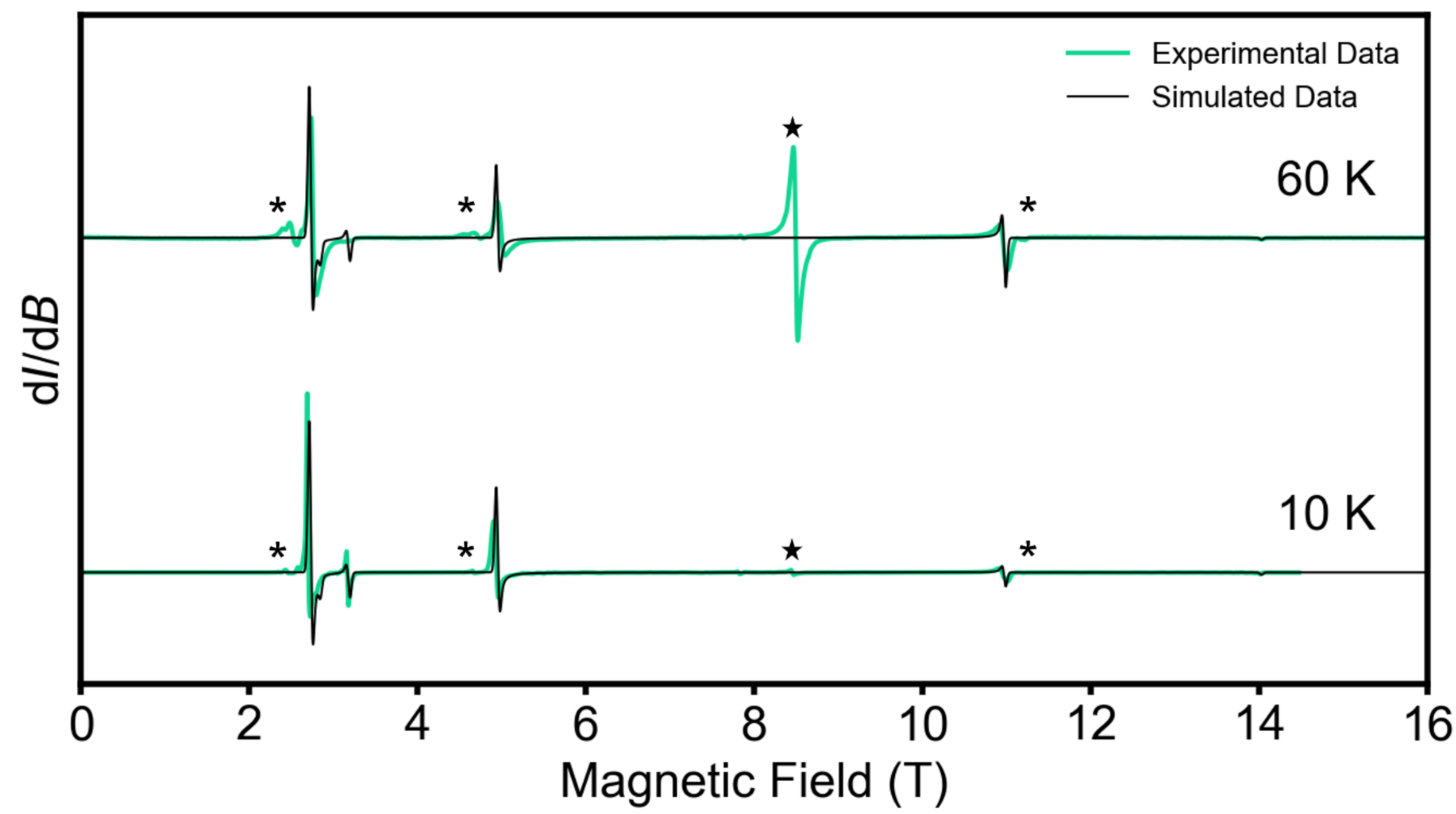

Figure S35 | Variable-temperature HFHF cw-EPR spectra for **1** at 235 GHz**.** Stars denote Cr^{3+} impurities, asterisks denote byproduct **2**, green lines denote experimental data, and black lines denote simulated data.

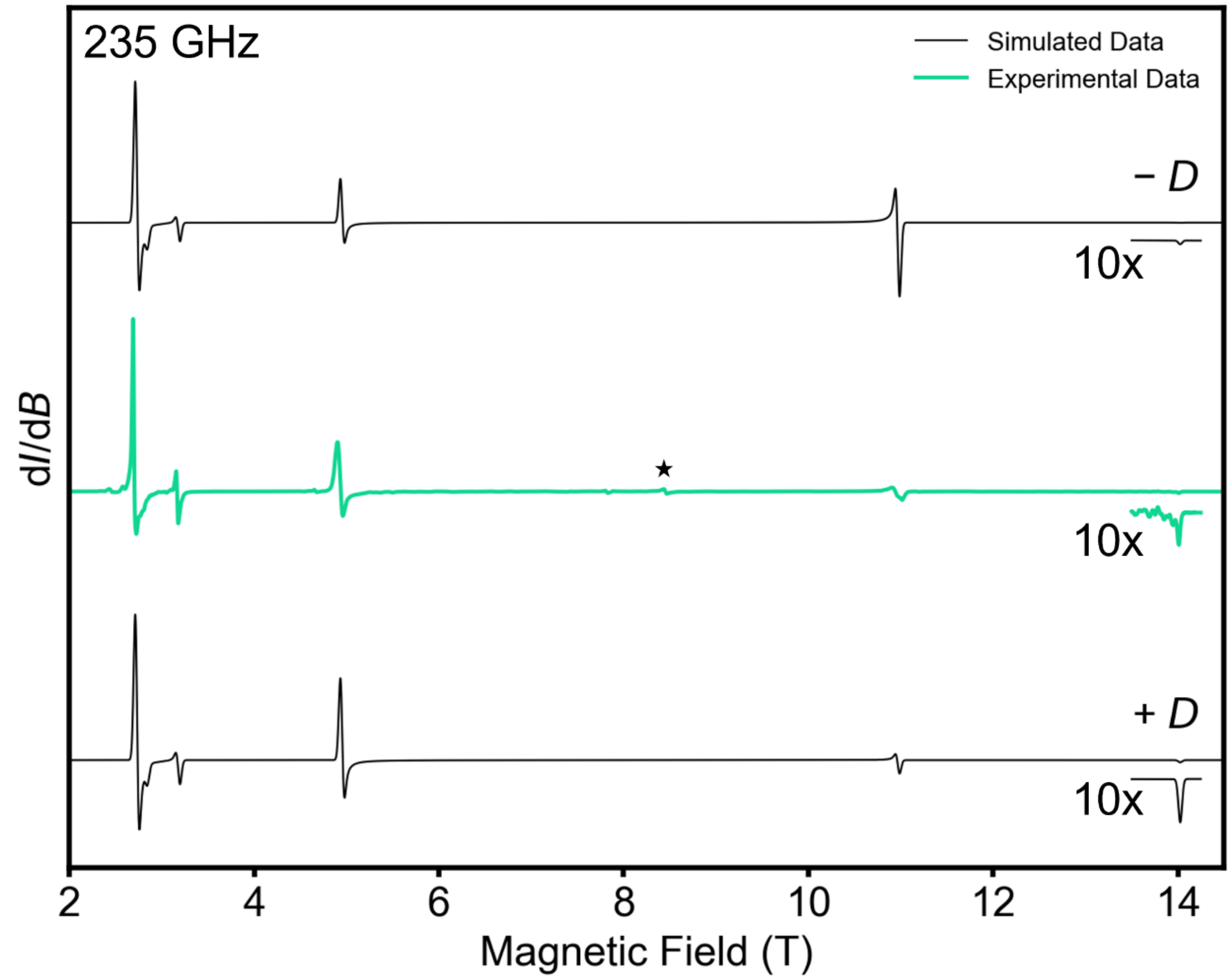

Figure S36 | Simulated EPR spectra for **1** using the spin Hamiltonian parameters in Table S21, a temperature of 10 K, and either a negative (top) or positive (bottom) value of *D***.** The experimental spectrum best agrees with the simulated EPR spectrum using a positive value of *D*. Stars denote Cr^{3+} impurities, green lines denote experimental data, and black lines denote simulated data.

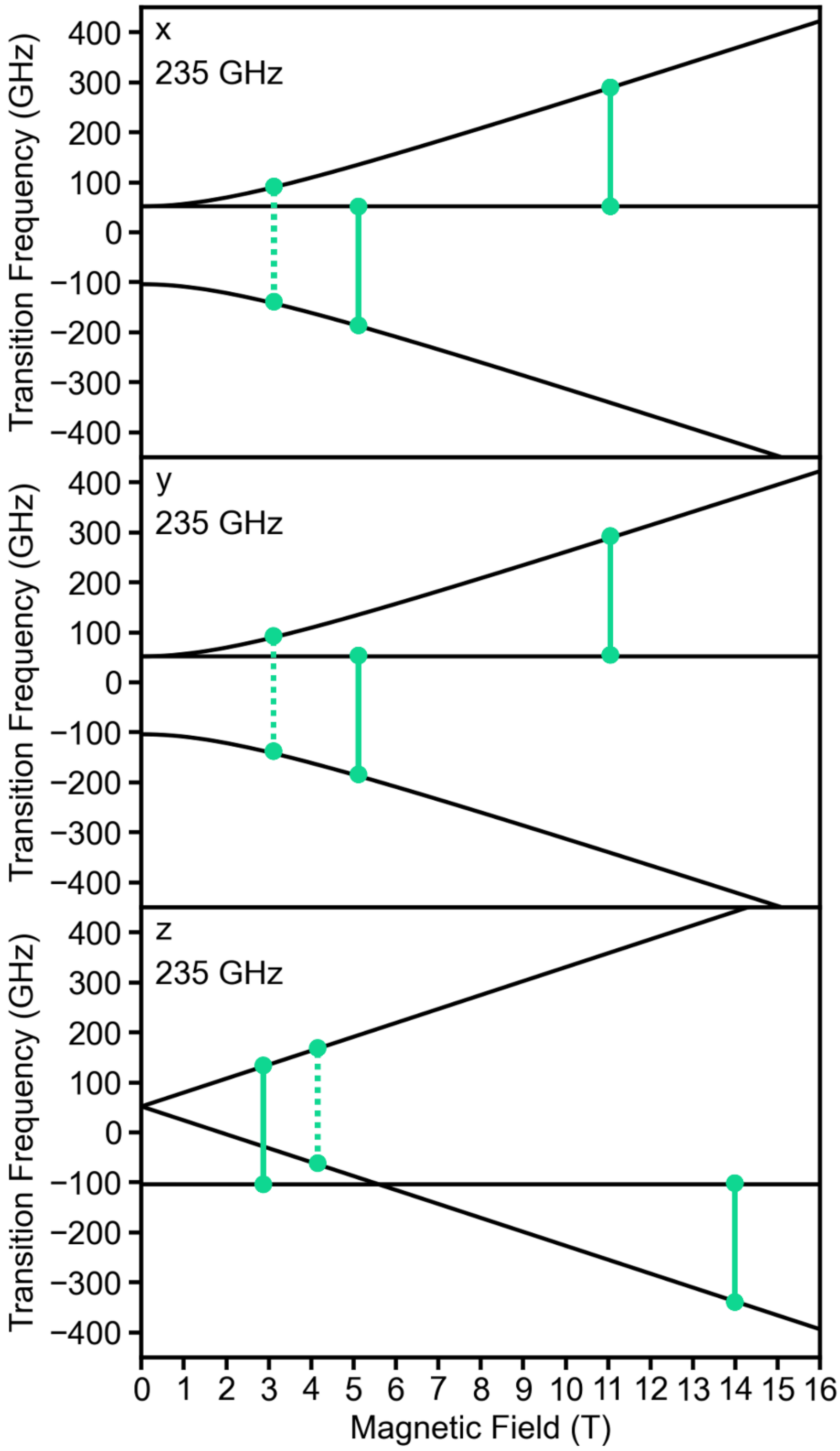

Figure S37 | Zeeman diagrams along the x, y, and z axes for **1** based on the spin Hamiltonian parameters in Table S21. The solid green lines denote the allowed ($\Delta m_s = 1$) and the dashed green lines denote the unallowed ($\Delta m_s = 2$) transitions at 235 GHz. These Zeeman diagrams were simulated using a positive value of D; the higher-energy transition around 11 T for the x, y axes should diminish in intensity as temperature decreases and that state depopulates if the sign of D is indeed positive.

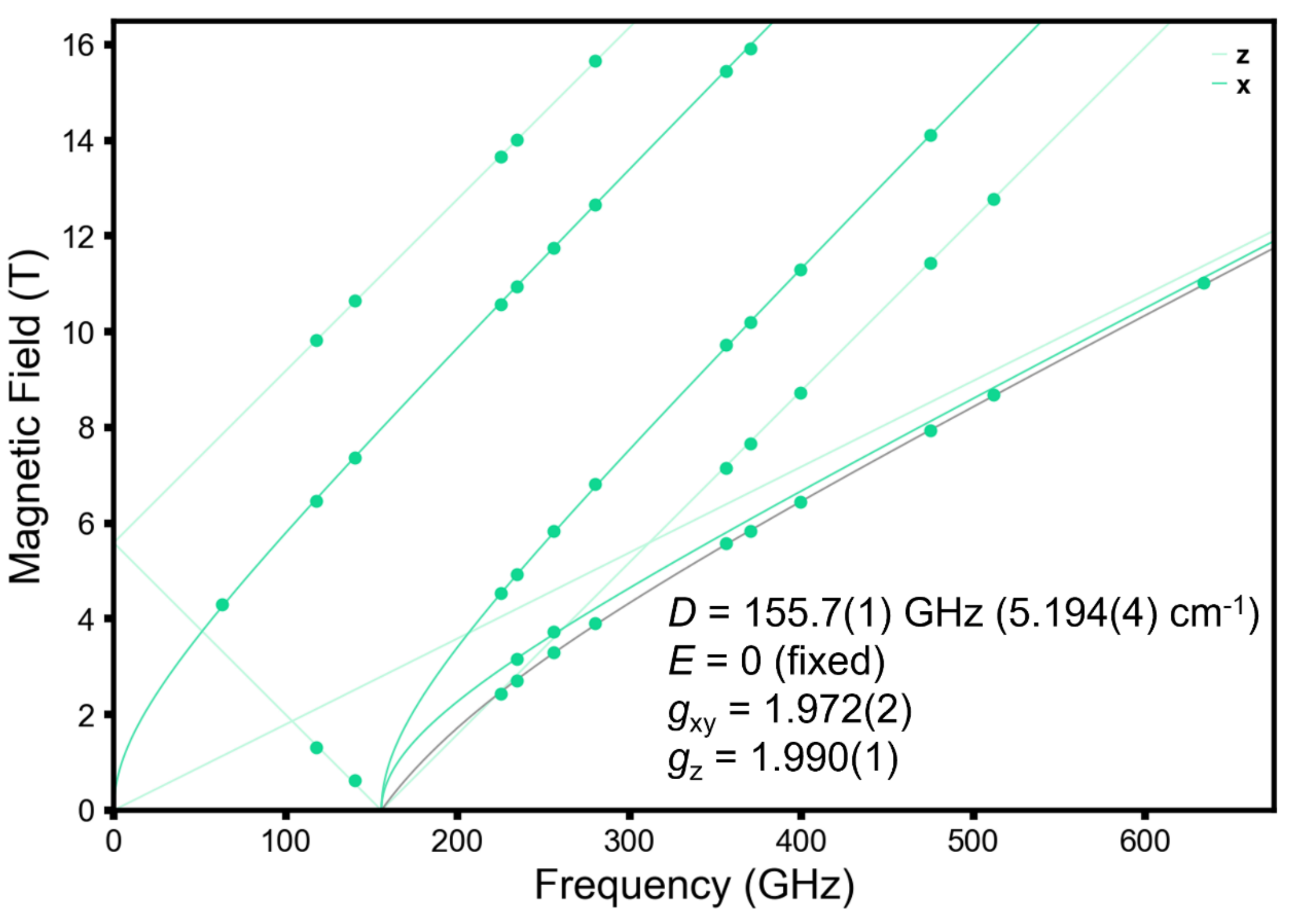

Figure S38 | Frequency dependencies of the resonance fields observed in **1** at 10 K. Green datapoints are features from experimental spectra. The light green and dark green lines are transitions simulated along the molecular orientations along the x and z axes using the spin Hamiltonian parameters in Table S21. The gray line represents the "half-field" transition, which is an off-axial turning point, and generated from the resonance fields read out of the simulated powder spectra.

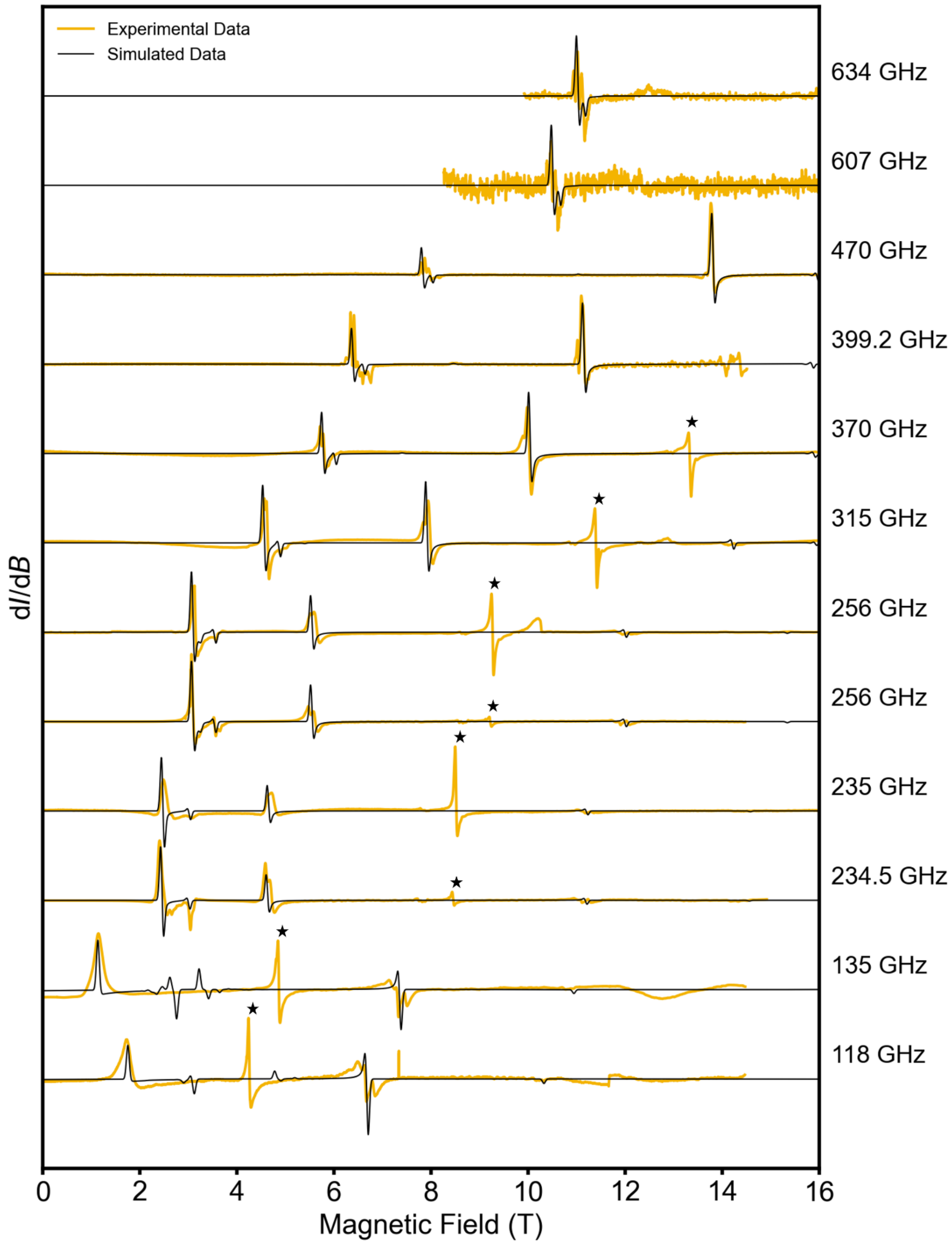

Figure S39 | Variable-frequency HFHF cw-EPR spectra for **2** at 10 K using spin Hamiltonian parameters from Table S21**.** Stars denote Cr^{3+} impurities, yellow lines denote experimental data, and black lines denote simulated data.

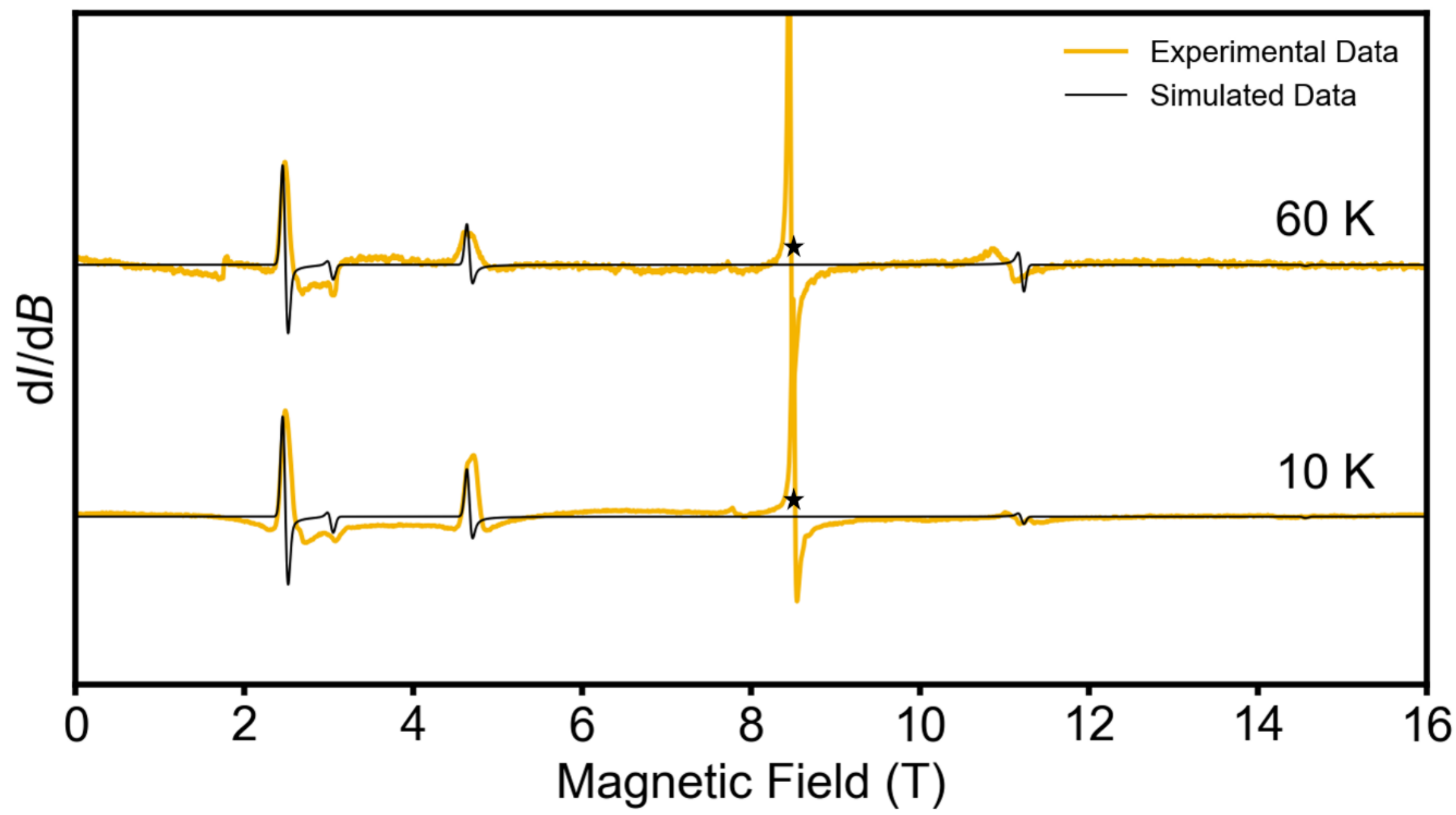

Figure S40 | Variable-temperature HFHF cw-EPR spectra for **2** at 235 GHz**.** Stars denote Cr^{3+} impurities, yellow lines denote experimental data, and black lines denote simulated data.

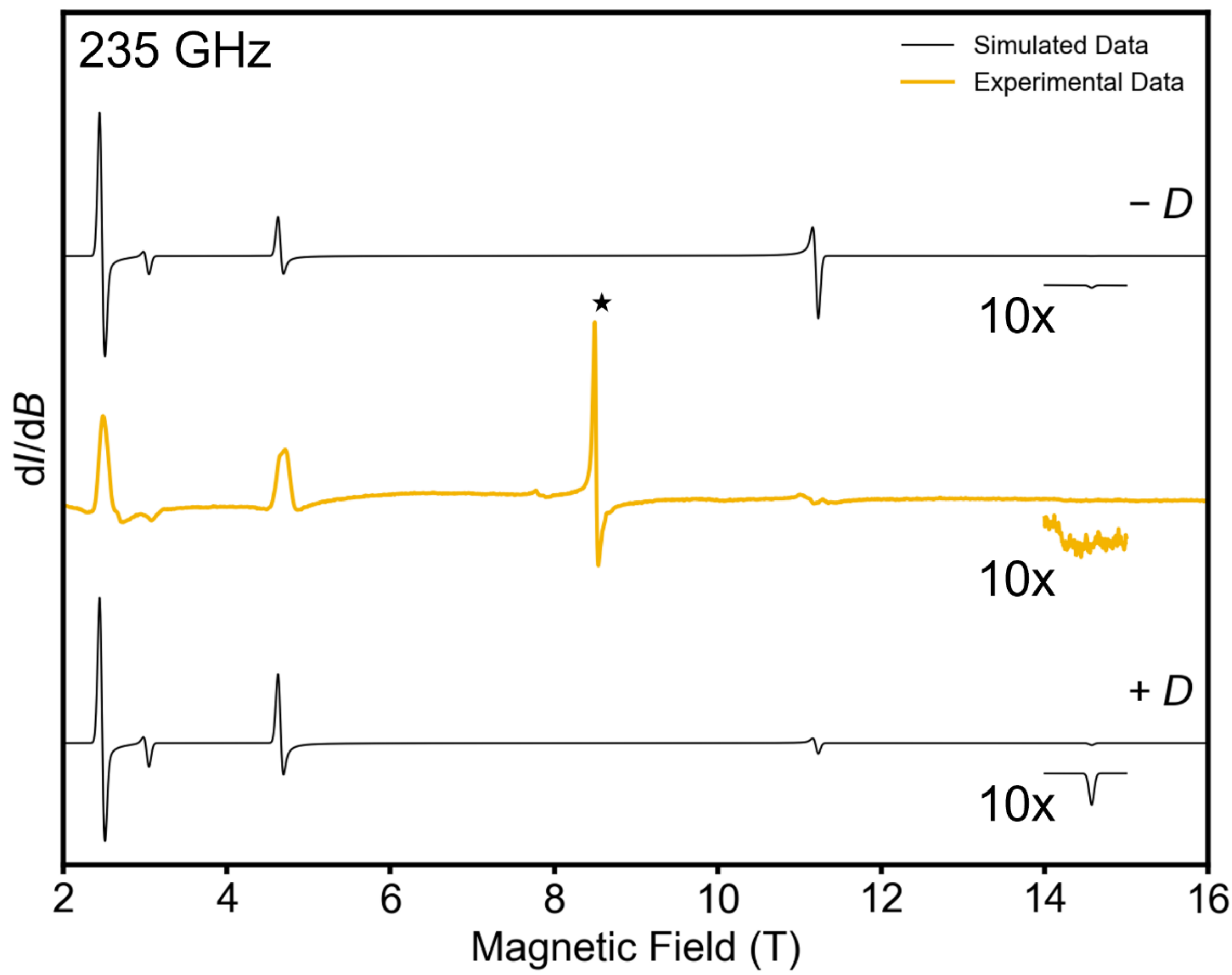

Figure S41 | Simulated EPR spectra for **2** using the spin Hamiltonian parameters in Table S21, a temperature of 10 K, and either a negative (top) or positive (bottom) value of *D***.** The experimental spectrum best agrees with the simulated EPR spectrum using a positive value of *D*. Stars denote Cr^{3+} impurities, yellow lines denote experimental data, and black lines denote simulated data.

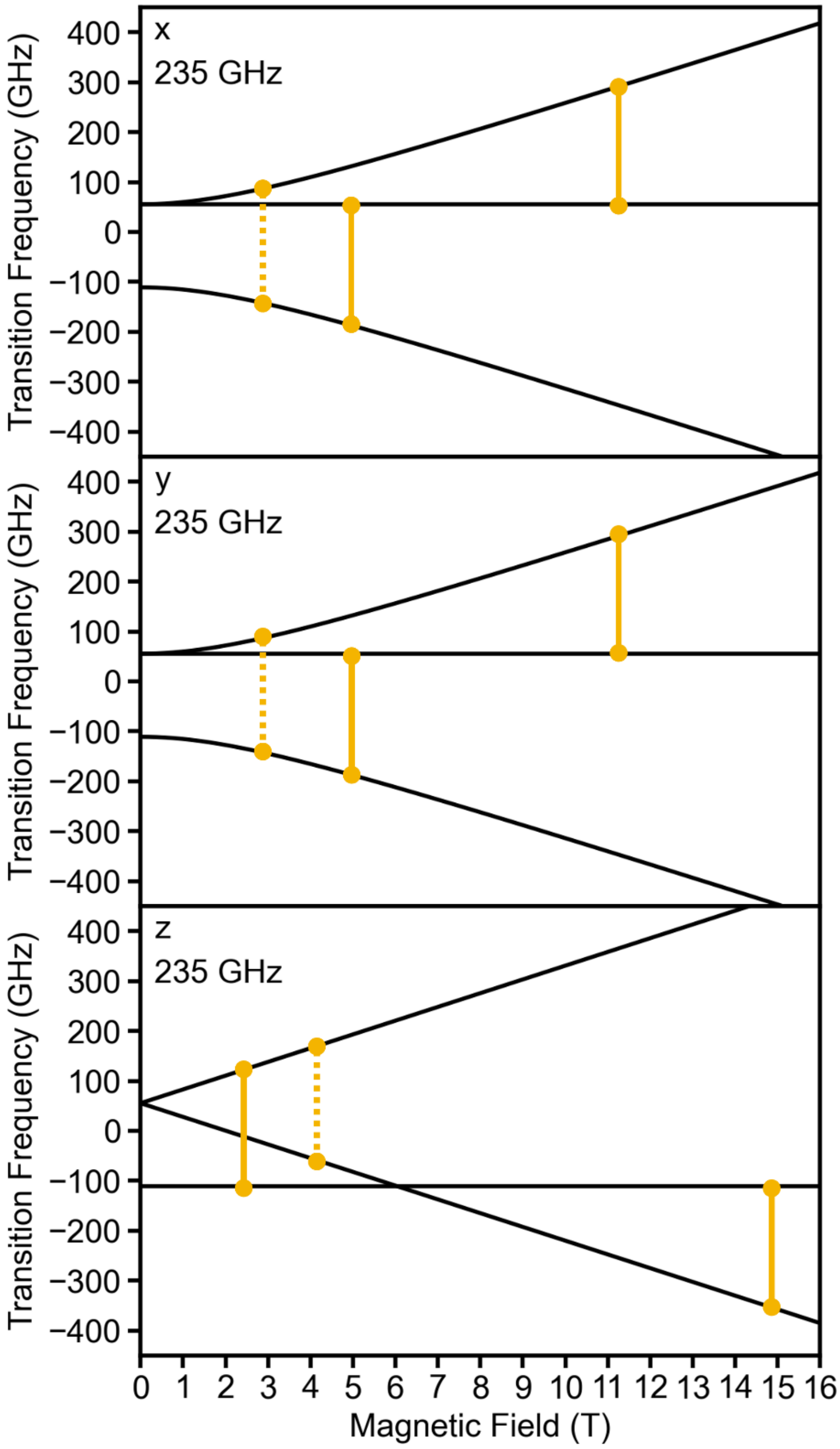

Figure S42 | Zeeman diagrams along the x, y, and z axes for **2** based on the spin Hamiltonian parameters in Table S21. The solid yellow lines denote the allowed ($\Delta m_s = 1$) and the dashed yellow lines denote unallowed ($\Delta m_s = 2$) transitions at 235 GHz. These Zeeman diagrams were simulated using a positive value of *D*; the higher-energy transition around 11 T for the x, y axes should diminish in intensity as temperature decreases and that state depopulates if the sign of *D* is indeed positive.

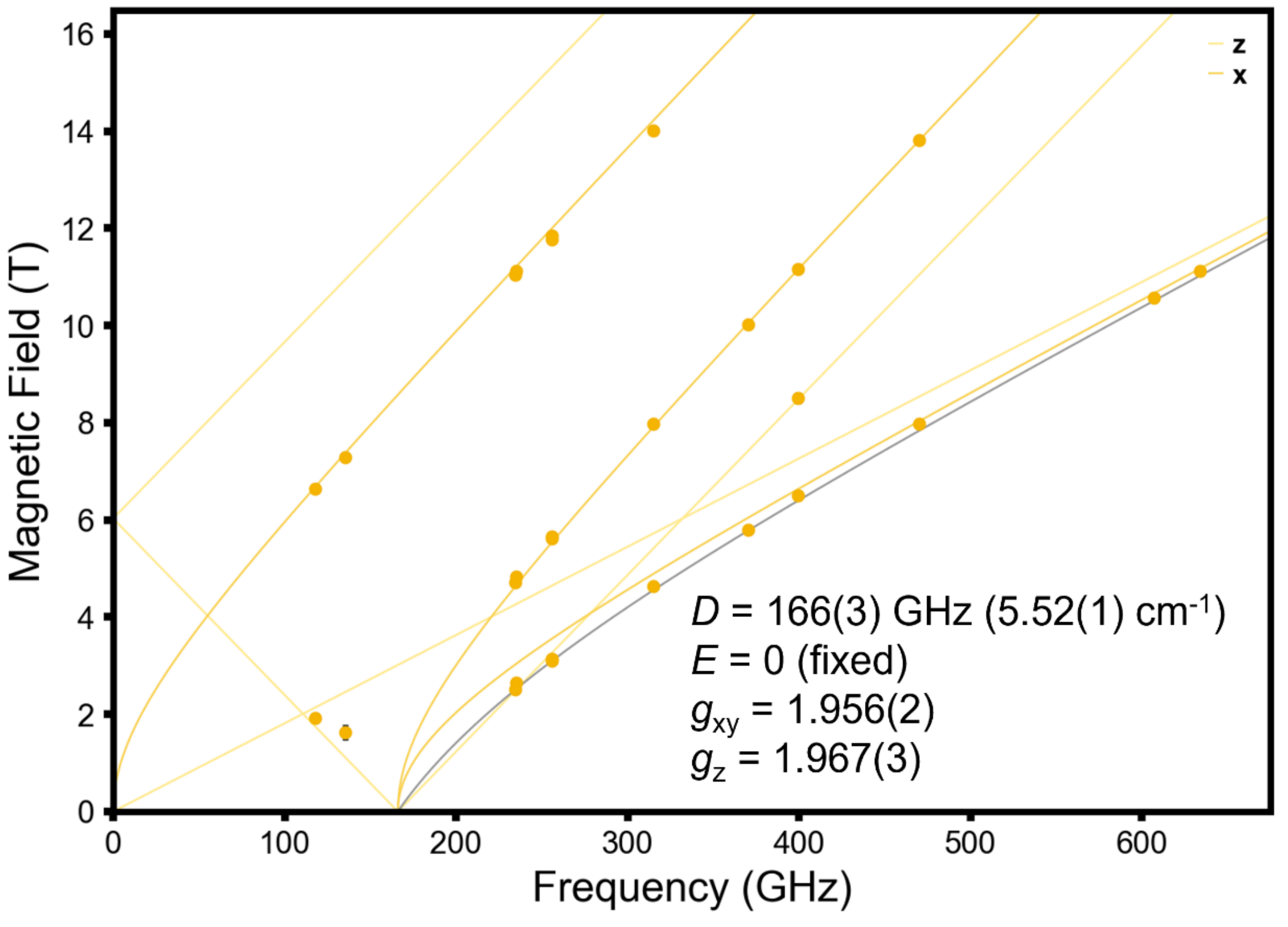

Figure S43 | Frequency dependencies of the resonance fields observed in **2** at 10 K. Yellow datapoints are features from experimental spectra. The light yellow and dark yellow lines are transitions simulated along the molecular orientations along the x and z axes using the spin Hamiltonian parameters in Table S21. The gray line represents the "half-field" transition, which is an off-axial turning point, and generated from the resonance fields read out of the simulated powder spectra.

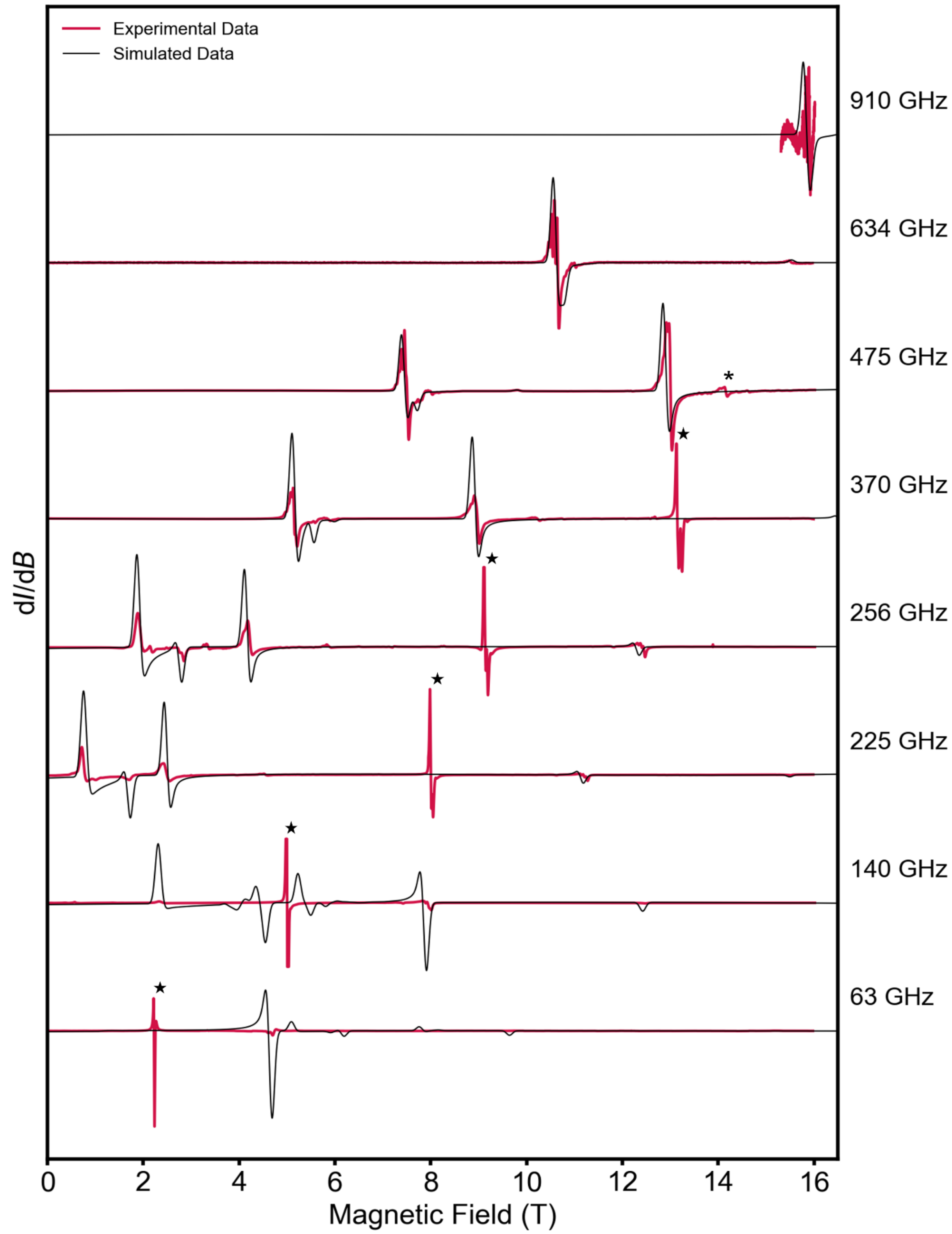

Figure S44 | Variable-frequency HFHF cw-EPR spectra for **3** at 10 K using spin Hamiltonian parameters from Table S21**.** Stars denote Cr^{3+} impurities, asterisks denote byproduct **2**, pink lines denote experimental data, and black lines denote simulated data.

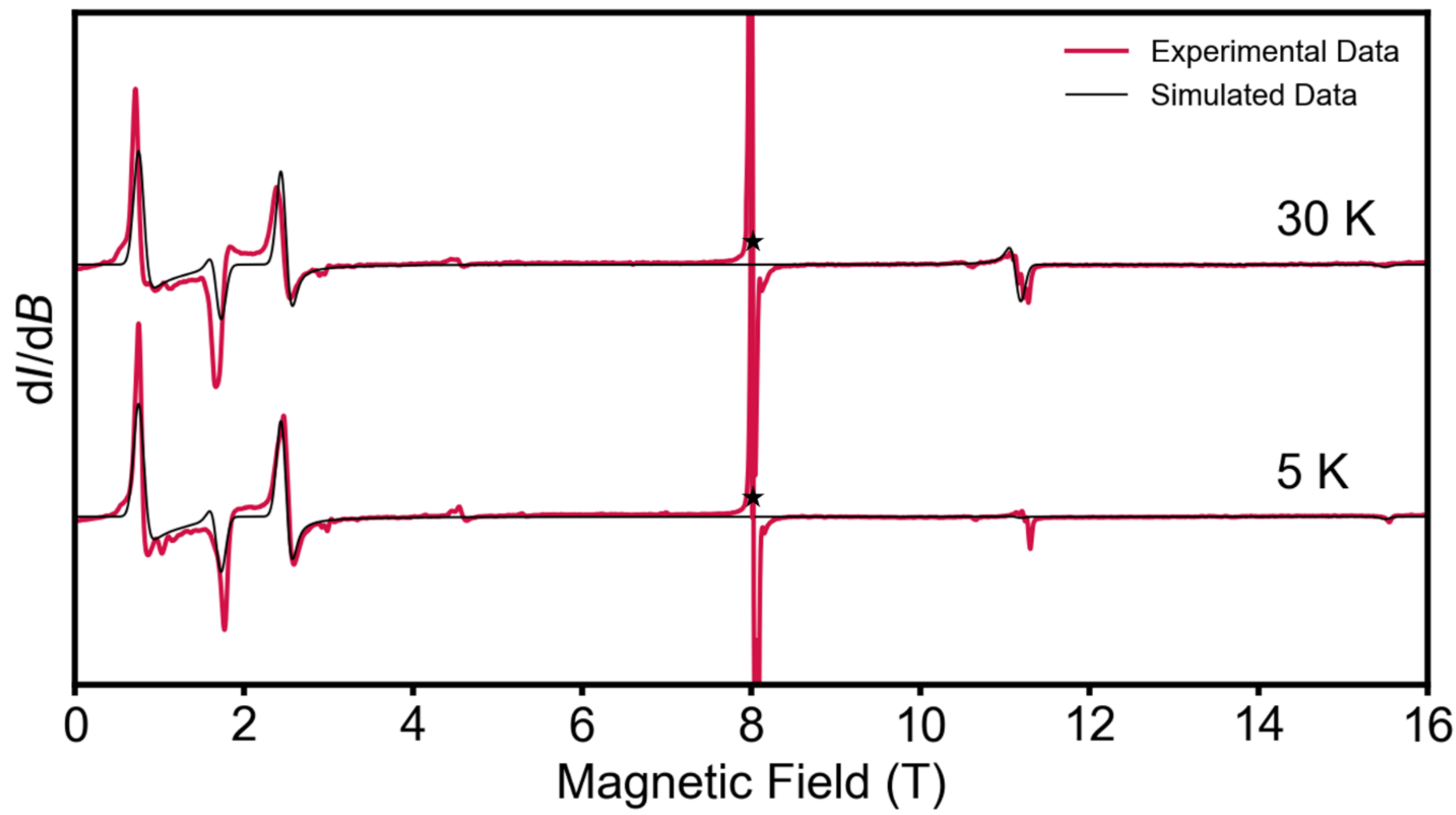

Figure S45 | Variable-temperature HFHF cw-EPR spectra for **3** at 225 GHz. Stars denote Cr^{3+} impurities, pink lines denote experimental data, and black lines denote simulated data**.**

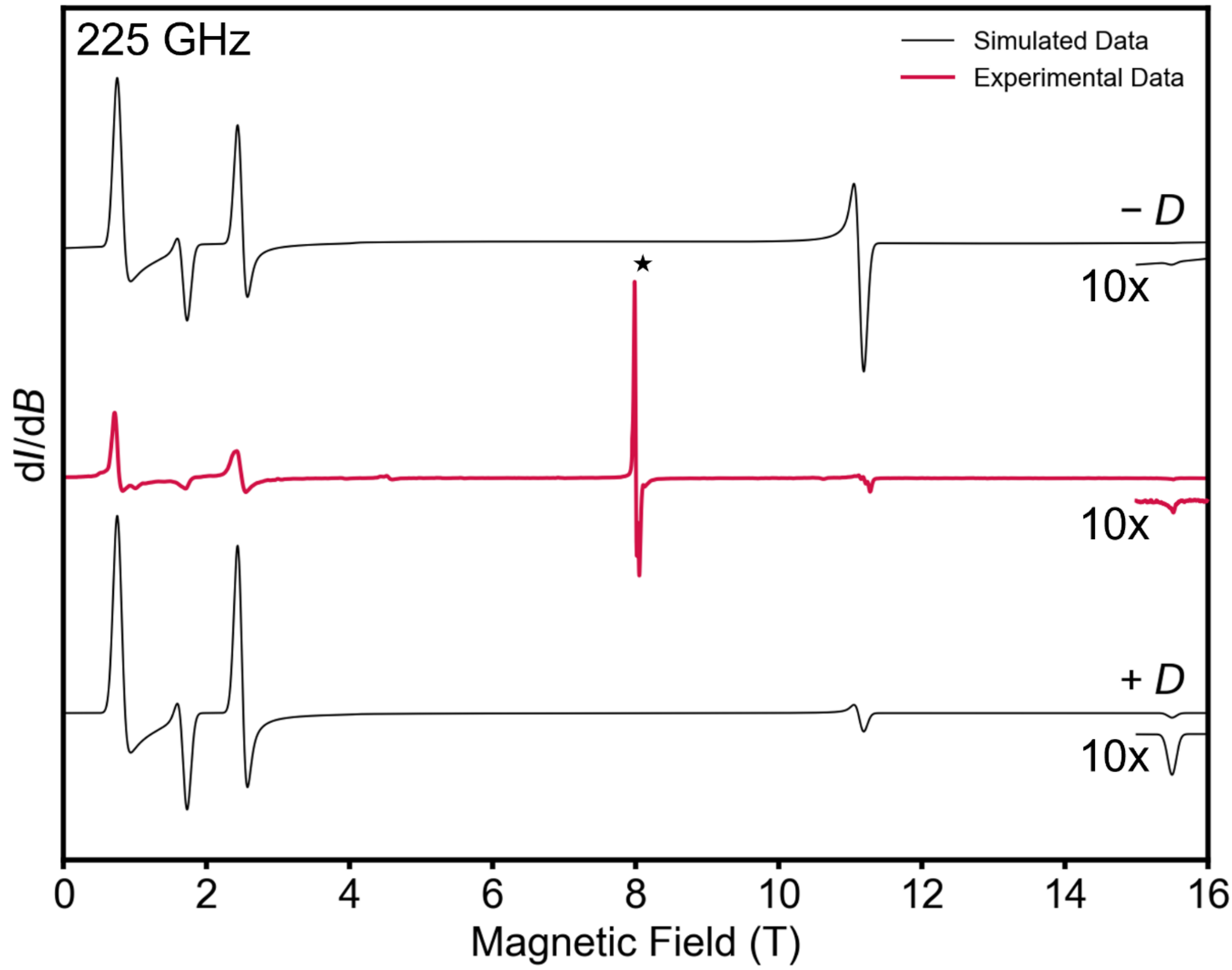

Figure S46 | Simulated EPR spectra for **3** using the spin Hamiltonian parameters in Table S21, a temperature of 10 K, and either a negative (top) or positive (bottom) value of *D***.** The experimental spectrum best agrees with the simulated EPR spectrum using a positive value of *D*. Stars denote Cr^{3+} impurities, pink lines denote experimental data, and black lines denote simulated data.

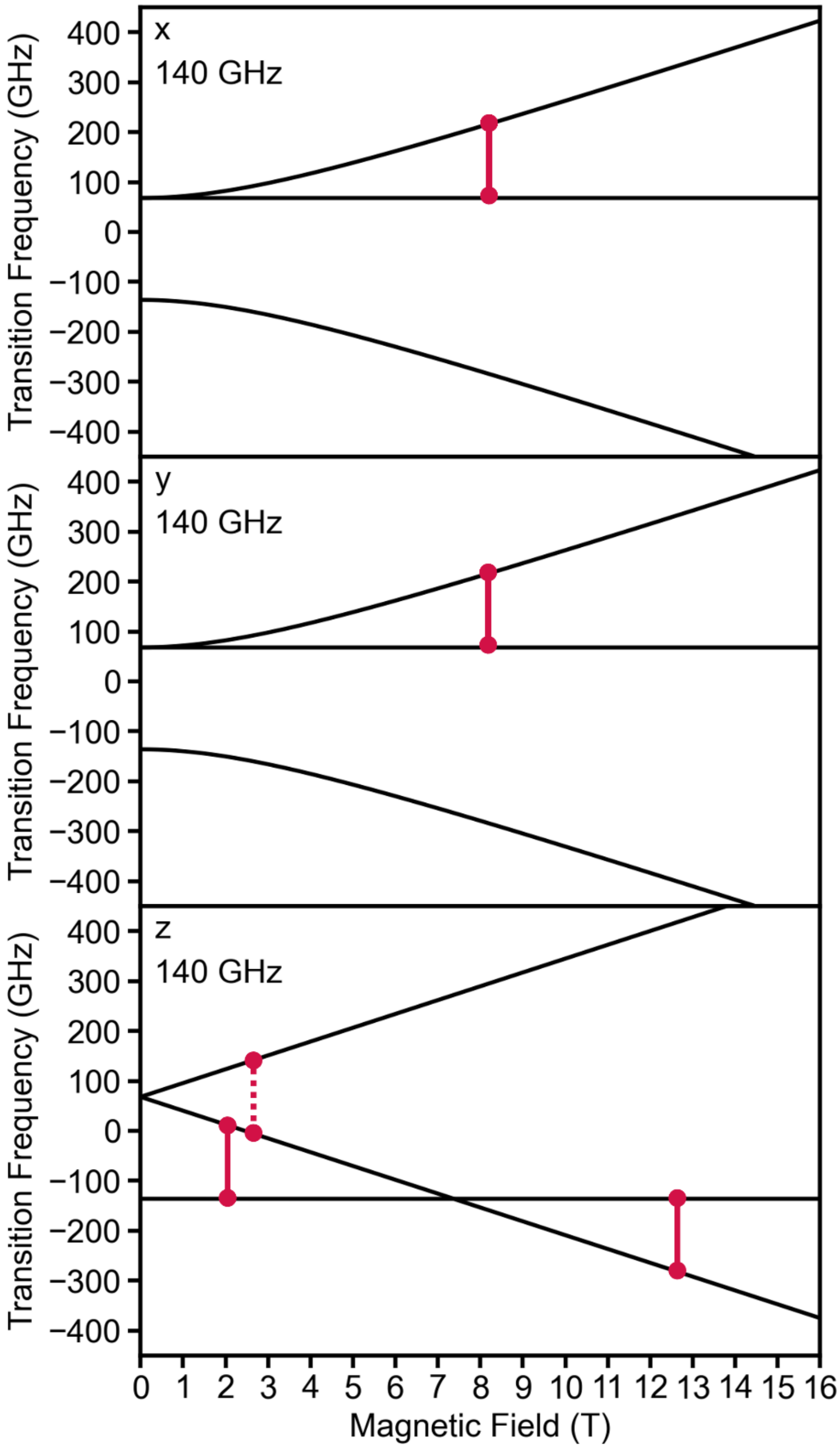

Figure S47 | Zeeman diagrams along the x, y, and z axes for **3** based on the spin Hamiltonian parameters in Table S21. The solid pink lines denote the allowed ($\Delta m_s = 1$) and the dashed pink lines denote the forbidden ($\Delta m_s = 2$) transitions at 140 GHz. These Zeeman diagrams were simulated using a positive value of D; the lower-energy transition around 2.5 T for the x, y axes should increase in intensity as temperature decreases and that state populates if the sign of D is indeed positive.

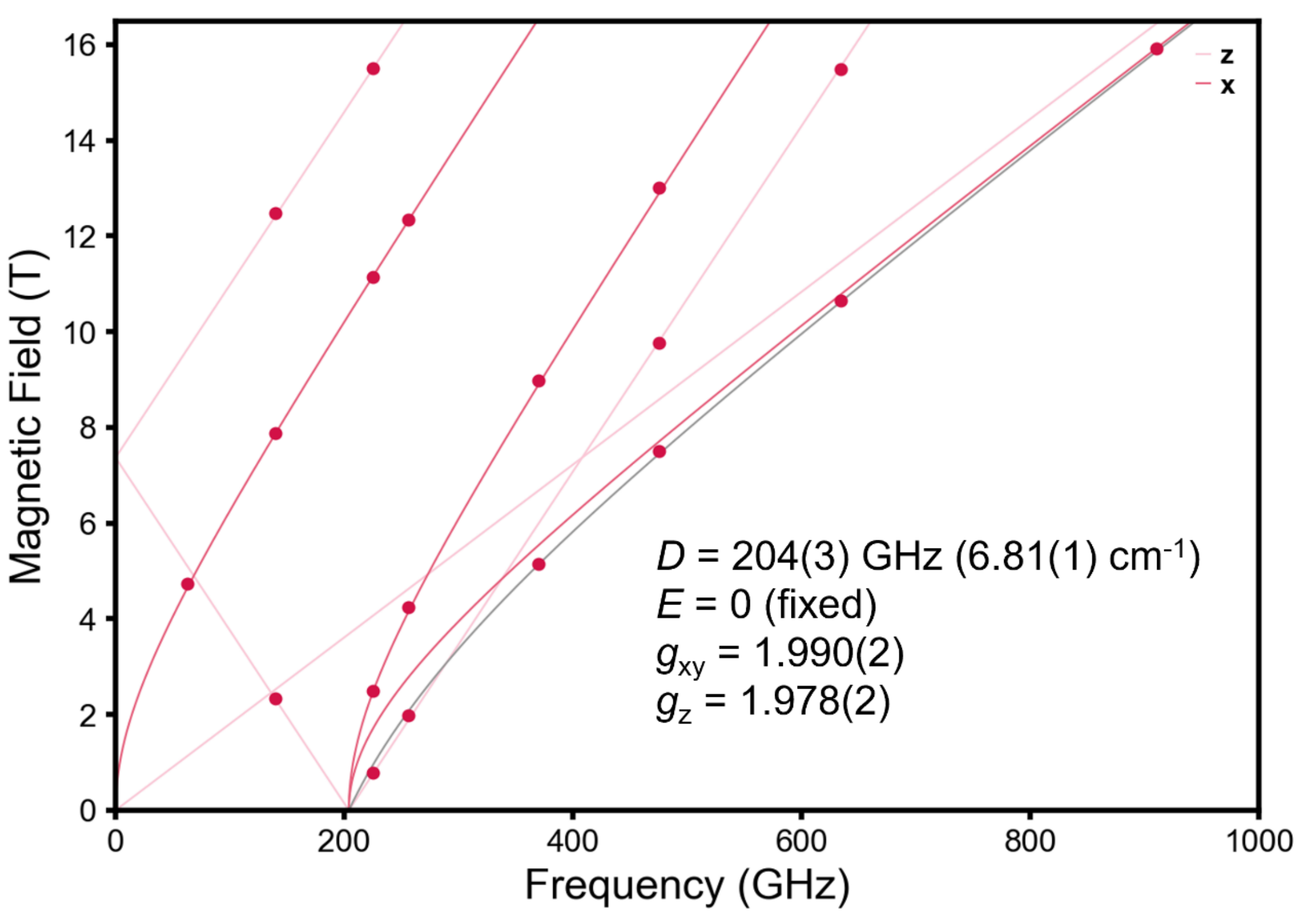

Figure S48 | Frequency dependencies of the resonance fields observed in **3** at 10 K. Pink datapoints are features from experimental spectra. The light pink and dark pink lines are transitions simulated along the molecular orientations along the x and z axes using the spin Hamiltonian parameters in Table S21. The gray line represents the "half-field" transition, which is an off-axial turning point, and generated from the resonance fields read out of the simulated powder spectra.

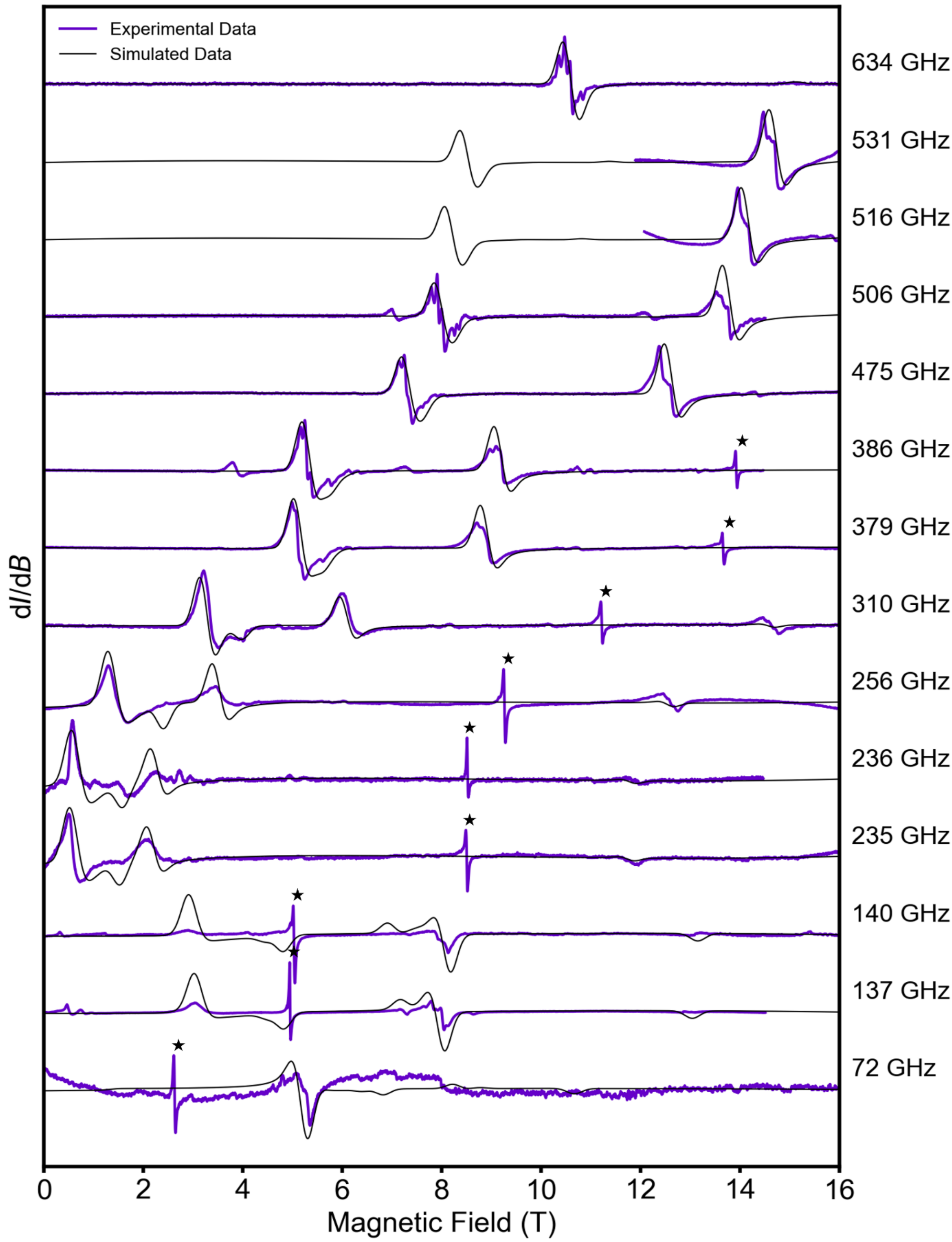

Figure S49 | Variable-frequency HFHF cw-EPR spectra for **4** at 10 K using spin Hamiltonian parameters from Table S21**.** Stars denote Cr^{3+} impurities, purple lines denote experimental data, and black lines denote simulated data.

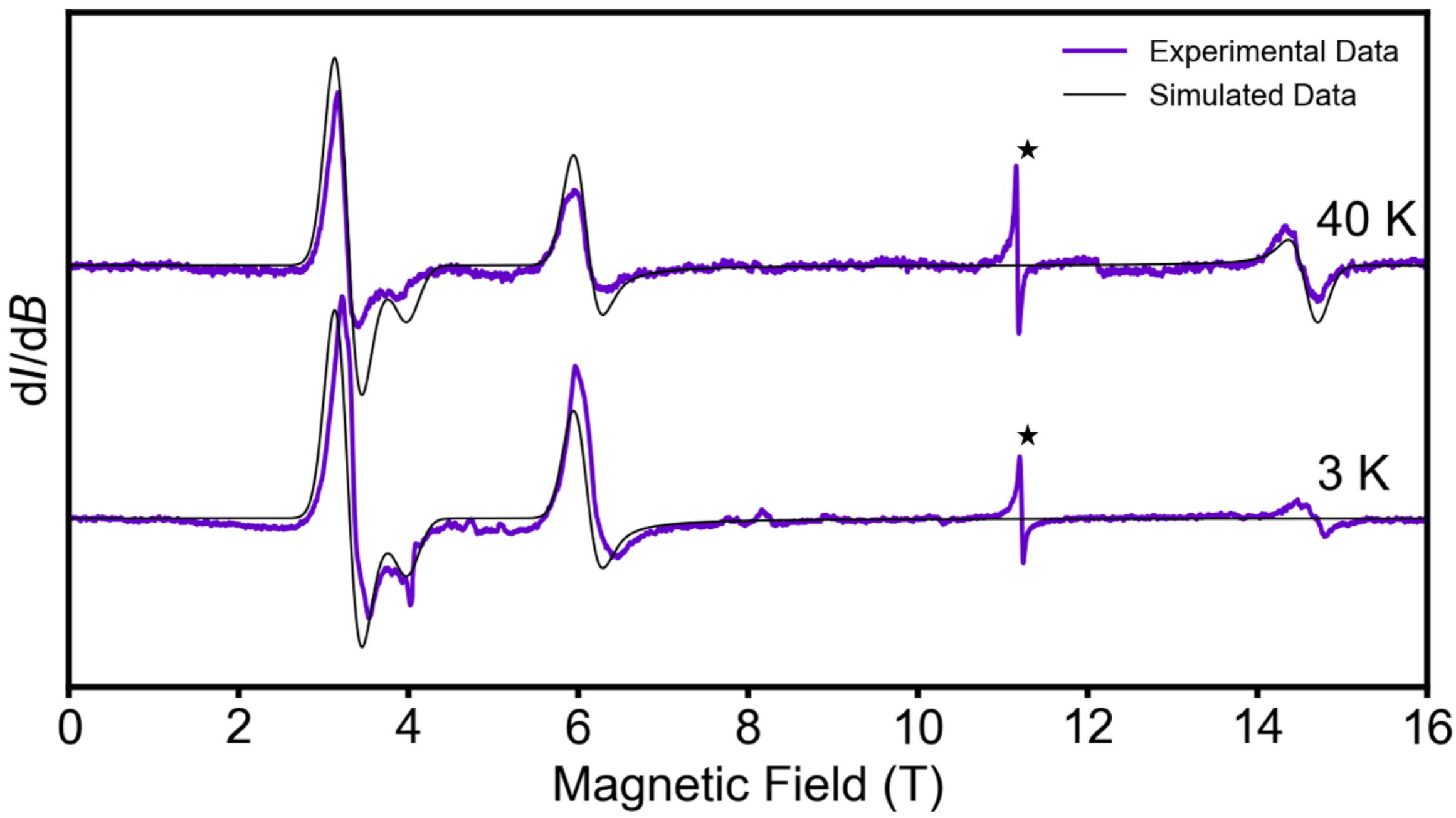

Figure S50 | Variable-temperature HFHF cw-EPR spectra for **4** at 310 GHz. Stars denote Cr^{3+} impurities, purple lines denote experimental data, and black lines denote simulated data**.**

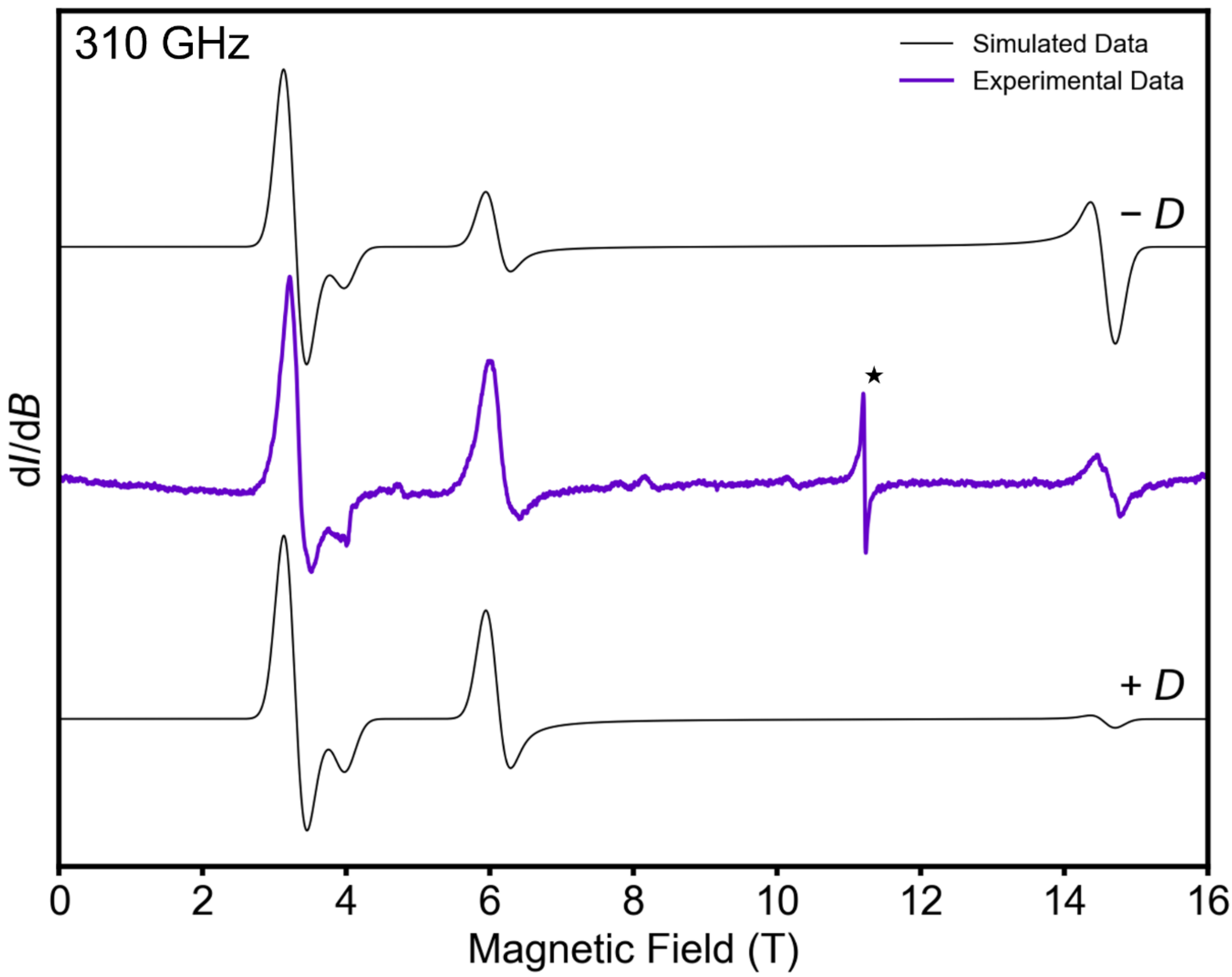

Figure S51 | Simulated EPR spectra for **4** using the spin Hamiltonian parameters in Table S21, a temperature of 10 K, and either a negative (top) or positive (bottom) value of *D***.** The experimental spectrum best agrees with the simulated EPR spectrum using a positive value of *D*. Stars denote Cr^{3+} impurities, purple lines denote experimental data, and black lines denote simulated data.

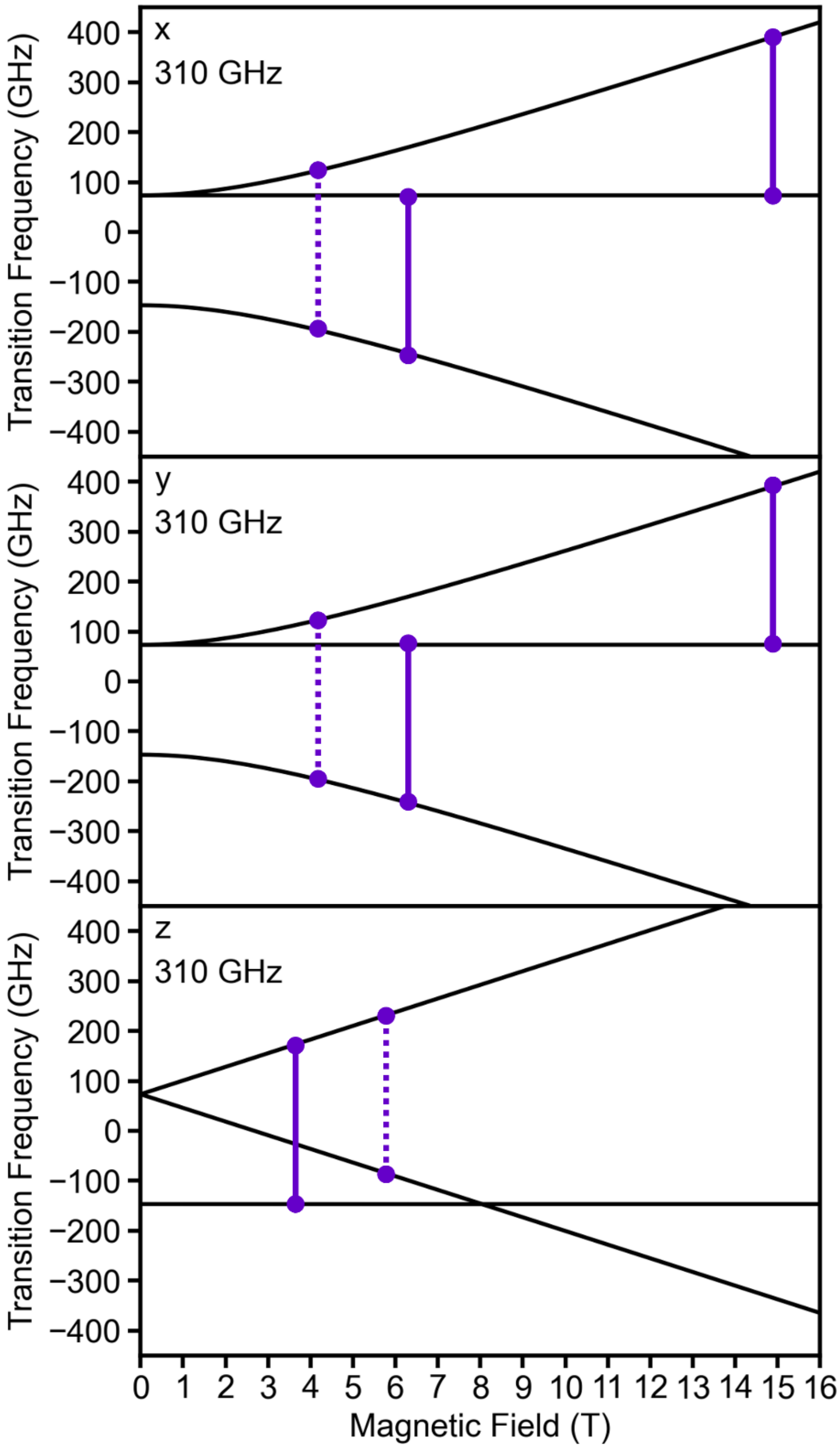

Figure S52 | Zeeman diagrams along the x, y, and z axes for **4** based on the spin Hamiltonian parameters in Table S21. The solid purple lines denote the allowed ($\Delta m_s = 1$) and the dashed purple lines denote the forbidden ($\Delta m_s = 2$) transitions at 310 GHz. These Zeeman diagrams were simulated using a positive value of *D*; the higher-energy transition around 14.5 T for the x, y axes should diminish in intensity as temperature decreases and that state depopulates if the sign of *D* is indeed positive.

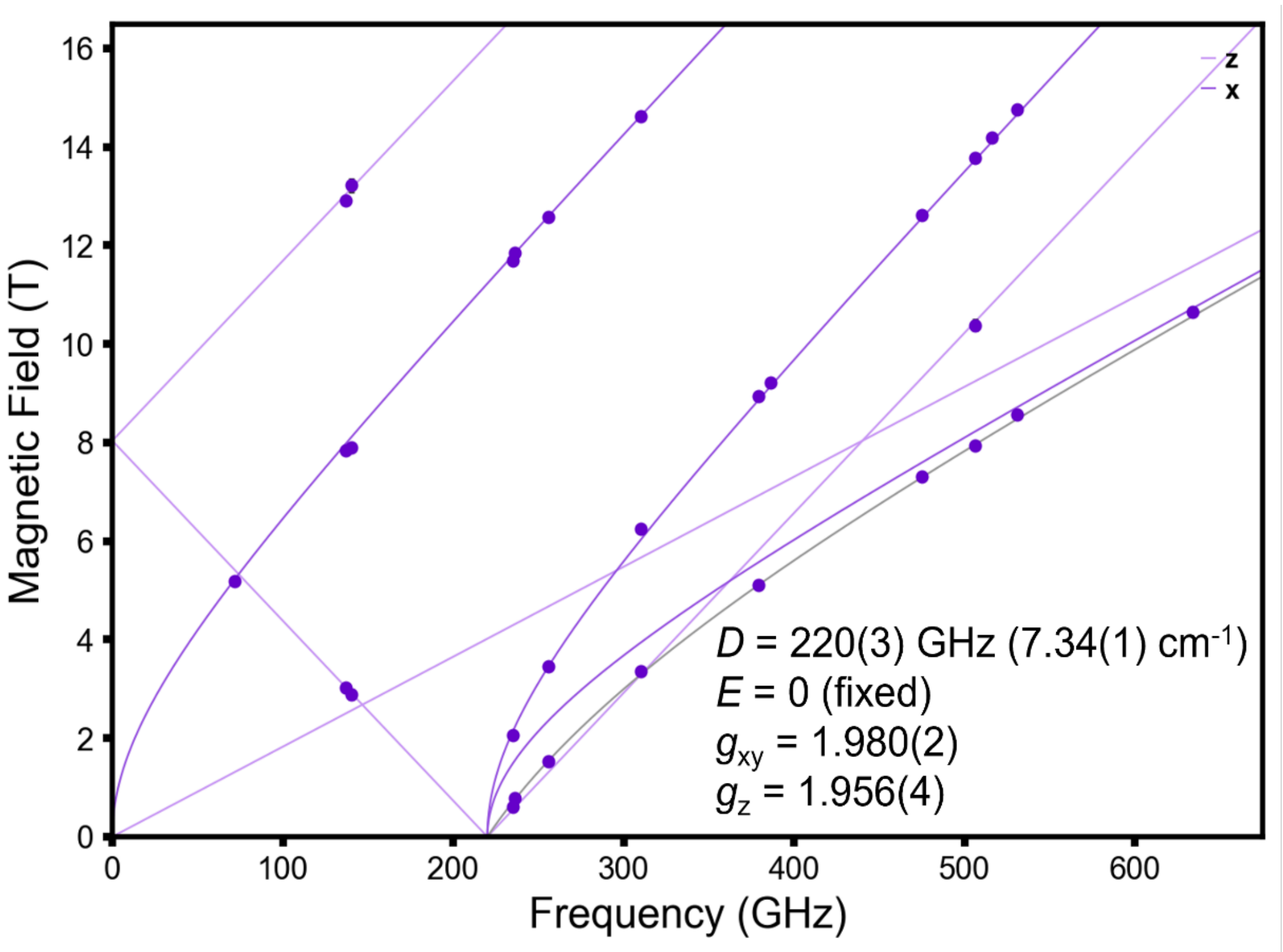

Figure S53 | Frequency dependencies of the resonance fields observed in **4** at 10 K. Purple datapoints are features from experimental spectra. The light purple and dark purple lines are transitions simulated along the molecular orientations along the x and z axes using the spin Hamiltonian parameters in Table S21. The gray line represents the "half-field" transition, which is an off-axial turning point, and generated from the resonance fields read out of the simulated powder spectra.

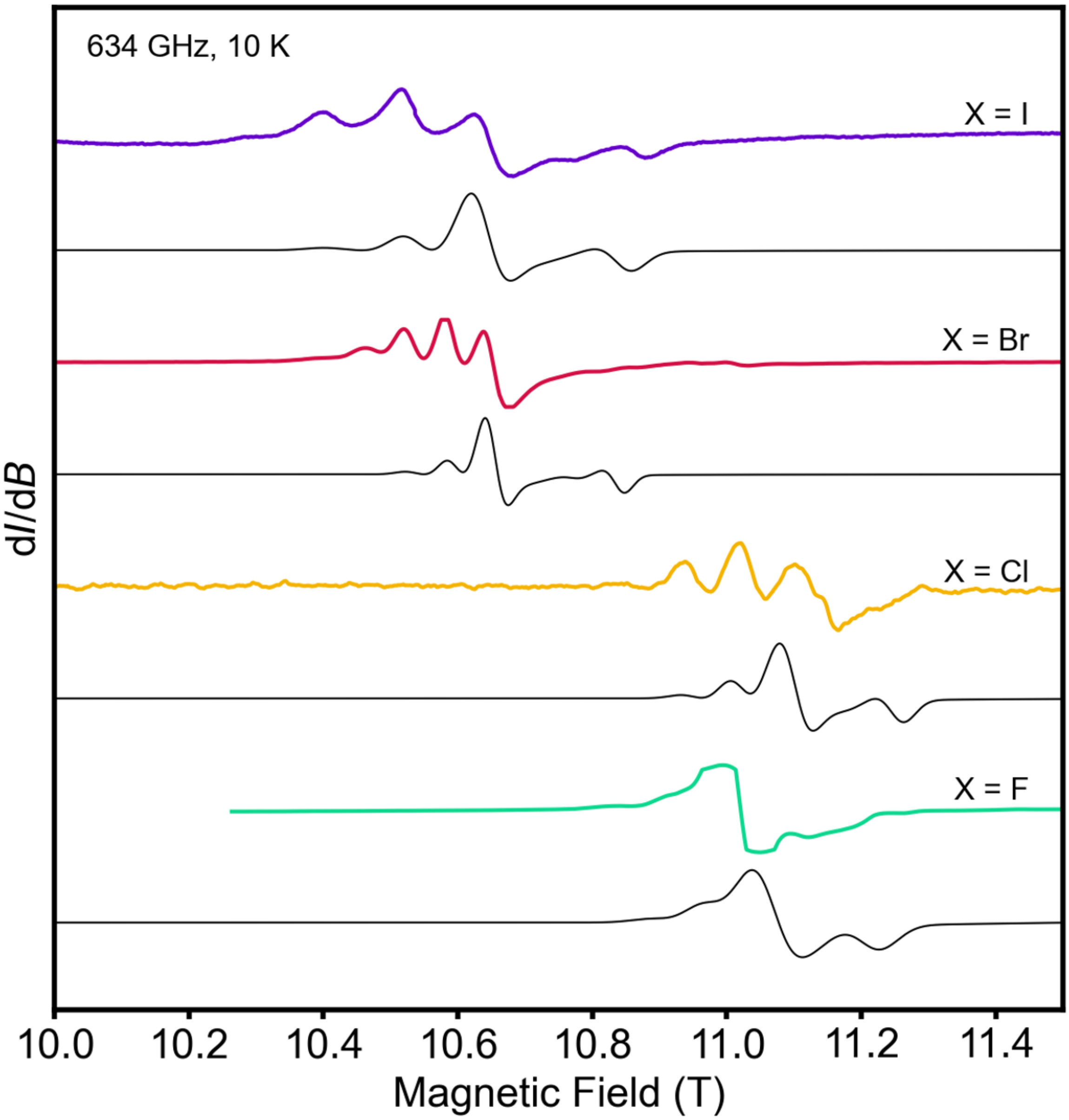

Figure S54 | Exchange coupling modeled in the forbidden ($\Delta m_s = 2$) half-field transitions of complexes **1**–**4** using the spin Hamiltonian parameters in Table S21 and Table S22. We see field-dependent splitting in many features of **1**–**4** at high field, and is particularly evident in the half-field ($\Delta m_s = 2$) transitions. We attribute this phenomenon to a weak through-space exchange interaction enabled by short Cr—Cr distances (8.0–8.3 Å) in the pure samples used for these measurements; similar interactions have been documented by HFHF cw-EPR spectroscopy.[36]

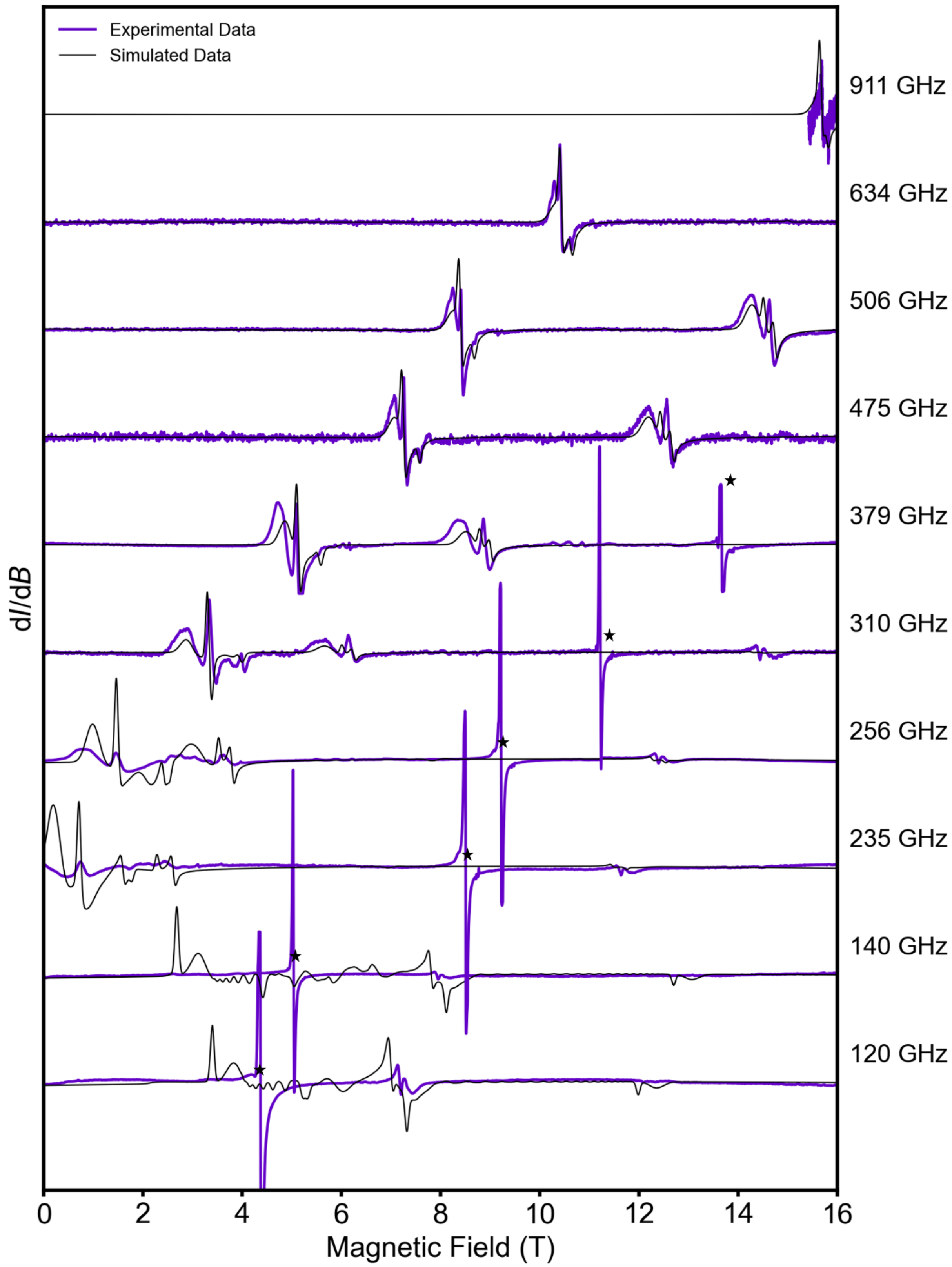

Figure S55 | Variable-frequency HFHF cw-EPR spectra for **4′** at 10 K using spin Hamiltonian parameters from Table S21**.** Stars denote Cr^{3+} impurities, purple lines denote experimental data, and black lines denote simulated data.

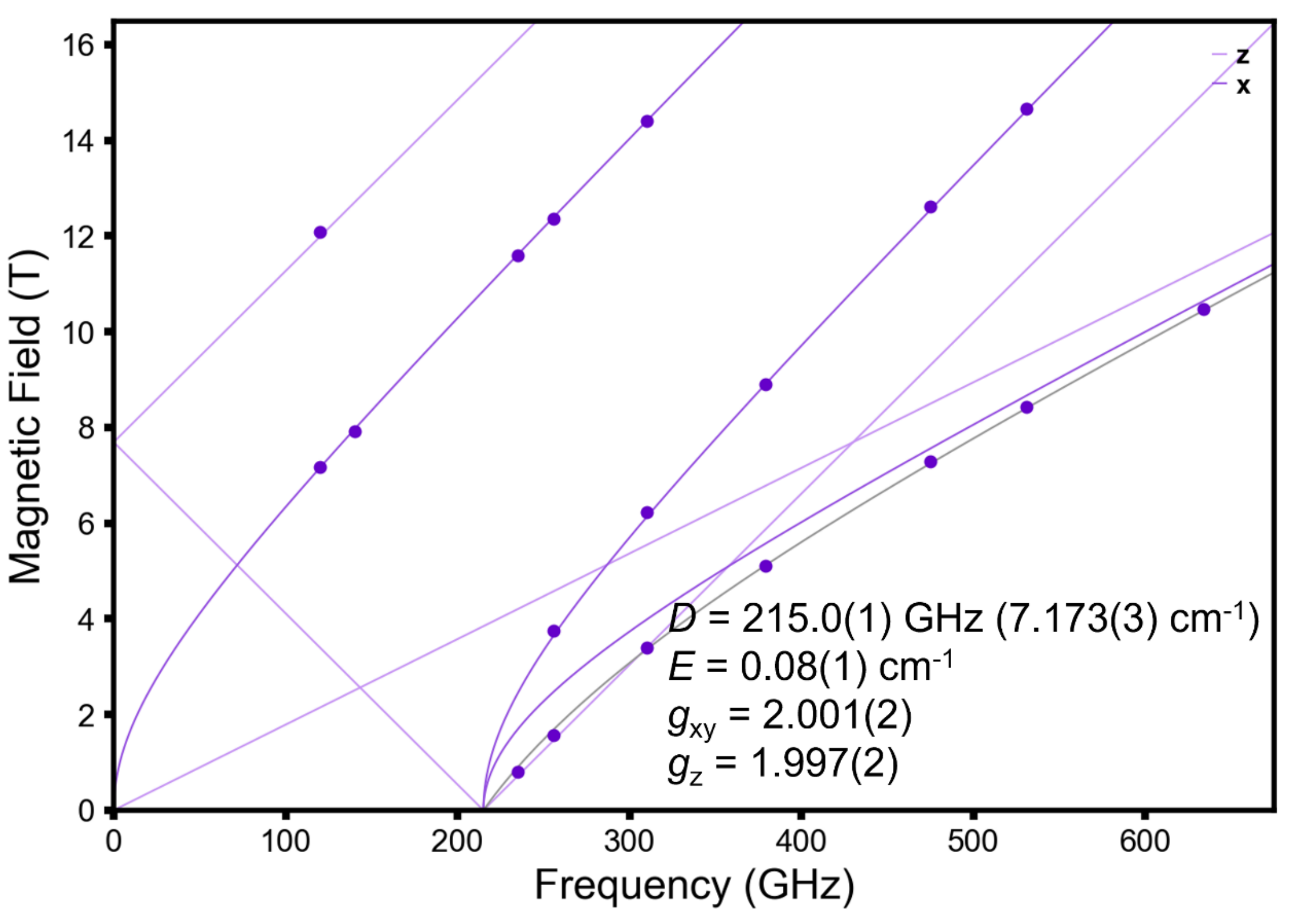

Figure S56 | Frequency dependencies of the resonance fields observed for the isolated species in **4′** at 10 K. Purple datapoints are features from experimental spectra. The light purple and dark purple lines are transitions simulated along the molecular orientations along the x and z axes using the spin Hamiltonian parameters in Table S21. The gray line represents the “half-field” transition, which is an off-axial turning point, and generated from the resonance fields read out of the simulated powder spectra.

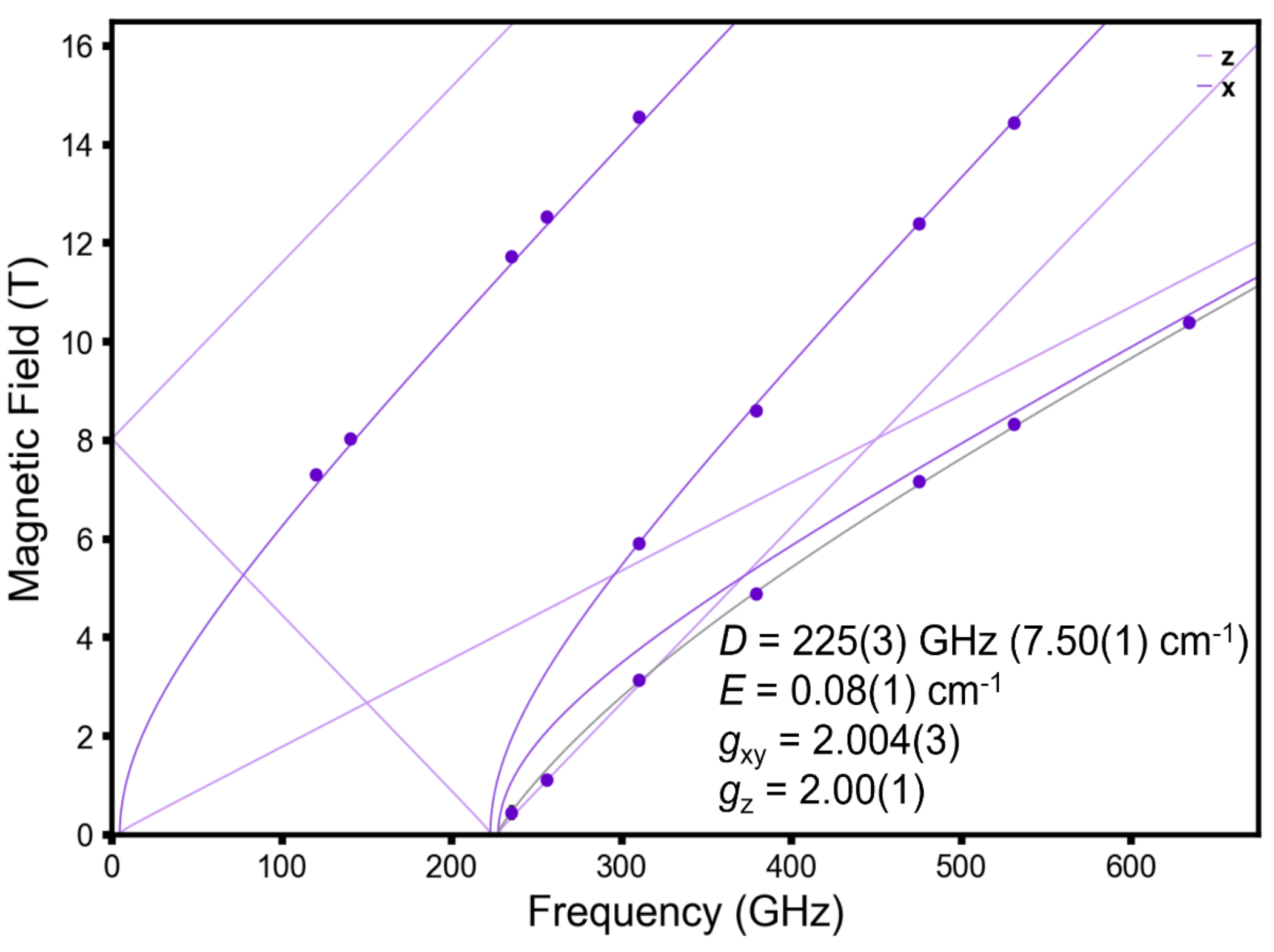

Figure S57 | Frequency dependencies of the resonance fields observed for the concentrated species in **4′** at 10 K. Purple datapoints are features from experimental spectra. The light purple and dark purple lines are transitions simulated along the molecular orientations along the x and z axes using the spin Hamiltonian parameters in Table S21. The gray line represents the "half-field" transition, which is an off-axial turning point, and generated from the resonance fields read out of the simulated powder spectra.

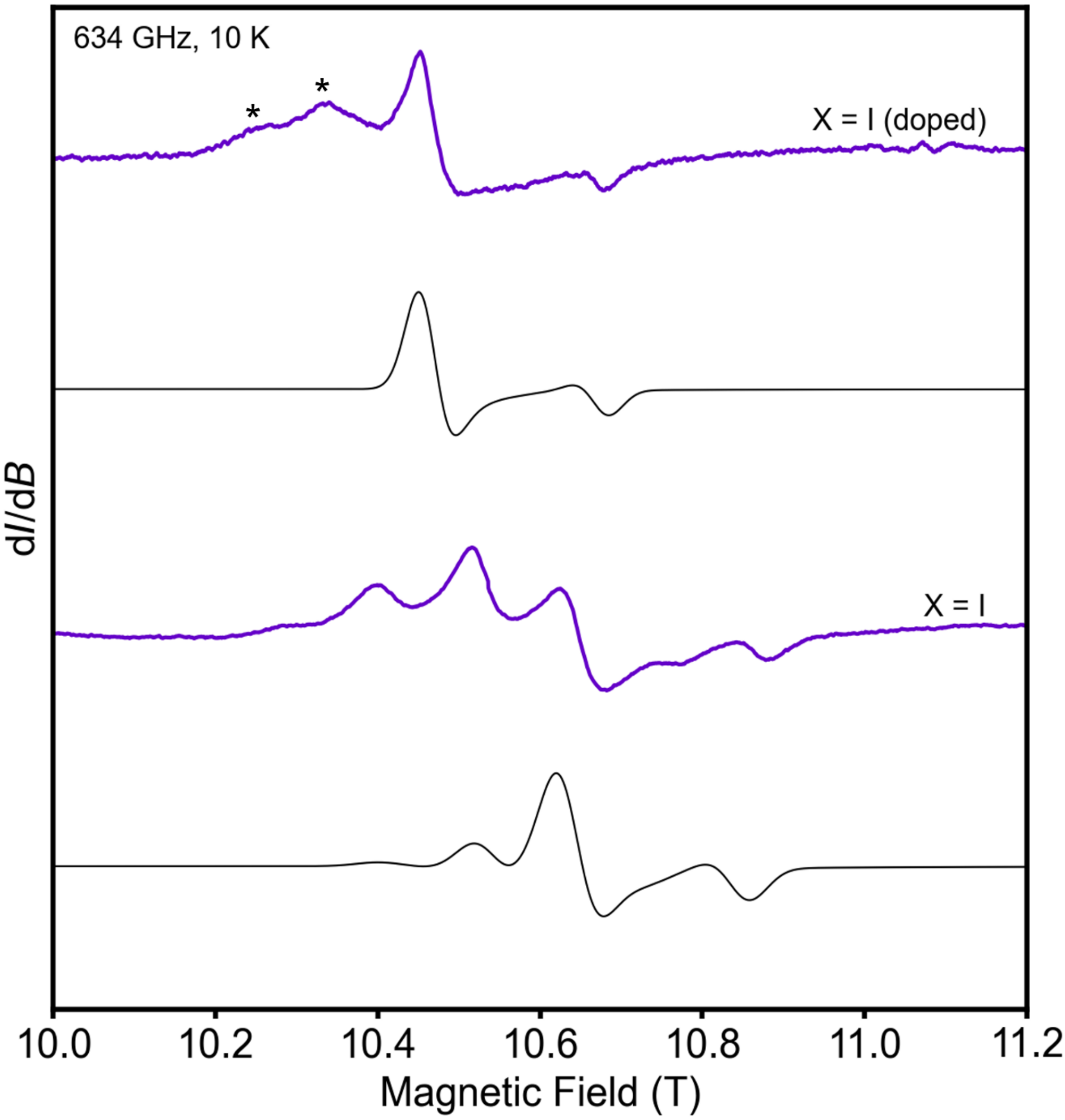

Figure S58 | Top: the forbidden ($\Delta m_s = 2$) half-field transition of **4′** using the spin Hamiltonian parameters in Table S21. Bottom: the forbidden ($\Delta m_s = 2$) half-field transition of **4** using the spin Hamiltonian parameters in Table S21 and Table S22. Asterisks denote remaining high spin concentrations within **4′,** purple lines denote experimental data, and black lines denote simulated data.

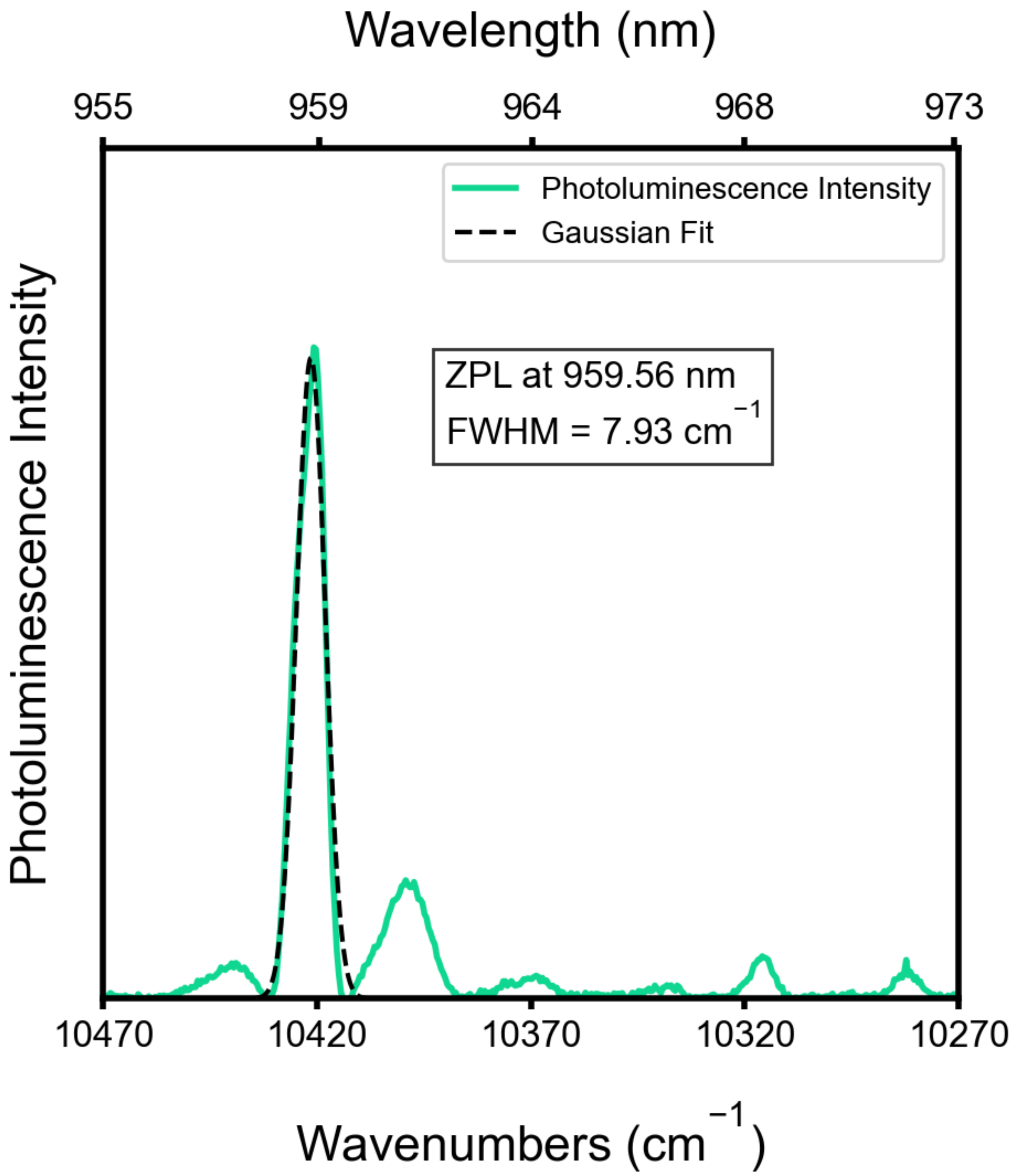

Figure S59 | PL collected on ZPL of **1′** with a 300-second exposure time, 15 mW of 660 nm excitation, a slit width of 50 µm, and a grating density of 1200 g/mm at 2.5 K.

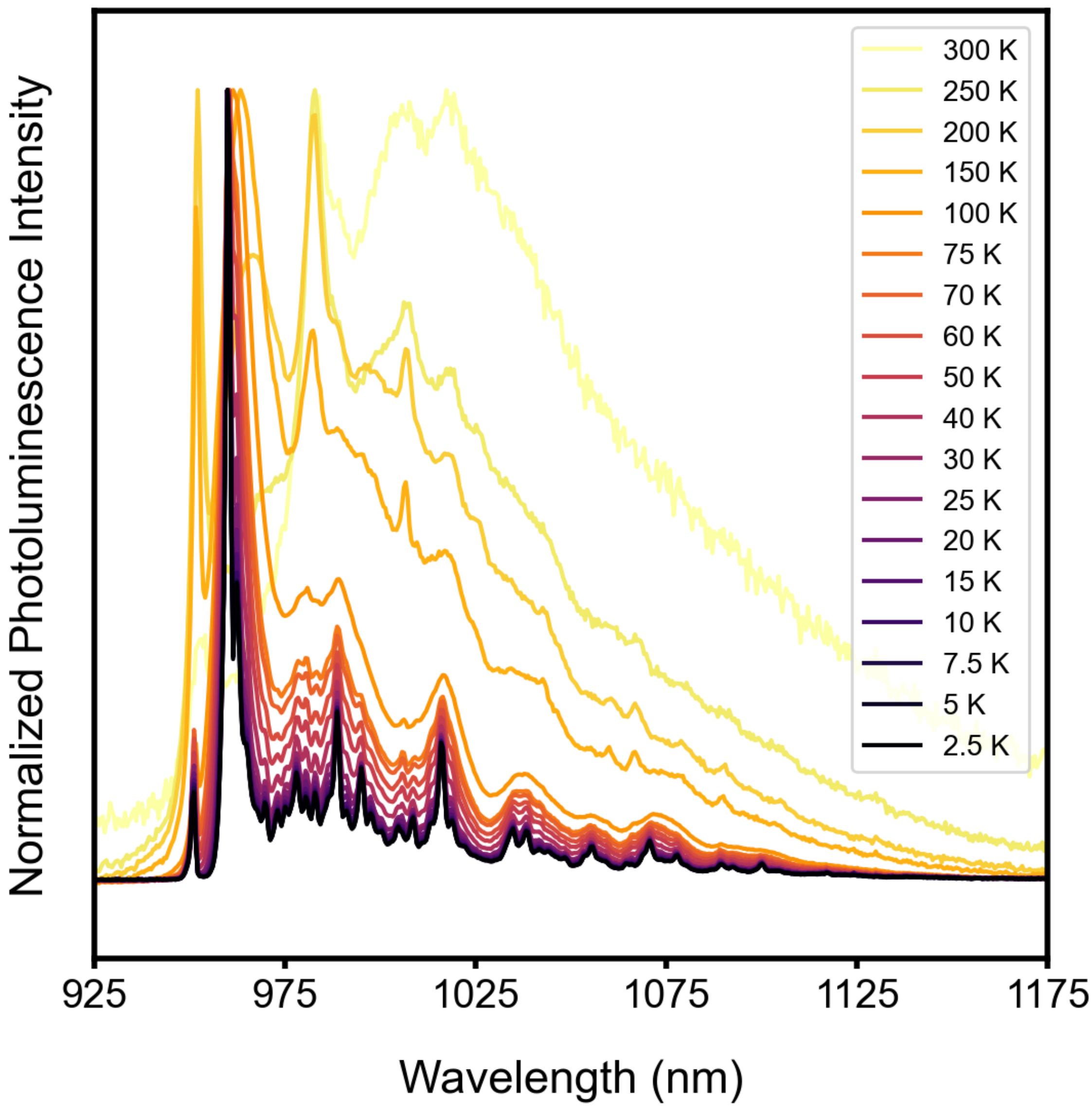

Figure S60 | Variable-temperature PL collected on **1′** with 30-second exposure time, 4 mW of 660 nm excitation, a slit width of 50 μm, and a grating density of 150 g/mm.

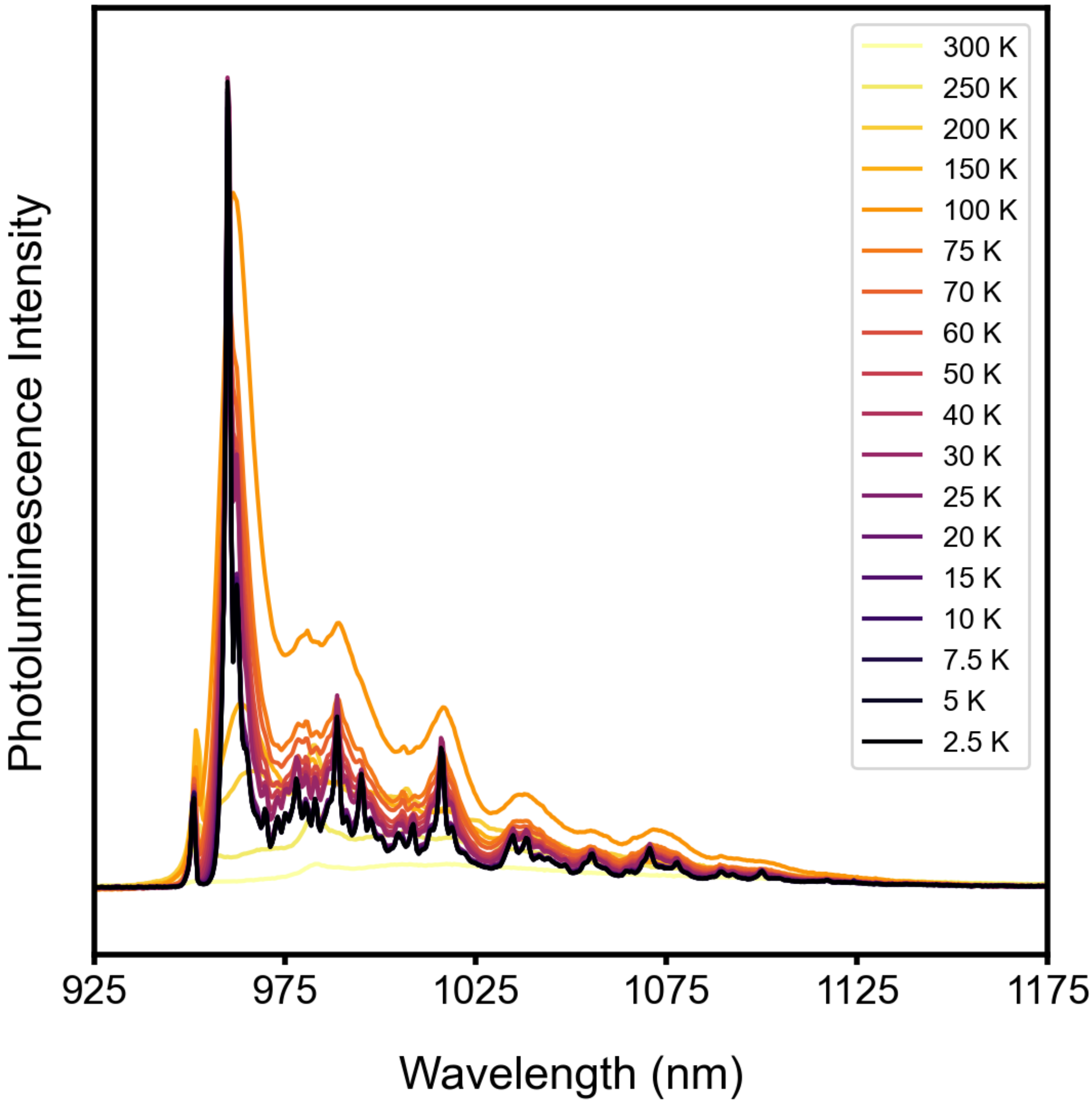

Figure S61 | Variable-temperature PL collected on **1′** with 30-second exposure time, 4 mW of 660 nm excitation, a slit width of 50 μm, and a grating density of 150 g/mm.

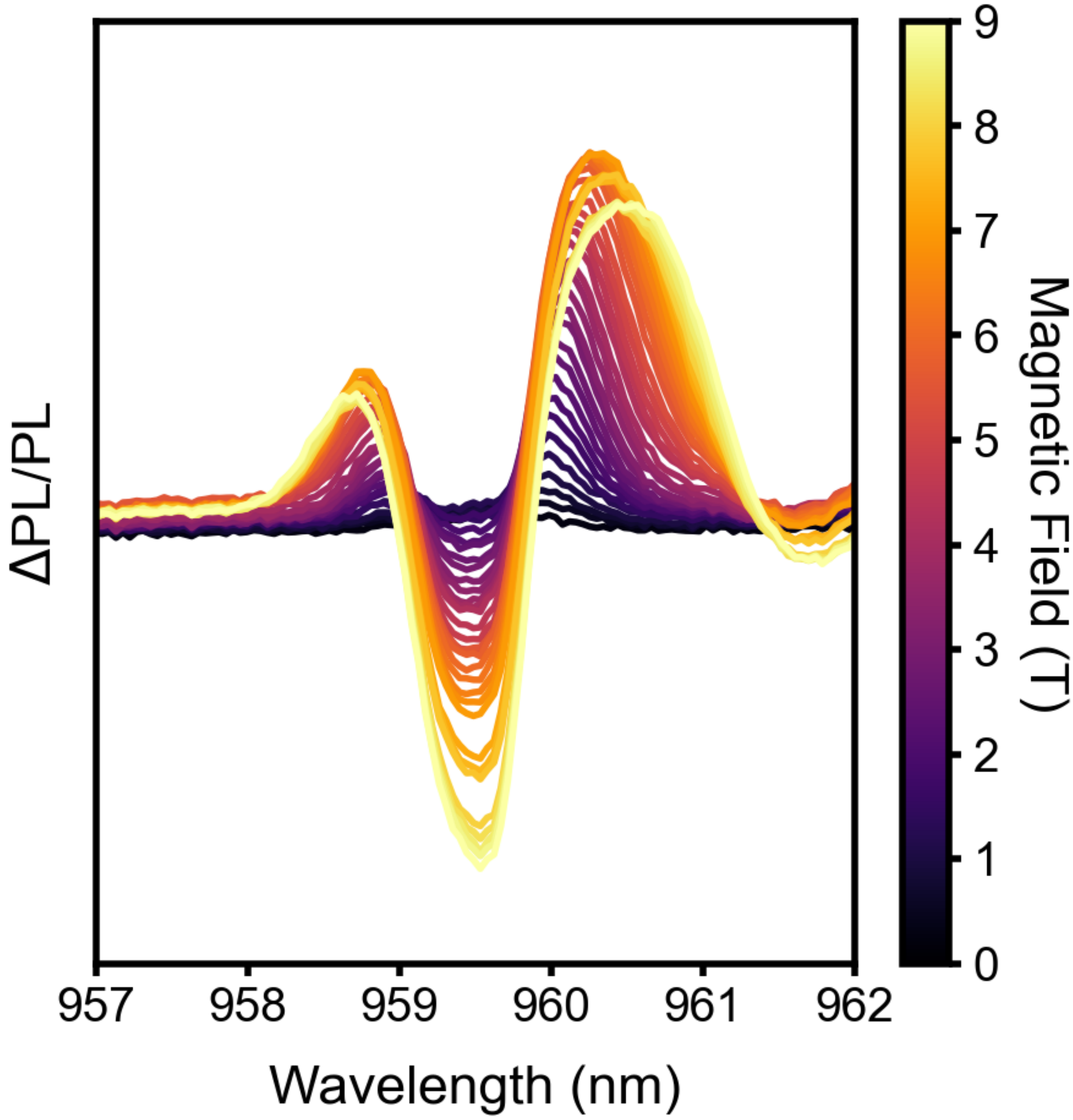

Figure S62 | Variable-field differential PL collected on **1′** with 300-second exposure time,15 mW of 660 nm excitation, a slit width of 50 μm, and a grating density of 1200 g/mm at 2.5 K.

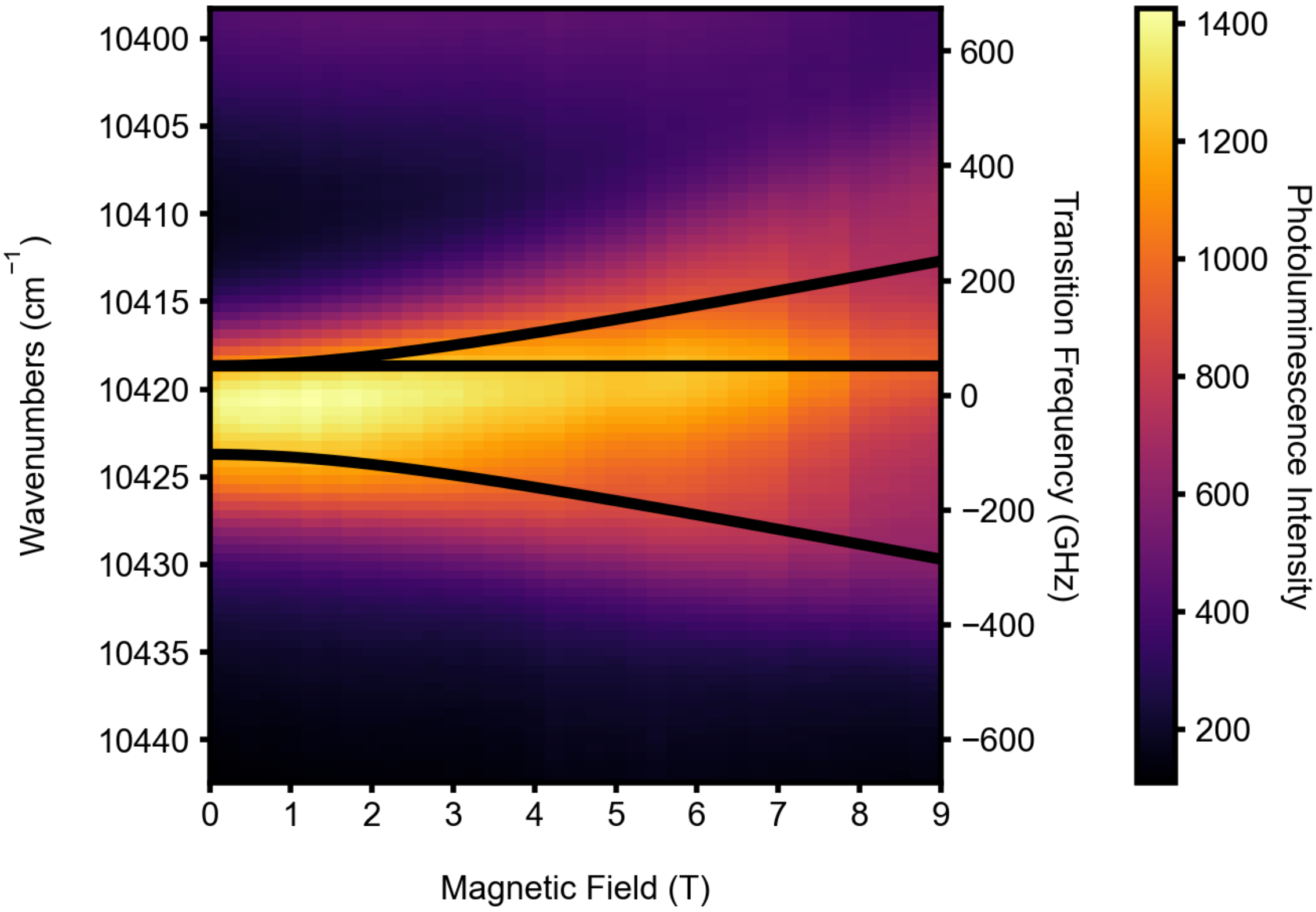

Figure S63 | Variable-field PL collected on ZPL of **1′** with 300-second exposure time, 15 mW of 660 nm excitation, a slit width of 50 μm, and a grating density of 1200 g/mm at 2.5 K. Zeeman diagram was modeled with D = +5.14 cm^{-1}, g_{iso} = 1.977, and θ = 90˚.

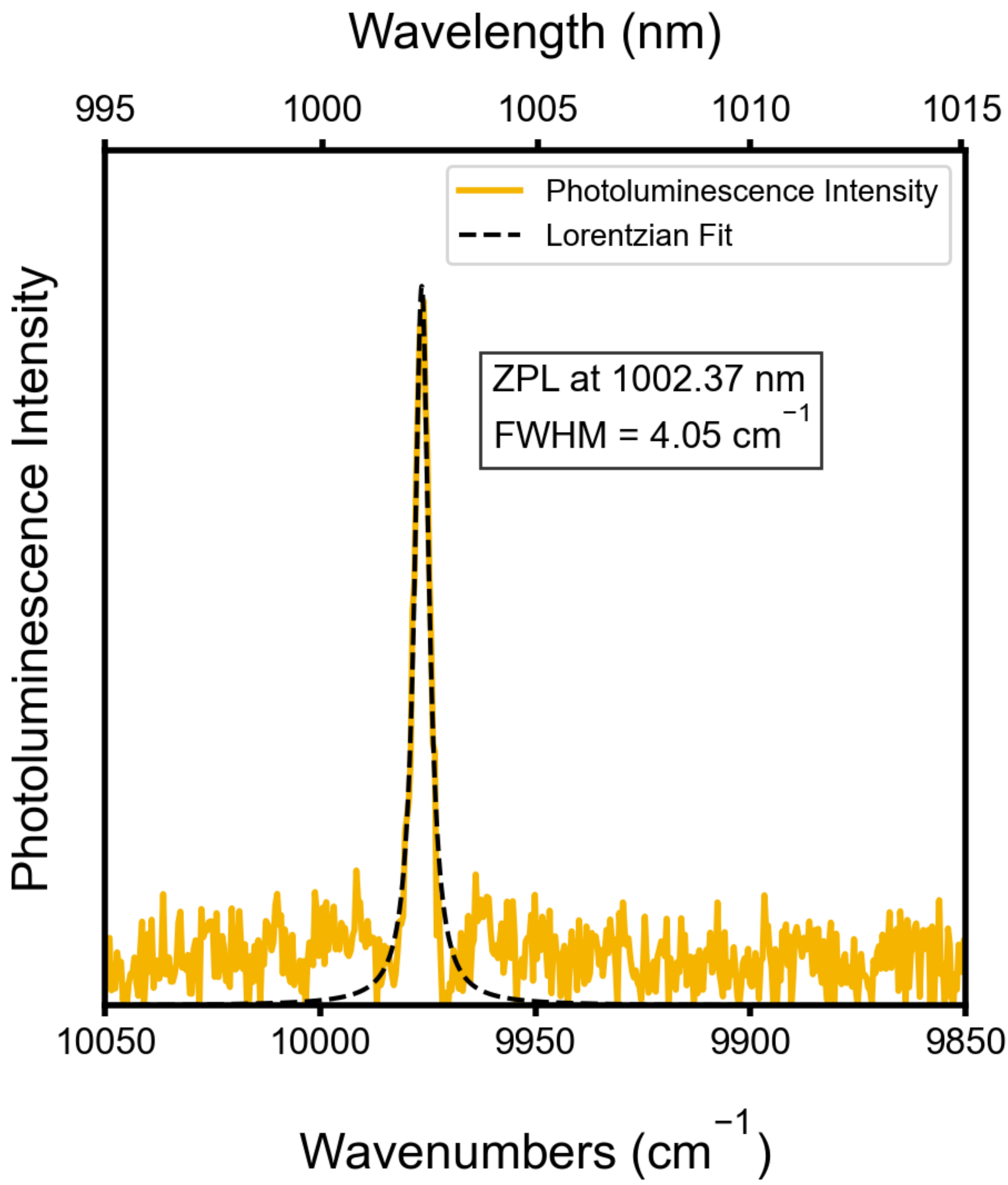

Figure S64 | PL collected on ZPL of **2′** with 60-second exposure time, 10 mW of 660 nm excitation, a slit width of 50 µm, and a grating density of 600 g/mm at 1.8 K.

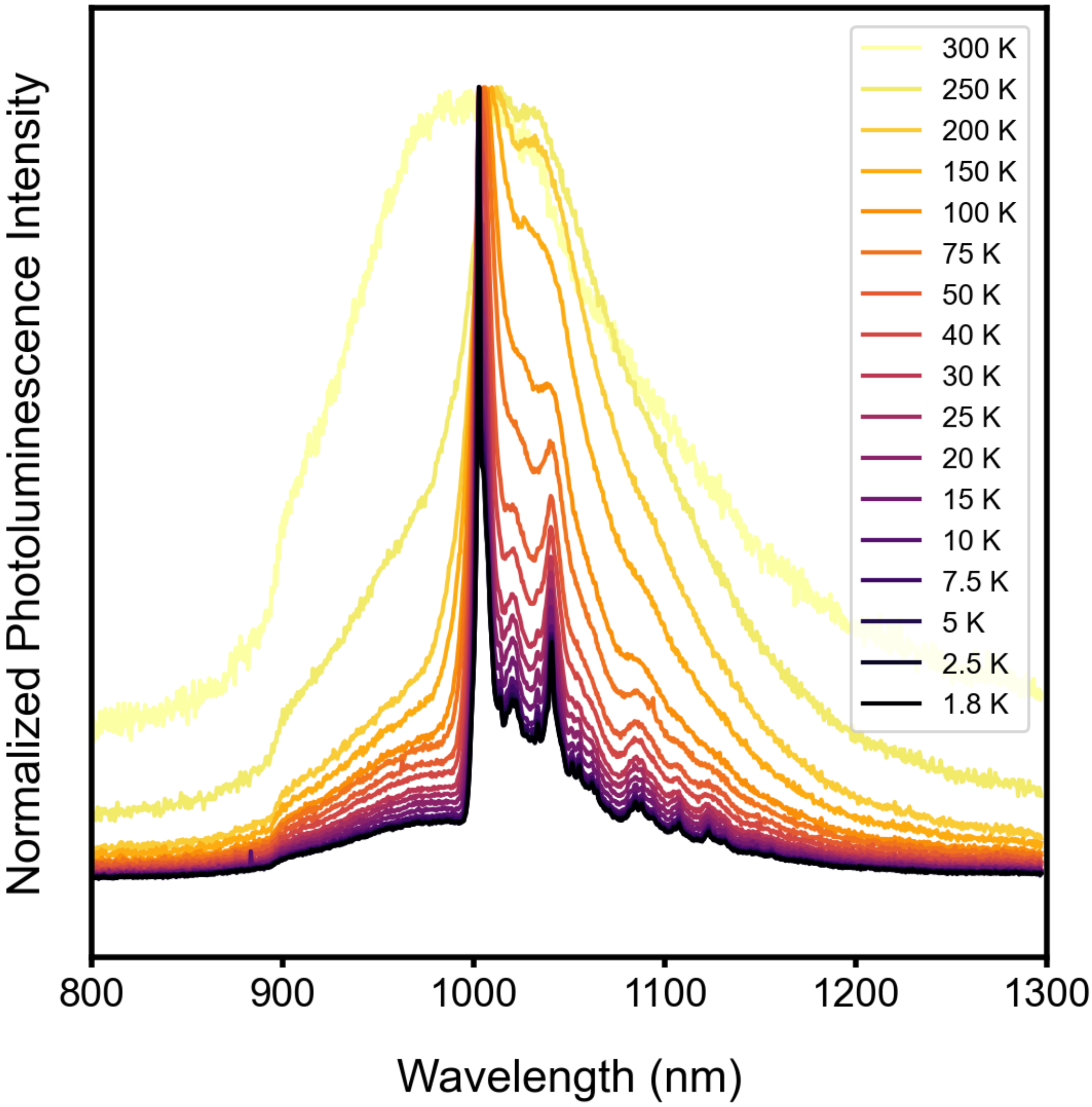

Figure S65 | Variable-temperature PL collected on **2′** with 60-second exposure time, 10 mW of 660 nm excitation, a slit width of 50 µm, and a grating density of 150 g/mm.

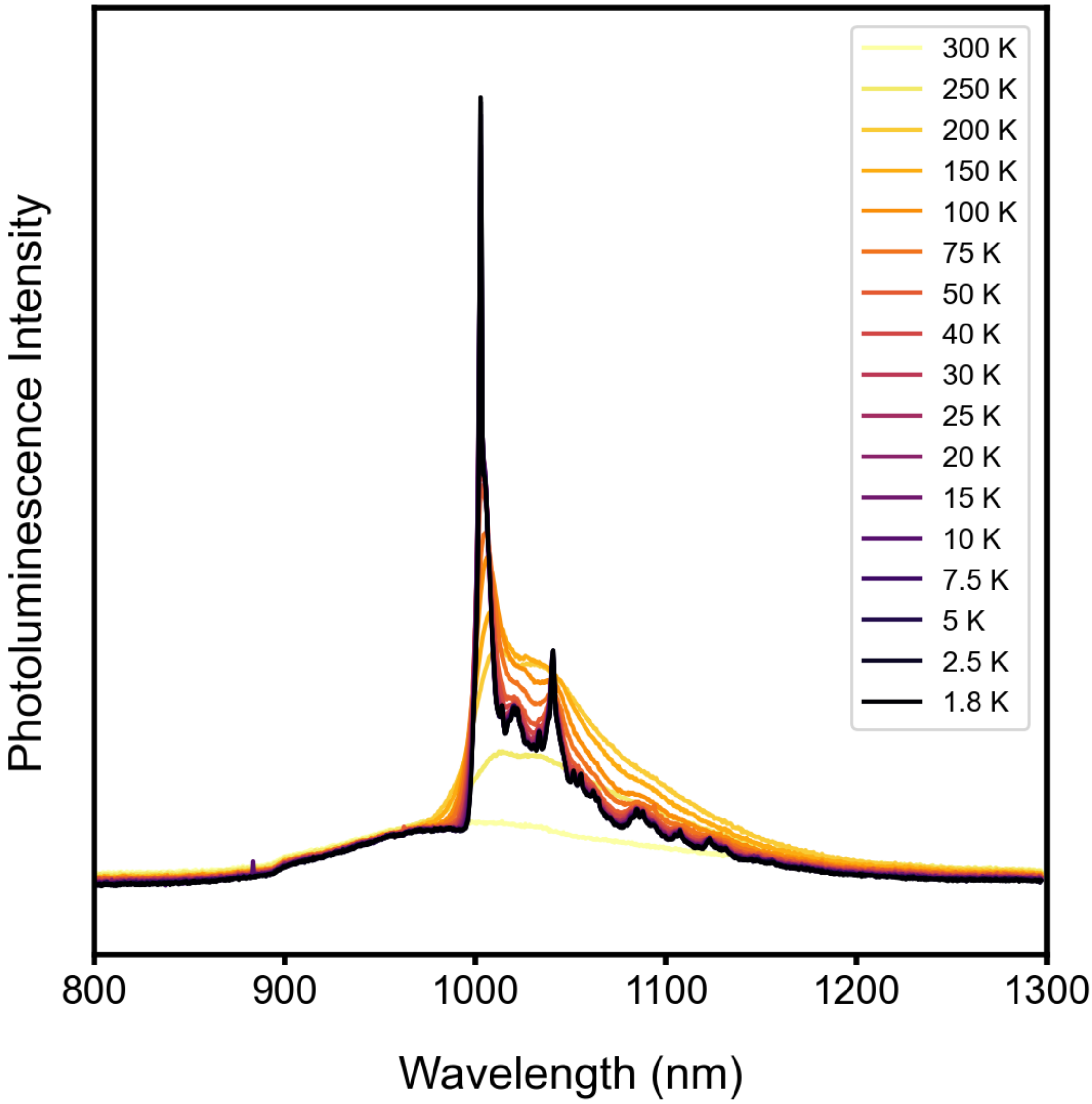

Figure S66 | Variable-temperature PL collected on **2′** with 60-second exposure time, 10 mW of 660 nm excitation, a slit width of 50 μm, and a grating density of 150 g/mm.

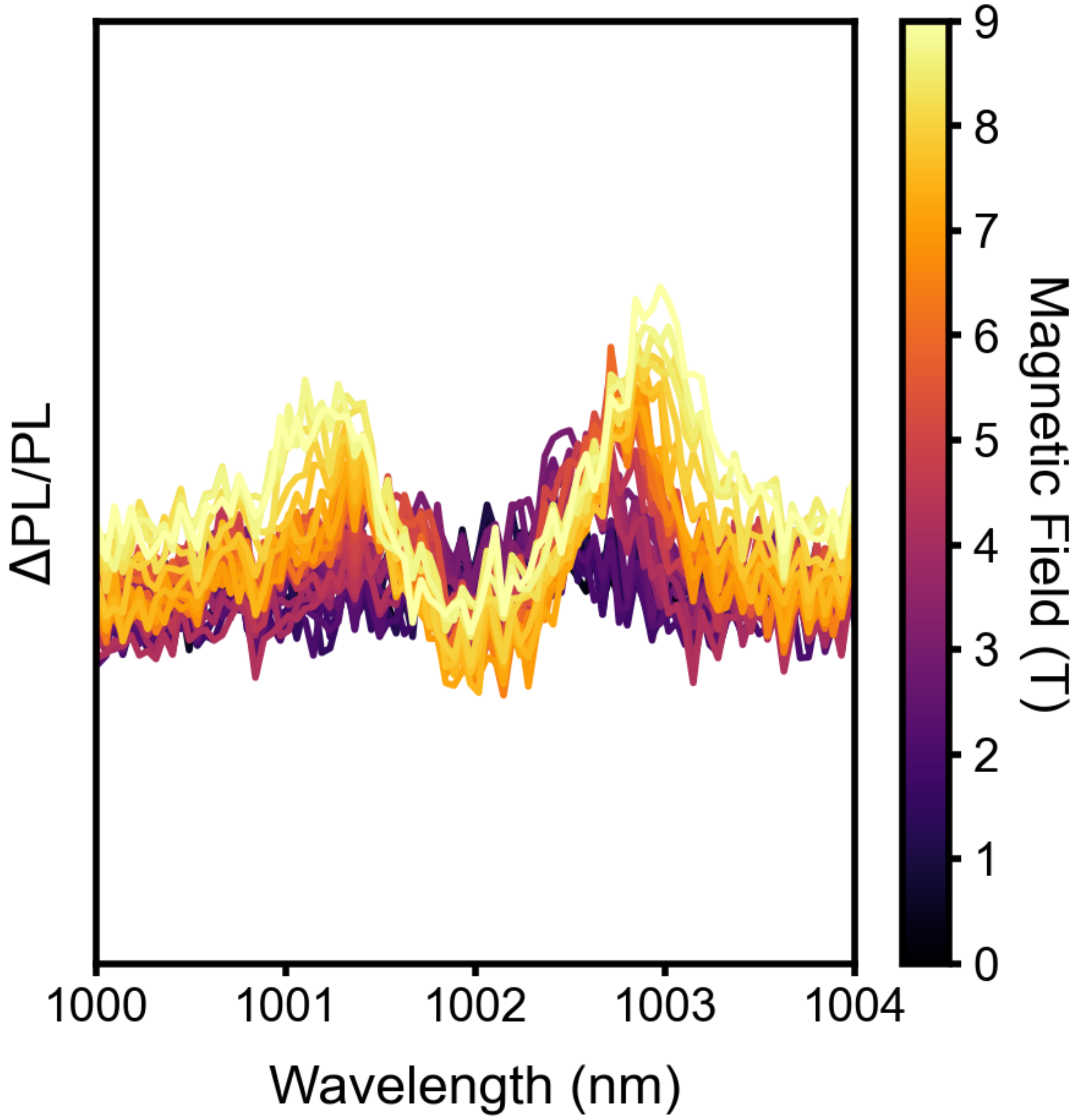

Figure S67 | Variable-field PL collected on ZPL of **2′** with 180-second exposure time, 24.8 mW of 660 nm excitation, a slit width of 50 µm, and a grating density of 1200 g/mm at 1.8 K.

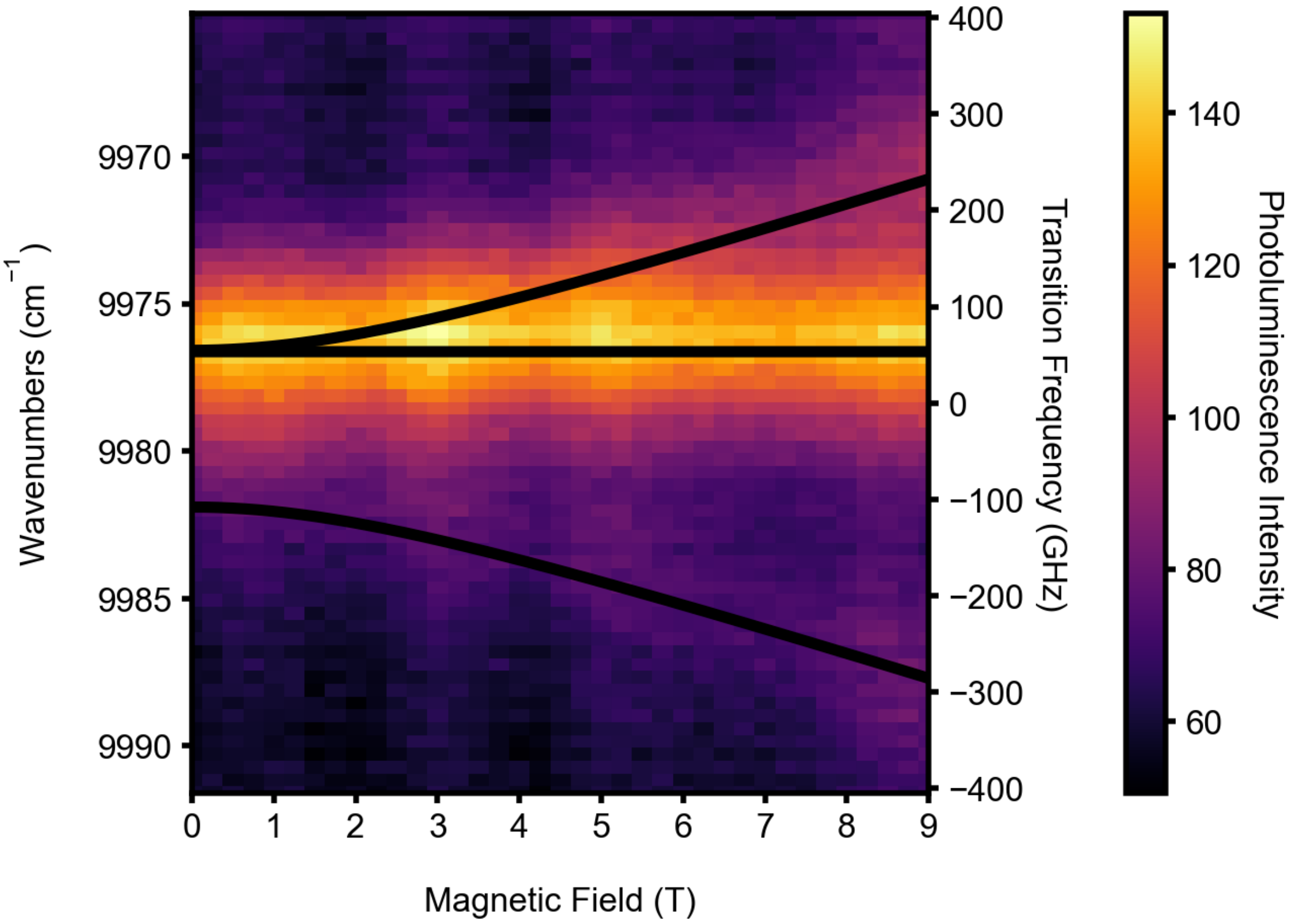

Figure S68 | Variable-field PL collected on ZPL of **2′** with 180-second exposure time, 24.8 mW of 660 nm excitation, a slit width of 50 µm, and a grating density of 1200 g/mm at 1.8 K. Zeeman diagram was modeled with $D = +5.44$ cm^{-1}, $g_{iso} = 1.959$, and $\theta = 90^{\circ}$.

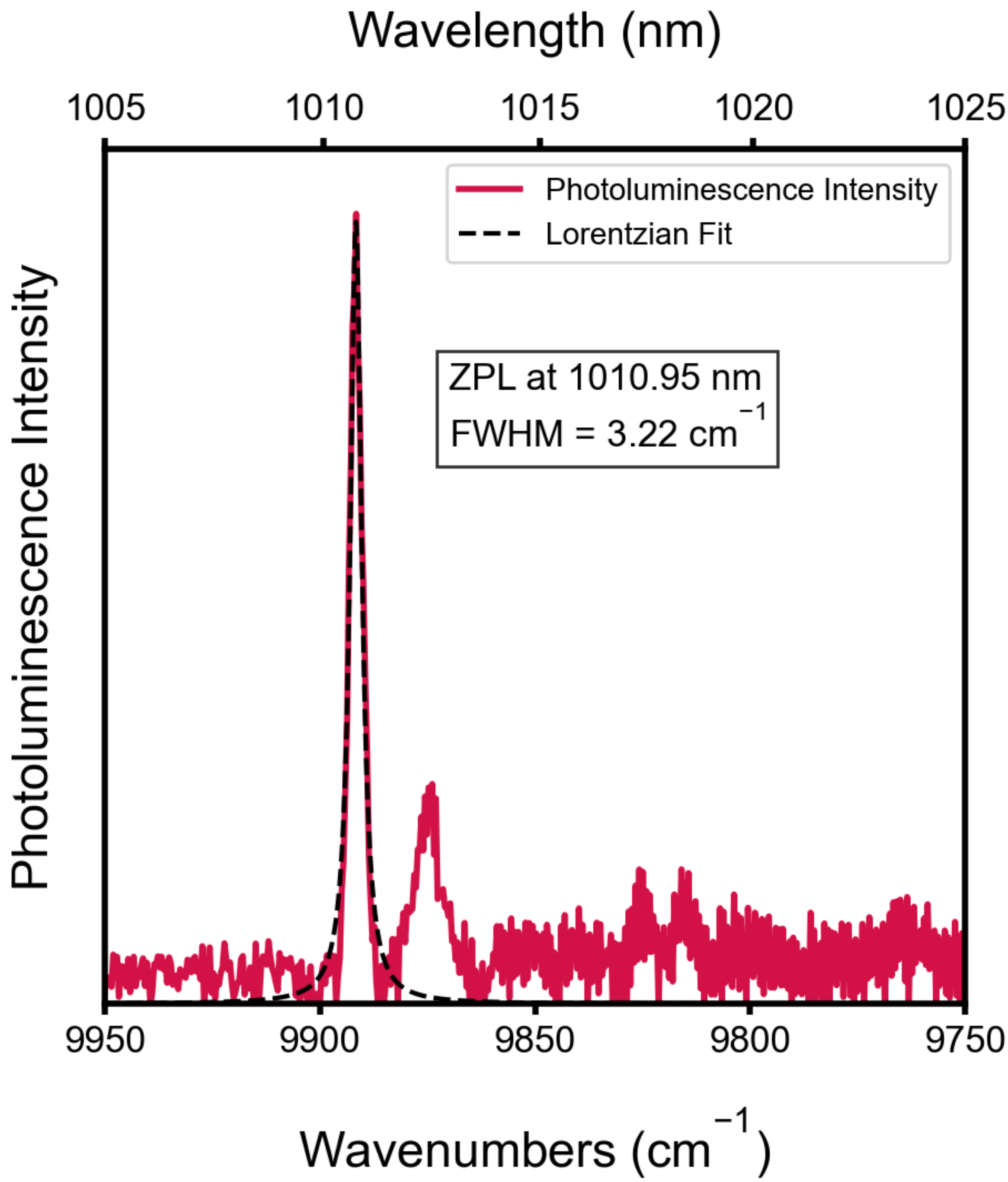

Figure S69 | PL collected on ZPL of **3′** with 180-second exposure time, 10 mW of 660 nm excitation, a slit width of 50 μm, and a grating density of 1200 g/mm at 1.8 K.

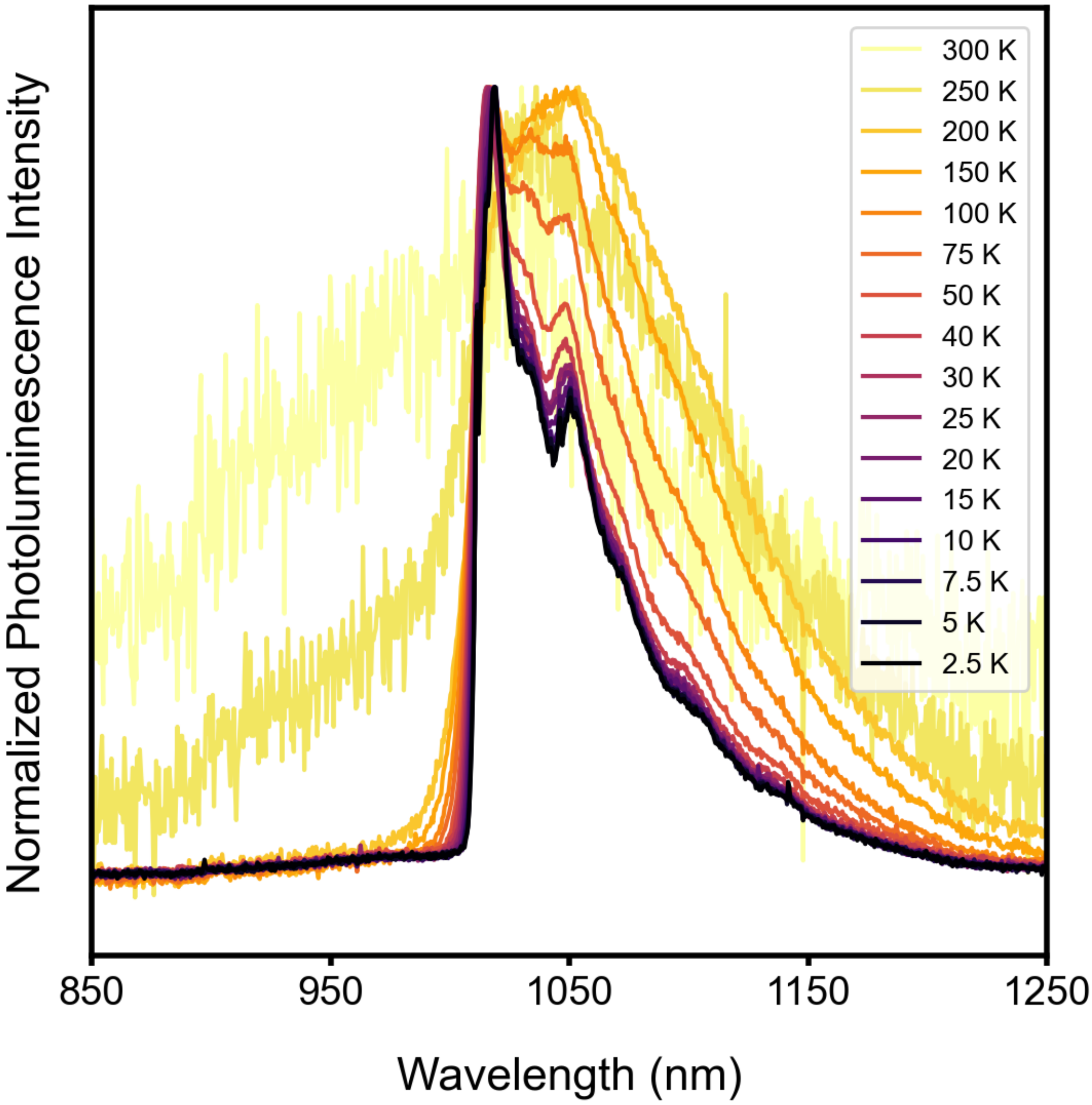

Figure S70 | Variable-temperature PL collected on **3′** with 60-second exposure time, 10 mW of 660 nm excitation, a slit width of 50 µm, and a grating density of 150 g/mm.

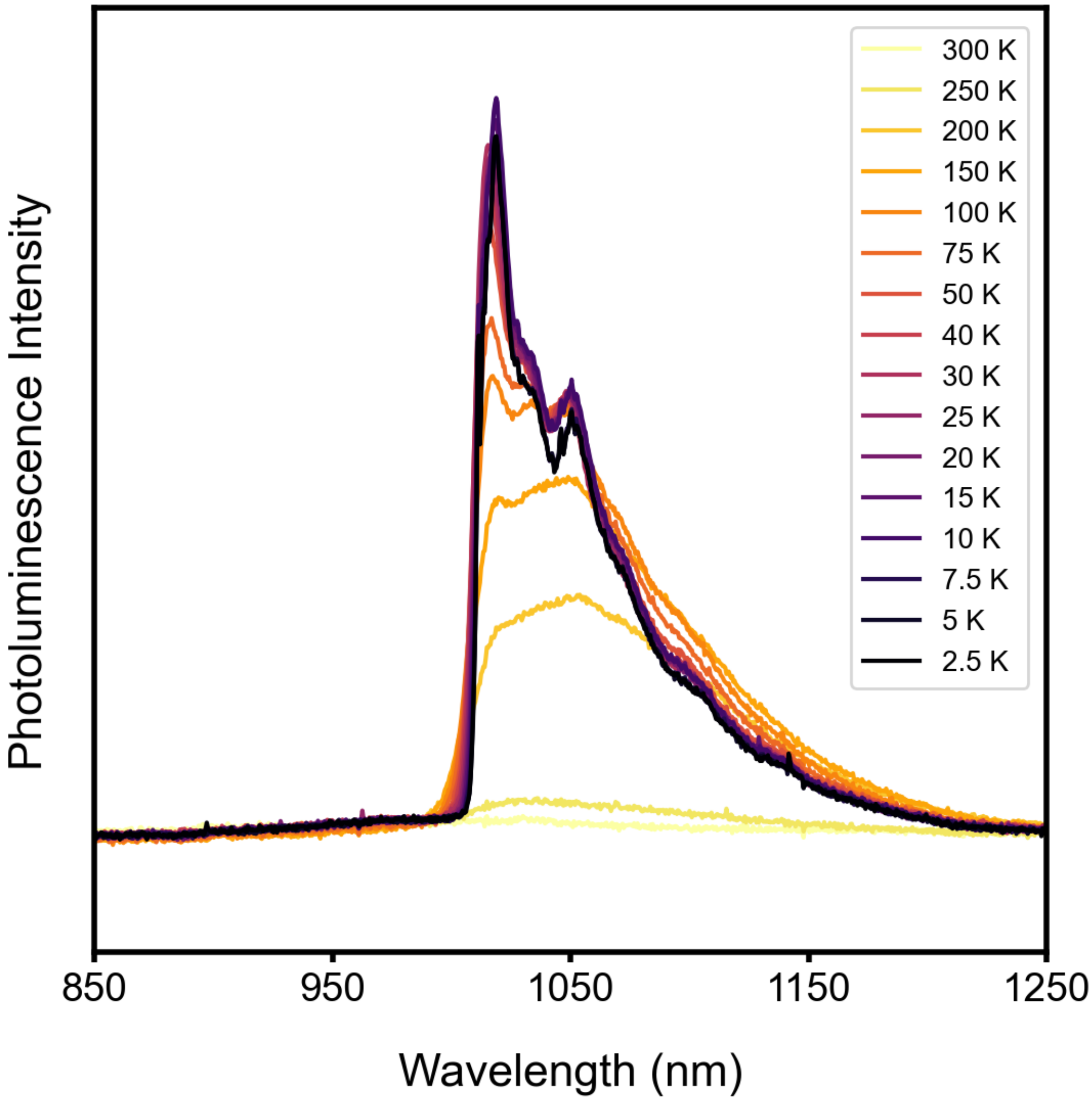

Figure S71 | Variable-temperature PL collected on **3′** with 60-second exposure time, 10 mW of 660 nm excitation, a slit width of 50 µm, and a grating density of 150 g/mm.

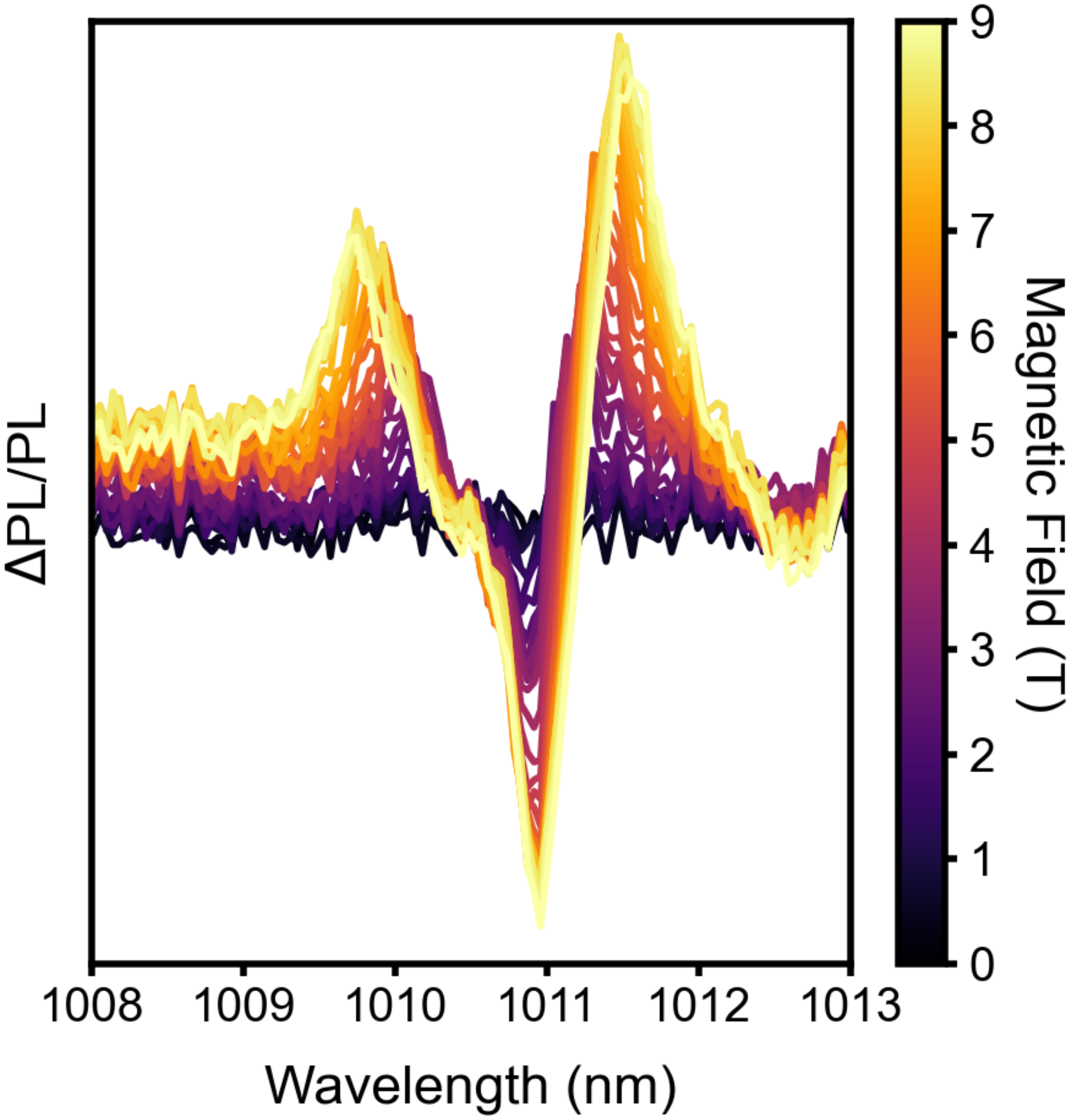

Figure S72 | Variable-field PL collected on ZPL of **3′** with 180-second exposure time, 10 mW of 660 nm excitation, a slit width of 50 μm, and a grating density of 1200 g/mm at 1.8 K.

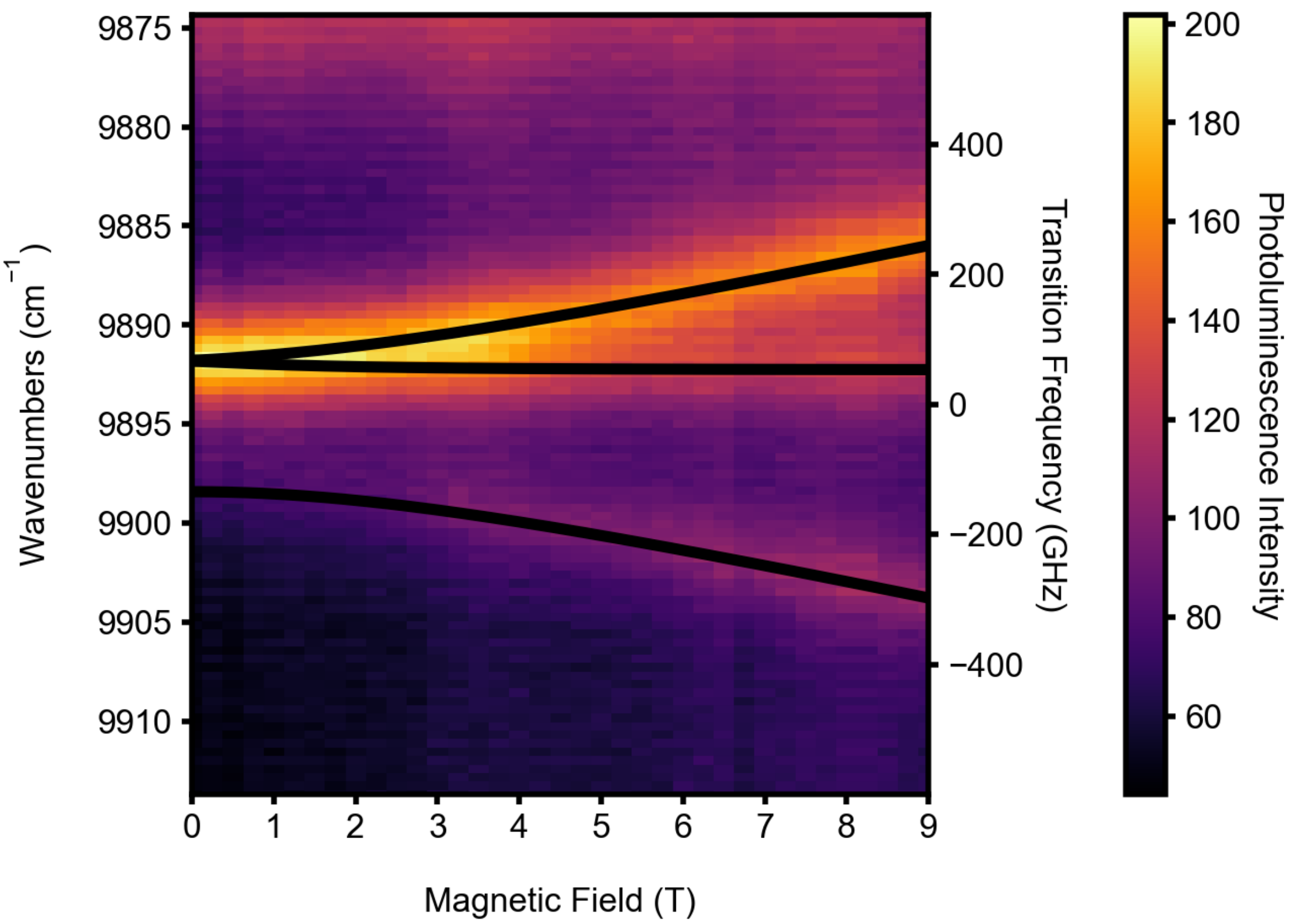

Figure S73 | Variable-field PL collected on ZPL of **3′** with 180-second exposure time, 10 mW of 660 nm excitation, a slit width of 50 µm, and a grating density of 1200 g/mm at 1.8 K. Zeeman diagram was modeled with D = +6.72 cm^{-1}, g_{iso} = 1.978, and θ = 75°.

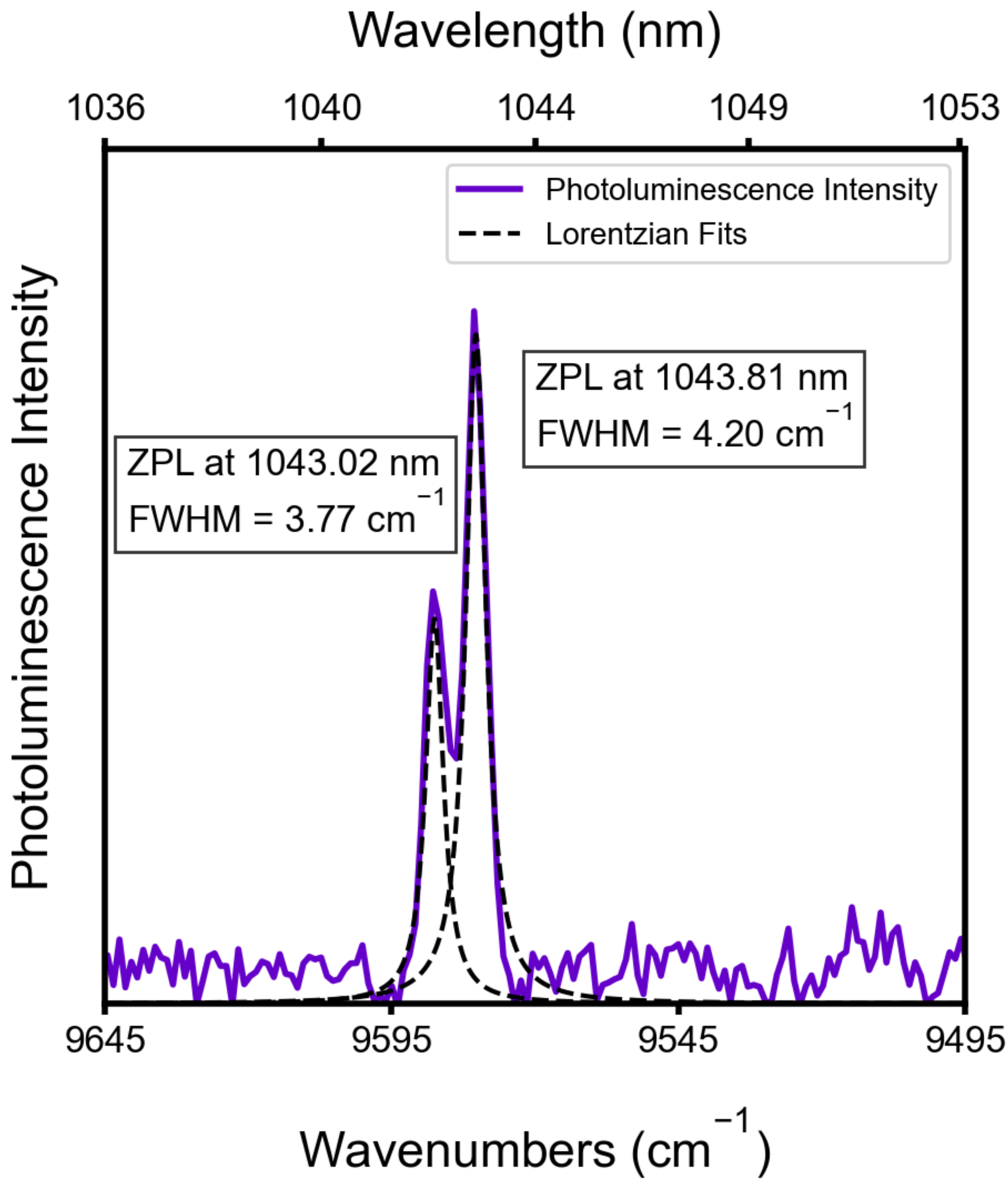

Figure S74 | PL collected on ZPL of **4′** with 180-second exposure time, 10 mW of 660 nm excitation, a slit width of 50 µm, and a grating density of 1200 g/mm at 1.8 K.

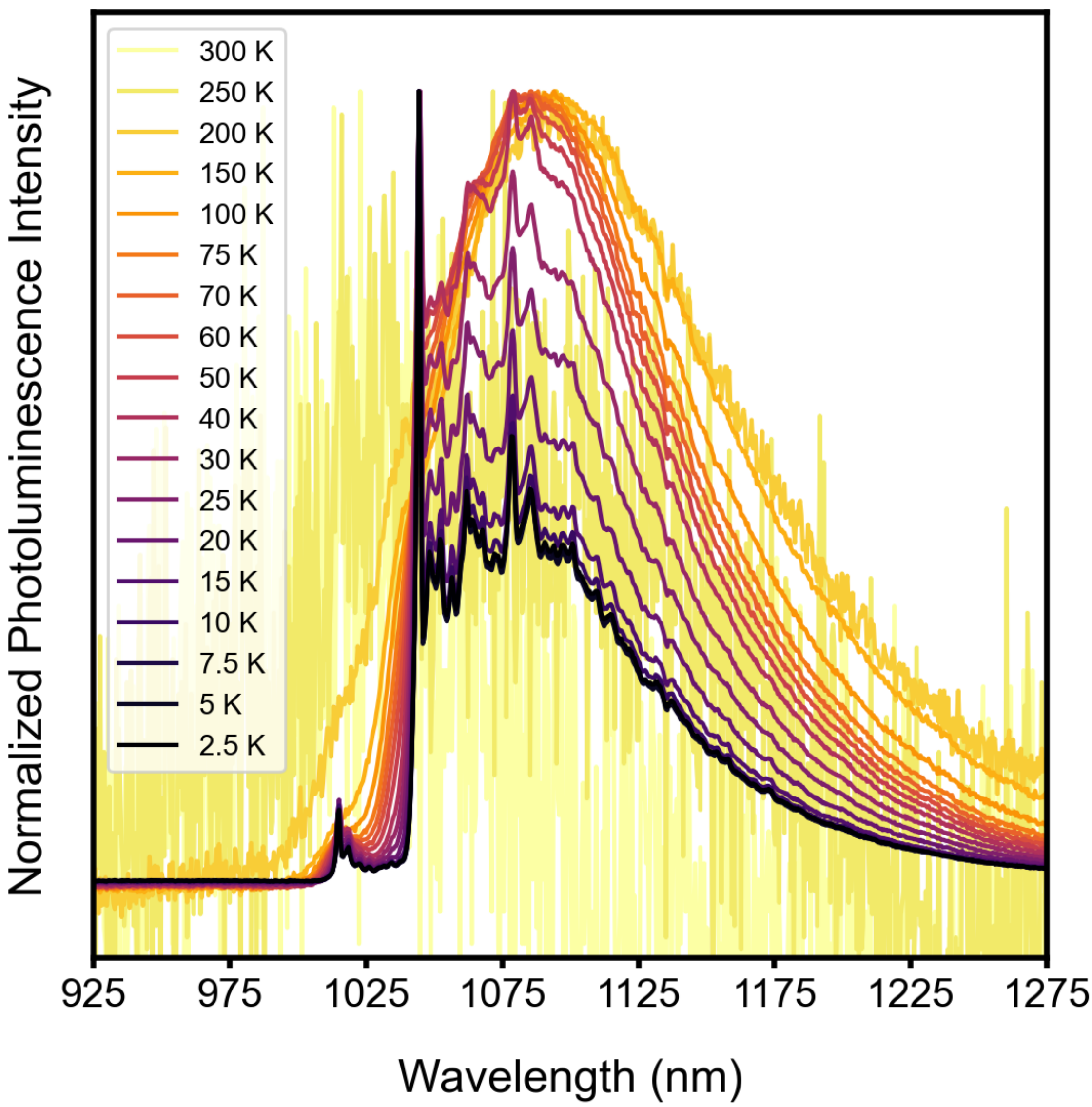

Figure S75 | Variable-temperature PL collected on **4′** with 30-second exposure time, 4 mW of 660 nm excitation, a slit width of 50 µm, and a grating density of 150 g/mm.

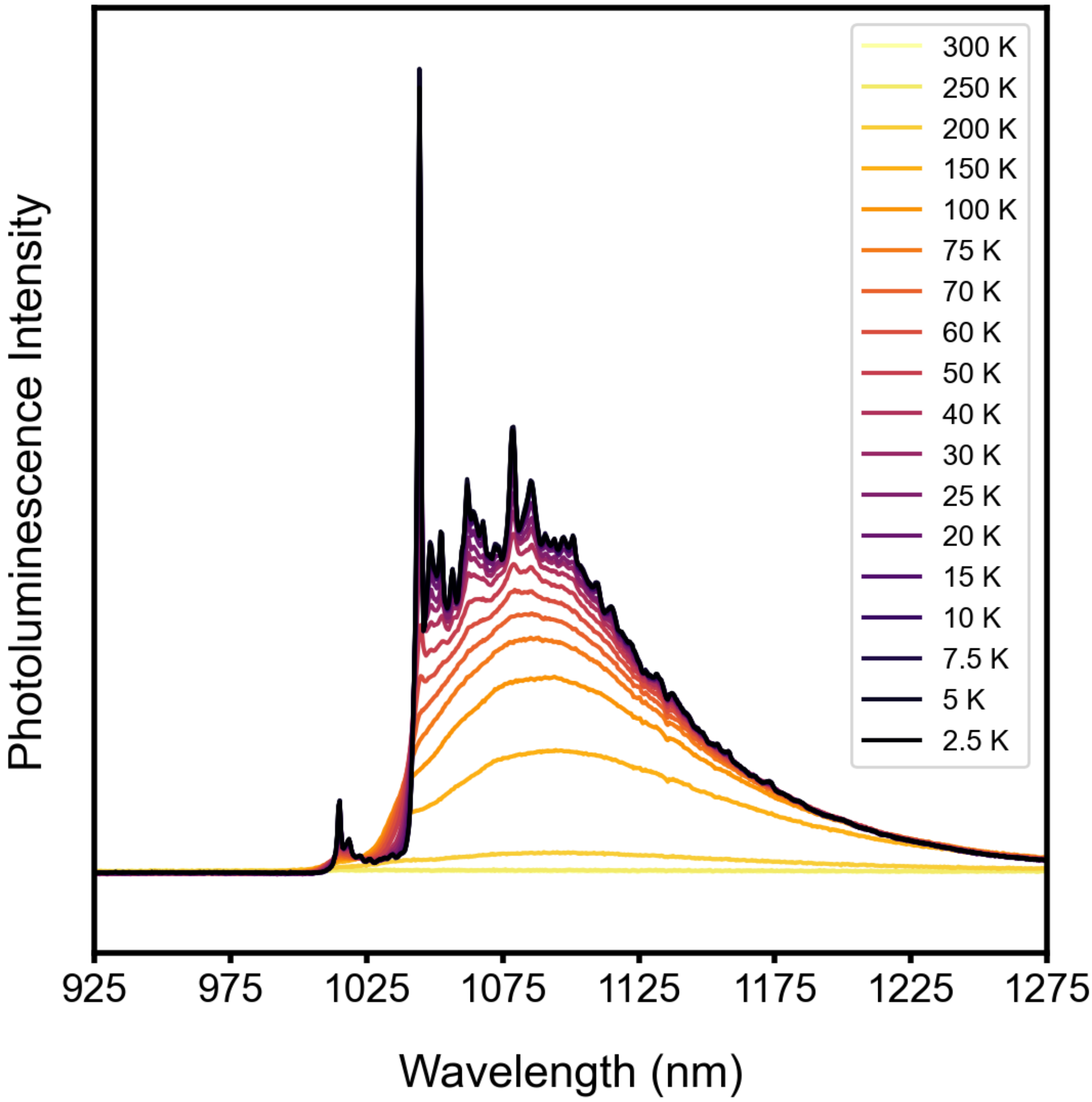

Figure S76 | Variable-temperature PL collected on **4′** with 30-second exposure time, 4 mW of 660 nm excitation, a slit width of 50 µm, and a grating density of 150 g/mm.

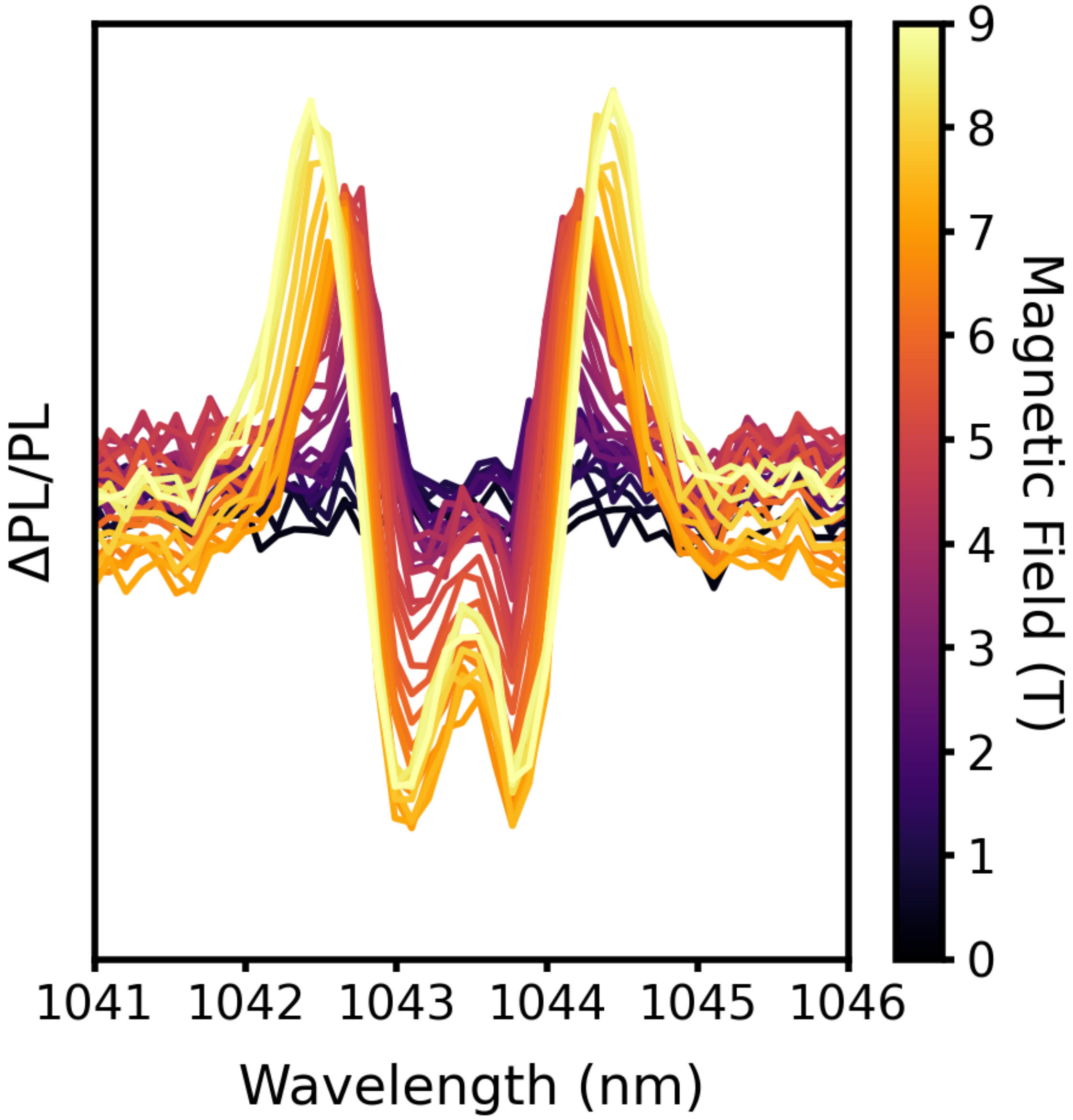

Figure S77 | Variable-field PL collected on ZPL of **4′** with 180-second exposure time, 4 mW of 660 nm excitation, a slit width of 50 µm, and a grating density of 1200 g/mm at 1.8 K.

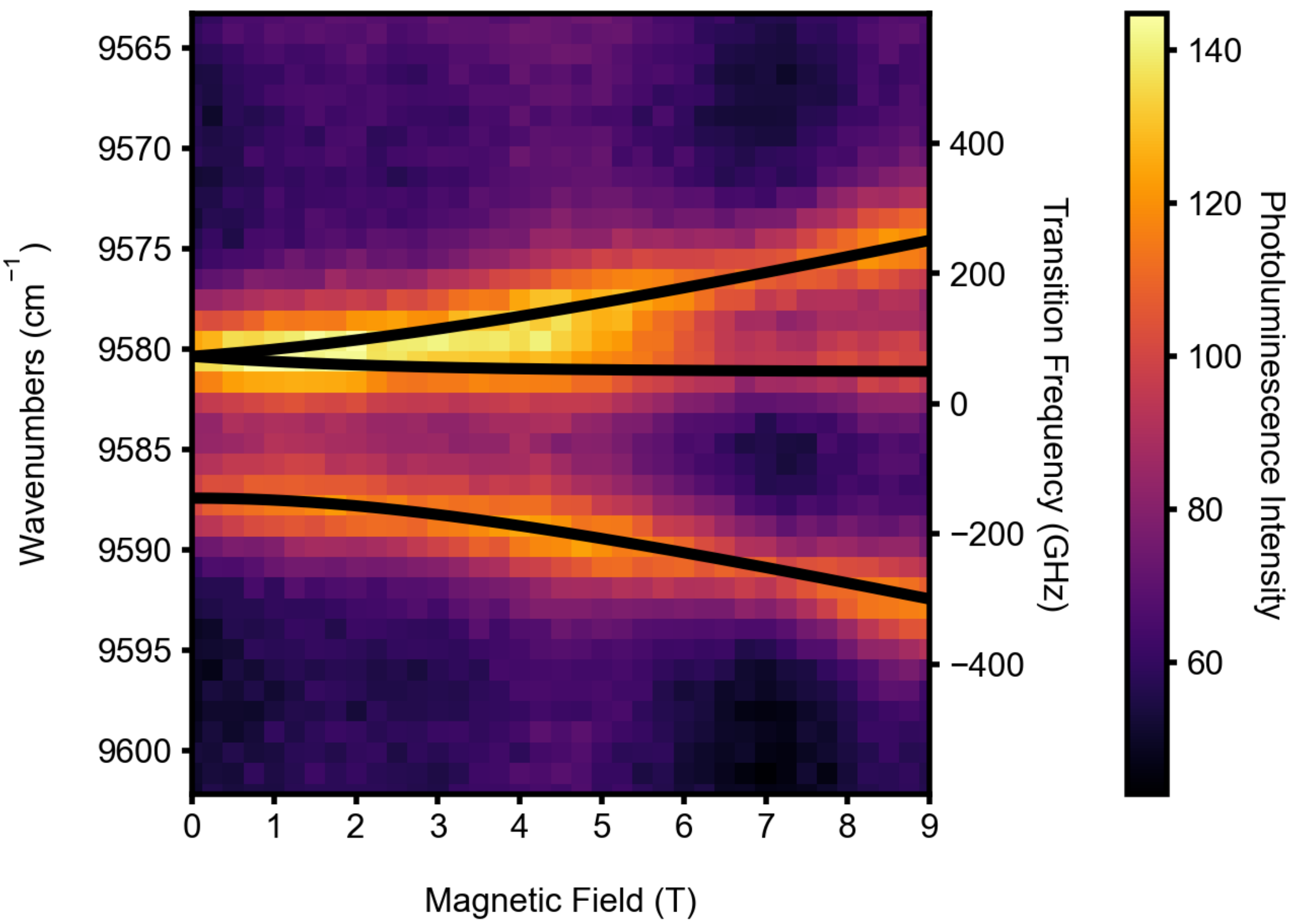

Figure S78 | Variable-field PL collected on ZPL of **4′** with 180-second exposure time, 4 mW of 660 nm excitation, a slit width of 50 µm, and a grating density of 1200 g/mm at 1.8 K. Zeeman diagram was modeled with D = +7.26 cm^{-1}, g_{xy} = 1.990, g_z = 1.954, and θ = 70˚.

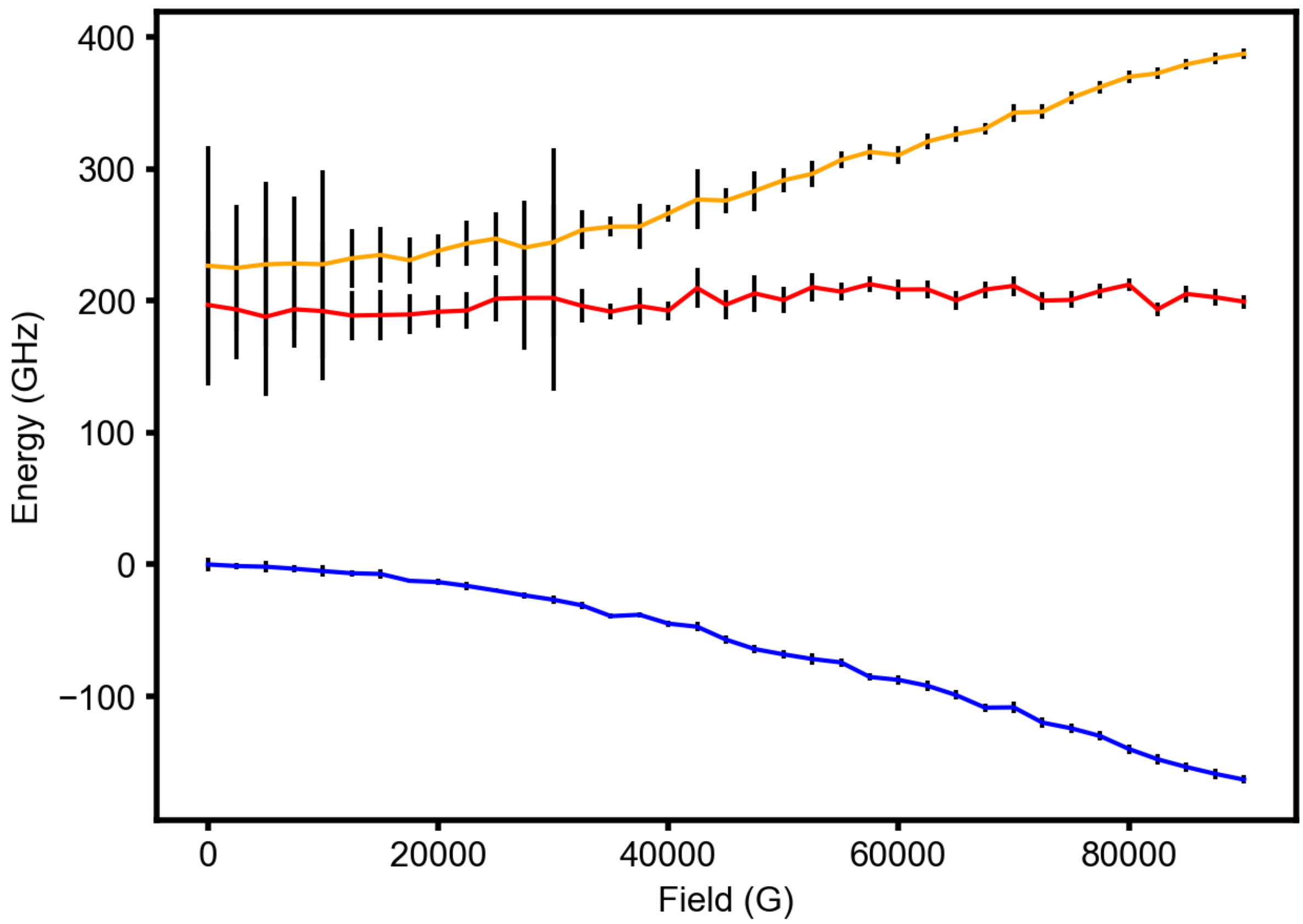

Figure S79 | Peak centers obtained from freely fitting VFPL collected on ZPL of **4′** with three Lorentzians (yellow, red, blue lines), and associated standard deviation error (black lines) from 0–9 T with 0.25 T increments.

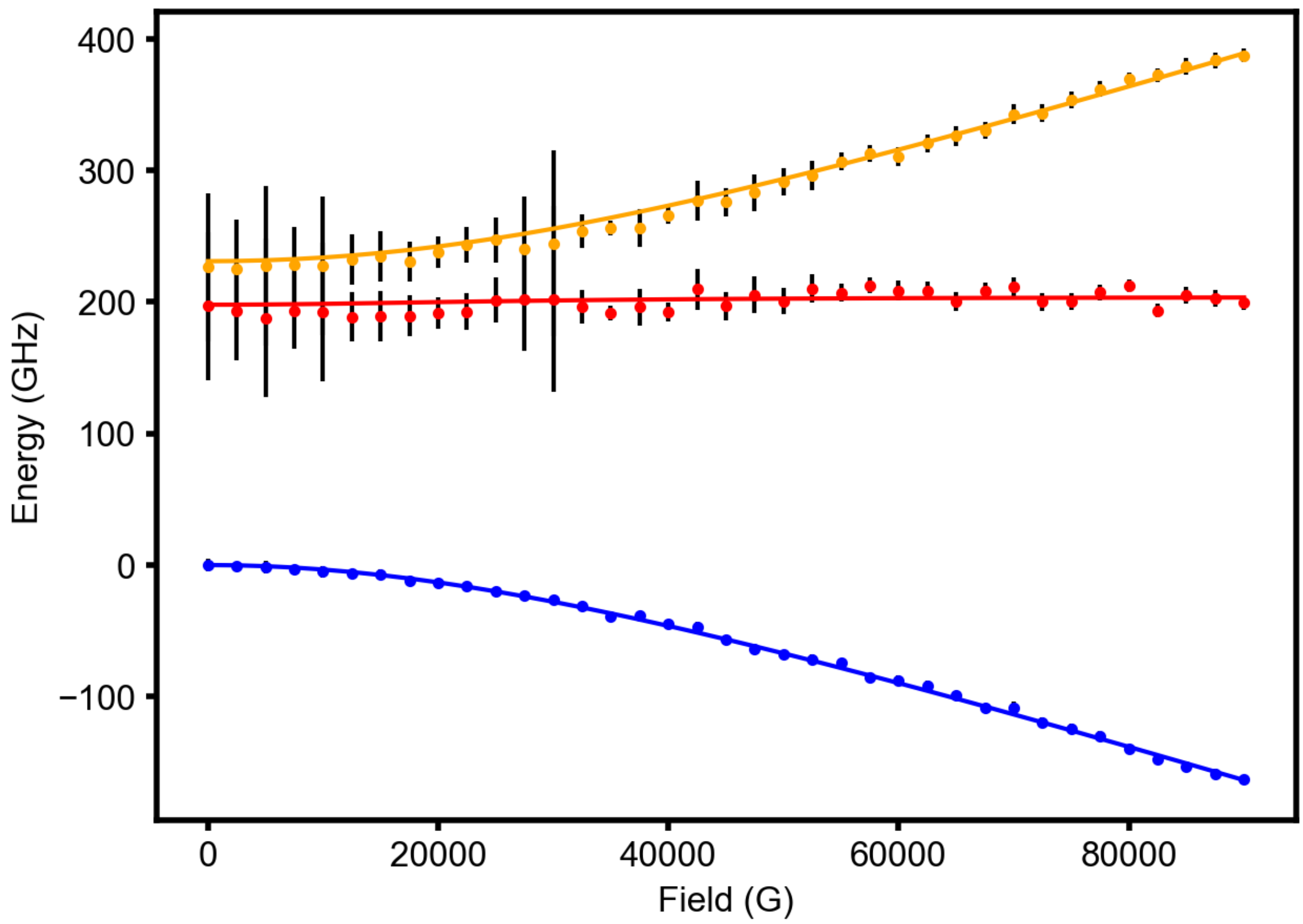

Figure S80 | Zeeman fit of peak centers obtained from fitting VFPL collected on ZPL of **4′**.

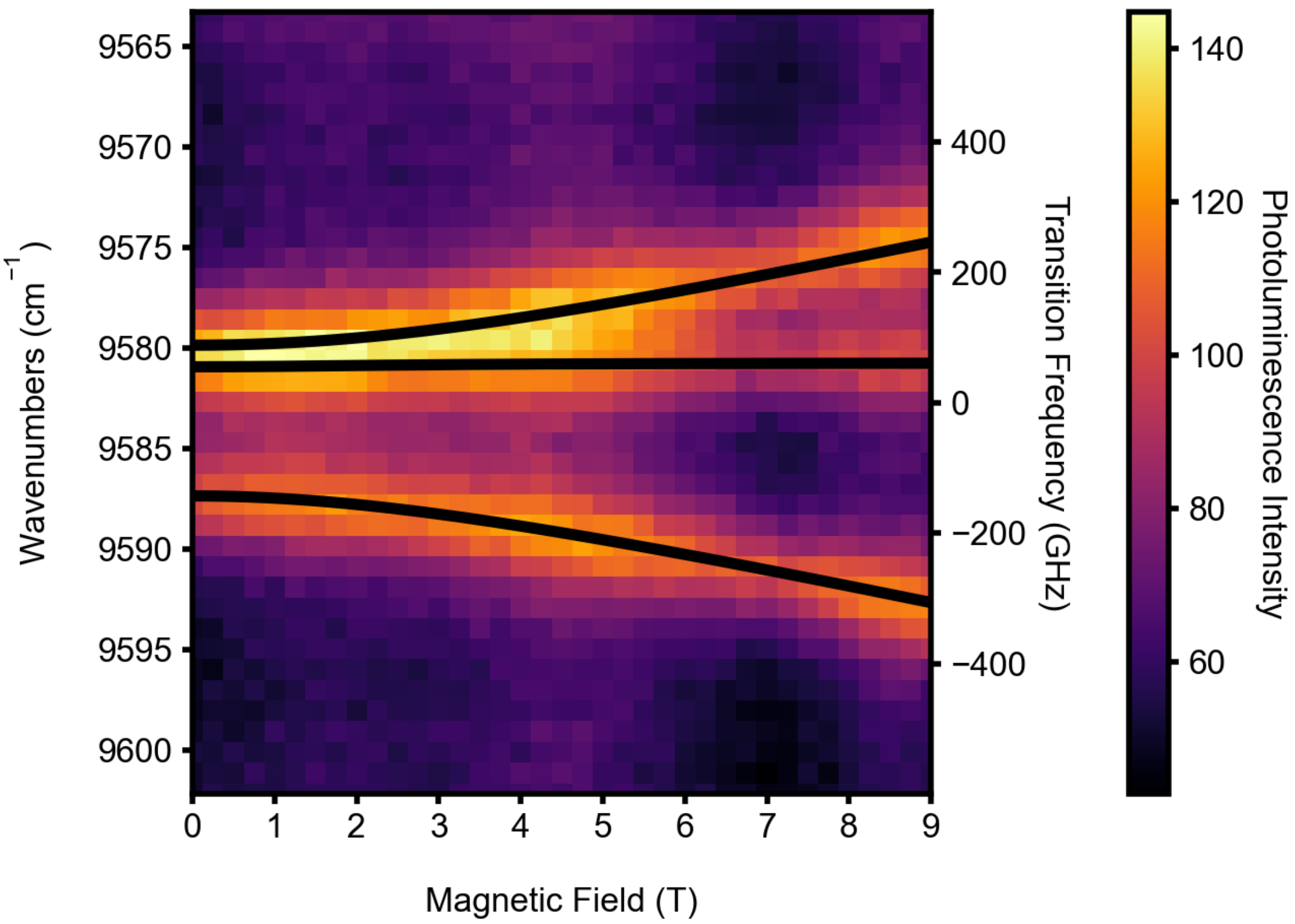

Figure S81 | Variable-field PL collected on ZPL of **4′** with 180-second exposure time, 4 mW of 660 nm excitation, a slit width of 50 μm, and a grating density of 1200 g/mm at 1.8 K. Zeeman diagram was modeled with parameters obtained from fitting peak centers.

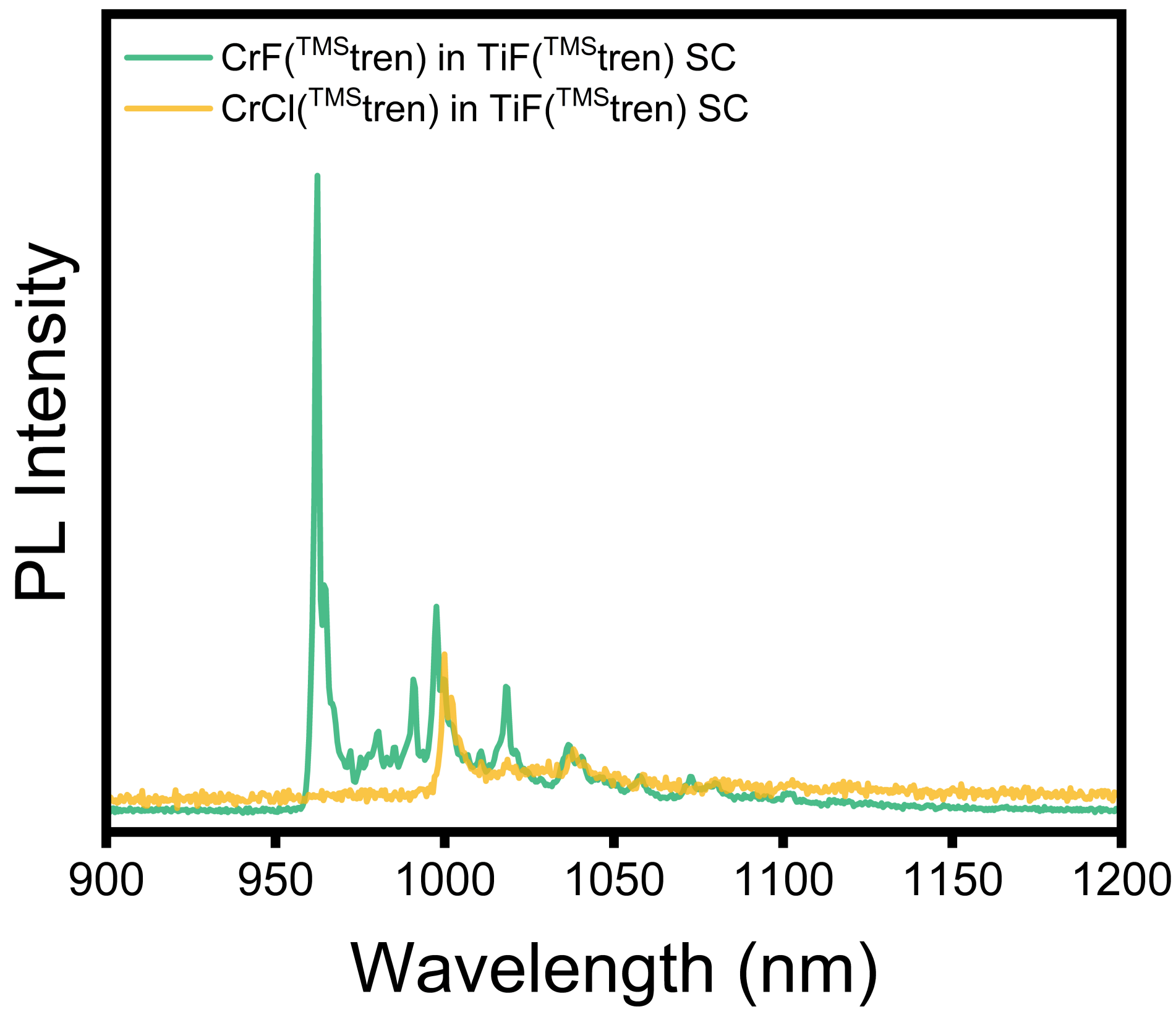

Figure S82 | PL collected on **1′** (green) overlayed with **2** diluted in **5** (yellow).

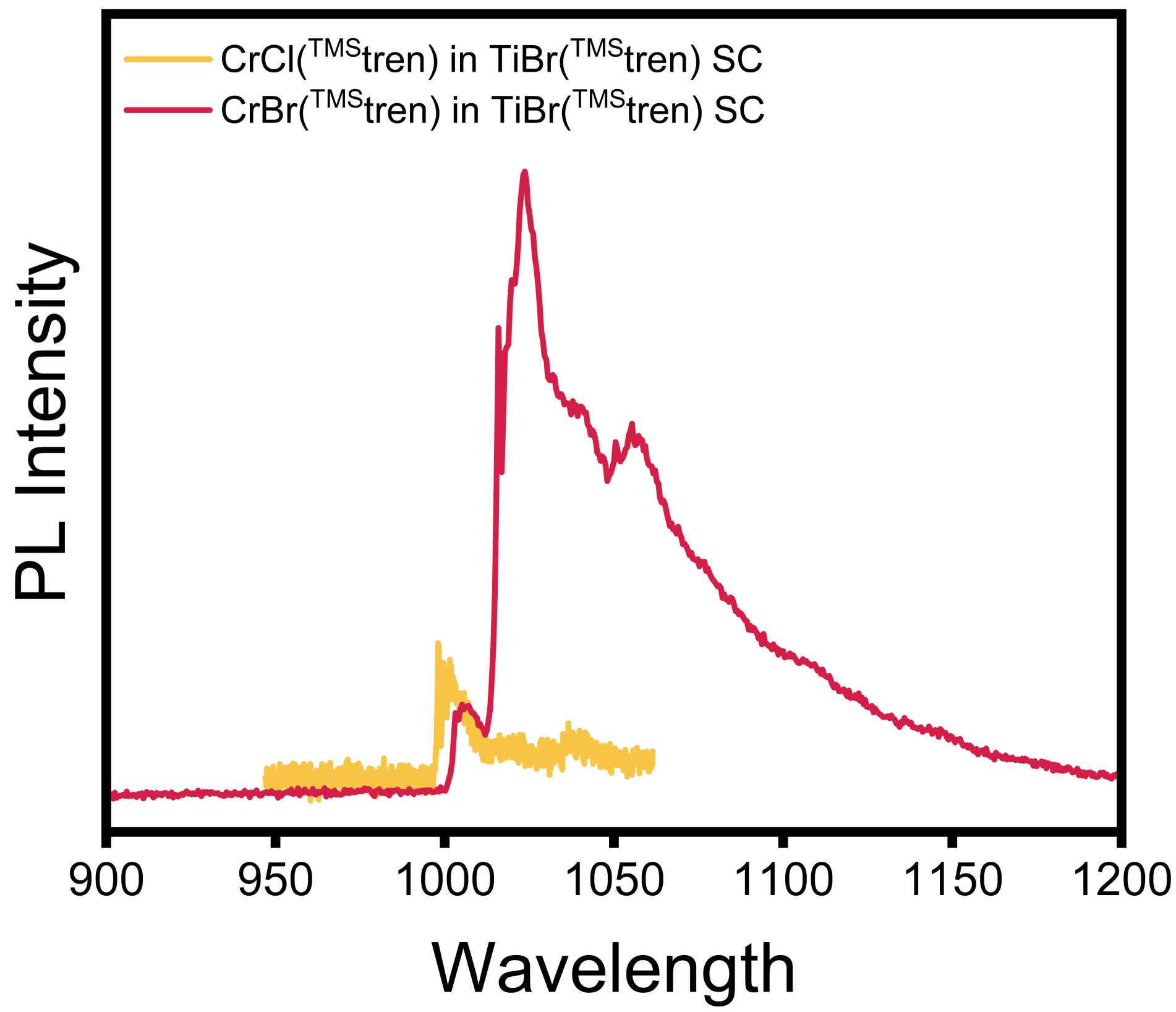

Figure S83 | PL collected on **3′** (red) overlayed with **2** diluted in **7** (yellow).

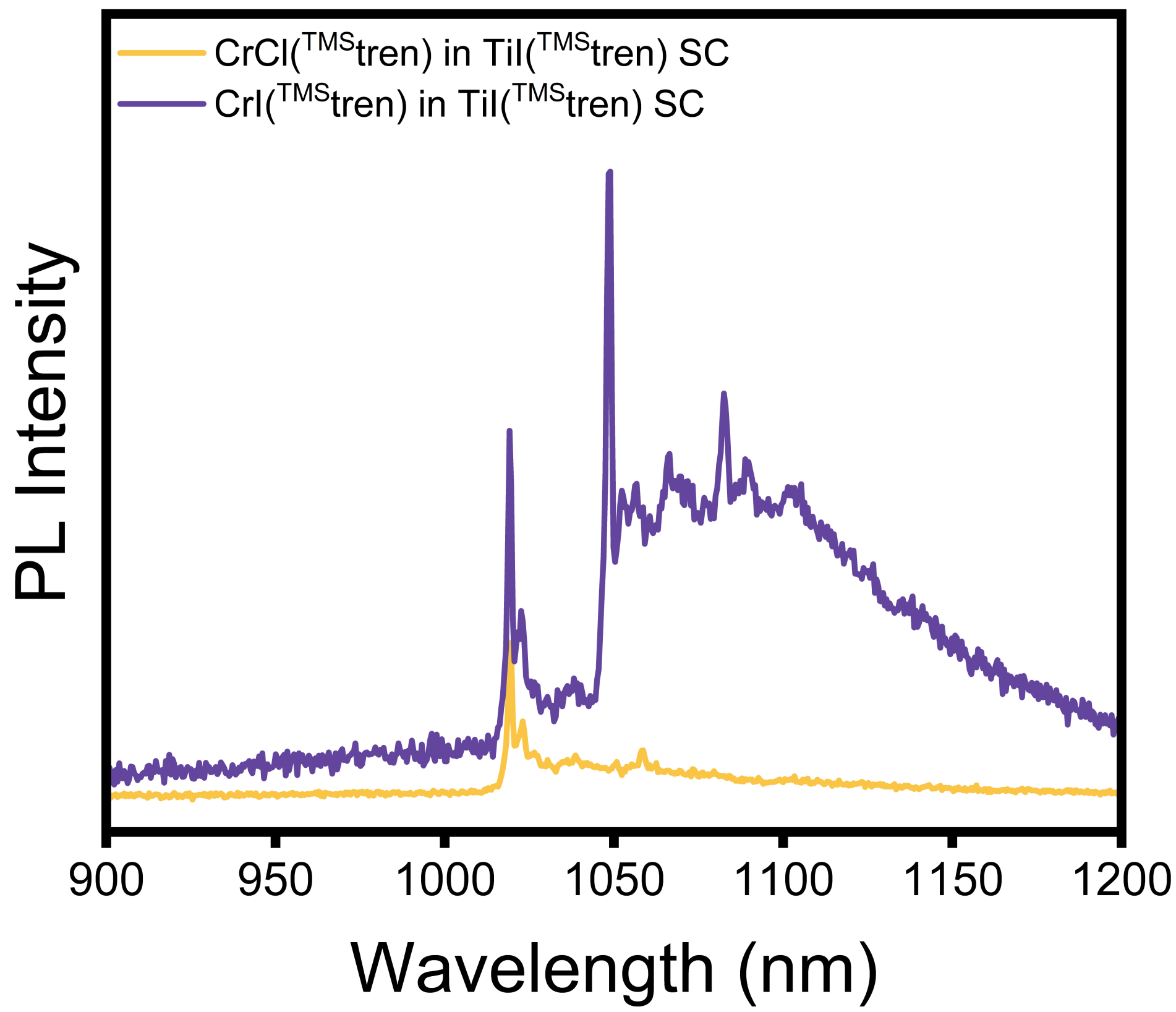

Figure S84 | PL collected on **4′** (purple) overlayed with **2** diluted in **8** (yellow).

References

(1) Boudjouk, P.; So, J.-H.; Ackermann, M. N.; Hawley, S. E.; Turk, B. E. Solvated and Unsolvated Anhydrous Metal Chlorides from Metal Chloride Hydrates. In *Inorg. Synth.*; John Wiley & Sons, Ltd; pp 108–111.

(2) Manxzer, L. E.; Deaton, J.; Sharp, P.; Schrock, R. R. 31. Tetrahydrofuran Complexes of Selected Early Transition Metals. In *Inorganic Syntheses*; John Wiley & Sons, Ltd; pp 135–140.

(3) Jura, M.; Levason, W.; Petts, E.; Reid, G.; Webster, M.; Zhang, W. Taking TiF4 Complexes to Extremes - the First Examples with Phosphine Co-Ligands. *Dalton Trans.* **2010**, *39*, 10264–10271.

(4) Gudat, Dietrich.; Verkade, J. G. New Azasilatranes: Synthesis and Substitution Reactions. *Organometallics* **1989**, *8*, 2772–2779.

(5) Cummins, C. C.; Schrock, R. R.; Davis, W. M. Synthesis of Terminal Vanadium(V) Imido, Oxo, Sulfido, Selenido, and Tellurido Complexes by Imido Group or Chalcogenide Atom Transfer to Trigonal Monopyramidal V[N3N] (N3N = $[(Me_3SiNCH_2CH2)_3N]^{3-}$). *Inorg. Chem.* **1994**, *33*, 1448–1457.

(6) Filippou, A. C.; Schneider, S.; Ziemer, B. Syntheses and Molecular Structures of Chromium(IV) Halides and Pseudohalides Bearing a Triamidoamine Ligand. *Eur. J. Inorg. Chem.* **2002**, *2002*, 2928–2935.

(7) Schneider, S.; Filippou, A. C. Triamidoamine Complexes of Chromium(III) and Chromium(IV). *Inorg. Chem.* **2001**, *40*, 4674–4677.

(8) Cummins, C. C.; Schrock, R. R.; Davis, W. M. Synthesis of Vanadium and Titanium Complexes of the Type $RM[(Me_3SiNCH_2CH_2)_3N]$ (R = Cl, Alkyl) and the Structure of $ClV[(Me_3SiNCH_2CH_2)_3N]$. *Organometallics* **1992**, *11*, 1452–1454.

(9) STOE (2023). X-Area, Chicago, IL, USA.

(10) Coppens, P. The Evaluation of Absorption and Extinction in Single-Crystal Structure Analysis. Crystallographic Computing. **1979**, No.

(11) Koziskova, J.; Hahn, F.; Richter, J.; Kožíšek, J. Comparison of Different Absorption Corrections on the Model Structure of Tetrakis(M2-Acetato)-Diaqua-Di-Copper(II). *Acta Chim. Slov.* **2016**, *9*, 136–140.

(12) Bruker (2015). SADABS. Bruker AXS Inc., Madison, Wisconsin, USA.

(13) Krause, L.; Herbst-Irmer, R.; Sheldrick, G. M.; Stalke, D. Comparison of Silver and Molybdenum Microfocus X-Ray Sources for Single-Crystal Structure Determination. *J. Appl. Crystallogr.* **2015**, *48*, 3–10.

(14) Dolomanov, O. V.; Bourhis, L. J.; Gildea, R. J.; Howard, J. a. K.; Puschmann, H. OLEX2: A Complete Structure Solution, Refinement and Analysis Program. *J. Appl. Crystallogr.* **2009**, *42*, 339–341.

(15) Sheldrick, G. M. SHELXT – Integrated Space-Group and Crystal-Structure Determination. *Acta Crystallogr., Sect. A: Found. Crystallogr.* **2015**, *71*, 3–8.

(16) Sheldrick, G. M. Crystal Structure Refinement with SHELXL. *Acta Crystallogr., Sect. C: Cryst. Struct. Commun.* **2015**, *71*, 3–8.

(17) van Tol, J.; Brunel, L.-C.; Wylde, R. J. A Quasioptical Transient Electron Spin Resonance Spectrometer Operating at 120 and 240 GHz. *Rev. Sci. Instrum.* **2005**, *76*, 074101.

(18) The MathWorks Inc. (2012). MATLAB (R2012b), Natick, MA, USA.

(19) Stoll, S.; Schweiger, A. EasySpin, a Comprehensive Software Package for Spectral Simulation and Analysis in EPR. *J. Magn. Reson.* **2006**, *178*, 42–55.

(20) morganbye. EPRtoolbox. https://sourceforge.net/projects/eprtoolbox/.

(21) Ozarowski, A. EPR Simulation Program for S>1/2. https://osf.io/z72tg/.

(22) Neese, F. An Improvement of the Resolution of the Identity Approximation for the Formation of the Coulomb Matrix. *J. Comput. Chem.* **2003**, *24*, 1740–1747.

(23) Helmich-Paris, B.; de Souza, B.; Neese, F.; Izsák, R. An Improved Chain of Spheres for Exchange Algorithm. *J. Chem. Phys.* **2021**, *155*, 104109.

(24) Humphrey, W.; Dalke, A.; Schulten, K. VMD: Visual Molecular Dynamics. *J. Mol. Graph.* **1996**, *14*, 33–38.

(25) Runge, E.; Gross, E. K. U. Density-Functional Theory for Time-Dependent Systems. *Phys. Rev. Lett.* **1984**, *52*, 997–1000.

(26) Martin, R. L. Natural Transition Orbitals. *J. Chem. Phys.* **2003**, *118*, 4775–4777.
(27) Addison, A. W.; Rao, T. N.; Reedijk, J.; Rijn, J. van; Verschoor, G. C. Synthesis, Structure, and Spectroscopic Properties of Copper(II) Compounds Containing Nitrogen–Sulphur Donor Ligands; the Crystal and Molecular Structure of Aqua[1,7-Bis(N-Methylbenzimidazol-2′-Yl)-2,6-Dithiaheptane]Copper(II) Perchlorate. *J. Chem. Soc., Dalton Trans.* **1984**, No., 1349–1356.
(28) Alvarez, S. SHAPE, 2.1.
(29) Figgis, B. N.; Hitchman, M. A. *Ligand Field Theory and Its Applications*; Special Topics in Inorganic Chemistry; Wiley.
(30) Whangbo, M.-H.; Gordon, E. E.; Xiang, H.; Koo, H.-J.; Lee, C. Prediction of Spin Orientations in Terms of HOMO–LUMO Interactions Using Spin–Orbit Coupling as Perturbation. *Acc. Chem. Res.* **2015**, *48*, 3080–3087.
(31) Xiang, H.; Lee, C.; Koo, H.-J.; Gong, X.; Whangbo, M.-H. Magnetic Properties and Energy-Mapping Analysis. *Dalton Trans.* **2013**, *42*, 823–853.
(32) Cahier, B.; Maurice, R.; Bolvin, H.; Mallah, T.; Guihéry, N. Tools for Predicting the Nature and Magnitude of Magnetic Anisotropy in Transition Metal Complexes: Application to Co(II) Complexes. *Magnetochemistry* **2016**, *2*, 31.
(33) Schweinfurth, D.; Sommer, M. G.; Atanasov, M.; Demeshko, S.; Hohloch, S.; Meyer, F.; Neese, F.; Sarkar, B. The Ligand Field of the Azido Ligand: Insights into Bonding Parameters and Magnetic Anisotropy in a Co(II)–Azido Complex. *J. Am. Chem. Soc.* **2015**, *137*, 1993–2005.
(34) Filippou, A. C.; Schneider, S.; Schnakenburg, G. A Pair of Remarkably Stable Mononuclear Chromium(III) and Chromium(IV) Hydrides. *Angew. Chem., Int. Ed.* **2003**, *42*, 4486–4489.
(35) Filippou, A. C.; Schneider, S. Unusually Stable Chromium(IV) Alkyls Bearing a Triamidoamine Ligand. *Organometallics* **2003**, *22*, 3010–3012.
(36) Sawka, J.; Bieńko, A.; Świtlicka, A.; Penkala, M.; Bieńko, D.; Rajnák, C.; Titiš, J.; Boča, R.; Ozarowski, A. Large Magnetic Anisotropy of Ni(II) Polynuclear Complexes Confirmed by Very Unusual HFEPR Spectra: Relaxation Behaviour of Six-Coordinate Ni(II) Dimers. *Dalton Trans.* **2025**, *54*, 13421–13425.
